# Evolution of microRNAs in Amoebozoa and implications for the origin of multicellularity

**DOI:** 10.1093/nar/gkae109

**Published:** 2024-02-20

**Authors:** Bart Edelbroek, Jonas Kjellin, Inna Biryukova, Zhen Liao, Torgny Lundberg, Angelika A Noegel, Ludwig Eichinger, Marc R Friedländer, Fredrik Söderbom

**Affiliations:** Department of Cell and Molecular Biology, Uppsala Biomedical Centre, Uppsala University, 75124 Uppsala, Sweden; Department of Cell and Molecular Biology, Uppsala Biomedical Centre, Uppsala University, 75124 Uppsala, Sweden; Science for Life Laboratory, The Department of Molecular Biosciences, The Wenner-Gren Institute, Stockholm University, 10691 Stockholm, Sweden; Department of Cell and Molecular Biology, Uppsala Biomedical Centre, Uppsala University, 75124 Uppsala, Sweden; Department of Cell and Molecular Biology, Uppsala Biomedical Centre, Uppsala University, 75124 Uppsala, Sweden; Centre for Biochemistry, Medical Faculty, University of Cologne, 50931 Cologne, Germany; Centre for Biochemistry, Medical Faculty, University of Cologne, 50931 Cologne, Germany; Science for Life Laboratory, The Department of Molecular Biosciences, The Wenner-Gren Institute, Stockholm University, 10691 Stockholm, Sweden; Department of Cell and Molecular Biology, Uppsala Biomedical Centre, Uppsala University, 75124 Uppsala, Sweden

## Abstract

MicroRNAs (miRNAs) are important and ubiquitous regulators of gene expression in both plants and animals. They are thought to have evolved convergently in these lineages and hypothesized to have played a role in the evolution of multicellularity. In line with this hypothesis, miRNAs have so far only been described in few unicellular eukaryotes. Here, we investigate the presence and evolution of miRNAs in Amoebozoa, focusing on species belonging to *Acanthamoeba*, *Physarum* and dictyostelid taxonomic groups, representing a range of unicellular and multicellular lifestyles. miRNAs that adhere to both the stringent plant and animal miRNA criteria were identified in all examined amoebae, expanding the total number of protists harbouring miRNAs from 7 to 15. We found conserved miRNAs between closely related species, but the majority of species feature only unique miRNAs. This shows rapid gain and/or loss of miRNAs in Amoebozoa, further illustrated by a detailed comparison between two evolutionary closely related dictyostelids. Additionally, loss of miRNAs in the *Dictyostelium discoideum drnB* mutant did not seem to affect multicellular development and, hence, demonstrates that the presence of miRNAs does not appear to be a strict requirement for the transition from uni- to multicellular life.

## Graphical Abstract

**Graphical Abstract:**
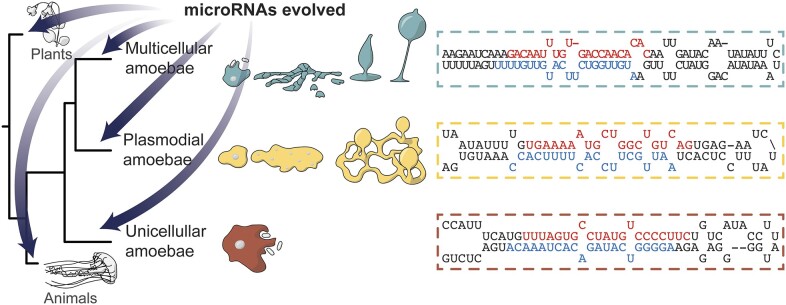

## Introduction

microRNAs (miRNAs) are small, ∼21 nucleotide (nt) non-coding RNAs (ncRNAs), which control gene expression to create intricate regulatory networks (1). The vast majority of miRNAs have been identified in animals and plants (2–7). In both these lineages, miRNA biogenesis involves transcription of larger primary, hairpin structured precursors (pri-miRNAs) that are processed to pre-miRNAs before being matured into miRNAs (8). This process is performed by a machinery derived from the ancestral RNA-interference (RNAi) machinery that dates back to the last eukaryotic ancestor and protects cells from viruses and mobilization of transposons (9–13). Once processed to mature miRNAs, these small RNAs specify genes to be regulated based on binding to their target mRNA(s) via sequence complementarity, thereby guiding the RNA induced silencing complex (RISC) to perform translational silencing and/or induce target RNA degradation (14). Even though miRNA maturation and formation of the RISC involve a number of associated proteins – to some extent differing between animal and plants – two proteins, Dicer and Argonaute, are central to a functional RNAi machinery regardless of organism. Dicer binds to miRNA hairpin structures and cleaves out double stranded miRNAs, consisting of a mir-5p and mir-3p duplex. Argonautes are the effector proteins, which select the miRNA (either the mir-5p or mir-3p) from the duplex. The mature miRNA then guides the Argonaute protein to the target RNA (14). Although the animal and plant miRNA machineries share many characteristics, miRNA regulation is generally thought to have evolved convergently. In particular, no miRNA sequences have been found to be conserved between plants and animals (2). The convergent evolution of miRNAs in plants and animals is, however, still up for debate (15,16).

It is perhaps no coincidence that the majority of miRNAs have been discovered in either plants or animals, where organisms show a high degree of organismal complexity (14,17). Both plants and animals independently evolved clonal multicellularity and regulatory genetic networks necessary for multicellular life (18). It is possible that miRNA-mediated gene regulation was already present during the transition from uni- to multicellularity, especially since miRNAs have been found in unicellular organisms of both the plant and animal lineages (19–22). Hence, miRNAs may have contributed in the evolution of multicellularity, but whether or not this is the case remains unclear (19,23,24).

To better address fundamental questions regarding the evolution of miRNAs and their contribution to multicellularity, stringent analysis of the small RNA populations in understudied eukaryotic lineages is needed (15). To our knowledge, only seven protists have been reported to carry miRNAs when considering a minimal set of established annotation criteria, i.e. (i) defined secondary structure of the hairpin with mapped mir-5p and mir-3p, (ii) evidence that the miRNA is precisely cleaved from the miRNA hairpin and no other small RNAs are generated and (iii) miRNA candidates are not derived from other structured ncRNAs. The protists that fulfill these criteria are two species of brown algae (24–26), a green alga (20,22,27), two dinoflagelates (28,29), one ichtyosporean species (19) and one social amoeba (30). The latter is part of the Amoebozoa taxonomic group, which belongs to the Amorphea supergroup that also includes animals and fungi (31). Species belonging to Amoebozoa exhibit diverse lifestyles and a varied level of developmental complexity. Some are strictly unicellular, such as the *Acanthamoeba* (32). Others, such as *Physarum polycephalum*, are unicellular but can switch from a free-living amoeboid to a plasmodial lifestyle in which it grows without cytokinesis. This results in a large intricate multinucleate network that permits sensing and transport of nutrients (33). Upon starvation, the plasmodium can differentiate to form sporangia (33). Another group of Amoebozoa is the Dictyostelia, estimated to date back 600 million years (34). Members of Dictyostelia switch from a unicellular life to an aggregative multicellular lifestyle upon starvation. Here, up to 100 000 cells stream together to form distinct multicellular structures, where cells can collaborate to move towards light (35). Finally, cells culminate to form a fruiting body (35). Even within the dictyostelids, the level of developmental complexity varies. Some, like *Acytostelium subglobosum*, form a fruiting body with one single cell type, while others, such as *Dictyostelium discoideum*, employ up to four major cell types. Dictyostelia has been subdivided into four monophyletic groups, and of these, group 4 contains some of the most developmentally complex dictyostelids (36). Members of this group have been observed to form migrating slugs where differentiated cells collaborate to perform phototaxis (37).

Thus far, *D. discoideum* is the only amoebozoan in which miRNAs have been convincingly identified (30,38–40). The *D. discoideum* miRNAs are mostly expressed from single intergenic regions and are upregulated upon multicellular development. They rely on the Dicer-like protein, DrnB, and a double-stranded RNA binding domain-containing protein RbdB for their processing (39,40). *D. discoideum* also encodes five Argonautes. Which one of these binds miRNAs is still unknown.

Here, we systematically investigated the small RNA repertoire of nine amoebozoan species to approach the evolutionary history of miRNAs. We also address the hypothesized role of miRNAs as a driver for the evolution of multicellularity. The amoebae we used were six dictyostelids, the closely related *P. polycephalum*, and two species belonging to *Acanthamoeba*. By using stringent miRNA identification criteria, high-confidence miRNAs were identified in all the multicellular dictyostelids, but also in *P. polycephalum* and the strictly unicellular *Acanthamoeba* species. One of the identified miRNAs is conserved within the lineage of *Acanthamoeba* and another is conserved between group 4 dictyostelids. No other conserved miRNAs were detected, and nearly all miRNAs appear to be recent innovations. The importance of miRNAs for unicellular life is indicated by the expression and conservation of miRNAs in amoebozoan species that lack a multicellular lifestyle, and is corroborated by the increased generation time of cells depleted of miRNAs in *D. discoideum*. Further, phylogenetic analysis of RNAi components shows a rapidly evolving and flexible system with their numbers varying between species. Together, our results indicate that miRNAs have evolved recently and rapidly throughout Amoebozoa, and that the miRNA regulatory networks are not exclusive to Amoebozoa that exhibit aggregative multicellularity.

## Materials and methods

### Strains and cell culture

Social amoebae were grown at 22°C on SM Agar/5 (Formedium) with 0.5% activated charcoal, in association with *Escherichia coli* 281. Cells were allowed to grow until they started clearing the plate from *E. coli*, but before starvation occurred, at which point they were harvested with a cell scraper (Thermo Scientific). Remaining *E. coli* were removed by four consecutive washes with KK2 buffer (2.2 g/l KH_2_PO_4_, 0.7 g/l K_2_HPO_4_) at 400×g, 5 min. One-third of the cells were resuspended in TRIzol Reagent (Invitrogen) and frozen at –20°C until further processing. The remaining two-thirds of the cells were seeded on non-nutrient agar with 0.5% activated charcoal to allow for starvation and subsequent development of the cells. One-third of the cells were harvested at the sorogen or slug stage (13–20 h post induction of starvation) and one-third of the cells at the sorocarp of fruiting body stage (23–40 h post induction of starvation) into TRIzol Reagent (Invitrogen) and frozen at –20°C. *Acanthamoeba* were first grown axenically in PYG w/ additives (ATTC medium 712 at 30°C) and subsequently grown and harvested similar to the social amoebae, on SM Agar/5 plates with charcoal, with *E. coli* 281 as food source. *Physarum polycephalum* was grown on oats on 2% agar. Parts of sclerotium were scraped from the plate and resuspended directly into TRIzol Reagent.

For the growth curves of *D. discoideum* wildtype (AX2) and *drnB*^−^ strains, cells were grown axenically in HL5-C (Formedium) with 100 μg/ml Pen Strep (Gibco) at 22°C, shaking at 180 rpm, and quantified in a hemocytometer. For strain numbers and access to strains see attributes file at SRA under BioProject accession number PRJNA972620.

### Knock out of RNAi machinery components in *D. discoideum*

Knockout strains of *agnB*, *agnC* and *agnE*, were prepared as described earlier for *drnB* (30). Briefly, two fragments of each gene were amplified from *D. discoideum* wildtype genomic DNA. For *agnB*, the fragments were amplified using primer pairs [p827–p828] and [p829–p830] (Supplementary Table S1), and these were inserted into a pLPBLP gene deletion vector (41). The *agnC* fragments were amplified with [p653–p654] and [p655–p656] primer pairs, and for *agnE*, [p657–p658] and [p659–p660] primer pairs were used (Supplementary Table S1), and fragments were inserted into a pKOSG gene deletion vector (42). Plasmids were transformed into XL10-Gold Ultracompetent Cells (Integrated Sciences) for amplification. For constructing gene knock-outs, 30 μg of the plasmids were digested to generate linearized blasticidin resistance casettes, flanked by the gene fragments. The *agnB*-pLPBLP plasmid was digested with KpnI and BcuI (Thermo Scientific), *agnC*-pKOSG and *agnE*-pKOSG were digested with PstI (Thermo Scientific). *D. discoideum* cells were prepared for transformation in the following way: 2 × 10^7^ cells were grown axenically in HL5-C (Formedium), harvested (300xg, 5 min), incubated in ice-old PDF buffer [20 mM KCl, 9.2 mM K_2_HPO_4_, 13.2 mM KH_2_PO_4_, 1 mM CaCl_2_, 2.5 mM MgSO_4_, pH 6.4] for 15 min, centrifuged at 300×g for 5 min and resuspended in [10 mM Na_2_PO_4_, 50 mM sucrose, 4°C]. The linearized knockout constructs were transformed by electroporation, and transformants were selected with 10 μg/ml blasticidin.

### Small RNA sequencing

In total, small RNA was sequenced from nine different species and three biological replicates from each species, which sums up to 27 sequenced small RNA libraries. RNA isolation was performed according to the TRIzol Reagent user guide (Invitrogen), except with an additional 75% EtOH wash of the RNA pellet. For social amoebae, RNA from different developmental stages was pooled using equal amounts of total RNA. Pooling of the different developmental stages provided a more managable number of libraries, allowing us to also sequence large number of small RNAs in biological replicates, which is important for stringent definition of new miRNAs. Small RNA sequencing libraries were generated according to the TruSeq Small RNA Library Prep Kit (Illumina). Libraries were sequenced on a NextSeq 500 System (Illumina). Development, RNA preparation and sequencing of the *drnB^−^*, *agnB^−^*, *agnC^−^* and *agnE^−^* knockout strains and wildtype (wt) was performed as in (39). For each of these strains, RNA was isolated from growing cells (three biological replicates) and 16h developed cells (two biological replicates) for a total of 25 libraries. Differential gene expression analysis was done in R using DESeq (43).

### miRNA identification

Quality control of raw sequencing reads was performed with miRTrace v1.0.1 (44), after which they were processed by trimming the sequencing adapters and selecting reads of size 18–35 nt with Cutadapt v4.4 (45). Small RNAs were mapped to the reference genome or *E. coli* genome, with a maximum of one mismatch, and multimapping reads placed according to the fractional weighted probability, using Shortstack v3.8.5 (46) (Supplementary Table S2). Additionally, using the same mapping strategy, all unmapped reads were cross mapped to the other amoeba genomes, but no contamination could be detected (Supplementary Table S2).

Clusters with potential miRNA candidates were selected and further analyzed with an in-house python script available on https://doi.org/10.5281/zenodo.7937209. Briefly, in the first round, exploratory analysis was done by mapping reads with no mismatches to each selected cluster and folding the cluster using the ViennaRNA RNAlib-2.6.2 python package (47), with standard settings and temperature of 22°C. If the cluster could be folded into a hairpin-structure, the positions of the mir-5p and mir-3p were defined based on the mapped reads. Clusters that yielded a hairpin and had at least a 30% 5′ precision of mir-5p and mir-3p were selected for a second round of analysis, similar to the first, but with additional plotting the read-mapping density of the cluster and a 2D representation of the hairpin structure. The characteristics of the hairpin as well as precision of the miRNA-duplex were calculated according to different miRNA-annotation criteria. miRNA-candidates were selected if they passed at least two out of three sets of stringent criteria (Supplementary Table S3). Candidates were manually inspected prior to annotation of *bona fide* miRNAs, to ensure the maximum level of confidence in the annotated miRNAs. A blast (48) search was performed with all *bona fide* miRNAs to detect homologous regions containing conservation of both the mir-5p and mir-3p in any Dictyostelia or *Acanthamoeba* with sequenced genomes. Candidate miRNA homologs were manually curated to verify folding of a hairpin structure and expression of small RNAs from these loci, for those species where we sequenced small RNAs. Code and utilized genomes are available on https://doi.org/10.5281/zenodo.7937209.

### Analysis of miRNAs

Analysis of the identified miRNAs was performed in R. Non-coding RNA regions were identified and annotated in the genome using Infernal v1.1.4 (49) with the Rfam covariance model filtered to Amoebozoa (50). Transposable elements were identified and annotated in the genome using tblastx (48) with reference transposable element sequences from Repbase (Genetic Information Research Institute) filtered to the studied organisms (evalue cutoff 10^−15^).

### Whole genome sequencing of *D. firmibasis*

For genomic DNA isolation, 6 × 10^8^*D. firmibasis* cells were harvested from clearing SM Agar/5 plates and washed five times with 50ml PDF buffer [20 mM KCl, 9.2 mM K_2_HPO_4_, 13.2 mM KH_2_PO_4_, 1 mM CaCl_2_, 2.5 mM MgSO_4_, pH 6.4]. Genomic DNA was isolated with QIAGEN 100/g Genomic-tips, according to the ‘Cell cultures’ protocol, but with 0.2 mg/ml RNase A added to the G2 buffer. Short DNA was filtered out with the PacBio Short read eliminator kit. Library preparation was performed with 1.5 μg of filtered genomic DNA using the SQK-LSK112 nanopore sequencing kit. 100 ng of final prepared library was loaded on a MinION Mk1C with R10.4 flowcell and sequencing was performed for 72 hours. Basecalling, adapter trimming and read-splitting was performed with Guppy v6.3.2 (Oxford Nanopore). The data was filtered using Filtlong v0.2.1 (https://github.com/rrwick/Filtlong) to retain the best 5 Gbp of data and remove reads smaller than 1000 bp. Assembly was done using Flye v2.9.1 in nano-hq mode (51), and two rounds of long-read polishing were done using Medaka v1.7.2 (Oxford Nanopore). The filtered reads used for assembly can be accessed from NCBI BioProject PRJNA972620. The assembly used for downstream sRNA mapping and miRNA discovery can be downloaded from https://zenodo.org/doi/10.5281/zenodo.7937209.

### Sequencing of the conserved ddi-miR-1177 region

*Dictyostelium citrinum* and *Dictyostelium intermedium* cells were grown at 22°C on SM Agar/5 (Formedium) with *Klebsiella aerogenes* as food source. DNA was isolated as described previously (52). Primers for amplification of homologs of the ddi-mir-1177 region were designed based on orthologs of the genes up- and downstream of ddi-mir-1177 (DDB_G0287869 and DDB_G0287713 respectively). The intergenic regions homologous to ddi-mir-1177 were amplified by PCR containing 2 μl of lysate, 1U Taq polymerase (Thermo Scientific), 1x reaction buffer, 2 mM MgCl_2_, 0.2 mM dNTPs, 0.2 μM of p1301 and p1330 for *D. citrinum*, and 0.2 μM of p1331 and p1332 for *D. intermedium*, in a final volume of 40 μl (Supplementary Table S1). The PCR was performed as follows: 60 s at 95°C; [15 s at 95°C; 30 s at 46°C or 50°C for *D. citrinum* or *D. intermedium* respectively; 120 s at 62°C] × 35, followed by 7 min at 62°C. The amplified products were gel purified (GeneJET Gel Extraction Kit; Thermo Scientific) and TA-cloned (InsTAclone PCR Cloning Kit; Thermo Scientific). Plasmid DNA from positive clones was isolated (GeneJET Plasmid Miniprep Kit; Thermo Scientific) and Sanger-sequenced (Macrogen). The resulting sequences were aligned to the ddi-mir-1177 and dfi-mir-1177 intergenic region.

### mir-1177 Northern

Dictyostelid strains were grown in association with *K. aerogenes* at 22°C on SM Agar/5 (Formedium). After the majority of bacteria had been consumed, upon onset of development, the plates were harvested into TRIzol Reagent (Invitrogen), and RNA was isolated according to the user guide. 30 μg RNA samples were denatured in 47.5% formamide at 70°C for 5min. RNA was separated on a 12.5% acrylamide gel (7 M urea, 1× TBE) at 12W. In addition to the RNA samples, 0.5 μl of Decade RNA marker (Invitrogen) was loaded on the gel. Transfer, probing and washing was performed as described before (53), with probe p549 specific for miR-1177-5p or probe p1339 for U6 snRNA (Supplementary Table S1). Northern blots were exposed for up to 72 h to BAS-IP MS Phosphorimaging plates (Cytiva) and imaged by Amersham Typhoon (Cytiva).

### Synteny search and alignment of miRNA synteny blocks

The *D. firmibasis* genome assembly was compared to the *D. discoideum* reference genome, and synteny blocks were identified using Satsuma2 v2016-12-07 (54, https://github.com/bioinfologics/satsuma2). Blocks within 5000 bp of each other were merged and the synteny blocks containing miRNAs were visualized in R using the circlize v0.4.15 package (55). To more closely study the evolution of the miRNAs, the synteny blocks with miRNAs were aligned using Clustal Omega v1.2.4 (56).

### Calculation of genome metrics

Genome assembly metrics were calculated using a python script available on https://doi.org/10.5281/zenodo.7937209. BUSCO assessment was performed using BUSCO v5.3.1, with the *eukaryote_odb10* dataset (57). Average nucleotide identity was calculated with FastANI v1.34 (58).

### Phylogeny of RNAi machinery

Orthofinder v2.5.2 (59) was run with the protein fasta files available for the amoebae included in this study, to find orthogroups containing the Argonaute and Dicer proteins. Using the identified homologs, the genomes of *D. firmibasis* and *Acanthamoeba lenticulata* were searched using tblastn with a maximum e-value of 10^−15^ to identify regions of interest containing Argonaute or Dicer homologs. Conserved domains in the homolog candidates were identified using PfamScan (60). Sequences were aligned with MAFFT v7.520 (61), trimmed with trimAl v1.4.rev15 (62), and phylogenetic consensus trees were constructed using IQ-TREE v2.2.2.3 with the Blosum62 amino-acid exchange rate matrix (63).

## Results

### microRNAs are present in six out of six dictyostelid social amoebae studied

**Figure 1.**
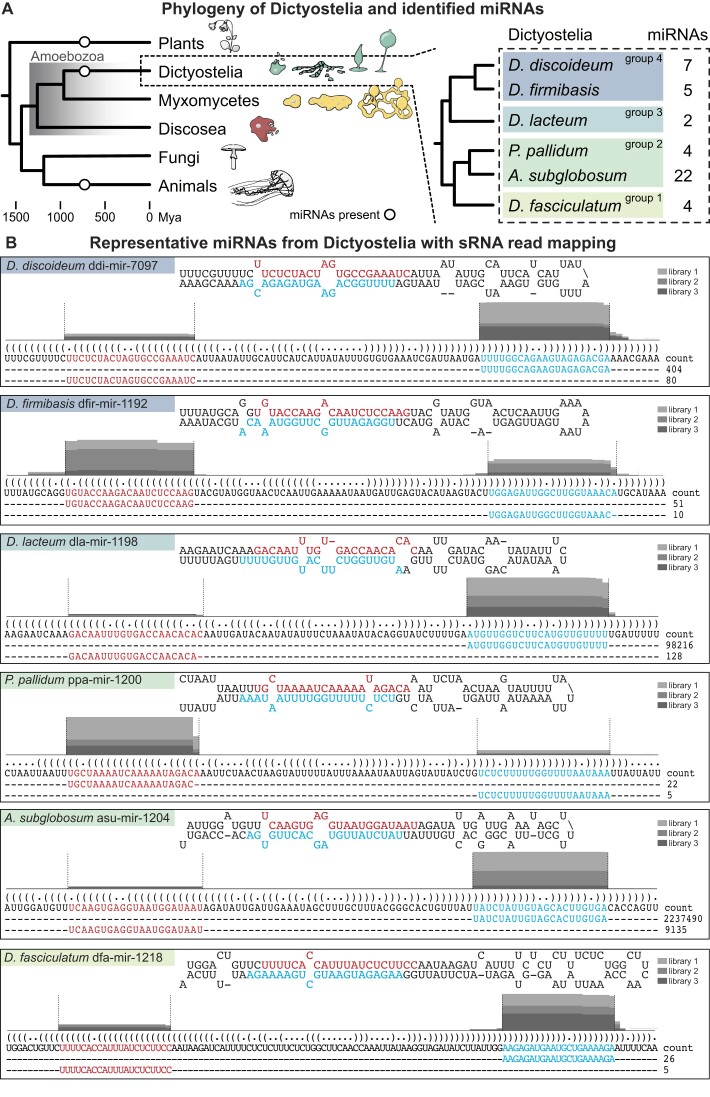
miRNAs identified in social amoebae. (**A**) Simplified phylogeny illustrating the position of Amoebozoa between animals and plants. The branches of Amoebozoa included in this study are shown with characteristic phenotypes: true multicellular aggregation for Dictyostelia, the multinucleate plasmodial stage in Myxomycetes, and single-cellular Discosea. The group of Dictyostelia is further enlarged to display the phylogeny of the four major groups of Dictyostelia social amoebae and the specific members included in this study. Number of miRNAs, identified in this study, for the different dictyostelids is indicated. Mya, million years ago. (**B**) Representative examples of miRNAs from each of the studied Dictyostelia. pri-miRNAs are visualized with their predicted secondary structures, with the miRNA-5p and miRNA-3p marked in red and cyan, respectively. The DNA sequences representing the pri-miRNAs are displayed in bracket notation with their most abundant reads mapping to the miRNA-5p and miRNA-3p arms, together with their read-counts. Histogram above the pri-miRNA DNA sequence shows proportion of sRNA reads mapping to each nucleotide, divided into three shades of grey for each of the three biological replicates. The histograms for dla-mir-1198-5p and asu-mir-1204-5p are not to scale, but shown for clarity. For detailed information, see Supplementary Data.

Our previous discovery of miRNAs in *D. discoideum* prompted us to investigate whether these small RNAs are also present in other social amoebae (30,38). If so, miRNAs may be important not only for cell differentiation in organisms exhibiting clonal multicellularity (e.g. animals and plants) but also for those that display aggregative multicellularity. To address this, we sequenced small RNAs from six species belonging to the four different phylogenetic groups of Dictyostelia (34,64) (Figure 1A). The rationale for investigating the small RNA population from distinct phylogenetic groups was to obtain a comprehensive picture of putative miRNA conservation, i.e. if specific miRNAs are conserved within a specific linage or present throughout Dictyostelia. To facilitate capturing of temporally expressed miRNAs, RNA was isolated from three distinct stages—vegetative growth and two stages of multicellular development (finger/slug stage and culminating fruiting bodies [Supplementary Figure S1]). RNA from the different developmental stages was combined to provide a more manageable number of libraries, allowing us to also sequence large number of small RNAs in biological replicates (three biological replicates for each amoeba), which is important for stringent definition of new miRNAs. The sequenced small RNAs were mapped against the genomes of the respective amoebae and the *E. coli* bacteria used as food source (Supplementary Table S2). Reads derived from bacteria and rRNA were removed from further analyses (Supplementary Figure S2a, Material and Methods). In agreement with the previous results from *D. discoideum* (30), the small RNAs showed a distinct peak at 21 nt for all social amoebae (Supplementary Figure S2b). Our earlier results in *D. discoideum* showed that the majority of these small RNAs were small interfering (si)RNAs (30,38,39,65).

miRNAs have been searched for mostly in animals and plants, using programs designed specifically for each linage (46,66). Hence, commonly only one search program is used for miRNA discovery. However, the biogenesis of *D. discoideum* miRNAs displays similarities to both plant and animal miRNA maturation, congruent with the phylogenetic position of this species (39,67,68). Based on these observations, the sequenced small RNAs were subjected to stringent analyses, applying sets of criteria used to define miRNAs in plants, animals, or both (2,69–71). To ensure that we would report only *bona fide* miRNAs, we exclusively considered candidate RNAs that adhered to at least two of the three sets of strict criteria (Supplementary Figure S3). The results were manually inspected to give an additional level of confidence. With this strategy, high-confidence miRNAs were identified in all analyzed dictyostelids (Supplementary Table S3). It should be noted that for all organisms, we identified miRNAs that adhered to all three criteria (Supplementary Table S3). Examples of miRNAs, predicted miRNA hairpins, and number of reads of the mir-5p and mir-3p for each organism, are illustrated in Figure 1B. Supplementary Data shows the details of the full set of identified miRNAs.

The analyses show that the number of different miRNAs detected in the different Dictyostelia species varies greatly, and that also expression levels seem highly variable (Supplementary Table S4). As in *D. discoideum*, most miRNAs in the other dictyostelids are expressed from a single, usually intergenic, region (Supplementary Table S4). For both asu-mir-1204 and dfa-mir-1220, however, we could detect eight and two paralogs with the exact same miRNA-duplex in the *A. subglobosum* and *Dictyostelium fasciculatum* genomes, respectively (Supplementary Table S4). Both miRNAs are present at relatively high numbers (especially asu-mir-1204), which may reflect that several of the loci are expressed. However, it is still unclear from which loci these particular miRNAs originate. Besides these, no further intra- or interspecies homology of miRNAs was found. It is worth noting that, due to the high level of stringency, not all previously identified miRNAs in *D. discoideum* were detected by the present search approach.

We could not detect a clear correlation between the number of miRNAs and organismal complexity. The largest number of miRNAs were detected in *A. subglobosum*, the only included social amoeba which lacks different cell types. In contrast, the group 4 Dictyostelia, which are the most complex dictyostelids, feature a more modest number of miRNAs. In sum, miRNAs are present in all phylogenetic groups of Dictyostelia, a lineage which originated 600 million years ago.

### miRNAs are present in true unicellular amoebae

**Figure 2.**
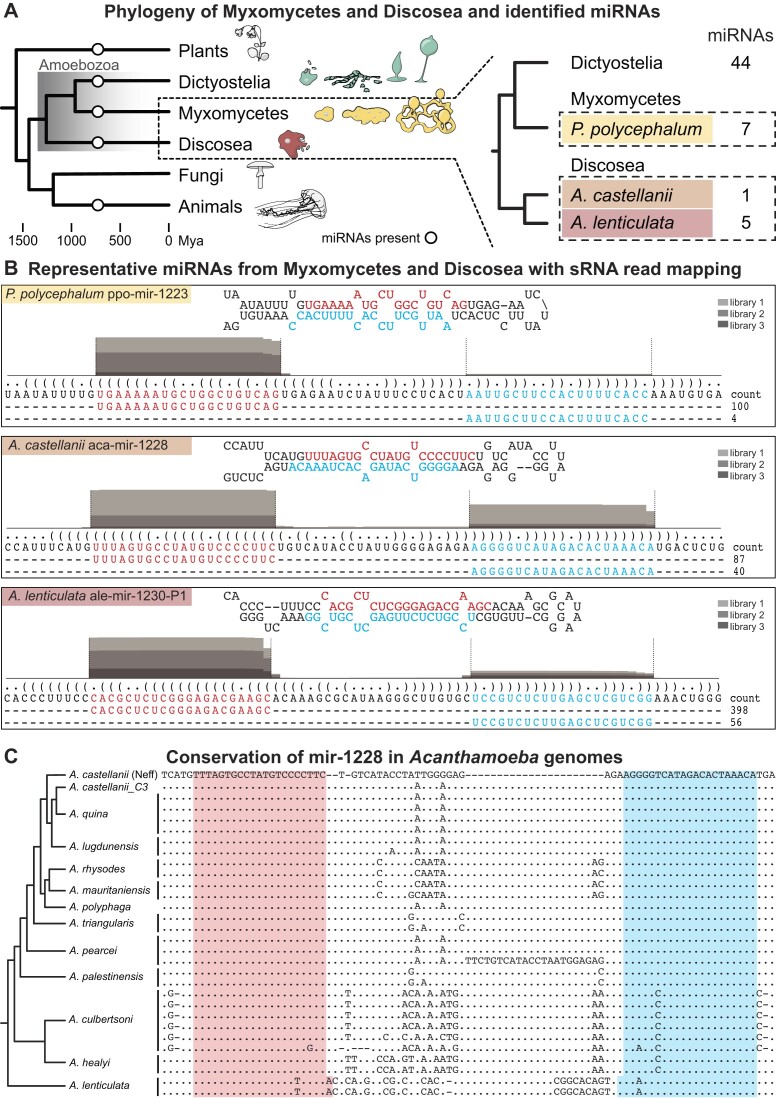
miRNAs identified in *Physarum polycephalum* and *Acanthamoeba* unicellular amoebae. (**A**) Simplified phylogeny of eukaryotes, focusing on the amoebozoan groups Myxomycetes and Discosea. The specific organisms included in this study, with the number of discovered miRNAs in each species, is displayed to the right. (**B**) Examples of miRNAs from *P. polycephalum*, *A. castellanii* and *A. lenticulata*. For further description of panel (B), see figure legend for Figure 1(B). For detailed information, see Supplementary Data. (**C**) Alignment of the predicted mir-1228 hairpin sequence identified in the indicated species. Predicted miRNA-5p and miRNA-3p positions are indicated by pink and cyan background, respectively. The start- and end-positions of the miRNA-5p and miRNA-3p are estimated based on the cleavage observed in *A. castellanii* (Neff strain), except for *A. lenticulata*, which has a 1-nt shifted cleavage. Simplified phylogenetic dendrogram is shown on the left, based on Corsaro (97).

The discovery of miRNAs in all tested dictyostelid social amoebae raised the question whether they are exclusive to aggregative multicellular amoebae or if they were already present in unicellular ancestors. The importance of miRNAs for developmental processes is indicated by their upregulation during *D. discoideum* development (30,38). To address the question if unicellular amoebae have miRNAs, we first sequenced small RNAs from *Physarum polycephalum* (Figure 2A). This Myxomycete is considered to be the closest sequenced unicellular relative to Dictyostelia and features a complex life cycle. It forms a multinucleate plasmodium but is not multicellular and lacks the social interaction specifying Dictyostelia (33,64). Stringent analyses of the sequencing data showed that *P. polycephalum* encodes seven distinct miRNAs (Figure 2B and Supplementary Tables 3 and 4). Importantly, this indicates that the presence of miRNAs is not restricted to amoebae that feature multicellular aggregative development. In contrast to Dictyostelia, most of the small RNAs in *P. polycephalum* are 20 nt long (Supplementary Figure S2, Supplementary Data). The identified *bona fide* miRNAs, however, are mostly 21 nt in length, just like the Dictyostelia miRNAs (Supplementary Figure S4).

The presence of miRNAs in *P. polycephalum* shows that they are not exclusive to Dictyostelia in the group of Amoebozoa. Therefore, we investigated whether miRNAs were also present in true unicellular amoebae that are evolutionarily even more distant to dictyostelids (64). We sequenced small RNAs from *Acanthamoeba castellanii* and *Acanthamoeba lenticulata* and identified one and five miRNAs, respectively (Figure 2, Supplementary Data, Supplementary Table S3). In *A. lenticulata*, two paralogs could be identified for both ale-mir-1228 and ale-mir-1230, and additionally an ortholog of ale-mir-1228 in *A. castellanii*: aca-mir-1228 (Supplementary Table S4). To our knowledge, the miRNAs in *P. polycephalum*, *A. castellanii* and *A. lenticulata* are the first to be reported in Amoebozoa outside of Dictyostelia. Taken together, our data show the presence of miRNAs also in unicellular amoeba, which may indicate roles for miRNAs in both uni- and multicellular life.

### miRNAs are conserved within *Acanthamoeba*

Using stringent search criteria for identification of miRNAs in small RNA sequence libraries is powerful. Adding conservation, when possible, gives an additional level of confidence (72). To further investigate the conservation of the miRNAs found in *A. lenticulata* and *A. castellanii*, we searched the available *Acanthamoeba* genomes (see Material and Methods). Orthologs of the miRNAs, as well as the predicted pre-miRNAs, were identified in 11 of 13 of the available genomes of species that phylogenetically lie between *A. castellanii* and *A. lenticulata* (Figure 2C). However, we could not find miRNA or precursor sequences in more distant *Acanthamoeba* species. As expected, mir-5p and mir-3p sequences are well conserved while the connecting sequences tend to be more variable. Notably, the nucleotide change in mir-3p sequences in *Acanthamoeba healyi* (compared to *A. castellianii*) is placed in a predicted bulge in the miRNA duplex, and the two single nucleotide changes in the mir-5p and mir-3p in *A. lenticulata* are compensatory (Supplementary Figure S5). Hence, these changes should have little impact on the pre-miRNA and miRNA duplex structure. The mir-1228 sequences in *Acanthamoeba cultbertsoni* feature the same nucleotide change as *A. healyi*, however, one of the sequences contains two more nucleotide changes in the miRNA duplex, predicted to cause additional bulges (Supplementary Figure S5). In summary, we provide evidence that numerous *Acanthamoeba* species harbor conserved miRNAs.

### miRNAs are not required for multicellularity in *D. discoideum*

**Figure 3.**
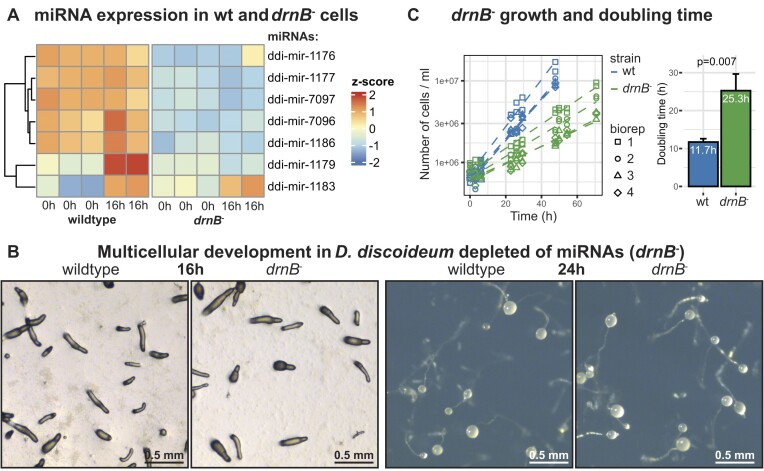
Effect of loss of miRNAs on multicellular development and growth in *D. discoideum*. (**A**) Heatmap showing expression of miRNAs in *D. discoideum*wildtype (wt) and *drnB^−^* strains in vegetative cells (0h) and cells during multicellular development (16 h). (**B)** Images of development for *D. discoideum* cells where *drnB* has been disrupted (*drnB*^−^). Images of development of the wildtype cells shown for comparison. Development was initiated 16 or 24 h prior to imaging. Photos show slugs (16 h) and fruiting bodies (24 h). (**C**) Growth curves of wt and *drnB*^−^ *D. discoideum* cells grown in liquid medium. Four biological replicates for each strain are shown. Doubling times are calculated from the growth curves and summarised in the bargraph. *P*-value from two-tailed t-test comparing the two strains (*n* = 4).

The presence of miRNAs in true unicellular amoebae as well as in social amoebae that can go through aggregative multicellularity, indicate that miRNAs are not required for transition from uni- to multicellular life. We have previously knocked out *drnB*, the gene encoding the Dicer-like protein DrnB in the genetically tractable model organism *D. discoideum*, and reported DrnB′s role in miRNA maturation (30,39). This is supported by the observation that *D. discoideum* miRNAs, presented in this study, are downregulated in the *drnB^−^* knockout strain (Figure 3A). In plants and animals, loss of miRNAs is paired with serious developmental defects (73,74), but in contrast, the *D. discoideum* multicellular development was unaffected in the *drnB^−^* knockout strain (Figure 3B). Instead, the *drnB^−^* cells have a slower growth rate in axenic media, suggesting that miRNAs have a regulatory role in the unicellular life stage of the amoeba (Figure 3C). Hence, miRNAs do not appear to be connected to the transition to multicellular life in Amoebozoa and have no crucial impact on multicellular development of *D. discoideum*.

### miRNA conserved within group 4 Dictyostelia

**Figure 4.**
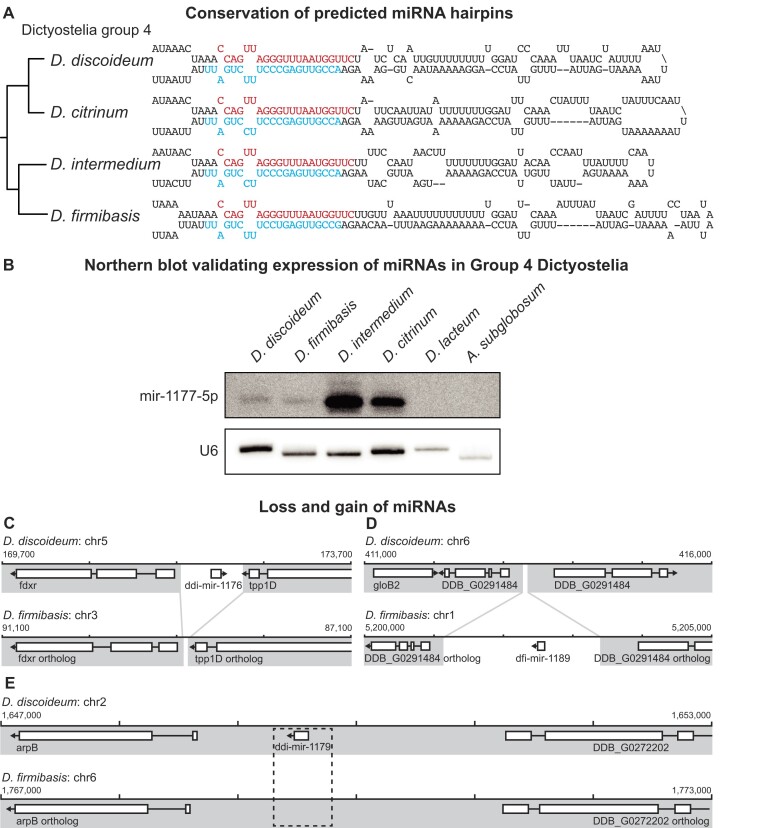
Conservation of mir-1177 in group 4 species of Dictyostelia. (**A**) Predicted secondary structure of the mir-1177 orthologs found in *D. discoideum*, *D. citrinum*, *D. intermedium* and *D. firmibasis*, with a simplified phylogeny shown on the left, based on Schilde *et al.* (64). The DNA sequences from *D. citrinum* and *D. intermedium* were resolved by sequencing the genomic region in which the ortholog is located. The *D. firmibasis* ortholog was resolved by whole genome sequencing. Positions of the mir-1177-5p and mir-1177-3p are indicated in red and cyan, respectively. (**B**) Expression of mir-1177-5p in the group 4 dictyostelids *D. discoideum*, *D. firmibasis*, *D. intermedium* and *D. citrinum* verified by northern blot. *D. lacteum* or *A. subglobosum*, outside group 4, showed no expression signal. Loading control by probing for U6 spliceosomal RNA. Both panels show different sections of the same membrane at different exposure times. (**C–E)** Detailed examples of syntenic regions containing miRNAs, suggesting different evolutionary histories. In some instances, the intergenic regions containing the miRNA are lacking in the other species, such as in (C) and (D). In other synteny blocks, the size of the intergenic region is preserved, but extensive conservation of the miRNA sequence, or expression of sRNAs from this region, is not supported (as in E).

The discovery of high-confidence miRNAs in all investigated Dictyostelia suggests that they are important regulatory small ncRNAs in these social amoebae. Even though miRNAs do not appear to have a major impact on overall *D. discoideum* development (Figure 3), they could still have been involved in the evolution of the social behavior in dictyostelid amoebae. If so, we may expect some miRNAs to be conserved throughout Dictyostelia. Surprisingly, we initially failed to find evidence for miRNA gene conservation between any of the species. However, a closer inspection of the small RNA sequences from *D. firmibasis* revealed a homolog of the *D. discoideum* miRNA ddi-mir-1177-5p. We reasoned that our initial failure to identify this homology was due to the quality of the *D. firmibasis* genome assembly (Supplementary Table S5) that prevented mapping of the ddi-mir-1177-5p homolog to the genome. Therefore, we carried out *de novo* nanopore long-read sequencing of the genome to obtain high coverage also of intergenic regions, which is challenging due to high AT-content (86.2% in intergenic regions for *D. discoideum*) (see Materials and methods). This reduced the number of contigs from 997 to 8 and also has BUSCO scores similar to that of *D. discoideum*, with 92.9% and 90.2% single copy orthologs in *D. firmibasis* and *D. discoideum*, respectively (Supplementary Table S5). By calculating the Average Nucleotide Identity (ANI) between *D. discoideum* and *D. firmibasis* (86.5%) (Materials and methods), we could confirm that these species are evolutionarily relatively close to each other, in particular when compared to other organisms in this study (Supplementary Table S5). Upon re-mapping of the small RNAs to the new improved *D. firmibasis* genome sequence, the identified miRNAs increased from five to nine (Supplementary Tables 3 and 4; Supplementary Data). As we had hypothesized, one newly mapped miRNA, dfi-mir-1177, was an ortholog of the *D. discoideum* miRNA ddi-mir-1177. Both of these miRNA genes lie in the intergenic region between (orthologs of) *D. discoideum* genes DDB_G0287869 and DDB_G0287713, an unresolved region in the previous version of the *D. firmibasis* genome. We next asked whether sequences for this miRNA were present in other group 4 dicyostelids, i.e. *D. citrinum* and *D. intermedium*. We searched their genomes for orthologs to DDB_G0287869 and DDB_G0287713 and PCR-amplified and sequenced the unresolved intergenic regions. In both cases, homologous sequences to ddi-mir-1177 and the miRNA hairpin were identified (Figure 4A, Supplementary Figure S6a), meaning that there is synteny in the mir-1177 region for all four genomes. Further analyses showed that while the mir-1177-5p is perfectly conserved between the analyzed group 4 dictyostelids, the mir-1177-3p has one or two nucleotide changes as compared to ddi-mir-1177-3p in *D. discoideum* (Supplementary Figure S6a). In analogy to the conserved *Acanthamoeba* miRNA, these changes are located in bulges or predicted to maintain base-pairing. Hence, they should not affect the structure of the miRNA-duplex. The remainder of the sequences, between mir-5p and mir-3p, are not as well conserved but still predicted to form pre-miRNA stem-loops (Figure 4A). Expression of the miRNA from all four species was verified by northern blot. By contrast, in line with RNA-seq results and genome analyses, no hybridization signal was detected from the more distantly related *Dictyostelium lacteum* and *A. subglobosum* (Figure 4B). These results show that at least one miRNA appears to be conserved exclusively within group 4 dictyostelids.

### Evolution of miRNAs in *D. firmibasis* and *D. discoideum*

Considering the relatively close evolutionary positions of *D. discoideum* and *D. firmibasis*, we were surprised that, except for ddi-mir-1177, none of the other miRNAs were conserved between these dictyostelids. To approach the evolution of miRNAs in these species, the two genomes were compared to identify synteny blocks. Whilst synteny between *D. discoideum* and distant social amoebae was previously reported to be low (34), we found that 84% of the *D. discoideum* genome was syntenic to *D. firmibasis*. The synteny regions that contained miRNA sequences in either species were aligned for analyses (Supplementary Figure S6b). Interestingly, for some *D. discoideum* miRNAs, the orthologous intergenic region in *D. firmibasis* is reduced in size indicating the absence of the whole miRNA locus (Figure 4C). The same was observed for some *D. firmibasis* miRNAs, such as dfi-mir-1192 (Figure 4D). Here, the orthologous intergenic region in *D. discoideum* is ∼2 kb shorter, completely lacking an orthologous gene for the miRNA. In other cases, the orthologous intergenic regions are of similar length, and the miRNA seems to have evolved through multiple nucleotide substitutions, as opposed to insertion/deletion of the entire locus. This is illustrated by the *D. discoideum* specific miRNA ddi-mir-1179. Here, the orthologous intergenic region in *D. firmibasis* also has the potential to be transcribed into an imperfectly paired hairpin. This might suggest how this miRNA has emerged in *D. discoideum*, or alternatively how it has been degenerated in *D. firmibasis* (Figure 4E, Supplementary Figure S6c). Taken together, by comparing the evolutionarily relatively closely related genomes of *D. discoideum* and *D. firmibasis*, we gain insights into the evolutionary history of miRNAs, which seems to depend on gains and losses of miRNA ‘modules’ as well as modification of stem-loop structures.

### Features of amoebozoan miRNAs

**Figure 5.**
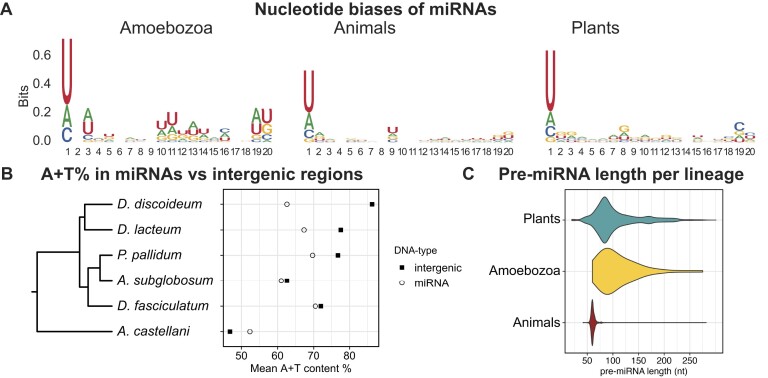
Characteristics of amoebozoan miRNAs. (**A**) Sequence logo of the amoebozoan miRNAs identified in this study, as well as animal miRNAs accessed from MirGeneDB (75), and plant miRNAs from PmiREN (76). miRNAs from all three major phylogenetic groups show a 5′U preference. (**B**) AT-content of intergenic regions and of miRNA for the analyzed species for which genome annotations are available. (**C**) Violin plots of the pre-miRNA lengths in plants, Amoebozoa and animals. Pre-miRNA lengths for each of the amoebozoan species shown in Supplementary Figure S7b.

The identified miRNAs in the different amoebae allowed for analyses of shared features. One general trend is the identity of the 5′ end, most frequently a uridine or, to a lesser extent, an adenosine or cytidine, as in plant and animal miRNAs (Figure 5A). Additionally, an apparent enrichment of nucleotides in the middle of the amoebozoan miRNAs could be observed, but we believe this is mainly due to the low number of sequences (61 miRNAs) relative to plants and animals (respectively 20 338 and 17 599 included in this study; (75,76)). The majority of miRNAs in the amoebae are derived from intergenic regions (Supplementary Figure S7a, Supplementary Table S4) which are AT-rich in social amoebae. However, the miRNAs are considerably less AT-rich, a pattern seen in other ncRNAs in the AT-rich genomes of dictyostelids (77,78) (Figure 5B). Although the majority of miRNAs originate from intergenic regions, miRNAs in *Polysphondylium pallidum* are mostly intronic, and in both *A. subglobosum* and *D. fasciculatum*, some miRNA sequences are in antisense orientation to annotated coding regions (Supplementary Figure S7a, Supplementary Table S4). The miRNAs are derived from predicted pre-miRNAs, varying in size within species but most often 75–125 nt in length, similar to the size of pre-miRNAs in plants. Two miRNAs, dfi-mir-1190 and ppo-mir-1226 are derived from unusually long hairpin precursors, more than 200 nt, and may reflect newly evolved miRNAs (see Discussion) (Figure 5C, Supplementary Figure S7b, Supplementary Table S4, Supplementary Data). As seen previously, not all small RNAs in amoebae are miRNAs. In *A. subglobosum*, the majority of the 21 nt RNAs are miRNAs, but in the other species, RNAs originating from e.g. transposons or unannotated intergenic regions dominate (Supplementary Figure S7c).

### The RNAi machinery in Amoebozoa

**Figure 6.**
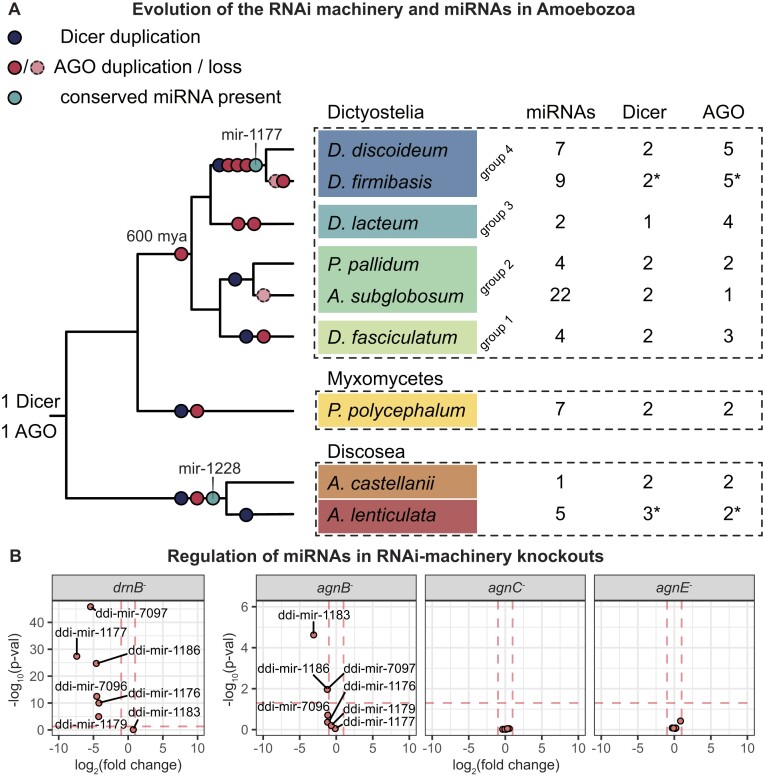
Model of the evolution of the miRNAs, Dicers, and Argonautes in Amoebozoa. (**A**) Number of miRNAs, Dicers and Argonautes (AGO) identified in each of the species. Asterisk (*) indicates that genome annotations or protein sequences were not available and these orthologs were identified manually. Schematic phylogeny of the Amoebozoa with root of the Dictyostelia at 600 mya according to Heidel *et al.* (34). Duplications/losses of the Dicer and Argonaute machinery are indicated on the dendrogram based on parsimonious interpretation of Supplementary Figure S8 and Supplementary Figure S9. For miRNAs where we could detect conservation between species, i.e. mir-1177 and mir-1229, their presence prior to speciation is indicated. (**B**) Volcano plots showing the regulation of miRNAs relative to wt in different RNAi machinery knockouts. log_2_(fold change) indicates the extent to which the miRNAs are up- or down-regulated, with vertical pink dashed lines marking a fold change of 0.5 and 2, respectively. –log_10_(*P*-val) shows the significance, with higher values for more significantly regulated miRNAs. The horizonal dashed pink line marks a *P*-value of 0.05.

Dicers and Argonautes are the most conserved components for processing and function of miRNAs (9,79). We asked how the evolution of the RNAi machinery matched the evolution of miRNAs in the studied amoebae. In most species, we could identify two Dicers and two or more Argonautes (Figure 6A, Supplementary Figures 8 and 9). Looking more closely at the evolution of Dicer proteins, a single copy of Dicer was likely present prior to the expansion of the dictyostelids, which then duplicated in Groups 1, 2 and 4, but not 3, leaving *D. lacteum* with just a single Dicer (Supplementary Figure S9; see also (68) for additional discussion). The *Acanthamoeba* appear to have experienced a duplication of Dicer prior to the *A. lenticulata–A. castellanii* split. Concerning the Argonautes, a single copy was most likely present in the last common ancestor of the Amoebozoa. A duplication event prior to the expansion of the dictyostelids resulted in two or more copies of the Argonautes in most of these species (Supplementary Figure S9). A duplication of the Argonautes is also suggested in *Acanthamoeba*, leaving both *A. lenticulata* and *A. castellanii* with two Argonaute paralogs. The expansion of the RNAi machinery in *Acanthamoeba* points towards the appearance of a specialized miRNA processing machinery, which would be in line with the discovery of conserved miRNAs in *Acanthamoeba*. The gains and losses of Dicer and Argonaute proteins, or number of miRNAs, do not seem to be correlated with genome size or genome reductions (Supplementary Table S5).

The duplications of Dicer and Argonaute proteins indicate that some of these might have evolved to support the biogenesis and function of miRNAs in these species. This is exemplified by *D. discoideum* where the two Dicer-like proteins, DrnA and DrnB, seem to have different functions, with DrnA likely involved in generating siRNAs that target transposons and DrnB dedicated to miRNA maturation (30,39,40,68). In order to study the Argonautes, we knocked out genes encoding AgnB, AgnC and AgnE in *D. discoideum*, and sequenced small RNAs. Disrupting *agnB*, showed a decrease in the number of most miRNAs, albeit generally to a lesser extent than in the *drnB^−^* knockout (Figure 6B). This decrease in miRNAs is expected for an Argonaute that directly binds and protects miRNAs (80–82). Knocking out *agnC* or *agnE* did not affect the level of miRNAs significantly (Figure 6B). In the *drnB^−^* knockout strain, ddi-mir-1183 numbers are not visibly affected, but processing precision is significantly reduced (39; Supplementary Figure S10). In the *agnB^−^* strain, the ddi-mir-1183 precision is similar to wildtype, but only a fraction of the miRNA reads remains, showing that its stability is impacted but the processing is unaffected (Supplementary Figure S10). Interestingly, *agnB* and *agnE* in *D. discoideum* are derived from the same ancestral Argonaute gene (Supplementary Figure S9), but the miRNA repertoire is only affected upon loss of *agnB*, highlighting how these paralogs might have evolved specialized functions in the cell.

Interestingly, while the RNA directed RNA polymerases (RdRPs) seem to have expanded in the Dictyostelia and *P. polycephalum*, we could not identify any genes for RdRPs in *A. castellanii*and*A. lenticulata* (Supplementary Figure S11). In *D. discoideum*, one of the RdRP’s, RrpC, is involved in generating small RNAs that appear to protect the genome from amplification of transposons (65,83,84). In fact, the great majority of small RNAs in *D. discoideum* are derived from the retrotransposon DIRS-1, which makes up about 3.3% of the genome (83,85). Also, in most of the other amoebae, except for the true unicellular amoebae, we see small RNAs matching transposons (Supplementary Figure S7c). Hence, the presence of RdRPs appears to be connected to small RNAs from transposons, and may play a role in inhibiting transposition in these organisms. Taken together, the RNAi machinery is present in all studied amoebozoan species and has been expanded by duplications. The flexibility of this machinery is demonstrated by the variable numbers of RNAi-genes in different organisms, underscored by the observation that morphological complexity coincides with an expanded repertoire of RNAi-related genes.

## Discussion

The great majority of miRNAs have been discovered in plants and animals, where they have been estimated to regulate nearly all biological processes. In particular, it was also suggested that miRNAs played a role in the evolution of multicellularity in these lineages (17,24,86). Hence, most of what we know about biogenesis, function, and evolution of miRNAs stems from studies in plants and animals. Although, to understand the commonality of miRNA regulation and their putative role in evolution of multicellularity, it is essential to investigate how widespread miRNAs are, i.e. in what other organisms they are present. In order to address this, we searched for miRNAs and their associated genes in Amoebozoa, a phylogenetic group positioned between plants and animals, in which a wide variety of both uni- and multicellular lifestyles can be found (31,67). Until now, high confidence miRNAs had only been described in the social amoeba *D. discoideum* (30,38–40). Here, we substantially expanded the number of amoebae carrying miRNAs by sequencing and stringently analyzing the small RNA repertoire of eight amoebozoan species in addition to *D. discoideum*. We hypothesized that miRNAs would be predominantly present in the multicellular social amoebae, supporting the evolution of their multicellular lifestyle. To study this, we included species belonging to Amoebozoa with a range of lifestyles, including several social amoebae from the monophyletic group Dictyostelia, which all feature aggregative multicellularity, as well as strictly unicellular species. Surprisingly, we identified miRNAs in all sequenced amoebae, including those lacking any sort of multicellular lifestyle, revealing that miRNAs are more widespread than previously believed. Furthermore, the fact that depletion of the miRNAs did not affect aggregation and multicellularity, and that miRNAs are present in true unicellular amoebae show that miRNAs are not required *per se* for transition from uni- to multicellular life (Figure 2, 3).

### Did miRNAs play a role in the evolution of multicellularity?

Our discovery of miRNAs in unicellular outgroups to Dictyostelia is analogous to the findings of miRNAs in unicellular organisms of both the plant and animal lineages (19,20,22). This suggests that the evolution of clonal multicellularity in plants and animals, as well as the evolution of aggregative multicellularity in Dictyostelia, did not coincide with the evolution of miRNAs and the miRNA machinery (Figure 6) (19,20,22). However, the importance of miRNAs in multicellular processes and development in animals is well documented. Many miRNAs are conserved in bilaterian animals, and mir-100/mir10, is found in almost all multicellular animals (70,87). Also in plants, some miRNAs are highly conserved (88). Dysregulation of these ancient conserved miRNAs often causes developmental phenotypes, illustrating the significance of these miRNAs for proper multicellular development (89–91). In contrast to the situation in animals and plants, we could not detect any conserved miRNAs throughout Dictyostelia. In addition, different studies have shown a correlation between the number of miRNAs and increasing complexity, though in plants this correlation is less clear. We do not see an obvious correlation between the number of miRNAs and complexity in Amoebozoa (Figure 6). Also, in a mutant *D. discoideum* strain depleted of miRNAs, no effect on multicellular development was observed (Figure 3). In animals, most miRNAs only partially regulate their targets and rather act as buffers to, for instance, stabilize phenotypes and dampen variability (88,92,93). Perhaps, this is also true for miRNA regulation in Dictyostelia, and more detailed studies of phenotypic effects are required to detect the influence of miRNAs. It would also be useful to investigate how other Dictyostelia respond to depletion of miRNAs.

### When did miRNAs evolve?

It is commonly believed that miRNAs evolved independently in plants and animals, following the expansion of their RNAi machinery (16,19). Partly, our data agree with this since we observe a low level of conservation of miRNAs indicating that miRNAs evolved independently and several times in Amoebozoa. Also, we see expansions of the miRNA machinery, i.e. genes for Dicers and Argonautes, simultaneously with the appearance of novel miRNAs (Figure 6). In the model *D. discoideum*, the expansion of the RNAi machinery is connected to division of labour. The two Dicer-like proteins, DrnA and DrnB (Figure 6A), appear to be involved in siRNA production and to produce mature miRNAs, respectively (30,39,68). Furthermore, five genes for Argonautes are present in *D. discoideum* (Figure 6A), where AgnA, AgnC and AgnE have been demonstrated to affect retrotransposons and knocking out AgnA increases the level of miRNAs, for unknown reasons (40,94,95). AgnD appears to be a pseudogene (67,96), while AgnB likely binds miRNAs (Figure 6B).

Given these findings, we may argue for the common model that RNAi was present in the last eukaryotic common ancestor where it protected against genomic invaders such as transposons and viruses (9). Hence, there has been, and is, a strong evolutionary pressure to maintain the RNAi machinery, which could act as a scaffold from which miRNAs can readily evolve in different organisms (14–16,25). This is further supported by our data showing that a minimal basic miRNA machinery (considering Argonautes and Dicers) can be sufficient, where some amoebozoan species feature only a single Dicer or a single Argonaute. Thus, the convergent evolutionary scenario is possible but the hypothesis of an ancient miRNA machinery cannot be ruled out and is discussed further below.

### How fast do miRNAs evolve?

While we found conservation of some miRNAs between unicellular members of *Acanthamoeba*, and also between the group 4 dictyostelids *D. discoideum* and *D. firmibasis*, the other identified miRNAs were all unique for each species. This indicates a rapid evolution of amoebozoan miRNAs. The presence of young miRNAs is corroborated by miRNAs dfi-mir-1190 and ppo-mir-1226 (Supplementary Data). These miRNAs are derived from unusually long hairpin structures, with no or only one mismatch in the miRNA duplex. These features have been suggested to signify newly evolved miRNAs (98). To analyze in more detail how some of these miRNAs evolved, we investigated the two most closely related organisms in our dataset, *D. discoideum* and *D. firmibasis*. While some of the miRNAs seem to have been acquired or lost gradually, for others we could identify insertions or deletions of whole miRNA modules (Figure 4). The fact that these two closely related organisms have such a diversified miRNA repertoire shows that broadly, their individual miRNAs are under weak evolutionary pressure, consistent with the minor phenotypic effects we observed upon depletion of miRNAs in *D. discoideum*. The rapid gain and loss of miRNAs between the two closely related species and the presence of miRNAs in many eukaryotic lineages could support that miRNAs were present in an ancient eukaryotic ancestor and that the fast turnover of miRNA may obscure their origin. We have recently initiated a study where we will acquire high-quality genomes of closely related amoebae and sequence their small RNAs to obtain a high-resolution picture of mechanisms driving miRNA evolution in Dictyostelia.

Taken together, this work greatly expands the number of miRNAs in Amoebozoa, and the number of amoebozoan species, or protists in general, that feature miRNAs. The presence of miRNAs throughout Amoebozoa indicates that miRNAs are much more common outside animals and plants than previously believed, and argues against the idea that miRNAs drive the evolution of multicellularity.

## Supplementary Material

gkae109_supplemental_files

## Data Availability

All sequencing data generated in this study can be accessed at NCBI BioProject PRJNA972620. sRNA sequencing data of different amoebozoan species for miRNA discovery are available with BioSample accessions SAMN35084086 - SAMN35084094. sRNA sequencing data to compare the *D. discoideum drnB*^−^ knockout strain and Argonaute knockout strains with wildtype strain are available with BioSample accessions SAMN35084095 - SAMN35084098 & SAMN39466833 - SAMN39466838. Third-generation sequencing data of *D. firmibasis*, used to assemble the genome, is available with BioSample accession SAMN35084099. The *D. firmibasis* genome assembly used in this study and data used for analysis of miRNAs, synteny between *D, firmibasis* and *D. discoideum*, and differential miRNA expression is available at https://doi.org/10.5281/zenodo.7937209 (data_in). Data generated such as miRNA fasta files and tables with miRNA characteristics, are available at https://doi.org/10.5281/zenodo.7937209 (data_out). For initial curation of the miRNA candidates, we used a python script available at https://doi.org/10.5281/zenodo.7937209 (miRNA_curation.py). A folder with scripts and sample data to perform an example run is provided in the repository. This repository also contains code for downstream analysis of the miRNAs, genome synteny analysis, and differential miRNA expression analysis, which was performed in R. Also included in the repository are the plots generated in the analysis, and helper scripts used for generating input data.

## Notes

Present address: Zhen Liao, Génétique Moléculaire, Génomique, Microbiologie (GMGM), University of Strasbourg, 67000 Strasbourg, France.
