# Supplementary material for: Evolution of microRNAs in Amoebozoa and implications for the origin of multicellularity
Source: Nucleic Acids Res. 2024 Feb 20;52(6):3121–36. doi: 10.1093/nar/gkae109 (PMC11014262; doi:10.1093/nar/gkae109)

Supplementary Data | Detailed depiction of identified miRNAs. Predicted miRNA hairpin sequences and structure displayed in bracket notion. Identified miRNA-5p is indicated in red, and the miRNA-3p in cyan. Lowercase nucleotides represent the 5' and 3' ends of the miRNA-5p and miRNA-3p. Below the sequence, all mapped small RNA reads are aligned to the miRNA hairpin and the number reads and their length are shown to the right. Results are shown for all small RNA sequencing libraries combined (denoted Libraries combined). For miRNAs that have fewer than 50,000 reads mapped, the results are shown for the individual libraries as well (denoted Library = 1 to 3). The percentage of reads that map to the exact 5' nucleotide of either the miRNA-5p or miRNA-3p is shown as 'precision' and calculated on the entire miRNA-hairpin (denoted total), and on the 5' and 3' arms of the miRNA hairpin (denoted 5p-arm and 3p-arm respectively). The folded miRNA hairpin structure, shown below the mapped small RNA, was predicted using the ViennaRNA RNAlib-2.6.2 python package, with 22°C folding temperature. At the bottom, a graph shows the read mapping density on the miRNA hairpin, with the miRNA-5p and miRNA-3p indicated in red and cyan respectively. The different shades of grey in the graph represent the different small RNA sequencing libraries. From some miRNA hairpins, two miRNA duplexes might be processed in tandem, in which case the two miRNA-5p sequences are colored red and yellow, and the two miRNA-3p sequences are colored dark blue and cyan. ddi-mir-1186 was named ddi_mir_can_D1 when it was first identified (Meier et al., 2016), but we have here renamed it to ddi-mir-1186.

Identified miRNAs are presented separately for each species:

D. discoideum.. p.2-9

D. firmibasis (genome GCA_000277485.1).............p.10-18

D. lacteum..p.19-21

P. pallidum... p.22-28

A. subglobosum..p.29-46

D. fasciculatum.. p.47-51

P. polycephalum... p.52-60

A. castellanii.. p.61

A. lenticulata..p.62-68

D. firmibasis (de-novo assembly).............................p. 69-85

D. discoideum ddi-mir-1176

```
((((((((((.((((((((.((((((((.((((.(((((((((.(.(.......).).))))))))).)))).)))))))).)))))))).))))))))))  -70.6 kcal/mol
UAUCAGGUGGcCAAUUUUUAUCAAGGAAAGcUGUAUCAUCAAGUGCCUCCCUCUGUCUCUUGAUGAUUCAGCcUUCCUUGACAAAAAUUGCCcACCUGAUA  count    length
Libraries combined          Precision: [ total = 98.5% | 5p-arm = 98.4% | 3p-arm = 100.0% ]
----------CCAAUUUUUAUCAAGGAAAGC----------------------------------------------------------------------  694      21
------------------------------------------------------------------------CUUCCUUGACAAAAAUUGCCC--------  29       21
------------------------------------------------------------------------CUUCCUUGACAAAAAUUGCC---------  15       20
----------CCAAUUUUUAUCAAGGAAAG-----------------------------------------------------------------------  24       20
---------GCCAAUUUUUAUCAAGGAAAG-----------------------------------------------------------------------  3        21
-----------CAAUUUUUAUCAAGGAAAGC----------------------------------------------------------------------  5        20
-------UGGCCAAUUUUUAUCAAGGAAAG-----------------------------------------------------------------------  3        23
---------GCCAAUUUUUAUCAAGGAAAGC----------------------------------------------------------------------  1        22
----------CCAAUUUUUAUCAAGGAAAGCU---------------------------------------------------------------------  1        22
Library = 1        Precision: [ total = 99.0% | 5p-arm = 98.9% | 3p-arm = 100.0% ]
----------CCAAUUUUUAUCAAGGAAAGC----------------------------------------------------------------------  171      21
------------------------------------------------------------------------CUUCCUUGACAAAAAUUGCCC--------  15       21
------------------------------------------------------------------------CUUCCUUGACAAAAAUUGCC---------  6        20
----------CCAAUUUUUAUCAAGGAAAG-----------------------------------------------------------------------  5        20
---------GCCAAUUUUUAUCAAGGAAAG-----------------------------------------------------------------------  1        21
-----------CAAUUUUUAUCAAGGAAAGC----------------------------------------------------------------------  1        20
Library = 2        Precision: [ total = 98.8% | 5p-arm = 98.7% | 3p-arm = 100.0% ]
----------CCAAUUUUUAUCAAGGAAAGC----------------------------------------------------------------------  71       21
----------CCAAUUUUUAUCAAGGAAAG-----------------------------------------------------------------------  4        20
------------------------------------------------------------------------CUUCCUUGACAAAAAUUGCCC--------  3        21
------------------------------------------------------------------------CUUCCUUGACAAAAAUUGCC---------  2        20
---------GCCAAUUUUUAUCAAGGAAAG-----------------------------------------------------------------------  1        21
Library = 3        Precision: [ total = 98.2% | 5p-arm = 98.1% | 3p-arm = 100.0% ]
----------CCAAUUUUUAUCAAGGAAAGC----------------------------------------------------------------------  452      21
----------CCAAUUUUUAUCAAGGAAAG-----------------------------------------------------------------------  15       20
------------------------------------------------------------------------CUUCCUUGACAAAAAUUGCCC--------  11       21
------------------------------------------------------------------------CUUCCUUGACAAAAAUUGCC---------  7        20
-----------CAAUUUUUAUCAAGGAAAGC----------------------------------------------------------------------  4        20
-------UGGCCAAUUUUUAUCAAGGAAAG-----------------------------------------------------------------------  3        23
---------GCCAAUUUUUAUCAAGGAAAG-----------------------------------------------------------------------  1        21
---------GCCAAUUUUUAUCAAGGAAAGC----------------------------------------------------------------------  1        22
----------CCAAUUUUUAUCAAGGAAAGCU---------------------------------------------------------------------  1        22
```

```
          c        A        A    U         U C UCC
UAUCAGGUGG CAAUUUUU UCAAGGAA GcUG AUCAUCAAG G C   \
AUAGUCCAcC GUUAAAAA AGUUCCUU CGAC UAGUAGUUC C G   C
          C        C        c    U         U U UCU
```

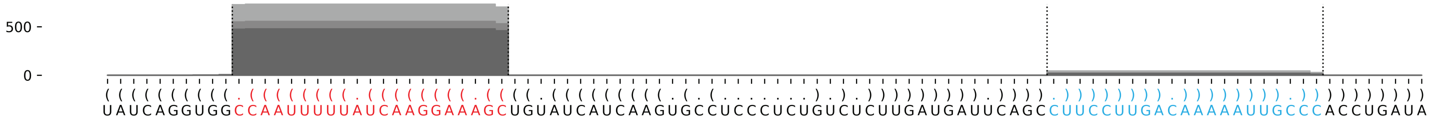

D. discoideum ddi-mir-1177

```
......((((.(((..((((((((((((((((.((.((.(((((((((((.((((..((((..(((((.(((((.......)))))))))))))))..)))))))))))))))).))))..)))))))))))))))..))).))))......   -65.48 kcal/mol
AUAAACUAAAcCAGUUAGGGUUUAAUGGUUcUAUCUCAAUUGUUUUUUUUUUGGAUCCCAAAUUUAAUCUAUUUUAAUUUAAAAAAUGAUUAUUUGUUAUCCAGGAAAAAUAACUGGAAAAGAaCCGUUGAGCCCUUUCUGAUuUAUUAAUU   count    length
Libraries combined          Precision: [ total = 98.7% | 5p-arm = 98.6% | 3p-arm = 99.2% ]
----------CCAGUUAGGGUUUAAUGGUUC-------------------------------------------------------------------------------------------------------------------------   612      21
--------------------------------------------------------------------------------------------------------------------------ACCGUUGAGCCCUUUCUG------------   28       18
----------CCAGUUAGGGUUUAAUGGUUCU------------------------------------------------------------------------------------------------------------------------   30       22
--------------------------------------------------------------------------------------------------------------------------ACCGUUGAGCCCUUUCUGAU----------   70       20
----------CCAGUUAGGGUUUAAUGGUU--------------------------------------------------------------------------------------------------------------------------   5        20
-----------CAGUUAGGGUUUAAUGGUUC-------------------------------------------------------------------------------------------------------------------------   8        20
--------------------------------------------------------------------------------------------------------------------------ACCGUUGAGCCCUUUCUGAUU---------   23       21
----------CCAGUUAGGGUUUAAUGGU---------------------------------------------------------------------------------------------------------------------------   1        19
----------CCAGUUAGGGUUUAAUGGUUCUA-----------------------------------------------------------------------------------------------------------------------   1        23
------------AGUUAGGGUUUAAUGGUUCU------------------------------------------------------------------------------------------------------------------------   1        20
-----------------------------------------------------------------------------------------------------AGGAAAAAUAACUGGAAAAGA------------------------------   1        21
--------------------------------------------------------------------------------------------------------------------------ACCGUUGAGCCCUUUCUGA-----------   6        19
--------------------------------------------------------------------------------------------------------------------------ACCGUUGAGCCCUUUCUGAUUU--------   1        22
Library = 1        Precision: [ total = 98.8% | 5p-arm = 98.8% | 3p-arm = 100.0% ]
----------CCAGUUAGGGUUUAAUGGUUC-------------------------------------------------------------------------------------------------------------------------   152      21
--------------------------------------------------------------------------------------------------------------------------ACCGUUGAGCCCUUUCUG------------   6        18
----------CCAGUUAGGGUUUAAUGGUUCU------------------------------------------------------------------------------------------------------------------------   4        22
--------------------------------------------------------------------------------------------------------------------------ACCGUUGAGCCCUUUCUGAU----------   4        20
----------CCAGUUAGGGUUUAAUGGUU--------------------------------------------------------------------------------------------------------------------------   2        20
-----------CAGUUAGGGUUUAAUGGUUC-------------------------------------------------------------------------------------------------------------------------   2        20
--------------------------------------------------------------------------------------------------------------------------ACCGUUGAGCCCUUUCUGAUU---------   2        21
Library = 2        Precision: [ total = 97.7% | 5p-arm = 97.5% | 3p-arm = 98.2% ]
----------CCAGUUAGGGUUUAAUGGUUC-------------------------------------------------------------------------------------------------------------------------   150      21
--------------------------------------------------------------------------------------------------------------------------ACCGUUGAGCCCUUUCUGAU----------   30       20
--------------------------------------------------------------------------------------------------------------------------ACCGUUGAGCCCUUUCUGAUU---------   12       21
--------------------------------------------------------------------------------------------------------------------------ACCGUUGAGCCCUUUCUG------------   9        18
----------CCAGUUAGGGUUUAAUGGUUCU------------------------------------------------------------------------------------------------------------------------   4        22
--------------------------------------------------------------------------------------------------------------------------ACCGUUGAGCCCUUUCUGA-----------   4        19
-----------CAGUUAGGGUUUAAUGGUUC-------------------------------------------------------------------------------------------------------------------------   3        20
----------CCAGUUAGGGUUUAAUGGU---------------------------------------------------------------------------------------------------------------------------   1        19
------------AGUUAGGGUUUAAUGGUUCU------------------------------------------------------------------------------------------------------------------------   1        20
-----------------------------------------------------------------------------------------------------AGGAAAAAUAACUGGAAAAGA------------------------------   1        21
--------------------------------------------------------------------------------------------------------------------------ACCGUUGAGCCCUUUCUGAUUU--------   1        22
Library = 3        Precision: [ total = 99.2% | 5p-arm = 99.1% | 3p-arm = 100.0% ]
----------CCAGUUAGGGUUUAAUGGUUC-------------------------------------------------------------------------------------------------------------------------   310      21
--------------------------------------------------------------------------------------------------------------------------ACCGUUGAGCCCUUUCUGAU----------   36       20
----------CCAGUUAGGGUUUAAUGGUUCU------------------------------------------------------------------------------------------------------------------------   22       22
--------------------------------------------------------------------------------------------------------------------------ACCGUUGAGCCCUUUCUG------------   13       18
--------------------------------------------------------------------------------------------------------------------------ACCGUUGAGCCCUUUCUGAUU---------   9        21
----------CCAGUUAGGGUUUAAUGGUU--------------------------------------------------------------------------------------------------------------------------   3        20
-----------CAGUUAGGGUUUAAUGGUUC-------------------------------------------------------------------------------------------------------------------------   3        20
--------------------------------------------------------------------------------------------------------------------------ACCGUUGAGCCCUUUCUGA-----------   2        19
----------CCAGUUAGGGUUUAAUGGUUCUA-----------------------------------------------------------------------------------------------------------------------   1        23
```

```
AUAAAC    c   UU                A   U  A           U    CC    UU     U     AAU
      UAAA CAG  AGGGUUUAAUGGUUcU -UC CA UUGUUUUUUUU GGAU  CAAA  UAAUC AUUUU   \
      AUuU GUC  UCCCGAGUUGCCaAGA  AG-GU AAUAAAAAGGA-CCUA  GUUU--AUUAG-UAAAA   U
UUAAUU    A   UU                AA     C                UU                 AAU
```

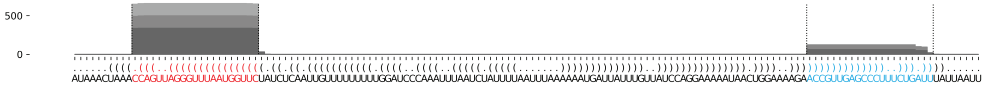

D. discoideum ddi-mir-1179

```
(((((((((((((.((.((((((((.(((((((((.((((((((((.(((........)))..)))))))))).))))))))))))))))))))))))))))))))        -56.04 kcal/mol
UUUAAUUCUUuGGGUCUCCAUUCAAAUUAAGUUcAGACUUAAAAAAUUUCCUUAGUUUGAAUUUUUUUUAGGUAUGAaCUUAAUUGGGUGGGACUAAaGAAUUAAA     count    length
Libraries combined          Precision: [ total = 98.8% | 5p-arm = 100.0% | 3p-arm = 92.3% ]
----------UGGGUCUCCAUUCAAAUUAAGUUC------------------------------------------------------------------------        65       24
----------UGGGUCUCCAUUCAAAUUAAGUU-------------------------------------------------------------------------        6        23
-----------------------------------------------------------------------------ACUUAAUUGGGUGGGACUAAA--------        8        21
------------------------------------------------------------------------UAUGAACUUAAUUGGGUGGGACU-----------        1        23
-----------------------------------------------------------------------------ACUUAAUUGGGUGGGACUAA---------        4        20
Library = 1        Precision: [ total = 100.0% | 5p-arm = 100.0% | 3p-arm = 100.0% ]
----------UGGGUCUCCAUUCAAAUUAAGUUC------------------------------------------------------------------------        14       24
----------UGGGUCUCCAUUCAAAUUAAGUU-------------------------------------------------------------------------        2        23
-----------------------------------------------------------------------------ACUUAAUUGGGUGGGACUAAA--------        2        21
Library = 2        Precision: [ total = 96.9% | 5p-arm = 100.0% | 3p-arm = 83.3% ]
----------UGGGUCUCCAUUCAAAUUAAGUUC------------------------------------------------------------------------        24       24
-----------------------------------------------------------------------------ACUUAAUUGGGUGGGACUAAA--------        3        21
----------UGGGUCUCCAUUCAAAUUAAGUU-------------------------------------------------------------------------        2        23
-----------------------------------------------------------------------------ACUUAAUUGGGUGGGACUAA---------        2        20
------------------------------------------------------------------------UAUGAACUUAAUUGGGUGGGACU-----------        1        23
Library = 3        Precision: [ total = 100.0% | 5p-arm = 100.0% | 3p-arm = 100.0% ]
----------UGGGUCUCCAUUCAAAUUAAGUUC------------------------------------------------------------------------        27       24
-----------------------------------------------------------------------------ACUUAAUUGGGUGGGACUAAA--------        3        21
----------UGGGUCUCCAUUCAAAUUAAGUU-------------------------------------------------------------------------        2        23
-----------------------------------------------------------------------------ACUUAAUUGGGUGGGACUAA---------        2        20
```

```
             G  U        A         G          U    CUU
UUUAAUUCUUuGG UC CCAUUCAA UUAAGUUcA ACUUAAAAAA -UUC   A
AAAUUAAGaAAUC-AG-GGUGGGUUA-AUUCaAGU UGGAUUUUUU  AAG   G
                                   A          UU   UUU
```

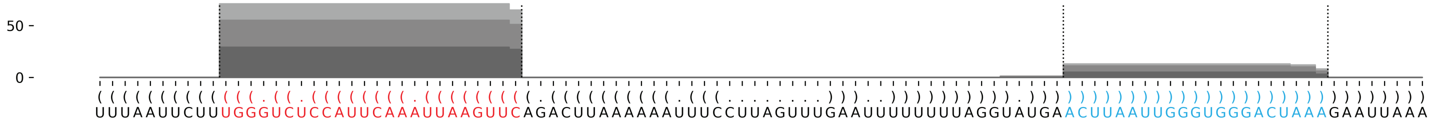

D. discoideum ddi-mir-7096

```
((((((((.(((((((((((((((.((((((((((((((((((((((((..........)))).))))))))))))))))))))).))))))))))))))).))))))))    -72.22 kcal/mol
UUAAACUGAGuCUGUGAACAAUCAAGAUUUgAUAAACACUAUUGAAAUUGAAAUUUUUUUCUAAUGGUGUUUAUCAaAUCUUAAUUGUUCACAGACcCAGUUUAA    count    length
Libraries combined         Precision: [ total = 99.5% | 5p-arm = 100.0% | 3p-arm = 99.5% ]
----------------------------------------------------------------------------AAUCUUAAUUGUUCACAGACC--------    386      21
----------------------------------------------------------------------------AAUCUUAAUUGUUCACAGACCC-------    15       22
----------UCUGUGAACAAUCAAGAUUUG--------------------------------------------------------------------------    7        21
----------------------------------------------------------------------------AAUCUUAAUUGUUCACAGACCCAG-----    1        24
----------------------------------------------------------------------------AAUCUUAAUUGUUCACAGAC---------    3        20
-------------------------------------------------------------------------------CUUAAUUGUUCACAGACC--------    1        18
Library = 1        Precision: [ total = 100.0% | 5p-arm = 100.0% | 3p-arm = 100.0% ]
----------------------------------------------------------------------------AAUCUUAAUUGUUCACAGACC--------    66       21
----------------------------------------------------------------------------AAUCUUAAUUGUUCACAGACCC-------    3        22
----------UCUGUGAACAAUCAAGAUUUG--------------------------------------------------------------------------    2        21
Library = 2        Precision: [ total = 100.0% | 5p-arm = 100.0% | 3p-arm = 100.0% ]
----------------------------------------------------------------------------AAUCUUAAUUGUUCACAGACC--------    72       21
----------------------------------------------------------------------------AAUCUUAAUUGUUCACAGACCC-------    5        22
----------UCUGUGAACAAUCAAGAUUUG--------------------------------------------------------------------------    2        21
Library = 3        Precision: [ total = 99.2% | 5p-arm = 100.0% | 3p-arm = 99.2% ]
----------------------------------------------------------------------------AAUCUUAAUUGUUCACAGACC--------    248      21
----------------------------------------------------------------------------AAUCUUAAUUGUUCACAGACCC-------    7        22
----------UCUGUGAACAAUCAAGAUUUG--------------------------------------------------------------------------    3        21
----------------------------------------------------------------------------AAUCUUAAUUGUUCACAGAC---------    3        20
----------------------------------------------------------------------------AAUCUUAAUUGUUCACAGACCCAG-----    1        24
-------------------------------------------------------------------------------CUUAAUUGUUCACAGACC--------    1        18
```

```
        A             C                         UUGA
UUAAACUG GuCUGUGAACAAU AAGAUUUgAUAAACACUAUU-GAAA    A
AAUUUGAC CAGACACUUGUUA UUCUAaACUAUUUGUGGUAA CUUU    A
        c             A                    U    UUUU
```

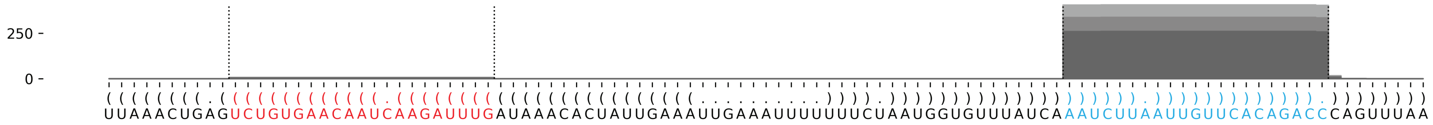

D. discoideum ddi-mir-1183

```
((((.((((((((((((((((((((((.(((((((.....(((((((((.(((((.((.(((((.(((....))).))))).))((....))))))).))))))))).....))))))).)))))))))))))))))))))).))))       -88.47 kcal/mol
UUAUACUAUAuAAGUGGUCGUUAAUUGCUGGcAGACCUCAGUUUACUCACACUAGACCACUGGCCGUCUUCUGGCUGCUAGCGGGCAAUGGCCUAGUAUGAGUAAAUGACCAUCUGCcACCAGUUAGCGGUCAUUUGUaUAGCAUGG       count    length
Libraries combined         Precision: [ total = 88.1% | 5p-arm = 88.1% | 3p-arm = 87.2% ]
----------UAAGUGGUCGUUAAUUGCUGGC-------------------------------------------------------------------------------------------------------------------       1471     22
-----------AAGUGGUCGUUAAUUGCUGGC-------------------------------------------------------------------------------------------------------------------       184      21
---------------------------------------------------------------------------------------------------------------------CACCAGUUAGCGGUCAUUUGUA--------       27       22
----------UAAGUGGUCGUUAAUUGC-----------------------------------------------------------------------------------------------------------------------       16       18
----------UAAGUGGUCGUUAAUUGCUGG--------------------------------------------------------------------------------------------------------------------       7        21
----------UAAGUGGUCGUUAAUUGCUG---------------------------------------------------------------------------------------------------------------------       7        20
--------------------------------AGACCUCAGUUUACUCACACU----------------------------------------------------------------------------------------------       5        21
--------UAUAAGUGGUCGUUAAUUGCU----------------------------------------------------------------------------------------------------------------------       3        21
----------UAAGUGGUCGUUAAUUGCU----------------------------------------------------------------------------------------------------------------------       8        19
------------AGUGGUCGUUAAUUGCUGGC-------------------------------------------------------------------------------------------------------------------       6        20
------------AGUGGUCGUUAAUUGCUGGCA------------------------------------------------------------------------------------------------------------------       2        21
----------------------------------------------------------------CGUCUUCUGGCUGCUAGC-----------------------------------------------------------------       1        18
---------------------------------------------------------------------UCUGGCUGCUAGCGGGCAAU----------------------------------------------------------       1        20
---------------------------------------------------------------------------------------------------------------------CACCAGUUAGCGGUCAUUUGU---------       5        21
---------------------------------------------------------------------------------------------------------------------CACCAGUUAGCGGUCAUU------------       2        18
--------------UGGUCGUUAAUUGCUGGC-------------------------------------------------------------------------------------------------------------------       1        18
-------------------------------CAGACCUCAGUUUACUCACACU----------------------------------------------------------------------------------------------       1        22
------------------------------------------------------------------------------------------------UAUGAGUAAAUGACCAUCUGC------------------------------       2        21
-------------------------------------------------------------------------------------------------AUGAGUAAAUGACCAUCUGCCACC--------------------------       1        24
---------------------------------------------------------------------------------------------------------------------------UUAGCGGUCAUUUGUAUAGC----       1        20
Library = 1        Precision: [ total = 86.6% | 5p-arm = 86.4% | 3p-arm = 93.8% ]
----------UAAGUGGUCGUUAAUUGCUGGC-------------------------------------------------------------------------------------------------------------------       497      22
-----------AAGUGGUCGUUAAUUGCUGGC-------------------------------------------------------------------------------------------------------------------       71       21
---------------------------------------------------------------------------------------------------------------------CACCAGUUAGCGGUCAUUUGUA--------       13       22
----------UAAGUGGUCGUUAAUUGC-----------------------------------------------------------------------------------------------------------------------       6        18
----------UAAGUGGUCGUUAAUUGCUGG--------------------------------------------------------------------------------------------------------------------       4        21
----------UAAGUGGUCGUUAAUUGCUG---------------------------------------------------------------------------------------------------------------------       4        20
--------------------------------AGACCUCAGUUUACUCACACU----------------------------------------------------------------------------------------------       4        21
--------UAUAAGUGGUCGUUAAUUGCU----------------------------------------------------------------------------------------------------------------------       2        21
----------UAAGUGGUCGUUAAUUGCU----------------------------------------------------------------------------------------------------------------------       2        19
------------AGUGGUCGUUAAUUGCUGGC-------------------------------------------------------------------------------------------------------------------       2        20
------------AGUGGUCGUUAAUUGCUGGCA------------------------------------------------------------------------------------------------------------------       1        21
----------------------------------------------------------------CGUCUUCUGGCUGCUAGC-----------------------------------------------------------------       1        18
---------------------------------------------------------------------UCUGGCUGCUAGCGGGCAAU----------------------------------------------------------       1        20
---------------------------------------------------------------------------------------------------------------------CACCAGUUAGCGGUCAUUUGU---------       1        21
---------------------------------------------------------------------------------------------------------------------CACCAGUUAGCGGUCAUU------------       1        18
Library = 2        Precision: [ total = 84.2% | 5p-arm = 84.7% | 3p-arm = 50.0% ]
----------UAAGUGGUCGUUAAUUGCUGGC-------------------------------------------------------------------------------------------------------------------       108      22
-----------AAGUGGUCGUUAAUUGCUGGC-------------------------------------------------------------------------------------------------------------------       18       21
----------UAAGUGGUCGUUAAUUGC-----------------------------------------------------------------------------------------------------------------------       2        18
----------UAAGUGGUCGUUAAUUGCU----------------------------------------------------------------------------------------------------------------------       1        19
------------AGUGGUCGUUAAUUGCUGGC-------------------------------------------------------------------------------------------------------------------       1        20
-------------------------------CAGACCUCAGUUUACUCACACU----------------------------------------------------------------------------------------------       1        22
---------------------------------------------------------------------------------------------------------------------CACCAGUUAGCGGUCAUUUGUA--------       1        22
---------------------------------------------------------------------------------------------------------------------------UUAGCGGUCAUUUGUAUAGC----       1        20
Library = 3        Precision: [ total = 89.6% | 5p-arm = 89.7% | 3p-arm = 85.7% ]
----------UAAGUGGUCGUUAAUUGCUGGC-------------------------------------------------------------------------------------------------------------------       866      22
-----------AAGUGGUCGUUAAUUGCUGGC-------------------------------------------------------------------------------------------------------------------       95       21
---------------------------------------------------------------------------------------------------------------------CACCAGUUAGCGGUCAUUUGUA--------       13       22
----------UAAGUGGUCGUUAAUUGC-----------------------------------------------------------------------------------------------------------------------       8        18
----------UAAGUGGUCGUUAAUUGCU----------------------------------------------------------------------------------------------------------------------       5        19
---------------------------------------------------------------------------------------------------------------------CACCAGUUAGCGGUCAUUUGU---------       4        21
----------UAAGUGGUCGUUAAUUGCUGG--------------------------------------------------------------------------------------------------------------------       3        21
```

D. discoideum ddi-mir-1183-continued

```
((((.((((((((((((((((((((((.(((((((.....(((((((((.(((((.((.(((((.(((....))).))))).))((....))))))).))))))))).....))))))).)))))))))))))))))))))).))))    -88.47 kcal/mol
UUAUACUAUAuAAGUGGUCGUUAAUUGCUGGcAGACCUCAGUUUACUCACACUAGACCACUGGCCGUCUUCUGGCUGCUAGCGGGCAAUGGCCUAGUAUGAGUAAAUGACCAUCUGCcACCAGUUAGCGGUCAUUUGUaUAGCAUGG    count   length
----------UAAGUGGUCGUUAAUUGCUG---------------------------------------------------------------------------------------------------------------------    3       20
------------AGUGGUCGUUAAUUGCUGGC-------------------------------------------------------------------------------------------------------------------    3       20
------------------------------------------------------------------------------------------------UAUGAGUAAAUGACCAUCUGC------------------------------    2       21
--------UAUAAGUGGUCGUUAAUUGCU----------------------------------------------------------------------------------------------------------------------    1       21
------------AGUGGUCGUUAAUUGCUGGCA------------------------------------------------------------------------------------------------------------------    1       21
--------------UGGUCGUUAAUUGCUGGC-------------------------------------------------------------------------------------------------------------------    1       18
--------------------------------AGACCUCAGUUUACUCACACU----------------------------------------------------------------------------------------------    1       21
-------------------------------------------------------------------------------------------------AUGAGUAAAUGACCAUCUGCCACC--------------------------    1       24
---------------------------------------------------------------------------------------------------------------------CACCAGUUAGCGGUCAUU------------    1       18
```

```
    A                      C       CCUCA         C     A    A     C   U
UUAU CUAUAuAAGUGGUCGUUAAUUG UGGcAGA     GUUUACUCA ACUAG --CC CUGGC GUC U
GGUA GAUaUGUUUACUGGCGAUUGAC AcCGUCU     UAAAUGAGU UGAUC   GG GAUCG CGG C
    C                      C       ACCAG         A     C G  C     U   U
                                                       G C
                                                      G   A
                                                       U-A
```

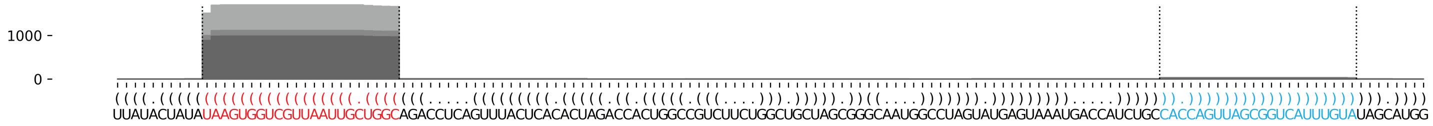

D. discoideum ddi-mir-7097

```
((((((((((.((((((((..((((((((((((((..((((..((((.(((.......)))))))..))))))))))))))))))..)))))))).))))))))))    -61.87 kcal/mol
UUUCGUUUUCuUCUCUACUAGUGCCGAAAUcAUUAAUAUUGCAUUCAUCAUUAUAUUUGUGUGAAAUCGAUUAAUGAuUUUGGCAGAAGUAGAGACGaAAACGAAA    count   length
Libraries combined          Precision: [ total = 91.2% | 5p-arm = 97.8% | 3p-arm = 90.1% ]
-----------------------------------------------------------------------------UUUUGGCAGAAGUAGAGACGA--------    404     21
----------UUCUCUACUAGUGCCGAAAUC---------------------------------------------------------------------------    80      21
-----------------------------------------------------------------------------UUUUGGCAGAAGUAGAGACG---------    36      20
-----------------------------------------------------------------------------UUUUGGCAGAAGUAGAGACGAAAA-----    46      24
-----------------------------------------------------------------------------UUUUGGCAGAAGUAGAGACGAAA------    40      23
-----------------------------------------------------------------------------UUUUGGCAGAAGUAGAGACGAA-------    25      22
----------UUCUCUACUAGUGCCGAAAU----------------------------------------------------------------------------    9       20
-----------------------------------------------------------------------------UUUUGGCAGAAGUAGAGAC----------    5       19
------------------------------------------------------------------------------UUUGGCAGAAGUAGAGACGA--------    3       20
---------CUUCUCUACUAGUGCCGAAAUC---------------------------------------------------------------------------    2       22
----------UUCUCUACUAGUGCCGAAA-----------------------------------------------------------------------------    1       19
----------------------------------------------------------------------------AUUUUGGCAGAAGUAGAGACGA--------    1       22
-----------------------------------------------------------------------------UUUUGGCAGAAGUAGAGACGAAAAC----    2       25
------------------------------------------------------------------------------UUUGGCAGAAGUAGAGACGAAA------    1       22
-------------------------------------------------------------------------------UUGGCAGAAGUAGAGACGA--------    1       19
--------------------------------------------------------------------------------UGGCAGAAGUAGAGACGAAAA-----    1       21
--------------------------------------------------------------------------------UGGCAGAAGUAGAGACGAA-------    1       19
Library = 1       Precision: [ total = 93.3% | 5p-arm = 97.4% | 3p-arm = 92.8% ]
-----------------------------------------------------------------------------UUUUGGCAGAAGUAGAGACGA--------    249     21
----------UUCUCUACUAGUGCCGAAAUC---------------------------------------------------------------------------    31      21
-----------------------------------------------------------------------------UUUUGGCAGAAGUAGAGACG---------    17      20
-----------------------------------------------------------------------------UUUUGGCAGAAGUAGAGACGAAAA-----    16      24
-----------------------------------------------------------------------------UUUUGGCAGAAGUAGAGACGAAA------    15      23
```

D. discoideum ddi-mir-7097-continued

```
((((((((((.((((((((..((((((((((((((..((((..((((.(((.......)))))))..))))))))))))))))))..)))))))).))))))))))       -61.87 kcal/mol
UUUCGUUUUCuUCUCUACUAGUGCCGAAAUcAUUAAUAUUGCAUUCAUCAUUAUAUUUGUGUGAAAUCGAUUAAUGAuUUUGGCAGAAGUAGAGACGaAAACGAAA       count    length
Libraries combined          Precision: [ total = 91.2% | 5p-arm = 97.8% | 3p-arm = 90.1% ]
-----------------------------------------------------------------------------UUUUGGCAGAAGUAGAGACGAA-------       12       22
----------UUCUCUACUAGUGCCGAAAU----------------------------------------------------------------------------       5        20
-----------------------------------------------------------------------------UUUUGGCAGAAGUAGAGAC----------       2        19
------------------------------------------------------------------------------UUUGGCAGAAGUAGAGACGA--------       2        20
---------CUUCUCUACUAGUGCCGAAAUC---------------------------------------------------------------------------       1        22
----------UUCUCUACUAGUGCCGAAA-----------------------------------------------------------------------------       1        19
----------------------------------------------------------------------------AUUUUGGCAGAAGUAGAGACGA--------       1        22
-----------------------------------------------------------------------------UUUUGGCAGAAGUAGAGACGAAAAC----       1        25
------------------------------------------------------------------------------UUUGGCAGAAGUAGAGACGAAA------       1        22
-------------------------------------------------------------------------------UUGGCAGAAGUAGAGACGA--------       1        19
--------------------------------------------------------------------------------UGGCAGAAGUAGAGACGAAAA-----       1        21
Library = 2        Precision: [ total = 89.7% | 5p-arm = 90.9% | 3p-arm = 89.4% ]
-----------------------------------------------------------------------------UUUUGGCAGAAGUAGAGACGA--------       31       21
----------UUCUCUACUAGUGCCGAAAUC---------------------------------------------------------------------------       9        21
-----------------------------------------------------------------------------UUUUGGCAGAAGUAGAGACGAAAA-----       5        24
-----------------------------------------------------------------------------UUUUGGCAGAAGUAGAGACGAAA------       4        23
-----------------------------------------------------------------------------UUUUGGCAGAAGUAGAGACG---------       3        20
-----------------------------------------------------------------------------UUUUGGCAGAAGUAGAGACGAA-------       3        22
---------CUUCUCUACUAGUGCCGAAAUC---------------------------------------------------------------------------       1        22
----------UUCUCUACUAGUGCCGAAAU----------------------------------------------------------------------------       1        20
-----------------------------------------------------------------------------UUUUGGCAGAAGUAGAGAC----------       1        19
Library = 3        Precision: [ total = 88.5% | 5p-arm = 100.0% | 3p-arm = 86.1% ]
-----------------------------------------------------------------------------UUUUGGCAGAAGUAGAGACGA--------       124      21
----------UUCUCUACUAGUGCCGAAAUC---------------------------------------------------------------------------       40       21
-----------------------------------------------------------------------------UUUUGGCAGAAGUAGAGACGAAAA-----       25       24
-----------------------------------------------------------------------------UUUUGGCAGAAGUAGAGACGAAA------       21       23
-----------------------------------------------------------------------------UUUUGGCAGAAGUAGAGACG---------       16       20
-----------------------------------------------------------------------------UUUUGGCAGAAGUAGAGACGAA-------       10       22
----------UUCUCUACUAGUGCCGAAAU----------------------------------------------------------------------------       3        20
-----------------------------------------------------------------------------UUUUGGCAGAAGUAGAGAC----------       2        19
-----------------------------------------------------------------------------UUUUGGCAGAAGUAGAGACGAAAAC----       1        25
------------------------------------------------------------------------------UUUGGCAGAAGUAGAGACGA--------       1        20
--------------------------------------------------------------------------------UGGCAGAAGUAGAGACGAA-------       1        19
```

```
          u        AG              AU    CA    U   UAU
UUUCGUUUUC UCUCUACU  UGCCGAAAUcAUUA  AUUG  UUCA CAU   \
AAAGCAAAaG AGAGAUGA  ACGGUUUuAGUAAU--UAGC  AAGU-GUG   A
          C        AG                    UA        UUU
```

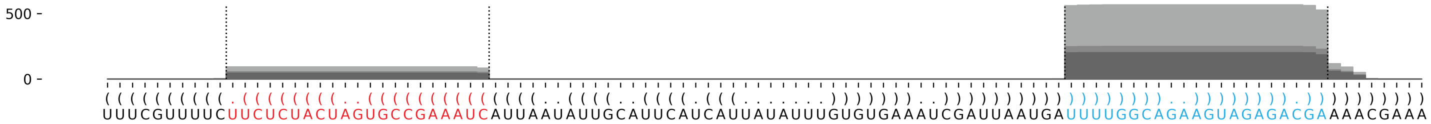

D. discoideum ddi-mir-1186 (ddi-mir-can_D1)

```
(((.((((((((((((((((((((((((((.((((((.((((.((((.((((.....)))).)))).))))))))))))))))))))))))))))))))))))).)))     -56.91 kcal/mol
UAUAGAAAUCuCUUUCUCUAAUUUCAUUUAuUAAAUUGUAAAUCACCCAUUUUAAUAAUGUGUGAGUUAUAUUUAAaAAAUGAAAUUAGAGAAAGGGaUUUUCAUG     count    length
Libraries combined         Precision: [ total = 97.8% | 5p-arm = 100.0% | 3p-arm = 97.3% ]
----------------------------------------------------------------------------AAAUGAAAUUAGAGAAAGGGA--------     60       21
----------------------------------------------------------------------------AAAUGAAAUUAGAGAAAGGGAU-------     46       22
----------UCUUUCUCUAAUUUCAUUUA---------------------------------------------------------------------------     1        20
----------------------------------------------------------------------------AAAUGAAAUUAGAGAAAGGGAUU------     1        23
----------UCUUUCUCUAAUUUCAUUUAUU-------------------------------------------------------------------------     18       22
----------UCUUUCUCUAAUUUCAUUUAU--------------------------------------------------------------------------     2        21
----------------------------------------------------------------------------AAAUGAAAUUAGAGAAAGGG---------     3        20
-----------------------------------------------------------------------------AAUGAAAUUAGAGAAAGGGAU-------     3        21
Library = 1        Precision: [ total = 100.0% | 5p-arm = 100.0% | 3p-arm = 100.0% ]
----------------------------------------------------------------------------AAAUGAAAUUAGAGAAAGGGA--------     11       21
----------------------------------------------------------------------------AAAUGAAAUUAGAGAAAGGGAU-------     9        22
----------UCUUUCUCUAAUUUCAUUUA---------------------------------------------------------------------------     1        20
----------------------------------------------------------------------------AAAUGAAAUUAGAGAAAGGGAUU------     1        23
Library = 2        Precision: [ total = 93.8% | 5p-arm = 100.0% | 3p-arm = 92.3% ]
----------------------------------------------------------------------------AAAUGAAAUUAGAGAAAGGGA--------     8        21
----------------------------------------------------------------------------AAAUGAAAUUAGAGAAAGGGAU-------     4        22
----------UCUUUCUCUAAUUUCAUUUAUU-------------------------------------------------------------------------     3        22
-----------------------------------------------------------------------------AAUGAAAUUAGAGAAAGGGAU-------     1        21
Library = 3        Precision: [ total = 97.9% | 5p-arm = 100.0% | 3p-arm = 97.5% ]
----------------------------------------------------------------------------AAAUGAAAUUAGAGAAAGGGA--------     41       21
----------------------------------------------------------------------------AAAUGAAAUUAGAGAAAGGGAU-------     33       22
----------UCUUUCUCUAAUUUCAUUUAUU-------------------------------------------------------------------------     15       22
----------------------------------------------------------------------------AAAUGAAAUUAGAGAAAGGG---------     3        20
----------UCUUUCUCUAAUUUCAUUUAU--------------------------------------------------------------------------     2        21
-----------------------------------------------------------------------------AAUGAAAUUAGAGAAAGGGAU-------     2        21
```

```
   A                          A      U    A    C    UU
UAU GAAAUCuCUUUCUCUAAUUUCAUUU uUAAAU GUAA UCAC CAUU  \
GUA UUUUaGGGAAAGAGAUUAAAGUAAa-AAUUUA-UAUU AGUG GUAA  A
   C                                     G    U    UA
```

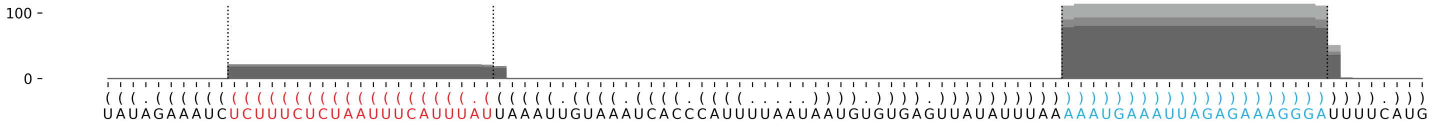

D. firmibasis dfi-mir-1190

((.((((((((((((((...(((((..........(((((((((.......(((((((((...)))))))))(((((.(((((.....(((((.......))))))))))).))))))))))))))).......)))))..))))))))))))))).)) -142.84 kcal/mol

CAACCAAAUCaAAAUUAUCAAAUGUCCUCAGcAAGAUAAUUCACAUAAUUCUUUAUUAAAUUCUUUUUUAAAUGGGUUAACUCUGAUCUAAAAUAUUAACAAAAUUAUUACUAUAAUAAUUUAUAAAUUGACCAUAAUCAUUUCUUUUUUAAAUGGGUUAUUUUAUAUUUAGAUCCAAAUAAUAUUUUUAAAGAAUUUAAUAAACAAUUAUGUGAAUUAUCUUGCuGAGGACAUUUGAUAAUUUUGaUUUGGUUG

Libraries combined Precision: [total = 76.9% | 5p-arm = 51.6% | 3p-arm = 78.1%]

read	count	length
UGAGGACAUUUGAUAAUUUUGA	59509	22
UGAGGACAUUUGAUAAUUUUG	30890	21
CUGAGGACAUUUGAUAAUUUU	9961	21
UGAGGACAUUUGAUAAUUUUGAU	5278	23
UGCUGAGGACAUUUGAUAAUU	4367	21
AAAAUUAUCAAAUGUCCUCAGC	2990	22
GCUGAGGACAUUUGAUAAUUU	2755	21
UUGCUGAGGACAUUUGAUAAU	2676	21
AAAUUAUCAAAUGUCCUCAGC	2111	21
UGAGGACAUUUGAUAAUUUU	1774	20
CUGAGGACAUUUGAUAAUUU	1633	20
CAAUUAUGUGAAUUAUCUUGC	996	21
CUGAGGACAUUUGAUAAUUUUG	768	22
UGAGGACAUUUGAUAAUUU	657	19
UGCUGAGGACAUUUGAUAAUUU	568	22
AUCUUGCUGAGGACAUUUGAU	453	21
UCUUGCUGAGGACAUUUGAUA	370	21
AAUUAUCAAAUGUCCUCAGC	388	20
UGAGGACAUUUGAUAAUUUUGAUU	392	24
ACAAUUAUGUGAAUUAUCUUGC	231	22
UUGCUGAGGACAUUUGAUAAUU	337	22
UGAGGACAUUUGAUAAUU	208	18
UGCUGAGGACAUUUGAUAAU	254	20
AAUUAUCAAAUGUCCUCAGCA	230	21
GAGGACAUUUGAUAAUUUUGA	206	21
GCUGAGGACAUUUGAUAAUU	221	20
CUGAGGACAUUUGAUAAUU	142	19
ACAAUUAUGUGAAUUAUCUUG	152	21
CUGAGGACAUUUGAUAAUUUUGA	133	23
AAAAUUAUCAAAUGUCCUCAG	127	21
AUCUUGCUGAGGACAUUUGAUA	97	22
UGAAUUAUCUUGCUGAGGAC	112	20
GAGGACAUUUGAUAAUUUUG	115	20
AGGACAUUUGAUAAUUUUGAU	78	21
AAAUUAUCAAAUGUCCUCAG	61	20
AAUUAUCUUGCUGAGGACAUU	75	21
GUGAAUUAUCUUGCUGAGGAC	64	21
CAAUUAUGUGAAUUAUCUUGCU	72	22
AGGACAUUUGAUAAUUUUGAUU	53	22
UGAAUUAUCUUGCUGAGGACA	61	21
UCUUGCUGAGGACAUUUGAUAA	45	22
AUCUUGCUGAGGACAUUUGA	21	20
CUGAGGACAUUUGAUAAUUUUGAU	23	24
AUUAUCAAAUGUCCUCAGC	30	19
AUCUUGCUGAGGACAUUUGAUAA	29	23
UCUUGCUGAGGACAUUUGAU	29	20
UUGCUGAGGACAUUUGAUAA	43	20
AAUUAUGUGAAUUAUCUUGCU	26	21
AUUAUCUUGCUGAGGACAUU	24	20
UUGCUGAGGACAUUUGAUAAUUU	24	23
CAAUUAUGUGAAUUAUCUUG	34	20
AAAUUAUCAAAUGUCCUCAGCA	17	22
ACAAUUAUGUGAAUUAUCUUGCU	24	23
CUGAGGACAUUUGAUAAU	26	18
UAUCAAAUGUCCUCAGCAAGA	18	21
AUUAUCUUGCUGAGGACAUUU	13	21
UUAUCUUGCUGAGGACAUUUG	10	21
AAUUAUCAAAUGUCCUCAG	11	19
AAUUAUCAAAUGUCCUCAGCAA	8	22
AAUUAUGUGAAUUAUCUUGC	13	20
UAUCUUGCUGAGGACAUUUGA	10	21
UCUUGCUGAGGACAUUUGAUAAU	18	23
GCUGAGGACAUUUGAUAAUUUU	12	22

D. firmibasis dfi-mir-1190 continued

((.(((((((((((((((...(((((..........((((((((((.......((((((((...)))))))))(((((.(((((.....(((((.......)))))))))).)))))))))))))))).......))))).))))))))))))))).))) −142.84 kcal/mol

CAACCAAAUCaAAAUUAUCAAAUGUCCUCAGcAAGAUAAUUCACAUAAUUCUUUAUUAAAUUCUUUUUUAAAUGGGUUAACUCUGAUCUAAAAUAUUAACAAAAUUAUUACUAUAAUAAUUUAUAAAUUGACCAUAAUCAUUUCUUUUUUAAAUGGGUUAUUUAUAUUUAGAUCCAAAUAAUAUUUUUAAAGAAUUUAAUAAACAAUUAUGUGAAUUAUCUUGCuGAGGACAUUUGAUAAUUUUGaUUUGGUUG

Libraries combined Precision: [total = 76.9% | 5p-arm = 51.6% | 3p-arm = 78.1%]

read	count	length
GCUGAGGACAUUUGAUAAU	11	19
AGGACAUUUGAUAAUUUUGA	9	20
UCAAAUGUCCUCAGCAAGAUA	12	21
AUCUUGCUGAGGACAUUUGAUAAU	12	24
CUUGCUGAGGACAUUUGAUA	4	20
ACAUUUGAUAAUUUUGAUUUG	3	21
AACAAUUAUGUGAAUUAUCUUGCU	6	24
CAAUUAUGUGAAUUAUCU	2	18
AUUAUGUGAAUUAUCUUGC	4	19
AUGUGAAUUAUCUUGCUGAGG	17	21
UGAAUUAUCUUGCUGAGGACAUU	5	23
GAAUUAUCUUGCUGAGGACAU	6	21
GAAUUAUCUUGCUGAGGACA	6	20
AAUUAUCUUGCUGAGGACAU	4	20
UGCUGAGGACAUUUGAUAA	6	19
GAGGACAUUUGAUAAUUUU	5	19
UUUGAUAAUUUUGAUUUGGUU	5	21
UUGAUAAUUUUGAUUUGGUUG	4	21
UCAAAAUUAUCAAAUGUCCUC	1	21
AAAAUUAUCAAAUGUCCUCAGCA	3	23
AAUUAUCAAAUGUCCUCAGCAAG	2	23
UUAUCAAAUGUCCUCAGC	1	18
UUAUCAAAUGUCCUCAGCA	3	19
UAUCAAAUGUCCUCAGCAAG	2	20
UCCUCAGCAAGAUAAUUCACA	2	21
AAGAUAAUUCACAUAAUUCUU	1	21
AAGAUAAUUCACAUAAUUCU	4	20
AAAUGGGUUAACUCUGAUCUA	1	21
AACAAUUAUGUGAAUUAUCUUGC	4	23
CAAUUAUGUGAAUUAUCUUGCUGA	1	24
AUUAUGUGAAUUAUCUUGCUG	7	21
AUGUGAAUUAUCUUGCUGAGGA	2	22
AUGUGAAUUAUCUUGCUGA	1	19
UGUGAAUUAUCUUGCUGAG	1	19
UGAAUUAUCUUGCUGAGGACAU	3	22
GAAUUAUCUUGCUGAGGACAUU	2	22
AAUUAUCUUGCUGAGGACA	2	19
AUUAUCUUGCUGAGGACAUUUGAU	1	24
UUAUCUUGCUGAGGACAUUUGAU	2	23
UAUCUUGCUGAGGACAUUUGAU	2	22
AUCUUGCUGAGGACAUUUG	1	19
CUUGCUGAGGACAUUUGAUAAU	3	22
UUGCUGAGGACAUUUGAUA	4	19
UGAGGACAUUUGAUAAUUUUGAUUU	10	25
GAGGACAUUUGAUAAUUUUGAUU	3	23
AGGACAUUUGAUAAUUUUG	5	19
GGACAUUUGAUAAUUUUGAUU	6	21
CAUUUGAUAAUUUUGAUUUGG	1	21
UCAAAAUUAUCAAAUGUCCUCAGC	1	24
UCAAAAUUAUCAAAUGUCCU	1	20
AAAUUAUCAAAUGUCCUCAGCAA	1	23
AUUAUCAAAUGUCCUCAG	3	18
AUUAUCAAAUGUCCUCAGCAA	1	21
AUUAUCAAAUGUCCUCAGCA	2	20
UUAUCAAAUGUCCUCAGCAAG	4	21
AUCAAAUGUCCUCAGCAAGAUA	1	22
AUCAAAUGUCCUCAGCAAGAUAAUUCACA	1	29
UCAAAUGUCCUCAGCAAGAU	1	20
CAAAUGUCCUCAGCAAGAUA	3	20
UGUCCUCAGCAAGAUAAUUC	1	20
CUCAGCAAGAUAAUUCACAUA	1	21
CAAGAUAAUUCACAUAAUUCU	1	21
CACAUAAUUCUUUAUUAAAUU	1	21

D. firmibasis dfi-mir-1190 continued

((.(((((((((((((((...(((((..........((((((((((.......(((((((((...)))))))))(((((.(((((.....(((((.......))))))))))).))))))))))))))).......)))))..))))))))))))))).))) −142.84 kcal/mol

CAACCAAAUCaAAAUUAUCAAAUGUCCUCAGcAAGAUAAUUCACAUAAUUCUUUAUUAAAUUCUUUUUUAAAUGGGUUAACUCUGAUCUAAAAUAUUAACAAAAUUAUUACUAUAAUAAUUUAUAAAUUGACCAUAAUCAUUUCUUUUUAAAUGGGUUAUUUUAUAUUUUAGAUCCAAAUAAUAUUUUAAAGAAUUUAAUAAACAAUUAUGUGAAUUAUCUUGCuGAGGACAUUUGAUAAUUUUGaUUUGGUUG

Libraries combined Precision: [total = 76.9% | 5p-arm = 51.6% | 3p-arm = 78.1%]

read	count	length
CUUUUUUAAAUGGGUUAACUC	1	21
AACAAUUAUGUGAAUUAUCUUG	13	22
ACAAUUAUGUGAAUUAUC	1	18
CAAUUAUGUGAAUUAUCUUGCUG	1	23
UUAUGUGAAUUAUCUUGCUGA	1	21
UAUGUGAAUUAUCUUGCUGAG	2	21
UAUGUGAAUUAUCUUGCUGAGG	2	22
AUGUGAAUUAUCUUGCUGAG	1	20
GUGAAUUAUCUUGCUGAGGACA	1	22
UGAAUUAUCUUGCUGAGGA	1	19
GAAUUAUCUUGCUGAGGAC	1	19
AAUUAUCUUGCUGAGGACAUUU	1	22
AUUAUCUUGCUGAGGACAU	2	19
AUUAUCUUGCUGAGGACAUUUGA	1	23
UAUCUUGCUGAGGACAUUUGAUA	1	23
UAUCUUGCUGAGGACAUUUG	1	20
UCUUGCUGAGGACAUUUGAUAAUU	1	24
CUUGCUGAGGACAUUUGAUAA	6	21
UGCUGAGGACAUUUGAUAAUUUU	1	23
GCUGAGGACAUUUGAUAA	1	18
GAGGACAUUUGAUAAUUU	5	18
GAGGACAUUUGAUAAUUUUGAU	8	22
AUUUGAUAAUUUUGAUUUGGU	1	21
AUUUGAUAAUUUUGAUUUGGUU	1	22
UUGAUAAUUUUGAUUUGGU	1	19

```
                                                  C                UUU      GGUUAACUCU           AUUAACA          C
CAACCAAAUCaAAAUUAUCAAAUGUCCUCAGcAAGAUAAUUCACAUAAUU UUUAUUAAAUUCUUU   AAAUG          GAUCUAAAAU       AAAUUAUUA U
GUUGGUUUaGUUUUAAUAGUUUACAGGAGuCGUUCUAUUAAGUGUAUUAA AAAUAAUUUAAGAAA  -UUUAU        ---CUAGAUUUUA---\   AUUGGGUAAA /
                                                  C                UU       AAUAAAC                   AU           A
                                                                                                      UA
                                                                                                      UA
                                                                                                      UA       AUAAU      CUU
                                                                                                    U   UUGACC      CAUUU    U
                                                                                                     \--AUUGG-----GUAAA    /
                                                                                                                        UUU
```

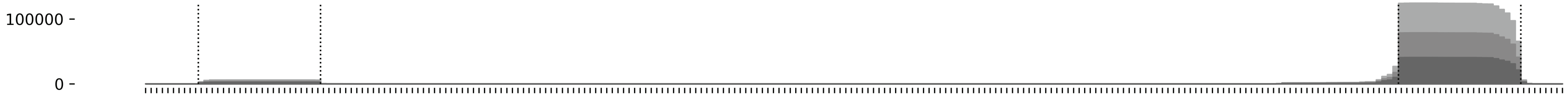

((.(((((((((((((((...(((((..........((((((((((.......(((((((((...)))))))))(((((.(((((.....(((((.......))))))))))).))))))))))))))).......)))))..))))))))))))))).))

CAACCAAAUCAAAAUUAUCAAAUGUCCUCAGCAAGAUAAUUCACAUAAUUCUUUAUUAAAUUCUUUUUUAAAUGGGUUAACUCUGAUCUAAAAUAUUAACAAAAUUAUUACUAUAAUAAUUUAUAAAUUGACCAUAAUCAUUUCUUUUUAAAUGGGUUAUUUUAUAUUUUAGAUCCAAAUAAUAUUUUAAAGAAUUUAAUAAACAAUUAUGUGAAUUAUCUUGCUGAGGACAUUUGAUAAUUUUGAUUUGGUUG

D. firmibasis dfi-mir-1191

```
((((((((((((((((.(((.(((((((((.((((((((((((((((((.(..(((.(((((.(.(((((((.((((((......)))))))......))))))).))))))...)))..)...)))))....)))))))))))))).))))))))).))).))))))))))))))))))   -95.3 kcal/mol
UCAUUAUGCAaACAAGUGCAAUCAGGAAAUaAAUACUAUAACUGAGCAAGUAUCAUUUCACAUCAAACAAACAAGAAGAAUUAAAAAGAGUUAGUUUAUUGGCUCUUGUUGGUUGAUCAUUGAUUUUUAAUACUGCGGUUGUUCAGUUAUAGUUUUUAuUUCCUCGUUACACUUGUUUgCAUAAUGA   count   length
Libraries combined    Precision: [ total = 97.1% | 5p-arm = 44.6% | 3p-arm = 97.8% ]
```

read	count	length
UUUCCUCGUUACACUUGUUUG	74616	21
UUUCCUCGUUACACUUGUUUGC	11194	22
UUUCCUCGUUACACUUGUUUGCA	5823	23
AACAAGUGCAAUCAGGAAAUA	464	21
UUUCCUCGUUACACUUGUUU	634	20
CAAACAAGUGCAAUCAGGAAAUA	328	23
UUUCCUCGUUACACUUGUUUGCAU	526	24
UAUUUCCUCGUUACACUUGUU	334	21
AAACAAGUGCAAUCAGGAAAUA	228	22
UAUUUCCUCGUUACACUUGUUU	337	22
UUUCCUCGUUACACUUGUU	281	19
AACAAGUGCAAUCAGGAAAU	89	20
UUCCUCGUUACACUUGUUUGCA	156	22
UCCUCGUUACACUUGUUUGCA	119	21
CAAACAAGUGCAAUCAGGAAAU	83	22
UUCCUCGUUACACUUGUUUG	89	20
UUAUUUCCUCGUUACACUUGU	76	21
UUCCUCGUUACACUUGUUUGC	81	21
UUUUAUUUCCUCGUUACACUUG	45	22
AUUUCCUCGUUACACUUGUUU	51	21
AAACAAGUGCAAUCAGGAAAU	30	21
UCCUCGUUACACUUGUUUGC	37	20
UUUUAUUUCCUCGUUACACUU	20	21
UUUUAUUUCCUCGUUACACU	31	20
UUUCCUCGUUACACUUGU	30	18
UAUUUCCUCGUUACACUUGUUUG	7	23
UCCUCGUUACACUUGUUUG	29	19
UUUCCUCGUUACACUUGUUUGCAUA	19	25
CAAACAAGUGCAAUCAGGAAA	7	21
AACAAGUGCAAUCAGGAAA	4	19
UUAUUUCCUCGUUACACUUGUU	10	22
UAUUUCCUCGUUACACUUGU	18	20
UUCCUCGUUACACUUGUU	3	18
ACAAGUGCAAUCAGGAAAUA	8	20
UUCCUCGUUACACUUGUUUGCAU	11	23
GCAAACAAGUGCAAUCAGGAAAU	2	23
AACAAGUGCAAUCAGGAAAUAA	3	22
ACAAGUGCAAUCAGGAAAU	1	19
UCAGGAAAUAAAUACUAUAAC	1	21
UAUUGGCUCUUGUUGGUUGAUCAUU	1	25
UGCGGUUGUUCAGUUAUAGUU	4	21
CGGUUGUUCAGUUAUAGUUU	1	20
AGUUAUAGUUUUUAUUUCCUC	1	21
UUAUAGUUUUUAUUUCCUCGUU	1	22
GUUUUUAUUUCCUCGUUACACU	6	22
UUUAUUUCCUCGUUACACUUG	1	21
UUAUUUCCUCGUUACACUUG	13	20
UAUUUCCUCGUUACACUUGUUUGC	1	24
UAUUUCCUCGUUACACUUG	3	19
UCCUCGUUACACUUGUUU	3	18
CCUCGUUACACUUGUUUGCA	5	20
CAAACAAGUGCAAUCAGGAAAUAA	1	24
AAACAAGUGCAAUCAGGAAAUAA	3	23

D. firmibasis dfi-mir-1191 continued

```
((((((((((((((((.(((.(((((((((.(((((((((((((((((((.(..(((.(((((.(.(((((((.((((((......)))))))......))))))).))))))...)))..)...)))))....)))))))))))))).)))))))))).))).))))))))))))))))) -95.3 kcal/mol
UCAUUAUGCAaACAAGUGCAAUCAGGAAAUaAAUACUAUAACUGAGCAAGUAUCAUUUCACAUCAAACAAACAAGAAGAAUUAAAAAGAGUUAGUUUAUUGGCUCUUGUUGGUUGAUCAUUGAUUUUUAAUACUGCGGUUGUUCAGUUAUAGUUUUUAuUUCCUCGUUACACUUGUUUgCAUAAUGA count length
Libraries combined    Precision: [ total = 97.1% | 5p-arm = 44.6% | 3p-arm = 97.8% ]
---------AAACAAGUGCAAUCAGGAAA-------------------------------------------------------------------------------------------------------------------------------------------------------------- 3 20
--------------AGUGCAAUCAGGAAAUAAAUA-------------------------------------------------------------------------------------------------------------------------------------------------------- 1 21
----------------------------------------------------------------------------------AAAAAGAGUUAGUUUAUUGGC------------------------------------------------------------------------------------ 1 21
-------------------------------------------------------------------------------------------------------------------------------------UGCGGUUGUUCAGUUAUAGUUU-------------------------------- 1 22
--------------------------------------------------------------------------------------------------------------------------------------GCGGUUGUUCAGUUAUAGUUU-------------------------------- 1 21
-----------------------------------------------------------------------------------------------------------------------------------------------------UAGUUUUAUUUCCUCGUUAC------------------ 4 21
--------------------------------------------------------------------------------------------------------------------------------------------------------UUUUAUUUCCUCGUUACACU--------------- 1 21
---------------------------------------------------------------------------------------------------------------------------------------------------------UUUAUUUCCUCGUUACAC---------------- 1 19
----------------------------------------------------------------------------------------------------------------------------------------------------------UUUAUUUCCUCGUUACACUUGU----------- 1 22
-------------------------------------------------------------------------------------------------------------------------------------------------------------AUUUCCUCGUUACACUUGUUUG-------- 10 22
-------------------------------------------------------------------------------------------------------------------------------------------------------------AUUUCCUCGUUACACUUGUU---------- 3 20
--------------------------------------------------------------------------------------------------------------------------------------------------------------UUUCCUCGUUACACUUGUUUGCAUAA--- 2 26
---------------------------------------------------------------------------------------------------------------------------------------------------------------UUCCUCGUUACACUUGUUU--------- 2 19
---------------------------------------------------------------------------------------------------------------------------------------------------------------UUCCUCGUUACACUUGUUUGCAUA---- 1 24
----------------------------------------------------------------------------------------------------------------------------------------------------------------UCCUCGUUACACUUGUUUGCAU----- 8 22
-----------------------------------------------------------------------------------------------------------------------------------------------------------------CCUCGUUACACUUGUUUG-------- 3 18
-----------------------------------------------------------------------------------------------------------------------------------------------------------------CCUCGUUACACUUGUUUGC------- 1 19
-----------------------------------------------------------------------------------------------------------------------------------------------------------------CCUCGUUACACUUGUUUGCAU----- 4 21
--------------------------------------------------------------------------------------------------------------------------------------------------------------------CGUUACACUUGUUUGCAUAAU-- 1 21
---------------------------------------------------------------------------------------------------------------------------------------------------------------------GUUACACUUGUUUGCAUAAUG- 2 21
```

```
                  C   C          U                        C-- UU   C       A A       A            AA
UCAUUAUGCAaACAAGUG AAU AGGAAAUaAA ACUAUAACUGAGCA-----AGUAU   A  UCA --AUCAA C AACAAGA -----GAAUUAA  A
AGUAAUACgUUUGUUCAC UUG UCCUUuAUUU UGAUAUUGACUUGU     UCAUA   U  AGU   UAGUU-G UUGUUCU      UUUGAUU  G
                  A   C          U              UGGCG     AUU UU   UAC       G       CGGUUA       GA
```

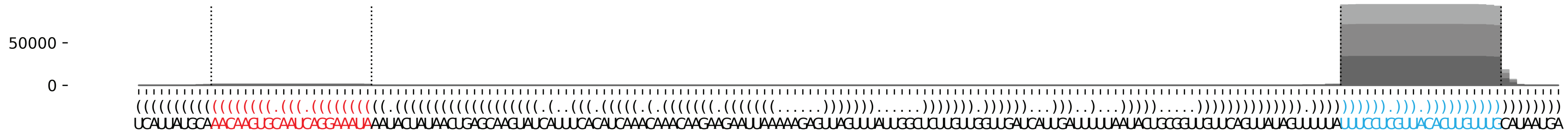

D. firmibasis dfi-mir-1192

```
((((((((.((.(((((((.((((((((((((((.((((...(((((((((........))))))))).)))).)))))))))))))).))))))).)).))))))))   -69.88 kcal/mol
UUUAUGCAGGuGUACCAAGACAAUCUCCAAgUACGUAUGGUAACUCAAUUGAAAAAUAAUGAUUGAGUACAUAAGUACUuGGAGAUUGGCUUGGUAAACaUGCAUAAA   count    length
Libraries combined         Precision: [ total = 84.5% | 5p-arm = 87.9% | 3p-arm = 76.9% ]
----------UGUACCAAGACAAUCUCCAAG-----------------------------------------------------------------------------   51       21
----UGCAGGUGUACCAAGACAAUC-----------------------------------------------------------------------------------   5        21
-------------------------------------------------------------------------------UGGAGAUUGGCUUGGUAAACA--------   9        21
--------------------------------------------------------------------------------GGAGAUUGGCUUGGUAAACAUG------   2        22
---------------------------------------------------------------------------UACUUGGAGAUUGGCUUGGUA------------   1        21
-------------------------------------------------------------------------------UGGAGAUUGGCUUGGUAAAC---------   10       20
--------------------------------------------------------------------------------GGAGAUUGGCUUGGUAAAC---------   1        19
------------UACCAAGACAAUCUCCAAGUA---------------------------------------------------------------------------   1        21
------------UACCAAGACAAUCUCCAAG-----------------------------------------------------------------------------   1        19
-----------------------------------------------------------------------------CUUGGAGAUUGGCUUGGUAAACA--------   2        23
-------------------------------------------------------------------------------UGGAGAUUGGCUUGGUAAA----------   1        19
Library = 1        Precision: [ total = 68.2% | 5p-arm = 80.0% | 3p-arm = 42.9% ]
----------UGUACCAAGACAAUCUCCAAG-----------------------------------------------------------------------------   12       21
----UGCAGGUGUACCAAGACAAUC-----------------------------------------------------------------------------------   3        21
-------------------------------------------------------------------------------UGGAGAUUGGCUUGGUAAACA--------   2        21
--------------------------------------------------------------------------------GGAGAUUGGCUUGGUAAACAUG------   2        22
---------------------------------------------------------------------------UACUUGGAGAUUGGCUUGGUA------------   1        21
-------------------------------------------------------------------------------UGGAGAUUGGCUUGGUAAAC---------   1        20
--------------------------------------------------------------------------------GGAGAUUGGCUUGGUAAAC---------   1        19
Library = 2        Precision: [ total = 92.0% | 5p-arm = 94.3% | 3p-arm = 86.7% ]
----------UGUACCAAGACAAUCUCCAAG-----------------------------------------------------------------------------   33       21
-------------------------------------------------------------------------------UGGAGAUUGGCUUGGUAAACA--------   7        21
-------------------------------------------------------------------------------UGGAGAUUGGCUUGGUAAAC---------   5        20
-----------------------------------------------------------------------------CUUGGAGAUUGGCUUGGUAAACA--------   2        23
----UGCAGGUGUACCAAGACAAUC-----------------------------------------------------------------------------------   1        21
------------UACCAAGACAAUCUCCAAGUA---------------------------------------------------------------------------   1        21
-------------------------------------------------------------------------------UGGAGAUUGGCUUGGUAAA----------   1        19
Library = 3        Precision: [ total = 83.3% | 5p-arm = 75.0% | 3p-arm = 100.0% ]
----------UGUACCAAGACAAUCUCCAAG-----------------------------------------------------------------------------   6        21
-------------------------------------------------------------------------------UGGAGAUUGGCUUGGUAAAC---------   4        20
----UGCAGGUGUACCAAGACAAUC-----------------------------------------------------------------------------------   1        21
------------UACCAAGACAAUCUCCAAG-----------------------------------------------------------------------------   1        19
```

```
        G  G       A              G    GUA        AAA
UUUAUGCA Gu UACCAAG CAAUCUCCAAgUAC UAUG   ACUCAAUUG   A
AAAUACGU CA AUGGUUC GUUAGAGGuUCAUG AUAC --UGAGUUAGU   A
        a  A       G              A    A          AAU
```

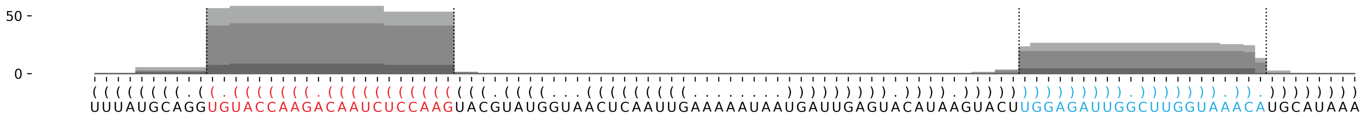

D. firmibasis dfi-mir-1195

```
(((.(((.(((((((((.(((((((((((((((((((..(((.(((((..((((((((....))))))))...........))))).)))..))))))))))))))))))).))))))))).))).)))    -69.84 kcal/mol
AGGUGAUCGUuGAUGUCGCCAAUGUAAUAAcAGAGGUUAAUUAUAUUAAAUAAUAUUUCAAUAAAUAUUAACAUUAACUUCUAAUACAAUAUACCUCUGUuAUUACAUUGGGGAUAUCAAcCGUCCCCU    count    length
Libraries combined         Precision: [ total = 97.7% | 5p-arm = 97.8% | 3p-arm = 34.5% ]
----------UGAUGUCGCCAAUGUAAUAAC--------------------------------------------------------------------------------------------------    530751   21
----------UGAUGUCGCCAAUGUAAUAA---------------------------------------------------------------------------------------------------    6469     20
---UGAUCGUUGAUGUCGCCAAUG---------------------------------------------------------------------------------------------------------    6127     21
----------UGAUGUCGCCAAUGUAAUAACA-------------------------------------------------------------------------------------------------    3143     22
---------UUGAUGUCGCCAAUGUAAUAAC--------------------------------------------------------------------------------------------------    1647     22
-----------GAUGUCGCCAAUGUAAUAAC--------------------------------------------------------------------------------------------------    1735     20
---------UUGAUGUCGCCAAUGUAAUAA---------------------------------------------------------------------------------------------------    701      21
------------AUGUCGCCAAUGUAAUAACAG------------------------------------------------------------------------------------------------    587      21
---------UUGAUGUCGCCAAUGUAAU-----------------------------------------------------------------------------------------------------    477      19
----------UGAUGUCGCCAAUGUAAU-----------------------------------------------------------------------------------------------------    483      18
-----------------------------------------------------------------------------------------------------AUUACAUUGGGGAUAUCAACC-------    398      21
------------AUGUCGCCAAUGUAAUAAC--------------------------------------------------------------------------------------------------    340      19
----------UGAUGUCGCCAAUGUAAUA----------------------------------------------------------------------------------------------------    305      19
----------------------------------------------------------------------------------------------------UAUUACAUUGGGGAUAUCAAC--------    188      21
---------UUGAUGUCGCCAAUGUAAUA----------------------------------------------------------------------------------------------------    165      20
-----------GAUGUCGCCAAUGUAAUAACA-------------------------------------------------------------------------------------------------    170      21
----------------------------------------------------------------------------------------------------UAUUACAUUGGGGAUAUCAA---------    77       20
------UCGUUGAUGUCGCCAAUGUA-------------------------------------------------------------------------------------------------------    68       20
-----------------------------------------------------------------------------------------------------AUUACAUUGGGGAUAUCAAC--------    55       20
-----------GAUGUCGCCAAUGUAAUAA---------------------------------------------------------------------------------------------------    59       19
-----AUCGUUGAUGUCGCCAAUGUA-------------------------------------------------------------------------------------------------------    39       21
----------UGAUGUCGCCAAUGUAAUAACAG------------------------------------------------------------------------------------------------    40       23
---UGAUCGUUGAUGUCGCCAA-----------------------------------------------------------------------------------------------------------    31       19
--------------GUCGCCAAUGUAAUAACAGAG----------------------------------------------------------------------------------------------    16       21
------------AUGUCGCCAAUGUAAUAACAGA-----------------------------------------------------------------------------------------------    27       22
-----------------------------------------------------------------------------------------------UCUGUUAUUACAUUGGGGAU--------------    12       20
---UGAUCGUUGAUGUCGCCAAU----------------------------------------------------------------------------------------------------------    20       20
-------------UGUCGCCAAUGUAAUAAC--------------------------------------------------------------------------------------------------    9        18
-----------------------------------------------------------------------------------------------------AUUACAUUGGGGAUAUCAA---------    18       19
---UGAUCGUUGAUGUCGCCA------------------------------------------------------------------------------------------------------------    9        18
----------------------------------------------------------------------------------------------------UAUUACAUUGGGGAUAUC-----------    9        18
-----AUCGUUGAUGUCGCCAAUG---------------------------------------------------------------------------------------------------------    12       19
-------------UGUCGCCAAUGUAAUAACAGAG----------------------------------------------------------------------------------------------    5        22
-----------GAUGUCGCCAAUGUAAUAACAG------------------------------------------------------------------------------------------------    8        22
--------------GUCGCCAAUGUAAUAACAG------------------------------------------------------------------------------------------------    5        19
--------------------------------------------------------------------------------------------------GUUAUUACAUUGGGGAUAUC-----------    4        20
---------------------------------------------------------------------------------------------------UUAUUACAUUGGGGAUAUCAA---------    4        21
-----------------------------------------------------------------------------------------------------AUUACAUUGGGGAUAUCAACCG------    7        22
---UGAUCGUUGAUGUCGCCAAUGU--------------------------------------------------------------------------------------------------------    11       22
------UCGUUGAUGUCGCCAAUG---------------------------------------------------------------------------------------------------------    2        18
----------UGAUGUCGCCAAUGUAAUAACAGA-----------------------------------------------------------------------------------------------    4        24
-----------GAUGUCGCCAAUGUAAUA----------------------------------------------------------------------------------------------------    6        18
------------AUGUCGCCAAUGUAAUAACA-------------------------------------------------------------------------------------------------    3        20
---------------UCGCCAAUGUAAUAACAGAGG---------------------------------------------------------------------------------------------    8        21
-------------------------------------------------------------------------------------------------UGUUAUUACAUUGGGGAUAUC-----------    8        21
----------------------------------------------------------------------------------------------------UAUUACAUUGGGGAUAUCAACC-------    5        22
------------------------------------------------------------------------------------------------------UUACAUUGGGGAUAUCAA---------    2        18
------------------------------------------------------------------------------------------------------UUACAUUGGGGAUAUCAAC--------    3        19
------------------------------------------------------------------------------------------------------UUACAUUGGGGAUAUCAACCG------    3        21
---UGAUCGUUGAUGUCGCCAAUGUA-------------------------------------------------------------------------------------------------------    3        23
----GAUCGUUGAUGUCGCCAAUG---------------------------------------------------------------------------------------------------------    7        20
------UCGUUGAUGUCGCCAAUGUAAU-----------------------------------------------------------------------------------------------------    1        22
-----------------------------------------------------------------------------------------------UCUGUUAUUACAUUGGGGAUA-------------    5        21
```

D. firmibasis dfi-mir-1195 continued

```
(((.(((.(((((((((.(((((((((((((((((((..(((.(((((..((((((((....))))))))...........))))).)))..))))))))))))))))))).))))))))).))).)))   -69.84 kcal/mol
AGGUGAUCGUuGAUGUCGCCAAUGUAAUAAcAGAGGUUAAUUAUAUUAAAUAAUAUUUCAAUAAAUAUUAACAUUAACUUCUAAUACAAUAUACCUCUGUuAUUACAUUGGGGAUAUCAAcCGUCCCCU   count   length
-----------------------------------------------------------------------------------------------------AUUACAUUGGGGAUAUCAACCGU-----   1       23
-----------------------------------------------------------------------------------------------------AUUACAUUGGGGAUAUCA----------   2       18
AGGUGAUCGUUGAUGUCGC--------------------------------------------------------------------------------------------------------------   1       19
--GUGAUCGUUGAUGUCGCCAAUG---------------------------------------------------------------------------------------------------------   1       22
-----AUCGUUGAUGUCGCCAAUGU--------------------------------------------------------------------------------------------------------   2       20
------UCGUUGAUGUCGCCAAUGUAA------------------------------------------------------------------------------------------------------   3       21
-------CGUUGAUGUCGCCAAUGUAAU-----------------------------------------------------------------------------------------------------   2       21
--------GUUGAUGUCGCCAAUGUAAU-----------------------------------------------------------------------------------------------------   1       20
--------GUUGAUGUCGCCAAUGUAAUA----------------------------------------------------------------------------------------------------   1       21
---------UUGAUGUCGCCAAUGUAA------------------------------------------------------------------------------------------------------   1       18
------------AUGUCGCCAAUGUAAUAA---------------------------------------------------------------------------------------------------   1       18
-------------UGUCGCCAAUGUAAUAACAGA-----------------------------------------------------------------------------------------------   4       21
--------------GUCGCCAAUGUAAUAACAGA-----------------------------------------------------------------------------------------------   1       20
---------------UCGCCAAUGUAAUAACAGAG----------------------------------------------------------------------------------------------   1       20
--------------------------------------------------------------------------------------CAAUAUACCUCUGUUAUUACAU---------------------   1       22
-----------------------------------------------------------------------------------------UAUACCUCUGUUAUUACAUUG-------------------   2       21
------------------------------------------------------------------------------------------AUACCUCUGUUAUUACAUUG-------------------   1       20
-----------------------------------------------------------------------------------------------UCUGUUAUUACAUUGGGGA---------------   1       19
-----------------------------------------------------------------------------------------------UCUGUUAUUACAUUGGGGAUAU------------   1       22
----------------------------------------------------------------------------------------------------UAUUACAUUGGGGAUAUCAACCGUC----   1       25
------------------------------------------------------------------------------------------------------UUACAUUGGGGAUAUCAACC-------   1       20
```

```
   U   C         G                   UA   A     AA                 C
AGG GAU GUuGAUGUC CCAAUGUAAUAAcAGAGGU  AUU UAUUA  ---------UAAUAUUU A
UCC CUG cAACUAUAG GGUUACAUUAuUGUCUCCA  UAA AUAAU           AUUAUAAA A
   C   C         G                   UA   C     CUUCAAUUACA        U
```

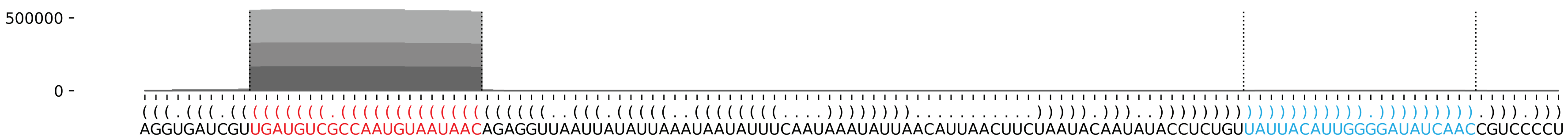

D. firmibasis dfi-mir-1196

```
(((((((((((((((((((((.((((((((((((((((((((((((...((((........)))).))))).....))))))))))))))))))).)))))))))))))))))))))   -75.37 kcal/mol
AAAAAAAACAuAGUGAAGAUGGGUAGAAGAuAAAAGCUGGUUUUGAAAAUUCAGAGAAUGGUGGGGUUAGAAAAUUACCAGCUUUUAUcUUCUACACAUCUUCACUAUgUUUUUUUU   count    length
Libraries combined         Precision: [ total = 76.8% | 5p-arm = 73.1% | 3p-arm = 79.1% ]
----------UAGUGAAGAUGGGUAGAAGAU--------------------------------------------------------------------------------------   18       21
----------------------------------------------------------------------------------------CUUCUACACAUCUUCACUAUG--------   34       21
--------------------------------------------------------------------------------------AUCUUCUACACAUCUUCACUA----------   2        21
-----------------------------------------------------------------------------------------UUCUACACAUCUUCACUAUGU-------   3        21
----------UAGUGAAGAUGGGUAGAAGAUA-------------------------------------------------------------------------------------   1        22
-----------------GAUGGGUAGAAGAUAAAAGCU-------------------------------------------------------------------------------   3        21
------------------AUGGGUAGAAGAUAAAAGCU-------------------------------------------------------------------------------   1        20
-----------AGUGAAGAUGGGUAGAAGAU--------------------------------------------------------------------------------------   1        20
----------------AGAUGGGUAGAAGAUAAAAGC--------------------------------------------------------------------------------   2        21
------------------------------------------------------------------------AAUUACCAGCUUUUAUCUUCUA-----------------------   1        22
-------------------------------------------------------------------------------------UAUCUUCUACACAUCUUCACU-----------   2        21
---------------------------------------------------------------------------------------UCUUCUACACAUCUUCACUAUG--------   1        22
Library = 1        Precision: [ total = 64.7% | 5p-arm = 75.0% | 3p-arm = 55.6% ]
----------UAGUGAAGAUGGGUAGAAGAU--------------------------------------------------------------------------------------   5        21
----------------------------------------------------------------------------------------CUUCUACACAUCUUCACUAUG--------   5        21
--------------------------------------------------------------------------------------AUCUUCUACACAUCUUCACUA----------   2        21
-----------------------------------------------------------------------------------------UUCUACACAUCUUCACUAUGU-------   2        21
----------UAGUGAAGAUGGGUAGAAGAUA-------------------------------------------------------------------------------------   1        22
-----------------GAUGGGUAGAAGAUAAAAGCU-------------------------------------------------------------------------------   1        21
------------------AUGGGUAGAAGAUAAAAGCU-------------------------------------------------------------------------------   1        20
Library = 2        Precision: [ total = 87.5% | 5p-arm = 71.4% | 3p-arm = 100.0% ]
----------------------------------------------------------------------------------------CUUCUACACAUCUUCACUAUG--------   9        21
----------UAGUGAAGAUGGGUAGAAGAU--------------------------------------------------------------------------------------   5        21
----------------AGAUGGGUAGAAGAUAAAAGC--------------------------------------------------------------------------------   1        21
-----------------GAUGGGUAGAAGAUAAAAGCU-------------------------------------------------------------------------------   1        21
Library = 3        Precision: [ total = 77.8% | 5p-arm = 72.7% | 3p-arm = 80.0% ]
----------------------------------------------------------------------------------------CUUCUACACAUCUUCACUAUG--------   20       21
----------UAGUGAAGAUGGGUAGAAGAU--------------------------------------------------------------------------------------   8        21
-------------------------------------------------------------------------------------UAUCUUCUACACAUCUUCACU-----------   2        21
-----------AGUGAAGAUGGGUAGAAGAU--------------------------------------------------------------------------------------   1        20
----------------AGAUGGGUAGAAGAUAAAAGC--------------------------------------------------------------------------------   1        21
-----------------GAUGGGUAGAAGAUAAAAGCU-------------------------------------------------------------------------------   1        21
------------------------------------------------------------------------AAUUACCAGCUUUUAUCUUCUA-----------------------   1        22
---------------------------------------------------------------------------------------UCUUCUACACAUCUUCACUAUG--------   1        22
-----------------------------------------------------------------------------------------UUCUACACAUCUUCACUAUGU-------   1        21
```

```
                     G                              AAA     GAG
AAAAAAAACAuAGUGAAGAUG GUAGAAGAuAAAAGCUGGU-----UUUGA   UUCA   A
UUUUUUUUgUAUCACUUCUAC CAUCUUcUAUUUUCGACCA     AGAUU --GGGU   A
                     A                   UUAAA     G      GGU
```

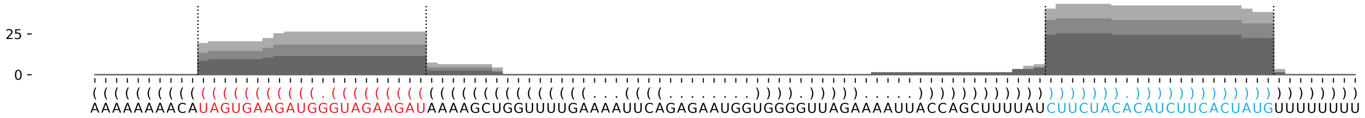

D. lacteum dla-mir-1197

```
(((((((((((((((((((..((((((((((((((((((((((.(((((..(((....(((((((........))))).))....)))..))))).))))))...)))))))))))))))))))))))))))))))))))      -93.54 kcal/mol
AUGAGAUGGUcUGAGAUAUAUGUGAGUAAUGAuAGGUAUCAUUCCUGCUUACUAGAAUUCUUAUUAAAUACAAAAUAAUGAUAGAUGGGUAGCAGUAAUGAUUGAACCUAUcAUUACUCAUAUAUCUCAGAcCAUCUCAU      count    length
Libraries combined         Precision: [ total = 94.3% | 5p-arm = 83.3% | 3p-arm = 96.6% ]
---------------------------------------------------------------------------------------------------------------CAUUACUCAUAUAUCUCAGAC--------      21       21
----------CUGAGAUAUAUGUGAGUAAUGA------------------------------------------------------------------------------------------------------------      2        22
---------UCUGAGAUAUAUGUGAGUAAUGAU-----------------------------------------------------------------------------------------------------------      1        24
----------CUGAGAUAUAUGUGAGUAAUGAU-----------------------------------------------------------------------------------------------------------      2        23
----------------------------------------------------------------------------------------------------------------AUUACUCAUAUAUCUCAGACC-------      1        21
----------CUGAGAUAUAUGUGAGUAAUG-------------------------------------------------------------------------------------------------------------      1        21
---------------------------------------------------------------------------------------------------------------CAUUACUCAUAUAUCUCAGACC-------      7        22
Library = 1        Precision: [ total = 80.0% | 5p-arm = 75.0% | 3p-arm = 83.3% ]
---------------------------------------------------------------------------------------------------------------CAUUACUCAUAUAUCUCAGAC--------      5        21
----------CUGAGAUAUAUGUGAGUAAUGA------------------------------------------------------------------------------------------------------------      2        22
---------UCUGAGAUAUAUGUGAGUAAUGAU-----------------------------------------------------------------------------------------------------------      1        24
----------CUGAGAUAUAUGUGAGUAAUGAU-----------------------------------------------------------------------------------------------------------      1        23
----------------------------------------------------------------------------------------------------------------AUUACUCAUAUAUCUCAGACC-------      1        21
Library = 2        Precision: [ total = 100.0% | 5p-arm = 100.0% | 3p-arm = 100.0% ]
---------------------------------------------------------------------------------------------------------------CAUUACUCAUAUAUCUCAGAC--------      9        21
---------------------------------------------------------------------------------------------------------------CAUUACUCAUAUAUCUCAGACC-------      5        22
----------CUGAGAUAUAUGUGAGUAAUGAU-----------------------------------------------------------------------------------------------------------      1        23
Library = 3        Precision: [ total = 100.0% | 5p-arm = 100.0% | 3p-arm = 100.0% ]
---------------------------------------------------------------------------------------------------------------CAUUACUCAUAUAUCUCAGAC--------      7        21
---------------------------------------------------------------------------------------------------------------CAUUACUCAUAUAUCUCAGACC-------      2        22
----------CUGAGAUAUAUGUGAGUAAUG-------------------------------------------------------------------------------------------------------------      1        21
```

```
                   AU                        C     UA    GAAU        AAA
AUGAGAUGGUcUGAGAUAU  GUGAGUAAUGAuAGGU---AUCAUU CUGCU  CUA    UC-UUAUU   U
UACUCUACcAGACUCUAUA--UACUCAUUAcUAUCCA   UAGUAA GACGA  GGU    AG AAUAA   A
                                     AGU      U     UG    AGAU  U     AAC
```

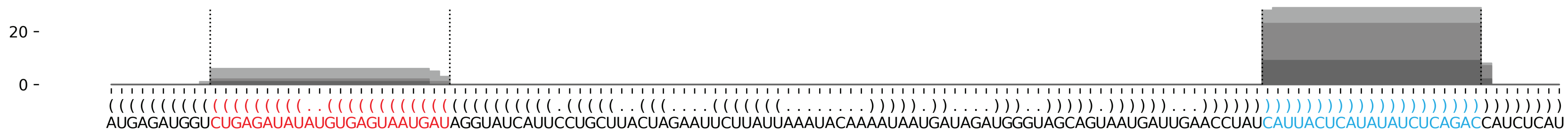

D. lacteum dla-mir-1198

```
((((((((((((((((.((.((((((((..(((..(((((..((((((....))))))...)))))..)))..))))))))..)).)))))))))))))))) -52.92 kcal/mol
AAGAAUCAAAgACAAUUUGUGACCAACACAcAAUUGAUACAAUAUAUUUCUAAAUAUACAGGUAUCUUUUGAaUGUUGGUCUUCAUGUUGUUUuUGAUUUUU count    length
Libraries combined         Precision: [ total = 99.8% | 5p-arm = 90.2% | 3p-arm = 99.9% ]
------------------------------------------------------------------------AUGUUGGUCUUCAUGUUGUUUU-------- 98216    22
------------------------------------------------------------------------AUGUUGGUCUUCAUGUUGUUUUU------- 12225    23
------------------------------------------------------------------------AUGUUGGUCUUCAUGUUGUUU--------- 12730    21
------------------------------------------------------------------------AUGUUGGUCUUCAUGUUGUU---------- 2703     20
------------------------------------------------------------------------AUGUUGGUCUUCAUGUUGU----------- 484      19
------------------------------------------------------------------------AUGUUGGUCUUCAUGUUG------------ 284      18
----------GACAAUUUGUGACCAACACA------------------------------------------------------------------------ 128      20
----------GACAAUUUGUGACCAACACAC----------------------------------------------------------------------- 84       21
-------------------------------------------------------------------------UGUUGGUCUUCAUGUUGUUUUU------- 56       22
-------------------------------------------------------------------------UGUUGGUCUUCAUGUUGUUUU-------- 58       21
----------GACAAUUUGUGACCAACAC------------------------------------------------------------------------- 52       19
---------AGACAAUUUGUGACCAACACA------------------------------------------------------------------------ 24       21
-------------------------------------------------------------------------UGUUGGUCUUCAUGUUGUUU--------- 9        20
----------GACAAUUUGUGACCAACA-------------------------------------------------------------------------- 10       18
--------------------------------------------------------------UAUCUUUUGAAUGUUGGUCU-------------------- 6        20
```

D. lacteum dla-mir-1198 continued

```
((((((((((((((.((.((((((((..(((..(((((..((((((....))))))...)))))..))).)).)))))))..)).))))))))))))))) -52.92 kcal/mol
AAGAAUCAAAgACAAUUUGUGACCAACACAcAAUUGAUACAAUAUAUUUCUAAAUAUACAGGUAUCUUUUGAaUGUUGGUCUUCAUGUUGUUUuUGAUUUUU count    length
--------------------------------------------------------------UAUCUUUUGAAUGUUGGUCUU------------------- 6        21
-----------------------------------------------------------------------AAUGUUGGUCUUCAUGUUGUUUU-------- 5        23
------------------------------------------------------------------------AUGUUGGUCUUCAUGUUGUUUUUG------ 5        24
--------------------------------------------------------------------------GUUGGUCUUCAUGUUGUUUU-------- 10       20
---------AGACAAUUUGUGACCAACACAC----------------------------------------------------------------------- 1        22
----------GACAAUUUGUGACCAACACACA---------------------------------------------------------------------- 1        22
--------------------GACCAACACACAAUUGAUACA------------------------------------------------------------- 3        21
-----------------------------------------------------------------------AAUGUUGGUCUUCAUGUUGUUU--------- 2        22
---------------------------------------------------------------------------UUGGUCUUCAUGUUGUUUU-------- 4        19
-----------------------------------------------------------------------------GGUCUUCAUGUUGUUUUU------- 1        18
---------AGACAAUUUGUGACCAACAC------------------------------------------------------------------------- 1        20
---------AGACAAUUUGUGACCAACA-------------------------------------------------------------------------- 1        19
--------------------------------------------------------------UAUCUUUUGAAUGUUGGUCUUC------------------ 1        22
--------------------------------------------------------------UAUCUUUUGAAUGUUGGUC--------------------- 1        19
-----------------------------------------------------------------------AAUGUUGGUCUUCAUGUUGUUUUU------- 2        24
------------------------------------------------------------------------AUGUUGGUCUUCAUGUUGUUUUUGAUU--- 1        27
-------------------------------------------------------------------------UGUUGGUCUUCAUGUUGUU---------- 2        19
---------------------------------------------------------------------------UUGGUCUUCAUGUUGUUUUUG------ 1        21
Library = 1       Precision: [ total = 99.9% | 5p-arm = 89.1% | 3p-arm = 99.9% ]
------------------------------------------------------------------------AUGUUGGUCUUCAUGUUGUUUU-------- 45957    22
------------------------------------------------------------------------AUGUUGGUCUUCAUGUUGUUUUU------- 6198     23
------------------------------------------------------------------------AUGUUGGUCUUCAUGUUGUUU--------- 6126     21
------------------------------------------------------------------------AUGUUGGUCUUCAUGUUGUU---------- 1191     20
------------------------------------------------------------------------AUGUUGGUCUUCAUGUUGU----------- 233      19
------------------------------------------------------------------------AUGUUGGUCUUCAUGUUG------------ 124      18
----------GACAAUUUGUGACCAACACA------------------------------------------------------------------------ 62       20
----------GACAAUUUGUGACCAACACAC----------------------------------------------------------------------- 44       21
-------------------------------------------------------------------------UGUUGGUCUUCAUGUUGUUUUU------- 26       22
-------------------------------------------------------------------------UGUUGGUCUUCAUGUUGUUUU-------- 23       21
----------GACAAUUUGUGACCAACAC------------------------------------------------------------------------- 21       19
---------AGACAAUUUGUGACCAACACA------------------------------------------------------------------------ 14       21
-------------------------------------------------------------------------UGUUGGUCUUCAUGUUGUUU--------- 7        20
----------GACAAUUUGUGACCAACA-------------------------------------------------------------------------- 3        18
--------------------------------------------------------------UAUCUUUUGAAUGUUGGUCU-------------------- 3        20
--------------------------------------------------------------UAUCUUUUGAAUGUUGGUCUU------------------- 2        21
-----------------------------------------------------------------------AAUGUUGGUCUUCAUGUUGUUUU-------- 2        23
------------------------------------------------------------------------AUGUUGGUCUUCAUGUUGUUUUUG------ 2        24
--------------------------------------------------------------------------GUUGGUCUUCAUGUUGUUUU-------- 2        20
---------AGACAAUUUGUGACCAACACAC----------------------------------------------------------------------- 1        22
----------GACAAUUUGUGACCAACACACA---------------------------------------------------------------------- 1        22
--------------------GACCAACACACAAUUGAUACA------------------------------------------------------------- 1        21
-----------------------------------------------------------------------AAUGUUGGUCUUCAUGUUGUUU--------- 1        22
---------------------------------------------------------------------------UUGGUCUUCAUGUUGUUUU-------- 1        19
-----------------------------------------------------------------------------GGUCUUCAUGUUGUUUUU------- 1        18
Library = 2       Precision: [ total = 99.8% | 5p-arm = 89.4% | 3p-arm = 99.9% ]
------------------------------------------------------------------------AUGUUGGUCUUCAUGUUGUUUU-------- 28380    22
------------------------------------------------------------------------AUGUUGGUCUUCAUGUUGUUU--------- 3721     21
------------------------------------------------------------------------AUGUUGGUCUUCAUGUUGUUUUU------- 3480     23
------------------------------------------------------------------------AUGUUGGUCUUCAUGUUGUU---------- 802      20
------------------------------------------------------------------------AUGUUGGUCUUCAUGUUGU----------- 130      19
------------------------------------------------------------------------AUGUUGGUCUUCAUGUUG------------ 86       18
----------GACAAUUUGUGACCAACACA------------------------------------------------------------------------ 34       20
----------GACAAUUUGUGACCAACACAC----------------------------------------------------------------------- 22       21
-------------------------------------------------------------------------UGUUGGUCUUCAUGUUGUUUU-------- 19       21
```

D. lacteum dla-mir-1198 continued

```
((((((((((((((((.((.((((((((..(((..(((((..((((((....))))))...)))))..)))..))))))))..)).)))))))))))))))) -52.92 kcal/mol
AAGAAUCAAAgACAAUUUGUGACCAACACAcAAUUGAUACAAUAUAUUUCUAAAUAUACAGGUAUCUUUUGAaUGUUGGUCUUCAUGUUGUUUuUGAUUUUU count    length
-------------------------------------------------------------------------UGUUGGUCUUCAUGUUGUUUUU------- 18       22
----------GACAAUUUGUGACCAACAC------------------------------------------------------------------------- 15       19
---------AGACAAUUUGUGACCAACACA------------------------------------------------------------------------ 7        21
----------GACAAUUUGUGACCAACA-------------------------------------------------------------------------- 5        18
--------------------------------------------------------------UAUCUUUUGAAUGUUGGUCUU------------------- 4        21
--------------------------------------------------------------------------GUUGGUCUUCAUGUUGUUUU-------- 4        20
------------------------------------------------------------------------AUGUUGGUCUUCAUGUUGUUUUUG------ 3        24
-----------------------------------------------------------------------AAUGUUGGUCUUCAUGUUGUUUUU------- 2        24
---------AGACAAUUUGUGACCAACAC------------------------------------------------------------------------- 1        20
--------------------GACCAACACACAAUUGAUACA------------------------------------------------------------- 1        21
--------------------------------------------------------------UAUCUUUUGAAUGUUGGUCU-------------------- 1        20
--------------------------------------------------------------UAUCUUUUGAAUGUUGGUCUUC------------------ 1        22
--------------------------------------------------------------UAUCUUUUGAAUGUUGGUC--------------------- 1        19
-----------------------------------------------------------------------AAUGUUGGUCUUCAUGUUGUUUU-------- 1        23
-------------------------------------------------------------------------UGUUGGUCUUCAUGUUGUUU--------- 1        20
---------------------------------------------------------------------------UUGGUCUUCAUGUUGUUUU-------- 1        19
Library = 3        Precision: [ total = 99.8% | 5p-arm = 93.2% | 3p-arm = 99.9% ]
------------------------------------------------------------------------AUGUUGGUCUUCAUGUUGUUUU-------- 23879    22
------------------------------------------------------------------------AUGUUGGUCUUCAUGUUGUUU--------- 2883     21
------------------------------------------------------------------------AUGUUGGUCUUCAUGUUGUUUUU------- 2547     23
------------------------------------------------------------------------AUGUUGGUCUUCAUGUUGUU---------- 710      20
------------------------------------------------------------------------AUGUUGGUCUUCAUGUUGU----------- 121      19
------------------------------------------------------------------------AUGUUGGUCUUCAUGUUG------------ 74       18
----------GACAAUUUGUGACCAACACA------------------------------------------------------------------------ 32       20
----------GACAAUUUGUGACCAACACAC----------------------------------------------------------------------- 18       21
----------GACAAUUUGUGACCAACAC------------------------------------------------------------------------- 16       19
-------------------------------------------------------------------------UGUUGGUCUUCAUGUUGUUUU-------- 16       21
-------------------------------------------------------------------------UGUUGGUCUUCAUGUUGUUUUU------- 12       22
--------------------------------------------------------------------------GUUGGUCUUCAUGUUGUUUU-------- 4        20
---------AGACAAUUUGUGACCAACACA------------------------------------------------------------------------ 3        21
----------GACAAUUUGUGACCAACA-------------------------------------------------------------------------- 2        18
--------------------------------------------------------------UAUCUUUUGAAUGUUGGUCU-------------------- 2        20
-----------------------------------------------------------------------AAUGUUGGUCUUCAUGUUGUUUU-------- 2        23
-------------------------------------------------------------------------UGUUGGUCUUCAUGUUGUU---------- 2        19
---------------------------------------------------------------------------UUGGUCUUCAUGUUGUUUU-------- 2        19
---------AGACAAUUUGUGACCAACA-------------------------------------------------------------------------- 1        19
--------------------GACCAACACACAAUUGAUACA------------------------------------------------------------- 1        21
-----------------------------------------------------------------------AAUGUUGGUCUUCAUGUUGUUU--------- 1        22
------------------------------------------------------------------------AUGUUGGUCUUCAUGUUGUUUUUGAUU--- 1        27
-------------------------------------------------------------------------UGUUGGUCUUCAUGUUGUUU--------- 1        20
---------------------------------------------------------------------------UUGGUCUUCAUGUUGUUUUUG------ 1        21
```

```
                U  U         CA    UU     AA       U
AAGAAUCAAAgACAAU UG -GACCAACA  cAA  GAUAC  -UAUAUU C
UUUUUAGUuUUUGUUG AC  CUGGUUGU  GUU  CUAUG   AUAUAA U
                U  UU        aA    UU     GAC      A
```

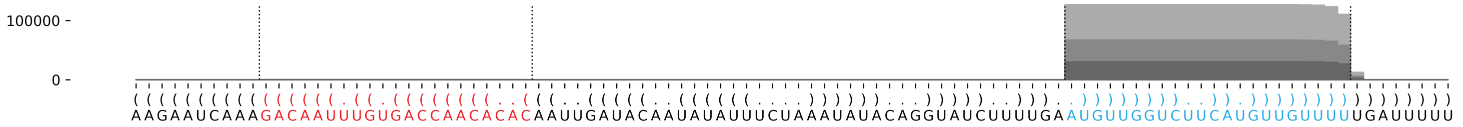

P. pallidum ppa-mir-1199

```
.......((((((((((((((((((((((((((((((((((((((((.........)))))))))))))))))))))))))))))))))))))))).......   -65.05 kcal/mol
UAAUUUUUUGaACAGACAUCUUGAAAAAAUAaAGUUAAAUUAAAGUUCAAAAAAUUAACUUUAAUUUAACUUUaUUUUUUCAAGAUGUCUGUUCaACUUAGGU   count   length
Libraries combined         Precision: [ total = 83.6% | 5p-arm = 72.6% | 3p-arm = 84.5% ]
-------------------------------------------------------------------------AUUUUUUCAAGAUGUCUGUUC---------   2199   21
----------AACAGACAUCUUGAAAAAAUA------------------------------------------------------------------------   153    21
--------------------------------------------------------------------------UUUUUUCAAGAUGUCUGUUCA--------   176    21
-------------------------------------------------------------------------AUUUUUUCAAGAUGUCUGUU----------   145    20
---------------------------------------------------------------------------------AAGAUGUCUGUUCAACUUAGG-   61     21
----------------------------------------------------------------------------------AGAUGUCUGUUCAACUUAGGU   65     21
--------------------------------------------------------------------------UUUUUUCAAGAUGUCUGUUC---------   63     20
--------UGAACAGACAUCUUGAAAAAA--------------------------------------------------------------------------   25     21
----------AACAGACAUCUUGAAAAAAU-------------------------------------------------------------------------   10     20
-------------------------------------------------------------------------AUUUUUUCAAGAUGUCUGUUCA--------   18     22
-------UUGAACAGACAUCUUGAAAAA---------------------------------------------------------------------------   9      21
-------------------------------------------------------------------AACUUUAUUUUUUCAAGAUGUC--------------   7      22
----------------------------------------------------------------------------------AGAUGUCUGUUCAACUUAGG-   4      20
-----------ACAGACAUCUUGAAAAAAUA------------------------------------------------------------------------   8      20
------------------------------------------------------------------UAACUUUAUUUUUUCAAGAUG----------------   3      21
----------------------------------------------------------------------UUUAUUUUUUCAAGAUGUCUGUU----------   5      23
------------------------------------------------------------------------UAUUUUUUCAAGAUGUCUGUUC---------   11     22
-------------------------------------------------------------------------AUUUUUUCAAGAUGUCUGU-----------   5      19
-----------------------------------------------------------------------------UUUCAAGAUGUCUGUUCAACU-----   4      21
-----------ACAGACAUCUUGAAAAAAUAA-----------------------------------------------------------------------   5      21
--------------------------------------------------------------------------UUUUUUCAAGAUGUCUGUU----------   9      19
-------UUGAACAGACAUCUUGAAAAAAU-------------------------------------------------------------------------   1      23
--------UGAACAGACAUCUUGAAAAA---------------------------------------------------------------------------   5      20
-------------------------------------------------------------------AACUUUAUUUUUUCAAGAUGUCUGUU----------   2      26
-------------------------------------------------------------------------AUUUUUUCAAGAUGUCUGUUCAA-------   1      23
---------------------------------------------------------------------------------AAGAUGUCUGUUCAACUUAGGU   3      22
-------UUGAACAGACAUCUUGAAAAAA--------------------------------------------------------------------------   3      22
--------UGAACAGACAUCUUGAAAAAAU-------------------------------------------------------------------------   2      22
---------GAACAGACAUCUUGAAAAAAUA------------------------------------------------------------------------   1      22
----------AACAGACAUCUUGAAAAAAUAA-----------------------------------------------------------------------   1      22
-----------ACAGACAUCUUGAAAAAA--------------------------------------------------------------------------   1      18
-----------ACAGACAUCUUGAAAAAAUAAA----------------------------------------------------------------------   1      22
-----------ACAGACAUCUUGAAAAAAU-------------------------------------------------------------------------   1      19
------------------------------------------------------------------UAACUUUAUUUUUUCAAGAUGU---------------   1      22
-------------------------------------------------------------------AACUUUAUUUUUUCAAGAUGUCUGU-----------   2      25
-------------------------------------------------------------------AACUUUAUUUUUUCAAGAUGUCUG------------   3      24
----------------------------------------------------------------------UUUAUUUUUUCAAGAUGUCUGU-----------   1      22
-----------------------------------------------------------------------UUAUUUUUUCAAGAUGUCUGU-----------   1      21
-----------------------------------------------------------------------UUAUUUUUUCAAGAUGUCUGUU----------   2      22
------------------------------------------------------------------------UAUUUUUUCAAGAUGUCUGUU----------   6      21
------------------------------------------------------------------------UAUUUUUUCAAGAUGUCUGUUCA--------   1      23
-------------------------------------------------------------------------AUUUUUUCAAGAUGUCUG------------   2      18
--------------------------------------------------------------------------UUUUUUCAAGAUGUCUGUUCAA-------   1      22
---------------------------------------------------------------------------UUUUUCAAGAUGUCUGUUCAA-------   1      21
-------------------------------------------------------------------------------UCAAGAUGUCUGUUCAACUUAGGU   1      24
--------------------------------------------------------------------------------CAAGAUGUCUGUUCAACUUAGG-   1      22
---------------------------------------------------------------------------------AAGAUGUCUGUUCAACUUAG--   1      20
Library = 1        Precision: [ total = 85.2% | 5p-arm = 77.9% | 3p-arm = 85.8% ]
-------------------------------------------------------------------------AUUUUUUCAAGAUGUCUGUUC---------   1005   21
----------AACAGACAUCUUGAAAAAAUA------------------------------------------------------------------------   74     21
--------------------------------------------------------------------------UUUUUUCAAGAUGUCUGUUCA--------   63     21
-------------------------------------------------------------------------AUUUUUUCAAGAUGUCUGUU----------   57     20
---------------------------------------------------------------------------------AAGAUGUCUGUUCAACUUAGG-   35     21
```

P. pallidum ppa-mir-1199 continued

```
.......((((((((((((((((((((((((((((((((((((((((.........)))))))))))))))))))))))))))))))))))))))).......          -65.05 kcal/mol
UAAUUUUUUGaACAGACAUCUUGAAAAAAUAaAGUUAAAUUAAAGUUCAAAAAAUUAACUUUAAUUUAACUUUaUUUUUUCAAGAUGUCUGUUCaACUUAGGU          count    length
----------------------------------------------------------------------------------AGAUGUCUGUUCAACUUAGGU          29       21
--------------------------------------------------------------------------UUUUUUCAAGAUGUCUGUUC---------          27       20
--------UGAACAGACAUCUUGAAAAAA--------------------------------------------------------------------------          11       21
----------AACAGACAUCUUGAAAAAAU-------------------------------------------------------------------------          7        20
-------------------------------------------------------------------------AUUUUUUCAAGAUGUCUGUUCA--------          6        22
-------UUGAACAGACAUCUUGAAAAA---------------------------------------------------------------------------          5        21
-------------------------------------------------------------------AACUUUAUUUUUUCAAGAUGUC--------------          4        22
----------------------------------------------------------------------------------AGAUGUCUGUUCAACUUAGG-          4        20
-----------ACAGACAUCUUGAAAAAAUA------------------------------------------------------------------------          3        20
------------------------------------------------------------------UAACUUUAUUUUUUCAAGAUG----------------          3        21
----------------------------------------------------------------------UUUAUUUUUUCAAGAUGUCUGUU----------          3        23
------------------------------------------------------------------------UAUUUUUUCAAGAUGUCUGUUC---------          3        22
-------------------------------------------------------------------------AUUUUUUCAAGAUGUCUGU-----------          3        19
-----------------------------------------------------------------------------UUUCAAGAUGUCUGUUCAACU-----          3        21
-----------ACAGACAUCUUGAAAAAAUAA-----------------------------------------------------------------------          2        21
--------------------------------------------------------------------------UUUUUUCAAGAUGUCUGUU----------          2        19
-------UUGAACAGACAUCUUGAAAAAAU-------------------------------------------------------------------------          1        23
--------UGAACAGACAUCUUGAAAAA---------------------------------------------------------------------------          1        20
-------------------------------------------------------------------AACUUUAUUUUUUCAAGAUGUCUGUU----------          1        26
-------------------------------------------------------------------------AUUUUUUCAAGAUGUCUGUUCAA-------          1        23
---------------------------------------------------------------------------------AAGAUGUCUGUUCAACUUAGGU          1        22
Library = 2       Precision: [ total = 78.7% | 5p-arm = 63.4% | 3p-arm = 80.5% ]
-------------------------------------------------------------------------AUUUUUUCAAGAUGUCUGUUC---------          526      21
--------------------------------------------------------------------------UUUUUUCAAGAUGUCUGUUCA--------          73       21
----------AACAGACAUCUUGAAAAAAUA------------------------------------------------------------------------          49       21
-------------------------------------------------------------------------AUUUUUUCAAGAUGUCUGUU----------          36       20
---------------------------------------------------------------------------------AAGAUGUCUGUUCAACUUAGG-          17       21
--------UGAACAGACAUCUUGAAAAAA--------------------------------------------------------------------------          11       21
-------------------------------------------------------------------------AUUUUUUCAAGAUGUCUGUUCA--------          10       22
--------------------------------------------------------------------------UUUUUUCAAGAUGUCUGUUC---------          9        20
----------------------------------------------------------------------------------AGAUGUCUGUUCAACUUAGGU          7        21
------------------------------------------------------------------------UAUUUUUUCAAGAUGUCUGUUC---------          5        22
------------------------------------------------------------------------UAUUUUUUCAAGAUGUCUGUU----------          5        21
-----------ACAGACAUCUUGAAAAAAUA------------------------------------------------------------------------          4        20
--------------------------------------------------------------------------UUUUUUCAAGAUGUCUGUU----------          4        19
-------UUGAACAGACAUCUUGAAAAAA--------------------------------------------------------------------------          3        22
--------UGAACAGACAUCUUGAAAAA---------------------------------------------------------------------------          3        20
-----------ACAGACAUCUUGAAAAAAUAA-----------------------------------------------------------------------          3        21
--------UGAACAGACAUCUUGAAAAAAU-------------------------------------------------------------------------          2        22
----------AACAGACAUCUUGAAAAAAU-------------------------------------------------------------------------          2        20
-------------------------------------------------------------------AACUUUAUUUUUUCAAGAUGUCUGU-----------          2        25
-------------------------------------------------------------------AACUUUAUUUUUUCAAGAUGUCUG------------          2        24
----------------------------------------------------------------------UUUAUUUUUUCAAGAUGUCUGUU----------          2        23
-----------------------------------------------------------------------UUAUUUUUUCAAGAUGUCUGUU----------          2        22
-------------------------------------------------------------------------AUUUUUUCAAGAUGUCUG------------          2        18
-------UUGAACAGACAUCUUGAAAAA---------------------------------------------------------------------------          1        21
----------AACAGACAUCUUGAAAAAAUAA-----------------------------------------------------------------------          1        22
-----------ACAGACAUCUUGAAAAAA--------------------------------------------------------------------------          1        18
-----------ACAGACAUCUUGAAAAAAUAAA----------------------------------------------------------------------          1        22
-----------ACAGACAUCUUGAAAAAAU-------------------------------------------------------------------------          1        19
------------------------------------------------------------------UAACUUUAUUUUUUCAAGAUGU---------------          1        22
-------------------------------------------------------------------AACUUUAUUUUUUCAAGAUGUC--------------          1        22
-------------------------------------------------------------------AACUUUAUUUUUUCAAGAUGUCUGUU----------          1        26
-----------------------------------------------------------------------UUAUUUUUUCAAGAUGUCUGU-----------          1        21
```

P. pallidum ppa-mir-1199 continued

```
.......((((((((((((((((((((((((((((((((((((((((.........)))))))))))))))))))))))))))))))))))))))).......       -65.05 kcal/mol
UAAUUUUUUGaACAGACAUCUUGAAAAAAUAaAGUUAAAUUAAAGUUCAAAAAAUUAACUUUAAUUUAACUUUaUUUUUUCAAGAUGUCUGUUCaACUUAGGU       count    length
------------------------------------------------------------------------UAUUUUUUCAAGAUGUCUGUUCA--------       1        23
---------------------------------------------------------------------------UUUUUCAAGAUGUCUGUUCAA-------       1        21
-----------------------------------------------------------------------------UUUCAAGAUGUCUGUUCAACU-----       1        21
-------------------------------------------------------------------------------UCAAGAUGUCUGUUCAACUUAGGU       1        24
--------------------------------------------------------------------------------CAAGAUGUCUGUUCAACUUAGG-       1        22
---------------------------------------------------------------------------------AAGAUGUCUGUUCAACUUAGGU       1        22
---------------------------------------------------------------------------------AAGAUGUCUGUUCAACUUAG--       1        20
Library = 3        Precision: [ total = 85.6% | 5p-arm = 77.5% | 3p-arm = 86.0% ]
-------------------------------------------------------------------------AUUUUUUCAAGAUGUCUGUUC---------       668      21
-------------------------------------------------------------------------AUUUUUUCAAGAUGUCUGUU----------       52       20
--------------------------------------------------------------------------UUUUUUCAAGAUGUCUGUUCA--------       40       21
----------AACAGACAUCUUGAAAAAAUA------------------------------------------------------------------------       30       21
----------------------------------------------------------------------------------AGAUGUCUGUUCAACUUAGGU       29       21
--------------------------------------------------------------------------UUUUUUCAAGAUGUCUGUUC---------       27       20
---------------------------------------------------------------------------------AAGAUGUCUGUUCAACUUAGG-       9        21
-------UUGAACAGACAUCUUGAAAAA---------------------------------------------------------------------------       3        21
--------UGAACAGACAUCUUGAAAAAA--------------------------------------------------------------------------       3        21
------------------------------------------------------------------------UAUUUUUUCAAGAUGUCUGUUC---------       3        22
--------------------------------------------------------------------------UUUUUUCAAGAUGUCUGUU----------       3        19
-------------------------------------------------------------------AACUUUAUUUUUUCAAGAUGUC--------------       2        22
-------------------------------------------------------------------------AUUUUUUCAAGAUGUCUGUUCA--------       2        22
-------------------------------------------------------------------------AUUUUUUCAAGAUGUCUGU-----------       2        19
--------UGAACAGACAUCUUGAAAAA---------------------------------------------------------------------------       1        20
---------GAACAGACAUCUUGAAAAAAUA------------------------------------------------------------------------       1        22
----------AACAGACAUCUUGAAAAAAU-------------------------------------------------------------------------       1        20
-----------ACAGACAUCUUGAAAAAAUA------------------------------------------------------------------------       1        20
-------------------------------------------------------------------AACUUUAUUUUUUCAAGAUGUCUG------------       1        24
----------------------------------------------------------------------UUUAUUUUUUCAAGAUGUCUGU-----------       1        22
------------------------------------------------------------------------UAUUUUUUCAAGAUGUCUGUU----------       1        21
--------------------------------------------------------------------------UUUUUUCAAGAUGUCUGUUCAA-------       1        22
---------------------------------------------------------------------------------AAGAUGUCUGUUCAACUUAGGU       1        22
```

```
UAAUUUU                                       CAAA
       UUGaACAGACAUCUUGAAAAAAUAaAGUUAAAUUAAAGUU    A
       AaCUUGUCUGUAGAACUUUUUUaUUUCAAUUUAAUUUCAA    /
UGGAUUC                                       UUAA
```

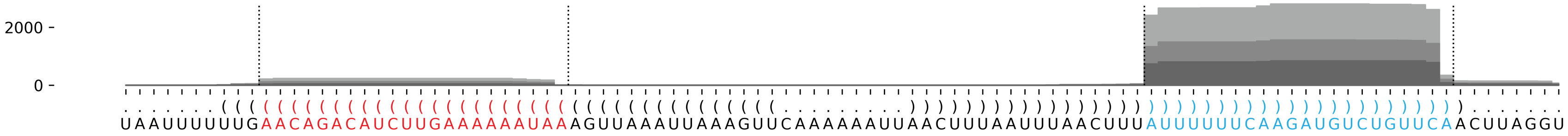

P. palliddum ppa-mir-1200

```
.....(((((((.((((((((((((.(((((.((....(((((.((((((.....)))))).)))))...)).))))).)))))))))))).))))))).....   -35.21 kcal/mol
CUAAUUAAUUuGCUAAAAUCAAAAAUAGACaAAUUCUAACUAAGUAUUUUUAUUUAAAAUAAUUAGUAUUAUCUGuCUCUUUUUGGUUUUAAUAAaUUAUUAUU   count   length
Libraries combined         Precision: [ total = 100.0% | 5p-arm = 100.0% | 3p-arm = 100.0% ]
----------UGCUAAAAUCAAAAAUAGACA-------------------------------------------------------------------------   21      21
----------UGCUAAAAUCAAAAAUAGAC--------------------------------------------------------------------------   22      20
---------------------------------------------------------------------------UCUCUUUUUGGUUUUAAUAAA--------   5       21
Library = 1        Precision: [ total = 100.0% | 5p-arm = 100.0% | 3p-arm = 100.0% ]
----------UGCUAAAAUCAAAAAUAGACA-------------------------------------------------------------------------   15      21
----------UGCUAAAAUCAAAAAUAGAC--------------------------------------------------------------------------   11      20
---------------------------------------------------------------------------UCUCUUUUUGGUUUUAAUAAA--------   4       21
Library = 2        Precision: [ total = 100.0% | 5p-arm = 100.0% | 3p-arm = 0% ]
----------UGCUAAAAUCAAAAAUAGACA-------------------------------------------------------------------------   4       21
----------UGCUAAAAUCAAAAAUAGAC--------------------------------------------------------------------------   3       20
Library = 3        Precision: [ total = 100.0% | 5p-arm = 100.0% | 3p-arm = 100.0% ]
----------UGCUAAAAUCAAAAAUAGAC--------------------------------------------------------------------------   8       20
----------UGCUAAAAUCAAAAAUAGACA-------------------------------------------------------------------------   2       21
---------------------------------------------------------------------------UCUCUUUUUGGUUUUAAUAAA--------   1       21
```

```
CUAAU        C            U     A  UCUA     G      UA
     UAAUUuG UAAAAUCAAAAA AGACa AU    ACUAA UAUUUU  U
     AUUaAAU AUUUUGGUUUUU UCuGU UA   -UGAUU AUAAAA  /
UUAUU        A            C     C  UUA      A      UU
```

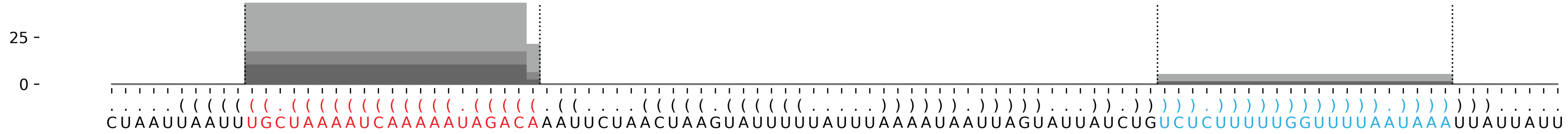

P. pallidum ppa-mir-1201

```
((.((((((((((.((((((.((((((((((((((((..((...))..))))).(((((...............))))).))))))))))).))))))).)))))))))).))      -54.86 kcal/mol
ACUCGGAUUAuCUUACACUUUCUCAUUCUAuCCGAAGCCAGUUCAACAAGCUUUGAGAUACCAAGAAUACAGAUCAUGUCUAGGAUaGAAUGGUAAAGUGUCAGAUaAUCUGUGU      count    length
Libraries combined          Precision: [ total = 99.1% | 5p-arm = 100.0% | 3p-arm = 99.0% ]
--------------------------------------------------------------------------------------AGAAUGGUAAAGUGUCAGAUA--------      479      21
--------------------------------------------------------------------------------------AGAAUGGUAAAGUGUCAGAU---------      35       20
--------------------------------------------------------------------------------------AGAAUGGUAAAGUGUCAGAUAAU------      2        23
----------UCUUACACUUUCUCAUUCUAU------------------------------------------------------------------------------------      4        21
--------------------------------------------------------------------------------------AGAAUGGUAAAGUGUCAGAUAA-------      1        22
-----------------------------------------------------------------------------------------AUGGUAAAGUGUCAGAUAAUC-----      2        21
------------------------------------------------------------------AUACAGAUCAUGUCUAGGAUA----------------------------      1        21
--------------------------------------------------------------------------------------AGAAUGGUAAAGUGUCAGA----------      2        19
---------------------------------------------------------------------------------------GAAUGGUAAAGUGUCAGAUA--------      1        20
-----------------------------------------------------------------------------------------AUGGUAAAGUGUCAGAUA--------      1        18
Library = 1         Precision: [ total = 99.6% | 5p-arm = 100.0% | 3p-arm = 99.6% ]
--------------------------------------------------------------------------------------AGAAUGGUAAAGUGUCAGAUA--------      247      21
--------------------------------------------------------------------------------------AGAAUGGUAAAGUGUCAGAU---------      21       20
--------------------------------------------------------------------------------------AGAAUGGUAAAGUGUCAGAUAAU------      2        23
----------UCUUACACUUUCUCAUUCUAU------------------------------------------------------------------------------------      1        21
--------------------------------------------------------------------------------------AGAAUGGUAAAGUGUCAGAUAA-------      1        22
-----------------------------------------------------------------------------------------AUGGUAAAGUGUCAGAUAAUC-----      1        21
Library = 2         Precision: [ total = 98.4% | 5p-arm = 100.0% | 3p-arm = 98.4% ]
--------------------------------------------------------------------------------------AGAAUGGUAAAGUGUCAGAUA--------      115      21
--------------------------------------------------------------------------------------AGAAUGGUAAAGUGUCAGAU---------      9        20
----------UCUUACACUUUCUCAUUCUAU------------------------------------------------------------------------------------      1        21
--------------------------------------------------------------------------------------AGAAUGGUAAAGUGUCAGA----------      1        19
-----------------------------------------------------------------------------------------AUGGUAAAGUGUCAGAUAAUC-----      1        21
-----------------------------------------------------------------------------------------AUGGUAAAGUGUCAGAUA--------      1        18
Library = 3         Precision: [ total = 98.4% | 5p-arm = 100.0% | 3p-arm = 98.4% ]
--------------------------------------------------------------------------------------AGAAUGGUAAAGUGUCAGAUA--------      117      21
--------------------------------------------------------------------------------------AGAAUGGUAAAGUGUCAGAU---------      5        20
----------UCUUACACUUUCUCAUUCUAU------------------------------------------------------------------------------------      2        21
------------------------------------------------------------------AUACAGAUCAUGUCUAGGAUA----------------------------      1        21
--------------------------------------------------------------------------------------AGAAUGGUAAAGUGUCAGA----------      1        19
---------------------------------------------------------------------------------------GAAUGGUAAAGUGUCAGAUA--------      1        20
```

```
                                C-
                               U  A
                                UA
                                GC
                               A  A
                               C  A
                                CG
                                GC
                                AU
                                AU
  U          U       C          GUG     CCAAGAA
AC CGGAUUAuCU ACACUUU UCAUUCUAuCC   AGAUA       U
UG GUCUAaUAGA UGUGAAA GGUAAGaUAGG --UCUGU       A
  U          C       U           A      ACUAGAC
```

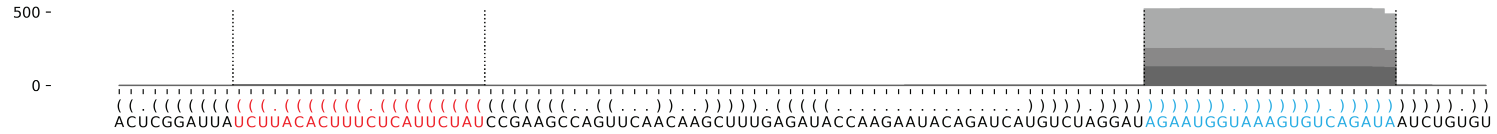

P. pallidum ppa-mir-1202

```
...(((((((.((.((((((((((.((((((.((((((((((.(((.((..((((((.((...(((.((((.(((((.....)))))..))))...))).))))))))..)).))).)..((((((((...((((..(((...)))..))))....))))))))))))))))).)))))).))))))))))).)).))))))))...   -101.16 kcal/mol
CCAUCGGAUGaUGCUUAACAGAUUUAAUUUcAAUUGGUACCAAGCUGACAUGUCUCUUUGUUUUGAAAGGGUAUCUUUAGACCAAAGACCACCCCUCUUCUACAAGAGAAUUGACAGGUCUGAUGGCUAUCUACAGAAUAGUCGCUAAGCUGUCUCUUAGUCGUCUGGUGUCAAGUGAaAUAGAAUCUGUUGUGCUUCaUCCGAGUA   count   length
Libraries combined      Precision: [ total = 99.2% | 5p-arm = 99.3% | 3p-arm = 99.2% ]
----------------------------------------------------------------------------------------------------------------------------------------------------------------------------------AAUAGAAUCUGUUGUGCUUCA--------   1794   21
----------------------------------------------------------------------------------------------------------------------------------------------------------------------------------AAUAGAAUCUGUUGUGCUUC---------   868   20
----------AUGCUUAACAGAUUUAAUUUC--------------------------------------------------------------------------------------------------------------------------------------------------------------------------------   534   21
----------------------------------------------------------------------------------------------------------------------------------------------------------------------------------AAUAGAAUCUGUUGUGCUUCAU-------   281   22
----------------------------------------------------------------------------------------------------------------------------------------------------------------------------------AAUAGAAUCUGUUGUGCUU----------   79   19
----------------------------------------------------------------------------------------------------------------------------------------------------------------------------------AAUAGAAUCUGUUGUGCU-----------   13   18
------------------------------------------------------------------------------------------------------------------------------------------------------UGUCUCUUAGUCGUCUGGUGUCAAGUGA-----------------------------   4   28
-------------------------------------------------------CUUUGUUUUGAAAGGGUAUCU-----------------------------------------------------------------------------------------------------------------------------------   2   21
----------------------------------------------------------------------------------------------------------------------------------------------------------------------------------AAUAGAAUCUGUUGUGCUUCAUC------   8   23
-------------------------------------UACCAAGCUGACAUGUCUCUU-----------------------------------------------------------------------------------------------------------------------------------------------------   2   21
-------------------------------------------------------------------------------------------------------AAGAGAAUUGACAGGUCUGAUG----------------------------------------------------------------------------------   1   22
-------------------------------------------------------------------------------------------------------AAGAGAAUUGACAGGUCUGAU-----------------------------------------------------------------------------------   3   21
-------------------------------------------------------------------------------------------------------------------------------------------------UAAGCUGUCUCUUAGUCGUC------------------------------------------   4   20
----------------------------------------------------------------------------------------------------------------------------------------------------------------------------------AAUAGAAUCUGUUGUGCUUCAUCCGAGUA   1   29
-----------------------------------------------------------------------------------------------------------------------------------------------------------------------------------AUAGAAUCUGUUGUGCUUCA--------   1   20
---------------------------------------------------------------------------------------------------CUACAAGAGAAUUGACAGGUC---------------------------------------------------------------------------------------   1   21
------------------------------------------------------------------------------------------------------CAAGAGAAUUGACAGGUCUGA------------------------------------------------------------------------------------   4   21
-------------------------------------------------------------------------------------------------------------------------------------------------UAAGCUGUCUCUUAGUCGU-------------------------------------------   2   19
-------------------------------------------------------------------------------------------------------------------------------------------------UAAGCUGUCUCUUAGUCGUCU-----------------------------------------   3   21
-------------------------------------------------------------------------------------------------------------------------------------------------------GUCUCUUAGUCGUCUGGUGUCAAGUGA-----------------------------   1   27
------------------------------------------------------------------------------------------------------------------------------------------------------------------------------------UAGAAUCUGUUGUGCUUCAU-------   1   20
Library = 1 Precision: [ total = 98.9% | 5p-arm = 98.0% | 3p-arm = 99.1% ]
----------------------------------------------------------------------------------------------------------------------------------------------------------------------------------AAUAGAAUCUGUUGUGCUUCA--------   484   21
----------------------------------------------------------------------------------------------------------------------------------------------------------------------------------AAUAGAAUCUGUUGUGCUUC---------   248   20
----------AUGCUUAACAGAUUUAAUUUC--------------------------------------------------------------------------------------------------------------------------------------------------------------------------------   145   21
----------------------------------------------------------------------------------------------------------------------------------------------------------------------------------AAUAGAAUCUGUUGUGCUUCAU-------   102   22
----------------------------------------------------------------------------------------------------------------------------------------------------------------------------------AAUAGAAUCUGUUGUGCUU----------   22   19
----------------------------------------------------------------------------------------------------------------------------------------------------------------------------------AAUAGAAUCUGUUGUGCU-----------   5   18
------------------------------------------------------------------------------------------------------------------------------------------------------UGUCUCUUAGUCGUCUGGUGUCAAGUGA-----------------------------   3   28
-------------------------------------------------------CUUUGUUUUGAAAGGGUAUCU-----------------------------------------------------------------------------------------------------------------------------------   2   21
----------------------------------------------------------------------------------------------------------------------------------------------------------------------------------AAUAGAAUCUGUUGUGCUUCAUC------   2   23
-------------------------------------UACCAAGCUGACAUGUCUCUU-----------------------------------------------------------------------------------------------------------------------------------------------------   1   21
-------------------------------------------------------------------------------------------------------AAGAGAAUUGACAGGUCUGAUG----------------------------------------------------------------------------------   1   22
-------------------------------------------------------------------------------------------------------AAGAGAAUUGACAGGUCUGAU-----------------------------------------------------------------------------------   1   21
-------------------------------------------------------------------------------------------------------------------------------------------------UAAGCUGUCUCUUAGUCGUC------------------------------------------   1   20
----------------------------------------------------------------------------------------------------------------------------------------------------------------------------------AAUAGAAUCUGUUGUGCUUCAUCCGAGUA   1   29
-----------------------------------------------------------------------------------------------------------------------------------------------------------------------------------AUAGAAUCUGUUGUGCUUCA--------   1   20
Library = 2 Precision: [ total = 99.1% | 5p-arm = 99.6% | 3p-arm = 99.0% ]
----------------------------------------------------------------------------------------------------------------------------------------------------------------------------------AAUAGAAUCUGUUGUGCUUCA--------   733   21
----------------------------------------------------------------------------------------------------------------------------------------------------------------------------------AAUAGAAUCUGUUGUGCUUC---------   351   20
----------AUGCUUAACAGAUUUAAUUUC--------------------------------------------------------------------------------------------------------------------------------------------------------------------------------   268   21
----------------------------------------------------------------------------------------------------------------------------------------------------------------------------------AAUAGAAUCUGUUGUGCUUCAU-------   101   22
----------------------------------------------------------------------------------------------------------------------------------------------------------------------------------AAUAGAAUCUGUUGUGCUU----------   38   19
----------------------------------------------------------------------------------------------------------------------------------------------------------------------------------AAUAGAAUCUGUUGUGCU-----------   5   18
----------------------------------------------------------------------------------------------------------------------------------------------------------------------------------AAUAGAAUCUGUUGUGCUUCAUC------   4   23
-------------------------------------------------------------------------------------------------------------------------------------------------UAAGCUGUCUCUUAGUCGUC------------------------------------------   3   20
-------------------------------------------------------------------------------------------------------AAGAGAAUUGACAGGUCUGAU-----------------------------------------------------------------------------------   2   21
-------------------------------------------------------------------------------------------------------------------------------------------------UAAGCUGUCUCUUAGUCGU-------------------------------------------   2   19
-------------------------------------------------------------------------------------------------------------------------------------------------UAAGCUGUCUCUUAGUCGUCU-----------------------------------------   2   21
-------------------------------------UACCAAGCUGACAUGUCUCUU-----------------------------------------------------------------------------------------------------------------------------------------------------   1   21
---------------------------------------------------------------------------------------------------CUACAAGAGAAUUGACAGGUC---------------------------------------------------------------------------------------   1   21
------------------------------------------------------------------------------------------------------CAAGAGAAUUGACAGGUCUGA------------------------------------------------------------------------------------   1   21
------------------------------------------------------------------------------------------------------------------------------------------------------UGUCUCUUAGUCGUCUGGUGUCAAGUGA-----------------------------   1   28
-------------------------------------------------------------------------------------------------------------------------------------------------------GUCUCUUAGUCGUCUGGUGUCAAGUGA-----------------------------   1   27
Library = 3 Precision: [ total = 99.5% | 5p-arm = 100.0% | 3p-arm = 99.5% ]
----------------------------------------------------------------------------------------------------------------------------------------------------------------------------------AAUAGAAUCUGUUGUGCUUCA--------   577   21
----------------------------------------------------------------------------------------------------------------------------------------------------------------------------------AAUAGAAUCUGUUGUGCUUC---------   269   20
----------AUGCUUAACAGAUUUAAUUUC--------------------------------------------------------------------------------------------------------------------------------------------------------------------------------   121   21
----------------------------------------------------------------------------------------------------------------------------------------------------------------------------------AAUAGAAUCUGUUGUGCUUCAU-------   78   22
----------------------------------------------------------------------------------------------------------------------------------------------------------------------------------AAUAGAAUCUGUUGUGCUU----------   19   19
------------------------------------------------------------------------------------------------------CAAGAGAAUUGACAGGUCUGA------------------------------------------------------------------------------------   3   21
----------------------------------------------------------------------------------------------------------------------------------------------------------------------------------AAUAGAAUCUGUUGUGCU-----------   3   18
```

P. pallidum ppa-mir-1202

```
...(((((((.((.(((((((((.(((((.(((((((((.(((.((..((((((.((...(((.((((.(((((.....)))))..))))...))).))))))))..)).))).)..((((((((...((((..(((...)))..))))....)))))))))))))))))).)))))).))))))))).)).))))))))...    -101.16 kcal/mol
CCAUCGGAUGaUGCUUAACAGAUUUAAUUUcAAUUGGUACCAAGCUGACAUGUCUCUUUGUUUUGAAAGGGUAUCUUUAGACCAAAGACCACCCCUCUUCUACAAGAGAAUUGACAGGUCUGAUGGCUAUCUACAGAAUAGUCGCUAAGCUGUCUCUUAGUCGUCUGGUGUCAAGUGAaAUAGAAUCUGUUGUGCUUCaUCCGAGUA    count  length
----------------------------------------------------------------------------------------------------------------------------------------------------------------------------------AAUAGAAUCUGUUGUGCUUCAUC------    2      23
-------------------------------------------------------------------------------------------------------------------------------------------------UAAGCUGUCUCUUAGUCGUCU-----------------------------------------    1      21
------------------------------------------------------------------------------------------------------------------------------------------------------------------------------------UAGAAUCUGUUGUGCUUCAU-------    1      20
```

```
CCA          U   U            A       A               G    A   UG       U   UUU    A--     A-      AG
   UCGGAUGa GC UAACAGAUUU AUUUcA UUGGUACCA----A CUG CA  UCUCUU GU    GAA    GGGU  UCUUU  A
   AGCCUaCU CG GUUGUCUAAG UAaAGU AACUGUGGU\    U GAC GU  AGAGAA-CA    CUU    CCCA  AGAAA  /
AUG          U   U            A       G          CGCU G    A   UA          U--    CUC     CC      CC
                                                 UA
                                                 GU     UCU-     AA    U
                                                 CGGCUA     ACAG   UAG C
                                                 \-UGAU     UGUC   AUC /
                                                       UCUC     GA    G
```

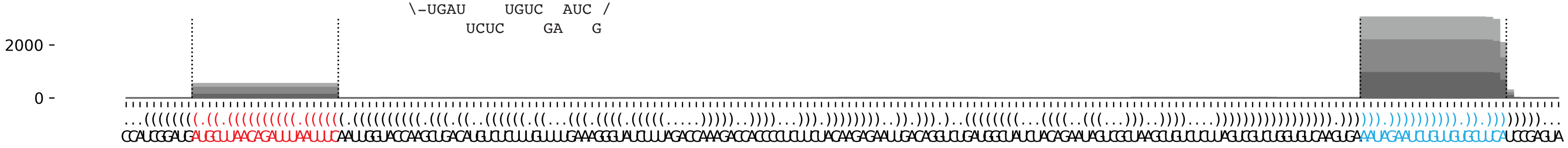

A. subglobosum asu-mir-1203

```
..((.((((((((((.((((((((((((((((.((.((((((((.(((.((....)).))).)).........)))))).)).))))))))))))))))).))))))))))).))..   -52.88 kcal/mol
AUCGUCUGUAcUUAUCUAAUACCCAAUAAAcAACAGUAAUCAGUAUCUAUCACCAGACAGAAGCAAGUCAAGUUGGUUAAUGAUGUuUAUUGGGUAUUAAAUAAGUaUGGUUGUC   count   length
Libraries combined         Precision: [ total = 92.0% | 5p-arm = 66.7% | 3p-arm = 92.1% ]
--------------------------------------------------------------------------------------UUAUUGGGUAUUAAAUAAGUA--------   1682    21
--------------------------------------------------------------------------------------UUAUUGGGUAUUAAAUAAGU---------   388     20
--------------------------------------------------------------------------------------UUAUUGGGUAUUAAAUAAGUAU-------   172     22
------------------------------------------------------------------------------------------UGGGUAUUAAAUAAGUAUGGU----   143     21
-----------------------------------------------------------------------------------------UUGGGUAUUAAAUAAGUAUGG-----   44      21
--------------------------------------------------------------------------------------UUAUUGGGUAUUAAAUAAG----------   5       19
-------------------------------------------------------------------------------------UUUAUUGGGUAUUAAAUAAGU---------   2       21
----------CUUAUCUAAUACCCAAUAAAC------------------------------------------------------------------------------------   4       21
-------------------------------------AAUCAGUAUCUAUCACCAGAC---------------------------------------------------------   2       21
--------------------------------------------------------------------------------------UUAUUGGGUAUUAAAUAAGUAUG------   1       23
-----------------------------------------------------------------------------------------UUGGGUAUUAAAUAAGUA--------   2       18
------------------------------------------------------------------------------------------UGGGUAUUAAAUAAGUAUGG-----   3       20
Library = 1        Precision: [ total = 93.7% | 5p-arm = 50.0% | 3p-arm = 93.8% ]
--------------------------------------------------------------------------------------UUAUUGGGUAUUAAAUAAGUA--------   944     21
--------------------------------------------------------------------------------------UUAUUGGGUAUUAAAUAAGU---------   216     20
--------------------------------------------------------------------------------------UUAUUGGGUAUUAAAUAAGUAU-------   86      22
------------------------------------------------------------------------------------------UGGGUAUUAAAUAAGUAUGGU----   57      21
-----------------------------------------------------------------------------------------UUGGGUAUUAAAUAAGUAUGG-----   24      21
--------------------------------------------------------------------------------------UUAUUGGGUAUUAAAUAAG----------   3       19
-------------------------------------------------------------------------------------UUUAUUGGGUAUUAAAUAAGU---------   2       21
----------CUUAUCUAAUACCCAAUAAAC------------------------------------------------------------------------------------   1       21
-------------------------------------AAUCAGUAUCUAUCACCAGAC---------------------------------------------------------   1       21
--------------------------------------------------------------------------------------UUAUUGGGUAUUAAAUAAGUAUG------   1       23
Library = 2        Precision: [ total = 90.3% | 5p-arm = 75.0% | 3p-arm = 90.4% ]
--------------------------------------------------------------------------------------UUAUUGGGUAUUAAAUAAGUA--------   562     21
--------------------------------------------------------------------------------------UUAUUGGGUAUUAAAUAAGU---------   136     20
--------------------------------------------------------------------------------------UUAUUGGGUAUUAAAUAAGUAU-------   79      22
------------------------------------------------------------------------------------------UGGGUAUUAAAUAAGUAUGGU----   61      21
-----------------------------------------------------------------------------------------UUGGGUAUUAAAUAAGUAUGG-----   17      21
----------CUUAUCUAAUACCCAAUAAAC------------------------------------------------------------------------------------   3       21
------------------------------------------------------------------------------------------UGGGUAUUAAAUAAGUAUGG-----   3       20
--------------------------------------------------------------------------------------UUAUUGGGUAUUAAAUAAG----------   2       19
-----------------------------------------------------------------------------------------UUGGGUAUUAAAUAAGUA--------   2       18
-------------------------------------AAUCAGUAUCUAUCACCAGAC---------------------------------------------------------   1       21
Library = 3        Precision: [ total = 88.7% | 5p-arm = 0% | 3p-arm = 88.7% ]
--------------------------------------------------------------------------------------UUAUUGGGUAUUAAAUAAGUA--------   176     21
--------------------------------------------------------------------------------------UUAUUGGGUAUUAAAUAAGU---------   36      20
------------------------------------------------------------------------------------------UGGGUAUUAAAUAAGUAUGGU----   25      21
--------------------------------------------------------------------------------------UUAUUGGGUAUUAAAUAAGUAU-------   7       22
-----------------------------------------------------------------------------------------UUGGGUAUUAAAUAAGUAUGG-----   3       21
```

```
AU   U           C                 A  G                    A   A  A
  CG CUGUAcUUAU  UAAUACCCAAUAAAcA  CA UAAUCA---------GU UCU UC C
  GU GGUaUGAAUA  AUUAUGGGUUAUuUGU  GU AUUGGU         CG AGA AG C
CU   U           A                 A  A      UGAACUGAA  A   C  A
```

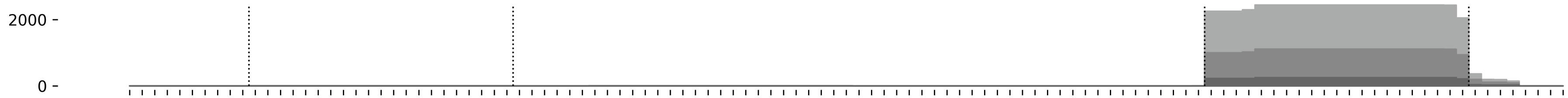


```
..((.((((((((((.((((((((((((((((.((.((((((((.(((.((....)).))).)).........)))))).)).))))))))))))))))).))))))))))).))..
AUCGUCUGUACUUAUCUAAUACCCAAUAAACAACAGUAAUCAGUAUCUAUCACCAGACAGAAGCAAGUCAAGUUGGUUAAUGAUGUUUAUUGGGUAUUAAAUAAGUAUGGUUGUC
```

A. subglobosum asu-mir-1204-P1-8

```
((((.((((.((((((..(((((((((((((((((.((.(((.((.(((...))))).))).)).)))))))))))))))))..)))))).))))))))). -48.86 kcal/mol
AUUGGAUGUUuCAAGUGAGGUAAUGGAUAAuAGAUAUUGAUUGAAAUAGCUUUGCUUUACGGGCACUGUUUAUuAUCUAUUGUAGCACUUGUGaCACCAGUU count    length
Libraries combined         Precision: [ total = 99.5% | 5p-arm = 98.4% | 3p-arm = 99.5% ]
-------------------------------------------------------------------------UAUCUAUUGUAGCACUUGUGA-------- 2237498  21
----------UCAAGUGAGGUAAUGGAUAAU----------------------------------------------------------------------- 9135     21
-------------------------------------------------------------------------UAUCUAUUGUAGCACUUGUG--------- 5991     20
-----------------------------------------------------------------------------UAUUGUAGCACUUGUGACACC---- 4953     21
------------------------------------------------------------------------UUAUCUAUUGUAGCACUUGUGA-------- 3192     22
-------------------------------------------------------------------------UAUCUAUUGUAGCACUUG----------- 2618     18
-------------------------------------------------------------------------UAUCUAUUGUAGCACUUGU---------- 1334     19
-------------------------------------------------------------------------UAUCUAUUGUAGCACUUGUGAC------- 792      22
--------------------------------------------------------------------------AUCUAUUGUAGCACUUGUGA-------- 597      20
-----------------------------------------------------------------------------UAUUGUAGCACUUGUGACAC----- 401      20
-------------------------------------------------------------------------UAUCUAUUGUAGCACUUGUGACA------ 390      23
----------UCAAGUGAGGUAAUGGAUAA------------------------------------------------------------------------ 338      20
-------------------------------------------------------------------------UAUCUAUUGUAGCACUUGUGACACC---- 294      25
-------------------------------------------------------------------------UAUCUAUUGUAGCACUUGUGACACCAG-- 280      27
--------------------------------------------------------------------------AUCUAUUGUAGCACUUGUGAC------- 121      21
----------UCAAGUGAGGUAAUGGAUA------------------------------------------------------------------------- 131      19
-----------------------------------------------------------------------------UAUUGUAGCACUUGUGACA------ 112      19
----------UCAAGUGAGGUAAUGGAUAAUA---------------------------------------------------------------------- 104      22
-------------------------------------------------------------------------UAUCUAUUGUAGCACUUGUGACAC----- 89       24
-------------------------------------------------------------------------UAUCUAUUGUAGCACUUGUGACACCA--- 86       26
-------------------------------------------------------------------------UAUCUAUUGUAGCACUUGUGACACCAGU- 62       28
---------------------------------------------------------------------------UCUAUUGUAGCACUUGUGA-------- 50       19
---------------------------------------------------------------------------UCUAUUGUAGCACUUGUGACACC---- 51       23
----------------------------------------------------------------------------CUAUUGUAGCACUUGUGA-------- 48       18
------UGUUUCAAGUGAGGUAAUGGA--------------------------------------------------------------------------- 38       21
----------UCAAGUGAGGUAAUGGAU-------------------------------------------------------------------------- 51       18
--------UUUCAAGUGAGGUAAUGGAUA------------------------------------------------------------------------- 31       21
---------UUCAAGUGAGGUAAUGGAUAA------------------------------------------------------------------------ 29       21
------------------------------------------------------------------------UUAUCUAUUGUAGCACUUGUG--------- 17       21
--------------------------------------------------------------------------AUCUAUUGUAGCACUUGUGACA------ 23       22
-----------------------------------------------------------------------------UAUUGUAGCACUUGUGACACCA--- 21       22
---------------------------------------------------------------------------UCUAUUGUAGCACUUGUGACA------ 23       21
----------------------------------------------------------------------UAUUAUCUAUUGUAGCACU------------- 12       19
---------------------------------------------------------------------------UCUAUUGUAGCACUUGUGACAC----- 9        22
-----------CAAGUGAGGUAAUGGAUAAU----------------------------------------------------------------------- 10       20
----------------------------------------------------------------------UAUUAUCUAUUGUAGCACUUG----------- 9        21
-----------------------------------------------------------------------------UAUUGUAGCACUUGUGAC------- 12       18
--------------------------------------------------------------------------AUCUAUUGUAGCACUUGUGACACC---- 6        24
---------------------------------------------------------------------------UCUAUUGUAGCACUUGUGAC------- 7        20
------UGUUUCAAGUGAGGUAAUGGAU-------------------------------------------------------------------------- 8        22
---------UUCAAGUGAGGUAAUGGAUAAU----------------------------------------------------------------------- 5        22
--------------------------------------------------------------------------AUCUAUUGUAGCACUUGUGACACCAG-- 6        26
----------------------------------------------------------------------------CUAUUGUAGCACUUGUGACACC---- 10       22
AUUGGAUGUUUCAAGUGAGGUAAUGGAUAAU----------------------------------------------------------------------- 3        31
------UGUUUCAAGUGAGGUAAUG----------------------------------------------------------------------------- 5        19
--------UUUCAAGUGAGGUAAUGGAUAA------------------------------------------------------------------------ 7        22
--------------------UAAUGGAUAAUAGAUAUUGAU------------------------------------------------------------- 2        21
---------------------AAUGGAUAAUAGAUAUUGAU------------------------------------------------------------- 2        20
-----------------------------------------------------------------------AUUAUCUAUUGUAGCACUUGU---------- 3        21
------------------------------------------------------------------------UUAUCUAUUGUAGCACUUGUGAC------- 5        23
------------------------------------------------------------------------UUAUCUAUUGUAGCACUUGUGACA------ 2        24
------------------------------------------------------------------------UUAUCUAUUGUAGCACUUG----------- 3        19
---GGAUGUUUCAAGUGAGGUA-------------------------------------------------------------------------------- 1        19
```

A. subglobosum asu-mir-1204 continued

```
((((.((((.((((((..((((((((((((((((((.((.(((.((.(((...)))))).))).)).))))))))))))))))..)))))).))))))))). -48.86 kcal/mol
AUUGGAUGUUuCAAGUGAGGUAAUGGAUAAuAGAUAUUGAUUGAAAUAGCUUUGCUUUACGGGCACUGUUUAUuAUCUAUUGUAGCACUUGUGaCACCAGUU count    length
Libraries combined          Precision: [ total = 99.5% | 5p-arm = 98.4% | 3p-arm = 99.5% ]
---GGAUGUUUCAAGUGAGGUAA------------------------------------------------------------------------------- 1        20
-------GUUUCAAGUGAGGUAAUGGAUA------------------------------------------------------------------------- 1        22
--------UUUCAAGUGAGGUAAUGGAUAAU----------------------------------------------------------------------- 1        23
---------UUCAAGUGAGGUAAUGGAUA------------------------------------------------------------------------- 3        20
------------AAGUGAGGUAAUGGAUAAU----------------------------------------------------------------------- 1        19
----------------GAGGUAAUGGAUAAUAGAU------------------------------------------------------------------- 1        19
-------------------------------------------------------UUUACGGGCACUGUUUAU----------------------------- 1        18
--------------------------------------------------------UUACGGGCACUGUUUAUUAUC------------------------- 6        21
----------------------------------------------------------ACGGGCACUGUUUAUUAUC------------------------- 1        19
----------------------------------------------------------ACGGGCACUGUUUAUUAUCUA----------------------- 1        21
-------------------------------------------------------------GGCACUGUUUAUUAUCUAUUG-------------------- 1        21
----------------------------------------------------------------------UAUUAUCUAUUGUAGCACUUGU---------- 2        22
------------------------------------------------------------------------UUAUCUAUUGUAGCACUUGU---------- 5        20
------------------------------------------------------------------------UUAUCUAUUGUAGCACUUGUGACACC---- 2        26
-------------------------------------------------------------------------UAUCUAUUGUAGCACUUGUGACACCAGUU 3        29
--------------------------------------------------------------------------AUCUAUUGUAGCACUUGUGACACCA--- 2        25
------------------------------------------------------------------------------AUUGUAGCACUUGUGACACC---- 1        20
-------------------------------------------------------------------------------UUGUAGCACUUGUGACACC---- 1        19
-------------------------------------------------------------------------------UUGUAGCACUUGUGACACCAG-- 3        21
----------------------------------------------------------------------------------UAGCACUUGUGACACCAGUU 2        20
AUUGGAUGUUUCAAGUGAGGUAAUGGAUAA------------------------------------------------------------------------ 1        30
---GGAUGUUUCAAGUGAGGUAAUGGA--------------------------------------------------------------------------- 1        24
------UGUUUCAAGUGAGGUAAUGG---------------------------------------------------------------------------- 1        20
-----------CAAGUGAGGUAAUGGAUAA------------------------------------------------------------------------ 2        19
----------------GAGGUAAUGGAUAAUAGAUAUU---------------------------------------------------------------- 1        22
-----------------------------------AUUGAUUGAAAUAGCUUUGCU---------------------------------------------- 1        21
-------------------------------------------AAAUAGCUUUGCUUUACGGGC-------------------------------------- 1        21
--------------------------------------------------------------------UUUAUUAUCUAUUGUAGCACU------------- 2        21
----------------------------------------------------------------------UAUUAUCUAUUGUAGCACUU------------ 2        20
------------------------------------------------------------------------UUAUCUAUUGUAGCACUUGUGACAC----- 1        25
--------------------------------------------------------------------------AUCUAUUGUAGCACUUGUG--------- 1        19
---------------------------------------------------------------------------UCUAUUGUAGCACUUGUGACACCA--- 1        24
---------------------------------------------------------------------------UCUAUUGUAGCACUUGUGACACCAG-- 2        25
--------------------------------------------------------------------------------UGUAGCACUUGUGACACCAGU- 1        21
----------------------------------------------------------------------------------UAGCACUUGUGACACCAGU- 1        19
```

```
      A    u      AG                 U  A   A  U   U
 AUUGG UGUU CAAGUG  GUAAUGGAUAAuAGAUA UG UUG AA AGC U
 UGACC-ACaG GUUCAC  UGUUAUCUAuUAUUUGU AC GGC UU-UCG /
U          U      GA                 C  G   A      U
```

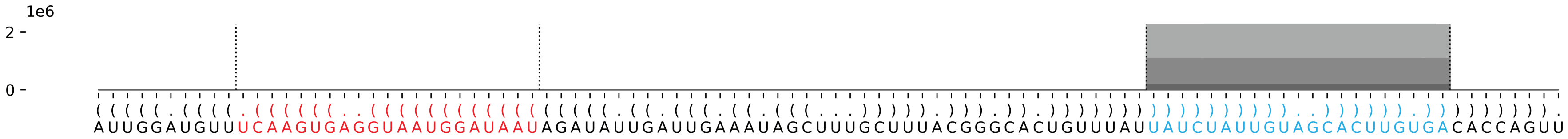

A. subglobosum asu-mir-1205

```
....((((((((((((((((..(.((((.((((((((..((((((((..((.((((((....(........)....)))))).))..)))))))).)))).)))).)))).)..))))))))))))))))....   -70.5 kcal/mol
UAUUAUGUCUuGAUGAACAUUUGAGUGGCGuUGAAUGGAAGUGUCUCAAUUAUGUUGACAUUCAUUCAAUCGUUCAUUGACAAGAGAGAGAUACUACAUUGCAGCuCCACACUUAUGUUCAUCAAgACAUCGAC   count    length
Libraries combined          Precision: [ total = 98.5% | 5p-arm = 98.6% | 3p-arm = 85.7% ]
----------UGAUGAACAUUUGAGUGGCGU-------------------------------------------------------------------------------------------------------   8502     21
----------UGAUGAACAUUUGAGUGGCG--------------------------------------------------------------------------------------------------------   584      20
----------UGAUGAACAUUUGAGUGGC---------------------------------------------------------------------------------------------------------   153      19
----------UGAUGAACAUUUGAGUGGCGUU------------------------------------------------------------------------------------------------------   123      22
----------UGAUGAACAUUUGAGUGG----------------------------------------------------------------------------------------------------------   68       18
---------UUGAUGAACAUUUGAGUGGCGU-------------------------------------------------------------------------------------------------------   49       22
---------UUGAUGAACAUUUGAGUGGCG--------------------------------------------------------------------------------------------------------   27       21
-----------GAUGAACAUUUGAGUGGCGU-------------------------------------------------------------------------------------------------------   30       20
---------UUGAUGAACAUUUGAGUGG----------------------------------------------------------------------------------------------------------   9        19
---------UUGAUGAACAUUUGAGUGGC---------------------------------------------------------------------------------------------------------   13       20
---------------------------------------------------------------------------------------------------------UCCACACUUAUGUUCAUCAAG--------   6        21
---------UUGAUGAACAUUUGAGUG-----------------------------------------------------------------------------------------------------------   3        18
---UAUGUCUUGAUGAACAUUUGAGUGGCGU-------------------------------------------------------------------------------------------------------   1        28
---------UUGAUGAACAUUUGAGUGGCGUU------------------------------------------------------------------------------------------------------   2        23
----------UGAUGAACAUUUGAGUGGCGUUG-----------------------------------------------------------------------------------------------------   1        23
------------------------------------------------------------UUCAUUCAAUCGUUCAUUGACAAGA-------------------------------------------------   1        25
-----------GAUGAACAUUUGAGUGGCG--------------------------------------------------------------------------------------------------------   3        19
-------------UGAACAUUUGAGUGGCGU-------------------------------------------------------------------------------------------------------   1        18
Library = 1        Precision: [ total = 98.6% | 5p-arm = 98.7% | 3p-arm = 75.0% ]
----------UGAUGAACAUUUGAGUGGCGU-------------------------------------------------------------------------------------------------------   3976     21
----------UGAUGAACAUUUGAGUGGCG--------------------------------------------------------------------------------------------------------   298      20
----------UGAUGAACAUUUGAGUGGC---------------------------------------------------------------------------------------------------------   78       19
----------UGAUGAACAUUUGAGUGGCGUU------------------------------------------------------------------------------------------------------   63       22
----------UGAUGAACAUUUGAGUGG----------------------------------------------------------------------------------------------------------   31       18
---------UUGAUGAACAUUUGAGUGGCGU-------------------------------------------------------------------------------------------------------   21       22
---------UUGAUGAACAUUUGAGUGGCG--------------------------------------------------------------------------------------------------------   18       21
-----------GAUGAACAUUUGAGUGGCGU-------------------------------------------------------------------------------------------------------   10       20
---------UUGAUGAACAUUUGAGUGG----------------------------------------------------------------------------------------------------------   4        19
---------UUGAUGAACAUUUGAGUGGC---------------------------------------------------------------------------------------------------------   3        20
---------------------------------------------------------------------------------------------------------UCCACACUUAUGUUCAUCAAG--------   3        21
---------UUGAUGAACAUUUGAGUG-----------------------------------------------------------------------------------------------------------   2        18
---UAUGUCUUGAUGAACAUUUGAGUGGCGU-------------------------------------------------------------------------------------------------------   1        28
---------UUGAUGAACAUUUGAGUGGCGUU------------------------------------------------------------------------------------------------------   1        23
----------UGAUGAACAUUUGAGUGGCGUUG-----------------------------------------------------------------------------------------------------   1        23
------------------------------------------------------------UUCAUUCAAUCGUUCAUUGACAAGA-------------------------------------------------   1        25
Library = 2        Precision: [ total = 98.5% | 5p-arm = 98.5% | 3p-arm = 100.0% ]
----------UGAUGAACAUUUGAGUGGCGU-------------------------------------------------------------------------------------------------------   3687     21
----------UGAUGAACAUUUGAGUGGCG--------------------------------------------------------------------------------------------------------   243      20
----------UGAUGAACAUUUGAGUGGC---------------------------------------------------------------------------------------------------------   67       19
----------UGAUGAACAUUUGAGUGGCGUU------------------------------------------------------------------------------------------------------   47       22
----------UGAUGAACAUUUGAGUGG----------------------------------------------------------------------------------------------------------   33       18
---------UUGAUGAACAUUUGAGUGGCGU-------------------------------------------------------------------------------------------------------   24       22
-----------GAUGAACAUUUGAGUGGCGU-------------------------------------------------------------------------------------------------------   14       20
---------UUGAUGAACAUUUGAGUGGCG--------------------------------------------------------------------------------------------------------   9        21
---------UUGAUGAACAUUUGAGUGGC---------------------------------------------------------------------------------------------------------   8        20
---------UUGAUGAACAUUUGAGUGG----------------------------------------------------------------------------------------------------------   3        19
-----------GAUGAACAUUUGAGUGGCG--------------------------------------------------------------------------------------------------------   3        19
---------------------------------------------------------------------------------------------------------UCCACACUUAUGUUCAUCAAG--------   2        21
---------UUGAUGAACAUUUGAGUG-----------------------------------------------------------------------------------------------------------   1        18
---------UUGAUGAACAUUUGAGUGGCGUU------------------------------------------------------------------------------------------------------   1        23
-------------UGAACAUUUGAGUGGCGU-------------------------------------------------------------------------------------------------------   1        18
Library = 3        Precision: [ total = 98.5% | 5p-arm = 98.5% | 3p-arm = 100.0% ]
----------UGAUGAACAUUUGAGUGGCGU-------------------------------------------------------------------------------------------------------   839      21
```

A. subgloboshum asu-mir-1205 continued

```
....((((((((((((((((..(.((((.((((((((..((((((((..((.((((((....(........)....)))))).))..)))))))).)))).)))).)))).)..))))))))))))))))....  -70.5 kcal/mol
UAUUAUGUCUuGAUGAACAUUUGAGUGGCGuUGAAUGGAAGUGUCUCAAUUAUGUUGACAUUCAUUCAAUCGUUCAUUGACAAGAGAGAGAUACUACAUUGCAGCuCCACACUUAUGUUCAUCAAgACAUCGAC  count   length
----------UGAUGAACAUUUGAGUGGCG--------------------------------------------------------------------------------------------------------  43      20
----------UGAUGAACAUUUGAGUGGCGUU------------------------------------------------------------------------------------------------------  13      22
----------UGAUGAACAUUUGAGUGGC---------------------------------------------------------------------------------------------------------  8       19
-----------GAUGAACAUUUGAGUGGCGU-------------------------------------------------------------------------------------------------------  6       20
---------UUGAUGAACAUUUGAGUGGCGU-------------------------------------------------------------------------------------------------------  4       22
----------UGAUGAACAUUUGAGUGG----------------------------------------------------------------------------------------------------------  4       18
---------UUGAUGAACAUUUGAGUGG----------------------------------------------------------------------------------------------------------  2       19
---------UUGAUGAACAUUUGAGUGGC---------------------------------------------------------------------------------------------------------  2       20
---------------------------------------------------------------------------------------------------------UCCACACUUAUGUUCAUCAAG--------  1       21
```

```
UAUU                UU A    C         GA        AA  A      CAUU AUU
    AUGUCUuGAUGAACAU  G GUGG GuUG-AAUG  AGUGUCUC  UU UGUUGA    C   C
    UACAgAACUACUUGUA  C CACC CGAC UUAC  UCAUAGAG  AG ACAGUU    G   A
CAGC                UU A    u    G    A-        AG  A      ACUU CUA
```

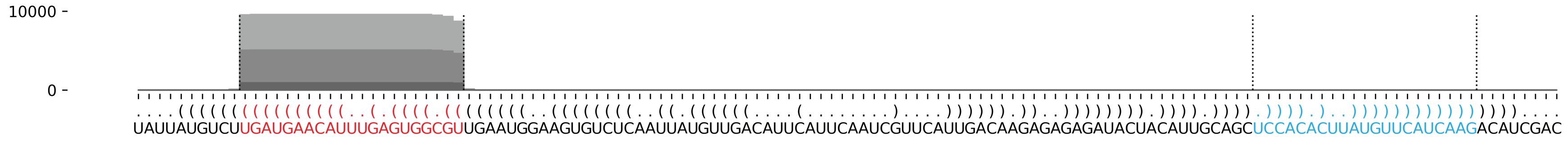

A. subglobosum asu-mir-1206

```
...(..(.((((((((.(((((((((((.(((...((((.((((((((((((.(((((((((((((..((.......))..)))))))).))))).)))))).)))...))).))))...))).))))))))))).)))))))).)..)...  -85.3 kcal/mol
UAUGUUAUCGaAUCGAGUGAGAUGAUGCUUaCCAAUGGAAUGACCAAGUGUCAGUGUUCAAACUGCAAUGUUAUAAUCACCGCAGUUUGUAAUACCGACGCUCUGGUAUUCAAUCCACCUGUAuGCAUCAUCUCGAUCGAUUCgCUUUCUAC  count   length
Libraries combined          Precision: [ total = 99.3% | 5p-arm = 50.0% | 3p-arm = 99.3% ]
---------------------------------------------------------------------------------------------------------------------------UGCAUCAUCUCGAUCGAUUCG--------  23374   21
----------------------------------------------------------------------------------------------------------------------------GCAUCAUCUCGAUCGAUUCG--------  131     20
---------------------------------------------------------------------------------------------------------------------------UGCAUCAUCUCGAUCGAUU----------  14      19
---------------------------------------------------------------------------------------------------------------------------UGCAUCAUCUCGAUCGAUUCGC-------  23      22
---------------------------------------------------------------------------------------------------------------------------UGCAUCAUCUCGAUCGAUUCGCU------  14      23
---------------------------------------------------------------------------------------------------------------------------UGCAUCAUCUCGAUCGAUUC---------  16      20
-------------------------------------------------------------------------------------------------------------------------------UCAUCUCGAUCGAUUCGCU------  8       19
----UUAUCGAAUCGAGUGAGAUGA-------------------------------------------------------------------------------------------------------------------------------  1       21
----UUAUCGAAUCGAGUGAGAU---------------------------------------------------------------------------------------------------------------------------------  1       19
-----UAUCGAAUCGAGUGAGAUGA-------------------------------------------------------------------------------------------------------------------------------  1       20
------AUCGAAUCGAGUGAGAUGAU------------------------------------------------------------------------------------------------------------------------------  1       20
----------AAUCGAGUGAGAUGAUGCUU--------------------------------------------------------------------------------------------------------------------------  1       20
---------------------------------------------AAGUGUCAGUGUUCAAACUGC--------------------------------------------------------------------------------------  1       21
-------------------------------------------------------------------------------------UUUGUAAUACCGACGCUCUGGU---------------------------------------------  1       22
--------------------------------------------------------------------------------------UUGUAAUACCGACGCUCUGGUAU-------------------------------------------  2       23
-------------------------------------------------------------------------------------------------------------------------UAUGCAUCAUCUCGAUCGAUUCGC-------  1       24
--------------------------------------------------------------------------------------------------------------------------AUGCAUCAUCUCGAUCGAUUCG--------  1       22
---------------------------------------------------------------------------------------------------------------------------UGCAUCAUCUCGAUCGAU-----------  1       18
-----------------------------------------------------------------------------------------------------------------------------CAUCAUCUCGAUCGAUUCGCU------  1       21
-------------------------------------------------------------------------------------------------------------------------------UCAUCUCGAUCGAUUCGCUUU----  2       21
-------------------------------------------------------------------------------------------------------------------------------UCAUCUCGAUCGAUUCGCUUUCU--  2       23
-------------------------------------------------------------------------------------------------------------------------------UCAUCUCGAUCGAUUCGCUUUC---  2       22
----------AAUCGAGUGAGAUGAUGCUUA-------------------------------------------------------------------------------------------------------------------------  4       21
-------------------------------------------------------------------------UAAUCACCGCAGUUUGUAAUAC---------------------------------------------------------  1       22
--------------------------------------------------------------------------------------UUGUAAUACCGACGCUCUGGU---------------------------------------------  1       21
--------------------------------------------------------------------------------------------------------------------------AUGCAUCAUCUCGAUCGAUUC---------  1       21
```

A. subglobosum asu-mir-1206 continued

```
...(..(.((((((((.(((((((((((.(((...((((.((((((((((((.(((((((((((((..((.......))..)))))))).))))).)))))).)))...))).)))).)))...))).))))))))))).)))))))).)..)...   -85.3 kcal/mol
UAUGUUAUCGaAUCGAGUGAGAUGAUGCUUaCCAAUGGAAUGACCAAGUGUCAGUGUUCAAACUGCAAUGUUAUAAUCACCGCAGUUUGUAAUACCGACGCUCUGGUAUUCAAUCCACCUGUAuGCAUCAUCUCGAUCGAUUCgCUUUCUAC   count   length
Library = 1        Precision: [ total = 99.3% | 5p-arm = 16.7% | 3p-arm = 99.3% ]
---------------------------------------------------------------------------------------------------------------------------UGCAUCAUCUCGAUCGAUUCG--------   11768   21
----------------------------------------------------------------------------------------------------------------------------GCAUCAUCUCGAUCGAUUCG--------   72      20
---------------------------------------------------------------------------------------------------------------------------UGCAUCAUCUCGAUCGAUU----------   8       19
---------------------------------------------------------------------------------------------------------------------------UGCAUCAUCUCGAUCGAUUCGC-------   8       22
---------------------------------------------------------------------------------------------------------------------------UGCAUCAUCUCGAUCGAUUCGCU------   4       23
---------------------------------------------------------------------------------------------------------------------------UGCAUCAUCUCGAUCGAUUC---------   3       20
-------------------------------------------------------------------------------------------------------------------------------UCAUCUCGAUCGAUUCGCU------   2       19
----UUAUCGAAUCGAGUGAGAUGA-------------------------------------------------------------------------------------------------------------------------------   1       21
----UUAUCGAAUCGAGUGAGAU---------------------------------------------------------------------------------------------------------------------------------   1       19
-----UAUCGAAUCGAGUGAGAUGA-------------------------------------------------------------------------------------------------------------------------------   1       20
------AUCGAAUCGAGUGAGAUGAU------------------------------------------------------------------------------------------------------------------------------   1       20
----------AAUCGAGUGAGAUGAUGCUU--------------------------------------------------------------------------------------------------------------------------   1       20
---------------------------------------------AAGUGUCAGUGUUCAAACUGC--------------------------------------------------------------------------------------   1       21
-------------------------------------------------------------------------------------UUUGUAAUACCGACGCUCUGGU---------------------------------------------   1       22
--------------------------------------------------------------------------------------UUGUAAUACCGACGCUCUGGUAU-------------------------------------------   1       23
-------------------------------------------------------------------------------------------------------------------------UAUGCAUCAUCUCGAUCGAUUCGC-------   1       24
--------------------------------------------------------------------------------------------------------------------------AUGCAUCAUCUCGAUCGAUUCG--------   1       22
---------------------------------------------------------------------------------------------------------------------------UGCAUCAUCUCGAUCGAU-----------   1       18
-----------------------------------------------------------------------------------------------------------------------------CAUCAUCUCGAUCGAUUCGCU------   1       21
-------------------------------------------------------------------------------------------------------------------------------UCAUCUCGAUCGAUUCGCUUU----   1       21
-------------------------------------------------------------------------------------------------------------------------------UCAUCUCGAUCGAUUCGCUUUCU--   1       23
-------------------------------------------------------------------------------------------------------------------------------UCAUCUCGAUCGAUUCGCUUUC---   1       22
Library = 2        Precision: [ total = 99.4% | 5p-arm = 100.0% | 3p-arm = 99.4% ]
---------------------------------------------------------------------------------------------------------------------------UGCAUCAUCUCGAUCGAUUCG--------   9733    21
----------------------------------------------------------------------------------------------------------------------------GCAUCAUCUCGAUCGAUUCG--------   50      20
---------------------------------------------------------------------------------------------------------------------------UGCAUCAUCUCGAUCGAUUC---------   12      20
---------------------------------------------------------------------------------------------------------------------------UGCAUCAUCUCGAUCGAUUCGC-------   11      22
---------------------------------------------------------------------------------------------------------------------------UGCAUCAUCUCGAUCGAUUCGCU------   8       23
---------------------------------------------------------------------------------------------------------------------------UGCAUCAUCUCGAUCGAUU----------   6       19
----------AAUCGAGUGAGAUGAUGCUUA-------------------------------------------------------------------------------------------------------------------------   4       21
-------------------------------------------------------------------------------------------------------------------------------UCAUCUCGAUCGAUUCGCU------   4       19
-------------------------------------------------------------------------UAAUCACCGCAGUUUGUAAUAC---------------------------------------------------------   1       22
--------------------------------------------------------------------------------------UUGUAAUACCGACGCUCUGGUAU-------------------------------------------   1       23
--------------------------------------------------------------------------------------UUGUAAUACCGACGCUCUGGU---------------------------------------------   1       21
--------------------------------------------------------------------------------------------------------------------------AUGCAUCAUCUCGAUCGAUUC---------   1       21
-------------------------------------------------------------------------------------------------------------------------------UCAUCUCGAUCGAUUCGCUUU----   1       21
-------------------------------------------------------------------------------------------------------------------------------UCAUCUCGAUCGAUUCGCUUUCU--   1       23
-------------------------------------------------------------------------------------------------------------------------------UCAUCUCGAUCGAUUCGCUUUC---   1       22
Library = 3        Precision: [ total = 99.4% | 5p-arm = 0% | 3p-arm = 99.4% ]
---------------------------------------------------------------------------------------------------------------------------UGCAUCAUCUCGAUCGAUUCG--------   1873    21
----------------------------------------------------------------------------------------------------------------------------GCAUCAUCUCGAUCGAUUCG--------   9       20
---------------------------------------------------------------------------------------------------------------------------UGCAUCAUCUCGAUCGAUUCGC-------   4       22
---------------------------------------------------------------------------------------------------------------------------UGCAUCAUCUCGAUCGAUUCGCU------   2       23
-------------------------------------------------------------------------------------------------------------------------------UCAUCUCGAUCGAUUCGCU------   2       19
---------------------------------------------------------------------------------------------------------------------------UGCAUCAUCUCGAUCGAUUC---------   1       20
```

```
UAU UU U        G           U   CAA    A                A              AA  UUA
   G  A CGaAUCGA UGAGAUGAUGC UaC   UGGA UGA---CCA-AGUGUC GUGUU-CAAACUGC  UG    U
   C  U gCUUAGCU GCUCUACUACG AUG   ACCU ACU   GGU UCGCAG CAUAA GUUUGACG  AC    /
CAU UU C        A           u   UCC    A   UAU   C      C     U        CC  UAA
```

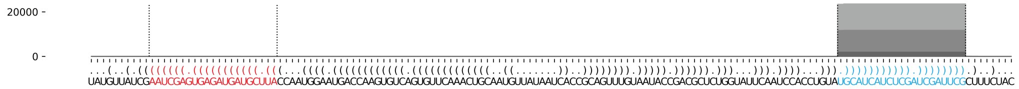

A. subglobosum asu-mir-1207

```
.....((((.(((((...((((((((((((((((.(((((((((.......((..((.(((((((((((((.(.....(((.(((....)))))).....).)))))))))))))))...))))))))))).))))))))))))))))...))))).)))).....   -87.6 kcal/mol
AACACACACUuGCGCUAUCUGUUCAAACAAuCGGAGUUACCGAGUACAACUCCUACGAUUAUAAGUGUCACGGACUACUCACUCAGCAAUGAUGAAUCAAUGGUGAUACUUGUGACGCAUGGCUCGGUAGUAUCGAUuGUUUGGACAGCUUGCGUAUgUGUUUACC   count   length
```

Libraries combined Precision: [total = 99.9% | 5p-arm = 87.5% | 3p-arm = 99.9%]

Read	count	length
UGUUUGGACAGCUUGCGUAUG	10264	21
UGUUUGGACAGCUUGCGUAUGU	354	22
UGUUUGGACAGCUUGCGUAU	220	20
UGUUUGGACAGCUUGCGUA	81	19
UGUUUGGACAGCUUGCGU	26	18
UUGUUUGGACAGCUUGCGUAUG	6	22
UGCGCUAUCUGUUCAAACAAU	6	21
AUUAUAAGUGUCACGGACU	1	19
UGGCUCGGUAGUAUCGAUUGU	1	21
GAUUGUUUGGACAGCUUGCGU	1	21
UUGUUUGGACAGCUUGCGUAU	2	21
GUUUGGACAGCUUGCGUAUG	1	20
UUUGGACAGCUUGCGUAUG	1	19
UGCGCUAUCUGUUCAAACAAUC	1	22
UGUUUGGACAGCUUGCGUAUGUG	1	23

Library = 1 Precision: [total = 99.8% | 5p-arm = 80.0% | 3p-arm = 99.8%]

Read	count	length
UGUUUGGACAGCUUGCGUAUG	4784	21
UGUUUGGACAGCUUGCGUAUGU	171	22
UGUUUGGACAGCUUGCGUAU	104	20
UGUUUGGACAGCUUGCGUA	39	19
UGUUUGGACAGCUUGCGU	13	18
UUGUUUGGACAGCUUGCGUAUG	5	22
UGCGCUAUCUGUUCAAACAAU	4	21
AUUAUAAGUGUCACGGACU	1	19
UGGCUCGGUAGUAUCGAUUGU	1	21
GAUUGUUUGGACAGCUUGCGU	1	21
UUGUUUGGACAGCUUGCGUAU	1	21
GUUUGGACAGCUUGCGUAUG	1	20
UUUGGACAGCUUGCGUAUG	1	19

Library = 2 Precision: [total = 100.0% | 5p-arm = 100.0% | 3p-arm = 100.0%]

Read	count	length
UGUUUGGACAGCUUGCGUAUG	4393	21
UGUUUGGACAGCUUGCGUAUGU	145	22
UGUUUGGACAGCUUGCGUAU	97	20
UGUUUGGACAGCUUGCGUA	34	19
UGUUUGGACAGCUUGCGU	12	18
UGCGCUAUCUGUUCAAACAAU	2	21
UGCGCUAUCUGUUCAAACAAUC	1	22
UUGUUUGGACAGCUUGCGUAUG	1	22
UGUUUGGACAGCUUGCGUAUGUG	1	23

Library = 3 Precision: [total = 99.9% | 5p-arm = 0% | 3p-arm = 99.9%]

Read	count	length
UGUUUGGACAGCUUGCGUAUG	1087	21
UGUUUGGACAGCUUGCGUAUGU	38	22
UGUUUGGACAGCUUGCGUAU	19	20
UGUUUGGACAGCUUGCGUA	8	19
UUGUUUGGACAGCUUGCGUAU	1	21
UGUUUGGACAGCUUGCGU	1	18

```
AACAC     U    UAU                  A          UACAACU  UA-   A               G ACUAC    C   G
     ACAC uGCGC   CUGUUCAAACAAuCGG GUUACCGAG        CC    CG UUAUAAGUGUCAC G      UCA UCA C
     UGUg AUGCG   GACAGGUUUGuUAGCU UGAUGGCUC-------GG    GC-AGUGUUCAUAGUG U      AGU-AGU A
CCAUU     U    UUC                  A                  UAC                 G AACUA        A
```

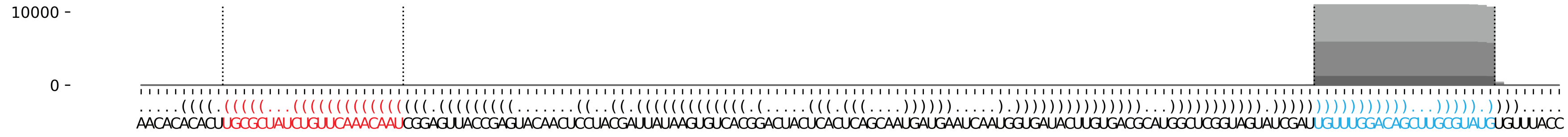

A. subglobosum asu-mir-1208

```
(((...((.(((((..(((((.(((((.((((.((.((((.....((((((((..(((((....)))))))))))))...((((.((((...........)))).)))))))).)).)))).))))).)))))))))).))..))).     -68.63 kcal/mol
GCGACUAGUGuGCUGUGAGAUUGGUCACCGAGcUGAUAGUAAGAAGGAGGUUGACGCACUGUAGAGUGCCAGUCUCCAACAUAACCAACAUUUAAUCUCAGUUGUUUAUACUACCAUCuCGUUGACUUAUCUCAGCACcCUUACGCG     count   length
Libraries combined          Precision: [ total = 97.8% | 5p-arm = 97.9% | 3p-arm = 89.8% ]
----------UGCUGUGAGAUUGGUCACCGAGC------------------------------------------------------------------------------------------------------------------     4493    23
----------UGCUGUGAGAUUGGUCACCG---------------------------------------------------------------------------------------------------------------------     3444    20
----------UGCUGUGAGAUUGGUCACCGAG-------------------------------------------------------------------------------------------------------------------     1724    22
----------UGCUGUGAGAUUGGUCACCGA--------------------------------------------------------------------------------------------------------------------     1025    21
----------------------------------------------------------------------------------------------------------------------UCGUUGACUUAUCUCAGCACC--------     211     21
----------UGCUGUGAGAUUGGUCACC----------------------------------------------------------------------------------------------------------------------     135     19
----------UGCUGUGAGAUUGGUCAC-----------------------------------------------------------------------------------------------------------------------     132     18
-----------GCUGUGAGAUUGGUCACCGAGC------------------------------------------------------------------------------------------------------------------     78      22
------------CUGUGAGAUUGGUCACCGAGCUG----------------------------------------------------------------------------------------------------------------     43      23
-----------GCUGUGAGAUUGGUCACCGAG-------------------------------------------------------------------------------------------------------------------     47      21
----------UGCUGUGAGAUUGGUCACCGAGCU-----------------------------------------------------------------------------------------------------------------     29      24
-------------UGUGAGAUUGGUCACCGAGCUG----------------------------------------------------------------------------------------------------------------     16      22
---------------UGAGAUUGGUCACCGAGCUG----------------------------------------------------------------------------------------------------------------     18      20
--------------------------------------------------------------------------------------------------------------------UCUCGUUGACUUAUCUCAGC-----------     17      20
-----------GCUGUGAGAUUGGUCACCG---------------------------------------------------------------------------------------------------------------------     14      19
----------UGCUGUGAGAUUGGUCACCGAGCUG----------------------------------------------------------------------------------------------------------------     14      25
-----------GCUGUGAGAUUGGUCACCGA--------------------------------------------------------------------------------------------------------------------     10      20
--------------------------------------------------------------------------------------------------------------------UCUCGUUGACUUAUCUCAGCA----------     5       21
-----------GCUGUGAGAUUGGUCACCGAGCU-----------------------------------------------------------------------------------------------------------------     2       23
--------------------------------------------------------------------------------------------------------------------------UGACUUAUCUCAGCACCCU------     1       19
----------UGCUGUGAGAUUGGUCACCGAGCUGA---------------------------------------------------------------------------------------------------------------     1       26
-----------GCUGUGAGAUUGGUCACCGAGCUG----------------------------------------------------------------------------------------------------------------     1       24
------------CUGUGAGAUUGGUCACCGAG-------------------------------------------------------------------------------------------------------------------     1       20
-------------UGUGAGAUUGGUCACCGAGC------------------------------------------------------------------------------------------------------------------     1       20
---------------UGAGAUUGGUCACCGAGC------------------------------------------------------------------------------------------------------------------     1       18
--------------------------------------------------------------------------------------------------------------------UCUCGUUGACUUAUCUCAG------------     1       19
Library = 1         Precision: [ total = 97.9% | 5p-arm = 98.0% | 3p-arm = 90.3% ]
----------UGCUGUGAGAUUGGUCACCGAGC------------------------------------------------------------------------------------------------------------------     2246    23
----------UGCUGUGAGAUUGGUCACCG---------------------------------------------------------------------------------------------------------------------     1687    20
----------UGCUGUGAGAUUGGUCACCGAG-------------------------------------------------------------------------------------------------------------------     865     22
----------UGCUGUGAGAUUGGUCACCGA--------------------------------------------------------------------------------------------------------------------     505     21
----------------------------------------------------------------------------------------------------------------------UCGUUGACUUAUCUCAGCACC--------     112     21
----------UGCUGUGAGAUUGGUCACC----------------------------------------------------------------------------------------------------------------------     69      19
----------UGCUGUGAGAUUGGUCAC-----------------------------------------------------------------------------------------------------------------------     57      18
-----------GCUGUGAGAUUGGUCACCGAGC------------------------------------------------------------------------------------------------------------------     29      22
------------CUGUGAGAUUGGUCACCGAGCUG----------------------------------------------------------------------------------------------------------------     25      23
-----------GCUGUGAGAUUGGUCACCGAG-------------------------------------------------------------------------------------------------------------------     23      21
----------UGCUGUGAGAUUGGUCACCGAGCU-----------------------------------------------------------------------------------------------------------------     18      24
-------------UGUGAGAUUGGUCACCGAGCUG----------------------------------------------------------------------------------------------------------------     11      22
---------------UGAGAUUGGUCACCGAGCUG----------------------------------------------------------------------------------------------------------------     8       20
--------------------------------------------------------------------------------------------------------------------UCUCGUUGACUUAUCUCAGC-----------     8       20
-----------GCUGUGAGAUUGGUCACCG---------------------------------------------------------------------------------------------------------------------     7       19
----------UGCUGUGAGAUUGGUCACCGAGCUG----------------------------------------------------------------------------------------------------------------     6       25
-----------GCUGUGAGAUUGGUCACCGA--------------------------------------------------------------------------------------------------------------------     6       20
--------------------------------------------------------------------------------------------------------------------UCUCGUUGACUUAUCUCAGCA----------     3       21
-----------GCUGUGAGAUUGGUCACCGAGCU-----------------------------------------------------------------------------------------------------------------     1       23
--------------------------------------------------------------------------------------------------------------------------UGACUUAUCUCAGCACCCU------     1       19
Library = 2         Precision: [ total = 97.6% | 5p-arm = 97.8% | 3p-arm = 87.5% ]
----------UGCUGUGAGAUUGGUCACCGAGC------------------------------------------------------------------------------------------------------------------     1762    23
----------UGCUGUGAGAUUGGUCACCG---------------------------------------------------------------------------------------------------------------------     1553    20
----------UGCUGUGAGAUUGGUCACCGAG-------------------------------------------------------------------------------------------------------------------     674     22
----------UGCUGUGAGAUUGGUCACCGA--------------------------------------------------------------------------------------------------------------------     438     21
----------------------------------------------------------------------------------------------------------------------UCGUUGACUUAUCUCAGCACC--------     77      21
```

A. subglobosum asu-mir-1208 continued

```
(((...((.(((((..(((((.(((((.((((.((.((((.....((((((((..(((((....)))))))))))))...((((.((((...........)))).)))))))).)).)))).))))).)))))))))).))..))).   -68.63 kcal/mol
GCGACUAGUGuGCUGUGAGAUUGGUCACCGAGcUGAUAGUAAGAAGGAGGUUGACGCACUGUAGAGUGCCAGUCUCCAACAUAACCAACAUUUAAUCUCAGUUGUUUAUACUACCAUCuCGUUGACUUAUCUCAGCACcCUUACGCG   count   length
----------UGCUGUGAGAUUGGUCAC-----------------------------------------------------------------------------------------------------------------------   68      18
----------UGCUGUGAGAUUGGUCACC----------------------------------------------------------------------------------------------------------------------   61      19
-----------GCUGUGAGAUUGGUCACCGAGC------------------------------------------------------------------------------------------------------------------   38      22
-----------GCUGUGAGAUUGGUCACCGAG-------------------------------------------------------------------------------------------------------------------   22      21
------------CUGUGAGAUUGGUCACCGAGCUG----------------------------------------------------------------------------------------------------------------   13      23
---------------UGAGAUUGGUCACCGAGCUG----------------------------------------------------------------------------------------------------------------   10      20
--------------------------------------------------------------------------------------------------------------------UCUCGUUGACUUAUCUCAGC-----------   8       20
----------UGCUGUGAGAUUGGUCACCGAGCU-----------------------------------------------------------------------------------------------------------------   7       24
----------UGCUGUGAGAUUGGUCACCGAGCUG----------------------------------------------------------------------------------------------------------------   6       25
-----------GCUGUGAGAUUGGUCACCG---------------------------------------------------------------------------------------------------------------------   5       19
-------------UGUGAGAUUGGUCACCGAGCUG----------------------------------------------------------------------------------------------------------------   5       22
-----------GCUGUGAGAUUGGUCACCGA--------------------------------------------------------------------------------------------------------------------   3       20
--------------------------------------------------------------------------------------------------------------------UCUCGUUGACUUAUCUCAGCA----------   2       21
----------UGCUGUGAGAUUGGUCACCGAGCUGA---------------------------------------------------------------------------------------------------------------   1       26
-----------GCUGUGAGAUUGGUCACCGAGCU-----------------------------------------------------------------------------------------------------------------   1       23
-----------GCUGUGAGAUUGGUCACCGAGCUG----------------------------------------------------------------------------------------------------------------   1       24
------------CUGUGAGAUUGGUCACCGAG-------------------------------------------------------------------------------------------------------------------   1       20
-------------UGUGAGAUUGGUCACCGAGC------------------------------------------------------------------------------------------------------------------   1       20
--------------------------------------------------------------------------------------------------------------------UCUCGUUGACUUAUCUCAG------------   1       19
Library = 3        Precision: [ total = 97.7% | 5p-arm = 97.8% | 3p-arm = 95.7% ]
----------UGCUGUGAGAUUGGUCACCGAGC------------------------------------------------------------------------------------------------------------------   485     23
----------UGCUGUGAGAUUGGUCACCG---------------------------------------------------------------------------------------------------------------------   204     20
----------UGCUGUGAGAUUGGUCACCGAG-------------------------------------------------------------------------------------------------------------------   185     22
----------UGCUGUGAGAUUGGUCACCGA--------------------------------------------------------------------------------------------------------------------   82      21
----------------------------------------------------------------------------------------------------------------------UCGUUGACUUAUCUCAGCACC--------   22      21
-----------GCUGUGAGAUUGGUCACCGAGC------------------------------------------------------------------------------------------------------------------   11      22
----------UGCUGUGAGAUUGGUCAC-----------------------------------------------------------------------------------------------------------------------   7       18
----------UGCUGUGAGAUUGGUCACC----------------------------------------------------------------------------------------------------------------------   5       19
------------CUGUGAGAUUGGUCACCGAGCUG----------------------------------------------------------------------------------------------------------------   5       23
----------UGCUGUGAGAUUGGUCACCGAGCU-----------------------------------------------------------------------------------------------------------------   4       24
----------UGCUGUGAGAUUGGUCACCGAGCUG----------------------------------------------------------------------------------------------------------------   2       25
-----------GCUGUGAGAUUGGUCACCGAG-------------------------------------------------------------------------------------------------------------------   2       21
-----------GCUGUGAGAUUGGUCACCG---------------------------------------------------------------------------------------------------------------------   2       19
-----------GCUGUGAGAUUGGUCACCGA--------------------------------------------------------------------------------------------------------------------   1       20
---------------UGAGAUUGGUCACCGAGC------------------------------------------------------------------------------------------------------------------   1       18
--------------------------------------------------------------------------------------------------------------------UCUCGUUGACUUAUCUCAGC-----------   1       20
```

```
    ACU  U     GU     U     C    c  A    AAGAA         AC    G
 GCG   AG GuGCU  GAGAU GGUCA CGAG UG UAGU     GGAGGUUG  GCACU U
 CGC   UC CACGA--CUCUA UCAGU GCuC AC AUCA-\ /CCUCUGAC--CGUGA A
G   AU-  c            U     U    U  C      UA                G
                                           AU C    AUUUA
                                           UAA CAAC     A
                                           \-U GUUG     /
                                              U    ACUCU
```

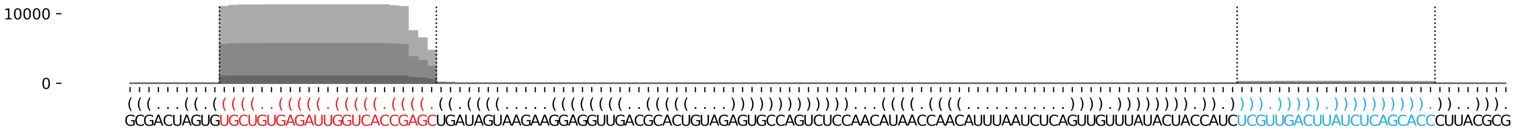

A. subglobosum asu-mir-1209

```
......(((...((((((((((((((((((((((((((.((((.(((.......((.((((((((...)))))))))))))..)))).))).)))))))))))))))))))))))...)))......  -73.19 kcal/mol
AUUGUUCAUUuGAACGAUUUUCACCAAAAUcAGGGAAUGGUCACUGGUUACUCAAGAUGUUCGGUCCGACCGAACACUCCGAUUGACGAUUCCCCUGAuUUUGGUGAAAAUCGUUAAUaUGUCGUAU  count   length
Libraries combined          Precision: [ total = 99.9% | 5p-arm = 99.9% | 3p-arm = 82.4% ]
----------UGAACGAUUUUCACCAAAAUC------------------------------------------------------------------------------------------------  19394   21
----------UGAACGAUUUUCACCAAAAU-------------------------------------------------------------------------------------------------  555     20
----------UGAACGAUUUUCACCAAAAUCA-----------------------------------------------------------------------------------------------  11      22
--------------------------------------------------------------------------------------------------UUUUGGUGAAAAUCGUUAAUA--------  14      21
-----------GAACGAUUUUCACCAAAAUC------------------------------------------------------------------------------------------------  14      20
--------UUUGAACGAUUUUCACCAAAAU-------------------------------------------------------------------------------------------------  1       22
---------UUGAACGAUUUUCACCAAAAUC------------------------------------------------------------------------------------------------  1       22
----------UGAACGAUUUUCACCAAAA--------------------------------------------------------------------------------------------------  4       19
-------------------------------AGGGAAUGGUCACUGGUUACUCAAG-----------------------------------------------------------------------  1       25
-------------------------------AGGGAAUGGUCACUGGUUACUC--------------------------------------------------------------------------  1       22
----------------------------------------------------CAAGAUGUUCGGUCCGACCGA------------------------------------------------------  1       21
--------------------------------------------------------------------------------------CGAUUCCCCUGAUUUUGGU----------------------  1       19
--------UUUGAACGAUUUUCACCAAAA--------------------------------------------------------------------------------------------------  1       21
---------UUGAACGAUUUUCACCAAAAU-------------------------------------------------------------------------------------------------  1       21
-------------------------------AGGGAAUGGUCACUGGUUACUCAAGAUGUUC-----------------------------------------------------------------  4       31
-------------------------------------------------------------CGGUCCGACCGAACACUCCGA---------------------------------------------  1       21
---------------------------------------------------------------------------------------------------UUUGGUGAAAAUCGUUAAUA--------  1       20
Library = 1         Precision: [ total = 99.9% | 5p-arm = 99.9% | 3p-arm = 85.7% ]
----------UGAACGAUUUUCACCAAAAUC------------------------------------------------------------------------------------------------  9493    21
----------UGAACGAUUUUCACCAAAAU-------------------------------------------------------------------------------------------------  276     20
----------UGAACGAUUUUCACCAAAAUCA-----------------------------------------------------------------------------------------------  6       22
--------------------------------------------------------------------------------------------------UUUUGGUGAAAAUCGUUAAUA--------  6       21
-----------GAACGAUUUUCACCAAAAUC------------------------------------------------------------------------------------------------  5       20
--------UUUGAACGAUUUUCACCAAAAU-------------------------------------------------------------------------------------------------  1       22
---------UUGAACGAUUUUCACCAAAAUC------------------------------------------------------------------------------------------------  1       22
----------UGAACGAUUUUCACCAAAA--------------------------------------------------------------------------------------------------  1       19
-------------------------------AGGGAAUGGUCACUGGUUACUCAAG-----------------------------------------------------------------------  1       25
-------------------------------AGGGAAUGGUCACUGGUUACUC--------------------------------------------------------------------------  1       22
----------------------------------------------------CAAGAUGUUCGGUCCGACCGA------------------------------------------------------  1       21
--------------------------------------------------------------------------------------CGAUUCCCCUGAUUUUGGU----------------------  1       19
Library = 2         Precision: [ total = 99.8% | 5p-arm = 99.8% | 3p-arm = 88.9% ]
----------UGAACGAUUUUCACCAAAAUC------------------------------------------------------------------------------------------------  8160    21
----------UGAACGAUUUUCACCAAAAU-------------------------------------------------------------------------------------------------  253     20
-----------GAACGAUUUUCACCAAAAUC------------------------------------------------------------------------------------------------  9       20
--------------------------------------------------------------------------------------------------UUUUGGUGAAAAUCGUUAAUA--------  8       21
----------UGAACGAUUUUCACCAAAAUCA-----------------------------------------------------------------------------------------------  4       22
-------------------------------AGGGAAUGGUCACUGGUUACUCAAGAUGUUC-----------------------------------------------------------------  3       31
---------UUGAACGAUUUUCACCAAAAU-------------------------------------------------------------------------------------------------  1       21
----------UGAACGAUUUUCACCAAAA--------------------------------------------------------------------------------------------------  1       19
-------------------------------------------------------------CGGUCCGACCGAACACUCCGA---------------------------------------------  1       21
Library = 3         Precision: [ total = 99.8% | 5p-arm = 99.9% | 3p-arm = 0% ]
----------UGAACGAUUUUCACCAAAAUC------------------------------------------------------------------------------------------------  1741    21
----------UGAACGAUUUUCACCAAAAU-------------------------------------------------------------------------------------------------  26      20
----------UGAACGAUUUUCACCAAAA--------------------------------------------------------------------------------------------------  2       19
--------UUUGAACGAUUUUCACCAAAA--------------------------------------------------------------------------------------------------  1       21
----------UGAACGAUUUUCACCAAAAUCA-----------------------------------------------------------------------------------------------  1       22
-------------------------------AGGGAAUGGUCACUGGUUACUCAAGAUGUUC-----------------------------------------------------------------  1       31
---------------------------------------------------------------------------------------------------UUUGGUGAAAAUCGUUAAUA--------  1       20
```

```
AUUGUU   UuG                          G    C    UUACUCA  A         C
      CAU   AACGAUUUUCACCAAAAUcAGGG-AAU GUCA -UGG       AG UGUUCGGU C
      GUa   UUGCUAAAAGUGGUUUuAGUCCC UUA CAGU  GCC-------UC-ACAAGCCA /
UAUGCU   UAA                      C   G    UA                     G
```

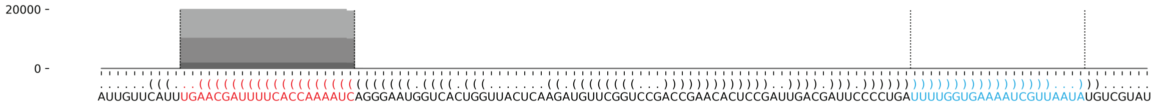

A. subglobosum asu-mir-1210

```
(((((((.(..((((.((((((((((.(((((..((((((((((.......((((((((...)))))))).....))))))))))..)))))...)))))))))).))))..).)))))))    -76.03 kcal/mol
GAUGAUGCGUuGAGCCGUACCAUUCUCUGUcUCUGUACAGAGUGCCCAUCUGGCAAGUGGACUACUUGCCCAUUACACUCUGUACUUGGAcACUUAGGAUGGUGCUGCUUCUcACAUUAUU    count  length
Libraries combined          Precision: [ total = 95.8% | 5p-arm = 96.2% | 3p-arm = 95.4% ]
----------UGAGCCGUACCAUUCUCUGUCU-----------------------------------------------------------------------------------------    116    22
------------------------------------------------------------------------------------------CACUUAGGAUGGUGCUGCUUC----------    92     21
------------------------------------------------------------------------------------------CACUUAGGAUGGUGCUGCUU-----------    59     20
----------UGAGCCGUACCAUUCUCUGUC------------------------------------------------------------------------------------------    67     21
------------------------------------------------------------------------------------------CACUUAGGAUGGUGCUGCUUCU---------    26     22
------------------------------------------------------------------------------------------CACUUAGGAUGGUGCUGCUUCUC--------    18     23
----------UGAGCCGUACCAUUCUCUG--------------------------------------------------------------------------------------------    16     19
------------------------------------------------------------------------------------------CACUUAGGAUGGUGCUGCU------------    9      19
------------AGCCGUACCAUUCUCUGUC------------------------------------------------------------------------------------------    3      19
------------AGCCGUACCAUUCUCUGUCU-----------------------------------------------------------------------------------------    5      20
----------UGAGCCGUACCAUUCUCUGU-------------------------------------------------------------------------------------------    4      20
--------------------------------------------------------------------------------UGUACUUGGACACUUAGGAUGG-------------------    6      22
-------------------------------------------------------------------------------CUGUACUUGGACACUUAGGAU---------------------    1      21
------------------------------------------------------------------------------------------CACUUAGGAUGGUGCUGC-------------    3      18
-------------------------------------------------------------------------------------------ACUUAGGAUGGUGCUGCUUC----------    1      20
--------------------------------------------------------------------------------UGUACUUGGACACUUAGGAUG--------------------    1      21
--------------------------------------------------------------------------------------------CUUAGGAUGGUGCUGCUUCUC--------    1      21
Library = 1         Precision: [ total = 95.1% | 5p-arm = 94.0% | 3p-arm = 96.2% ]
----------UGAGCCGUACCAUUCUCUGUCU-----------------------------------------------------------------------------------------    51     22
------------------------------------------------------------------------------------------CACUUAGGAUGGUGCUGCUUC----------    41     21
------------------------------------------------------------------------------------------CACUUAGGAUGGUGCUGCUU-----------    36     20
----------UGAGCCGUACCAUUCUCUGUC------------------------------------------------------------------------------------------    34     21
------------------------------------------------------------------------------------------CACUUAGGAUGGUGCUGCUUCU---------    9      22
------------------------------------------------------------------------------------------CACUUAGGAUGGUGCUGCUUCUC--------    9      23
----------UGAGCCGUACCAUUCUCUG--------------------------------------------------------------------------------------------    7      19
------------------------------------------------------------------------------------------CACUUAGGAUGGUGCUGCU------------    4      19
------------AGCCGUACCAUUCUCUGUC------------------------------------------------------------------------------------------    3      19
------------AGCCGUACCAUUCUCUGUCU-----------------------------------------------------------------------------------------    3      20
----------UGAGCCGUACCAUUCUCUGU-------------------------------------------------------------------------------------------    2      20
--------------------------------------------------------------------------------UGUACUUGGACACUUAGGAUGG-------------------    2      22
-------------------------------------------------------------------------------CUGUACUUGGACACUUAGGAU---------------------    1      21
------------------------------------------------------------------------------------------CACUUAGGAUGGUGCUGC-------------    1      18
-------------------------------------------------------------------------------------------ACUUAGGAUGGUGCUGCUUC----------    1      20
Library = 2         Precision: [ total = 96.0% | 5p-arm = 97.6% | 3p-arm = 94.4% ]
----------UGAGCCGUACCAUUCUCUGUCU-----------------------------------------------------------------------------------------    51     22
------------------------------------------------------------------------------------------CACUUAGGAUGGUGCUGCUUC----------    38     21
----------UGAGCCGUACCAUUCUCUGUC------------------------------------------------------------------------------------------    24     21
------------------------------------------------------------------------------------------CACUUAGGAUGGUGCUGCUU-----------    19     20
------------------------------------------------------------------------------------------CACUUAGGAUGGUGCUGCUUCU---------    13     22
------------------------------------------------------------------------------------------CACUUAGGAUGGUGCUGCUUCUC--------    7      23
----------UGAGCCGUACCAUUCUCUG--------------------------------------------------------------------------------------------    6      19
------------------------------------------------------------------------------------------CACUUAGGAUGGUGCUGCU------------    5      19
--------------------------------------------------------------------------------UGUACUUGGACACUUAGGAUGG-------------------    4      22
----------UGAGCCGUACCAUUCUCUGU-------------------------------------------------------------------------------------------    2      20
------------AGCCGUACCAUUCUCUGUCU-----------------------------------------------------------------------------------------    2      20
------------------------------------------------------------------------------------------CACUUAGGAUGGUGCUGC-------------    2      18
--------------------------------------------------------------------------------------------CUUAGGAUGGUGCUGCUUCUC--------    1      21
Library = 3         Precision: [ total = 98.0% | 5p-arm = 100.0% | 3p-arm = 95.8% ]
----------UGAGCCGUACCAUUCUCUGUCU-----------------------------------------------------------------------------------------    14     22
------------------------------------------------------------------------------------------CACUUAGGAUGGUGCUGCUUC----------    13     21
----------UGAGCCGUACCAUUCUCUGUC------------------------------------------------------------------------------------------    9      21
------------------------------------------------------------------------------------------CACUUAGGAUGGUGCUGCUU-----------    4      20
------------------------------------------------------------------------------------------CACUUAGGAUGGUGCUGCUUCU---------    4      22
```

A. subglobosum asu-mir-1210 continued

```
((((((.(..((((.((((((((((.(((((..((((((((((.......((((((((...))))))))).....))))))))))..)))))...))))))))))).)))).).)))))))      -76.03 kcal/mol
GAUGAUGCGUuGAGCCGUACCAUUCUCUGUcUCUGUACAGAGUGCCCAUCUGGCAAGUGGACUACUUGCCCAUUACACUCUGUACUUGGAcACUUAGGAUGGUGCUGCUUCUcACAUUAUU    count  length
----------UGAGCCGUACCAUUCUCUG--------------------------------------------------------------------------------------------    3      19
------------------------------------------------------------------------------------------CACUUAGGAUGGUGCUGCUUCUC--------    2      23
--------------------------------------------------------------------------------UGUACUUGGACACUUAGGAUG--------------------    1      21
```

```
       C  Uu    C          C        CU          CCCAUCU       G
GAUGAUG G  GAGC GUACCAUUCU --UGUcU  GUACAGAGUG         GGCAAGUG A
UUAUUAC c  UUCG CGUGGUAGGA    AcAGG  CAUGUCUCAC       --CCGUUCAU /
       A UC     U          UUC      UU          AUUAC         C
```

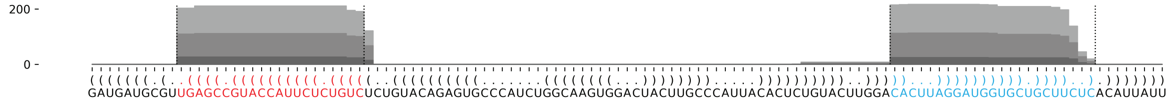

A. subglobosum asu-mir-1211

```
....(((((((((..(((.(((((((((((((((.(((((((((((.(.......).)).)))))))))).))))))))))))))))...))))))))))....      -55.45 kcal/mol
UCAUUAAGAAcCCGAUACAUAUGUCAUAUUGaUAUAAUGAAUAGGACGACUUUAACUUCAUUGUUCAUUCUAUCaAUAUGACAUUGUUAACGGGUuCUUACUUG      count  length
Libraries combined         Precision: [ total = 99.9% | 5p-arm = 66.7% | 3p-arm = 99.9% ]
--------------------------------------------------------------------------AAUAUGACAUUGUUAACGGGU---------      3469   21
--------------------------------------------------------------------------AAUAUGACAUUGUUAACGGG----------      152    20
--------------------------------------------------------------------------AAUAUGACAUUGUUAACGGGUU--------      161    22
--------------------------------------------------------------------------AAUAUGACAUUGUUAACGG-----------      43     19
--------------------------------------------------------------------------AAUAUGACAUUGUUAACG------------      5      18
----------CCCGAUACAUAUGUCAUAUUG-------------------------------------------------------------------------      4      21
-----------CCGAUACAUAUGUCAUAUUGA------------------------------------------------------------------------      2      21
-------------------------------------------------------------------------CAAUAUGACAUUGUUAACGGGU---------      1      22
--------------------------------------------------------------------------AAUAUGACAUUGUUAACGGGUUC-------      1      23
---------------------------------------------------------------------------AUAUGACAUUGUUAACGGGU---------      1      20
Library = 1        Precision: [ total = 99.9% | 5p-arm = 50.0% | 3p-arm = 100.0% ]
--------------------------------------------------------------------------AAUAUGACAUUGUUAACGGGU---------      1677   21
--------------------------------------------------------------------------AAUAUGACAUUGUUAACGGG----------      81     20
--------------------------------------------------------------------------AAUAUGACAUUGUUAACGGGUU--------      79     22
--------------------------------------------------------------------------AAUAUGACAUUGUUAACGG-----------      21     19
--------------------------------------------------------------------------AAUAUGACAUUGUUAACG------------      3      18
----------CCCGAUACAUAUGUCAUAUUG-------------------------------------------------------------------------      1      21
-----------CCGAUACAUAUGUCAUAUUGA------------------------------------------------------------------------      1      21
Library = 2        Precision: [ total = 99.9% | 5p-arm = 75.0% | 3p-arm = 100.0% ]
--------------------------------------------------------------------------AAUAUGACAUUGUUAACGGGU---------      1490   21
--------------------------------------------------------------------------AAUAUGACAUUGUUAACGGGUU--------      74     22
--------------------------------------------------------------------------AAUAUGACAUUGUUAACGGG----------      62     20
--------------------------------------------------------------------------AAUAUGACAUUGUUAACGG-----------      20     19
----------CCCGAUACAUAUGUCAUAUUG-------------------------------------------------------------------------      3      21
--------------------------------------------------------------------------AAUAUGACAUUGUUAACG------------      2      18
-----------CCGAUACAUAUGUCAUAUUGA------------------------------------------------------------------------      1      21
--------------------------------------------------------------------------AAUAUGACAUUGUUAACGGGUUC-------      1      23
Library = 3        Precision: [ total = 99.4% | 5p-arm = 0% | 3p-arm = 99.4% ]
--------------------------------------------------------------------------AAUAUGACAUUGUUAACGGGU---------      302    21
--------------------------------------------------------------------------AAUAUGACAUUGUUAACGGG----------      9      20
--------------------------------------------------------------------------AAUAUGACAUUGUUAACGGGUU--------      8      22
--------------------------------------------------------------------------AAUAUGACAUUGUUAACGG-----------      2      19
-------------------------------------------------------------------------CAAUAUGACAUUGUUAACGGGU---------      1      22
---------------------------------------------------------------------------AUAUGACAUUGUUAACGGGU---------      1      20
```

```
UCAU          AU   U              U            C ACU
    UAAGAAcCCG  ACA AUGUCAUAUUGaUA AAUGAAUAG-GA G   U
    AUUCuUGGGC   UGUUACAGUAUAaCUAU UUACUUGUU CU C   /
GUUC          AAU                 C          A  U AAU
```

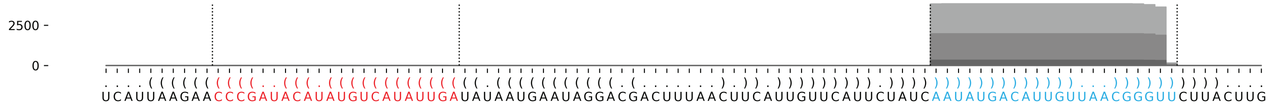

A. subglobosum asu-mir-1212

```
..((((.((((.(((((..(((((.((((((((.(((((..(((((.............))))).........."))))))))))))).)))))..))))).)))).))))..
```

```
..((((.((((.(((((..(((((.((((((((.(((((..(((((.............)))))..........))))))))))))).)))))..))))).)))).))))..   -51.1 kcal/mol
GUAUAUAUCAuUCAGUAUUCCAUCUGUGAUgUAUAUUGGUGCAGUCACAUUUCUGAUCGACUGGCCAUACUUCUCCAAUUACAuCACCGAUGGCCUACUGCAUgAGAUGUUC   count   length
Libraries combined         Precision: [ total = 99.4% | 5p-arm = 99.7% | 3p-arm = 99.1% ]
-----------------------------------------------------------------------------------UCACCGAUGGCCUACUGCAUG--------   962   21
----------UUCAGUAUUCCAUCUGUGAUG---------------------------------------------------------------------------------   916   21
-----------------------------------------------------------------------------------UCACCGAUGGCCUACUGCA----------   23    19
-----------------------------------------------------------------------------------UCACCGAUGGCCUACUGCAU---------   15    20
-------------------------------------------------------------------------------------ACCGAUGGCCUACUGCAUGAG------   3     21
----------UUCAGUAUUCCAUCUGUGA-----------------------------------------------------------------------------------   2     19
-----------UCAGUAUUCCAUCUGUGAUG---------------------------------------------------------------------------------   2     20
----------------AUUCCAUCUGUGAUGUAUAUUG--------------------------------------------------------------------------   1     22
---------------------------------------------------------------------------------CAUCACCGAUGGCCUACUG------------   1     19
---------------------------------------------------------------------------------CAUCACCGAUGGCCUACUGCA----------   1     21
------------------------------------------------------------------------------------------UGGCCUACUGCAUGAGAUGUUC   1     22
-----------------------------------------------------UGAUCGACUGGCCAUACUUCU--------------------------------------   1     21
------------------------------------------------------------------------------UUACAUCACCGAUGGCCUACU-------------   1     21
-----------------------------------------------------------------------------------UCACCGAUGGCCUACUGCAUGAGAUGU--   1     27
-----------------------------------------------------------------------------------UCACCGAUGGCCUACUGC-----------   1     18
-----------------------------------------------------------------------------------UCACCGAUGGCCUACUGCAUGA-------   1     22
Library = 1        Precision: [ total = 99.3% | 5p-arm = 99.6% | 3p-arm = 99.0% ]
-----------------------------------------------------------------------------------UCACCGAUGGCCUACUGCAUG--------   474   21
----------UUCAGUAUUCCAUCUGUGAUG---------------------------------------------------------------------------------   449   21
-----------------------------------------------------------------------------------UCACCGAUGGCCUACUGCA----------   11    19
-----------------------------------------------------------------------------------UCACCGAUGGCCUACUGCAU---------   8     20
-------------------------------------------------------------------------------------ACCGAUGGCCUACUGCAUGAG------   2     21
----------UUCAGUAUUCCAUCUGUGA-----------------------------------------------------------------------------------   1     19
-----------UCAGUAUUCCAUCUGUGAUG---------------------------------------------------------------------------------   1     20
----------------AUUCCAUCUGUGAUGUAUAUUG--------------------------------------------------------------------------   1     22
---------------------------------------------------------------------------------CAUCACCGAUGGCCUACUG------------   1     19
---------------------------------------------------------------------------------CAUCACCGAUGGCCUACUGCA----------   1     21
------------------------------------------------------------------------------------------UGGCCUACUGCAUGAGAUGUUC   1     22
Library = 2        Precision: [ total = 99.4% | 5p-arm = 99.8% | 3p-arm = 99.0% ]
----------UUCAGUAUUCCAUCUGUGAUG---------------------------------------------------------------------------------   398   21
-----------------------------------------------------------------------------------UCACCGAUGGCCUACUGCAUG--------   398   21
-----------------------------------------------------------------------------------UCACCGAUGGCCUACUGCA----------   12    19
-----------------------------------------------------------------------------------UCACCGAUGGCCUACUGCAU---------   5     20
----------UUCAGUAUUCCAUCUGUGA-----------------------------------------------------------------------------------   1     19
-----------UCAGUAUUCCAUCUGUGAUG---------------------------------------------------------------------------------   1     20
-----------------------------------------------------UGAUCGACUGGCCAUACUUCU--------------------------------------   1     21
------------------------------------------------------------------------------UUACAUCACCGAUGGCCUACU-------------   1     21
-----------------------------------------------------------------------------------UCACCGAUGGCCUACUGCAUGAGAUGU--   1     27
-----------------------------------------------------------------------------------UCACCGAUGGCCUACUGC-----------   1     18
-----------------------------------------------------------------------------------UCACCGAUGGCCUACUGCAUGA-------   1     22
-------------------------------------------------------------------------------------ACCGAUGGCCUACUGCAUGAG------   1     21
Library = 3        Precision: [ total = 100.0% | 5p-arm = 100.0% | 3p-arm = 100.0% ]
-----------------------------------------------------------------------------------UCACCGAUGGCCUACUGCAUG--------   90    21
----------UUCAGUAUUCCAUCUGUGAUG---------------------------------------------------------------------------------   69    21
-----------------------------------------------------------------------------------UCACCGAUGGCCUACUGCAU---------   2     20
```

```
GU    A    U     UU     U         U      UG---------      ACAUU
  AUAU UCAu CAGUA  CCAUC GUGAUgUA AUUGG               CAGUC     U
  UGUA AgUA GUCAU  GGUAG CACuACAU-UAACC               GUCAG     C
CU    G    C     CC     C                UCUUCAUACCG      CUAGU
```

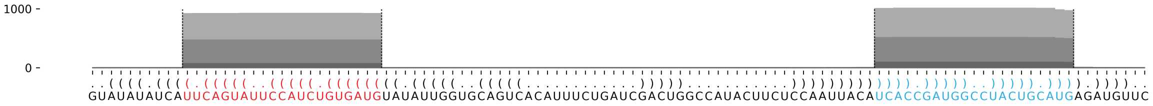

A. subglobosum asu-mir-1213

```
.....((((.(((((((((((((((((((((..(((((.(.(((.............))).).))))).)))))))))))))))))))))).))))).....      -67.09 kcal/mol
AGUCAGGACAaCAGUCUAACAUGGUAGCAUuAUACCAAACUAUUGCGAGUAACGAAGGGCAUUCGUUUGUAAUAAuGCUACCAUGUUAGACUGAUgUCCAAUCU      count    length
Libraries combined          Precision: [ total = 96.7% | 5p-arm = 91.9% | 3p-arm = 97.9% ]
---------------------------------------------------------------------------UGCUACCAUGUUAGACUGAUG--------      15455    21
----------ACAGUCUAACAUGGUAGCAUU-------------------------------------------------------------------------      3701     21
---------------------------------------------------------------------------UGCUACCAUGUUAGACUGA----------      321      19
--------------------------------------------------------------------------AUGCUACCAUGUUAGACUGAU---------      222      21
---------------------------------------------------------------------------UGCUACCAUGUUAGACUGAUGU-------      220      22
---------AACAGUCUAACAUGGUAGCAUU-------------------------------------------------------------------------      171      22
---------------------------------------------------------------------------UGCUACCAUGUUAGACUGAU---------      147      20
----------------------------------------------------------------------------GCUACCAUGUUAGACUGAUG--------      67       20
---------AACAGUCUAACAUGGUAGCAU--------------------------------------------------------------------------      54       21
-----------CAGUCUAACAUGGUAGCAUUA------------------------------------------------------------------------      47       21
-----------CAGUCUAACAUGGUAGCAUU-------------------------------------------------------------------------      28       20
----------ACAGUCUAACAUGGUAGCAU--------------------------------------------------------------------------      17       20
---------------------------------------------------------------------------UGCUACCAUGUUAGACUG-----------      28       18
----------------------------------------------------------------------------GCUACCAUGUUAGACUGAUGU-------      24       21
-----------------------------------------------------------------------------CUACCAUGUUAGACUGAUGUC------      13       21
---------AACAGUCUAACAUGGUAGC----------------------------------------------------------------------------      9        19
--------------------------------------------------------------------------AUGCUACCAUGUUAGACUGA----------      4        20
---------AACAGUCUAACAUGGUAGCAUUA------------------------------------------------------------------------      2        23
-----------CAGUCUAACAUGGUAGCAUUAU-----------------------------------------------------------------------      2        22
--------------------------------------------------------------------------AUGCUACCAUGUUAGACU------------      3        18
------------------------------------------------------------------------------UACCAUGUUAGACUGAUG--------      4        18
--------CAACAGUCUAACAUGGUAGCA---------------------------------------------------------------------------      2        21
---------AACAGUCUAACAUGGUAGCA---------------------------------------------------------------------------      8        20
----------ACAGUCUAACAUGGUAGCA---------------------------------------------------------------------------      3        19
----------ACAGUCUAACAUGGUAGCAUUA------------------------------------------------------------------------      7        22
------------AGUCUAACAUGGUAGCAUU-------------------------------------------------------------------------      3        19
-------------------------------------------------------------------------AAUGCUACCAUGUUAGACUGA----------      1        21
---------------------------------------------------------------------------UGCUACCAUGUUAGACUGAUGUC------      3        23
---------------------------------------------------------------------------UGCUACCAUGUUAGACUGAUGUCC-----      1        24
---------------------------------------------------------------------------UGCUACCAUGUUAGACUGAUGUCCA----      1        25
----------------------------------------------------------------------------GCUACCAUGUUAGACUGA----------      2        18
----------------------------------------------------------------------------GCUACCAUGUUAGACUGAU---------      2        19
-----------------------------------------------------------------------------CUACCAUGUUAGACUGAUG--------      2        19
------------------------------------------------------------------------------UACCAUGUUAGACUGAUGUCCAA---      1        23
------------------------------------------------------------------------------UACCAUGUUAGACUGAUGUCC-----      3        21
--------CAACAGUCUAACAUGGUAGC----------------------------------------------------------------------------      1        20
----------ACAGUCUAACAUGGUAGC----------------------------------------------------------------------------      2        18
```

```
AGUCA     a                      AC     U U   GAGUA
     GGACA CAGUCUAACAUGGUAGCAUuAU  CAAAC A UGC     A
     CCUgU GUCAGAUUGUACCAUCGuAAUA  GUUUG U ACG     C
UCUAA     A                      AU     C U   GGAAG
```

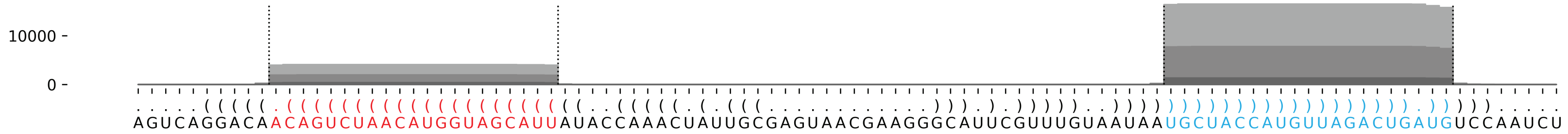

A. subglobosum asu-mir-1214

```
.(((.((.(((((((((((....((((((((((((.((((.((((..(((....))).)))))))).)))))))))))).....))))))))))).)).))).         -39.78 kcal/mol
CAUUUGUUUUaCAAAUGUUGAACAGAUAAUaAAUAUAUGAAGUAAAGAUACAAAUAUAUUGCUCGUUUAUUUAuUAUCUAUUGAAACAUUUGUGaUACCAGUU          count    length
Libraries combined          Precision: [ total = 99.5% | 5p-arm = 99.7% | 3p-arm = 98.7% ]
----------ACAAAUGUUGAACAGAUAAUA------------------------------------------------------------------------          322      21
-------------------------------------------------------------------------UUAUCUAUUGAAACAUUUGUGA--------          66       22
-------------------------------------------------------------------------UUAUCUAUUGAAACAUUUG-----------          5        19
----------ACAAAUGUUGAACAGAUAAU-------------------------------------------------------------------------          6        20
------------AAAUGUUGAACAGAUAAUA------------------------------------------------------------------------          1        19
-------------------------------------------------------------------------UUAUCUAUUGAAACAUUUGUGAU-------          4        23
----------ACAAAUGUUGAACAGAUAA--------------------------------------------------------------------------          1        19
-------------------------------------------------------------------------UUAUCUAUUGAAACAUUUGU----------          1        20
--------------------------------------------------------------------------UAUCUAUUGAAACAUUUGUGA--------          1        21
Library = 1        Precision: [ total = 99.5% | 5p-arm = 99.4% | 3p-arm = 100.0% ]
----------ACAAAUGUUGAACAGAUAAUA------------------------------------------------------------------------          157      21
-------------------------------------------------------------------------UUAUCUAUUGAAACAUUUGUGA--------          32       22
-------------------------------------------------------------------------UUAUCUAUUGAAACAUUUG-----------          4        19
----------ACAAAUGUUGAACAGAUAAU-------------------------------------------------------------------------          3        20
------------AAAUGUUGAACAGAUAAUA------------------------------------------------------------------------          1        19
-------------------------------------------------------------------------UUAUCUAUUGAAACAUUUGUGAU-------          1        23
Library = 2        Precision: [ total = 99.4% | 5p-arm = 100.0% | 3p-arm = 97.1% ]
----------ACAAAUGUUGAACAGAUAAUA------------------------------------------------------------------------          130      21
-------------------------------------------------------------------------UUAUCUAUUGAAACAUUUGUGA--------          30       22
----------ACAAAUGUUGAACAGAUAAU-------------------------------------------------------------------------          2        20
-------------------------------------------------------------------------UUAUCUAUUGAAACAUUUGUGAU-------          2        23
----------ACAAAUGUUGAACAGAUAA--------------------------------------------------------------------------          1        19
-------------------------------------------------------------------------UUAUCUAUUGAAACAUUUG-----------          1        19
-------------------------------------------------------------------------UUAUCUAUUGAAACAUUUGU----------          1        20
--------------------------------------------------------------------------UAUCUAUUGAAACAUUUGUGA--------          1        21
Library = 3        Precision: [ total = 100.0% | 5p-arm = 100.0% | 3p-arm = 100.0% ]
----------ACAAAUGUUGAACAGAUAAUA------------------------------------------------------------------------          35       21
-------------------------------------------------------------------------UUAUCUAUUGAAACAUUUGUGA--------          4        22
----------ACAAAUGUUGAACAGAUAAU-------------------------------------------------------------------------          1        20
-------------------------------------------------------------------------UUAUCUAUUGAAACAUUUGUGAU-------          1        23
```

```
C   U  U                 GAAC-            U    A    AG   C
 AUU GU UUaCAAAUGUU      AGAUAAUaAAUA AUGA GUAA  AUA A
 UGA CA aGUGUUUACAA      UCUAUuAUUUAU UGCU-CGUU -UAU A
U   C  U                 AGUUA            U         A    A
```

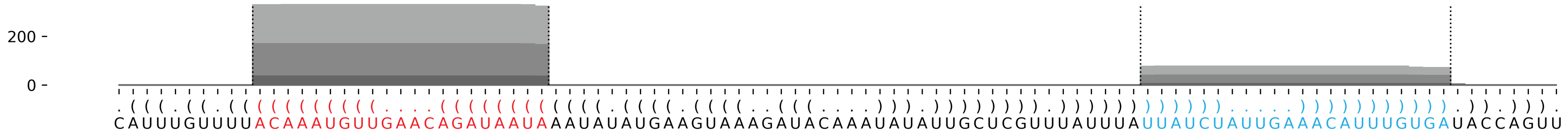

A. subglobosum asu-mir-1215

```
((((((((((((((((((.((((((((((((((((((((((((((((.((((.(((.((.......((((......))))......)).))).))))((((((((((.((((.........)))).)))......)))).))))))))))))))))))))))))))))))).))))))))))))))))))   -129.41 kcal/mol
UGAGCUUGGGaAUGGCACAACUGUAAAGAGgAUAUGAAACAUGAGUUGGUCAUUCAUUCUCUCCAAGUUAUUACAUUGACGACAUUGAUUGAUUGAUUGAGUGAGCGCGCGAUACUAACCCUCGCCCGCCAAUGAUCACAUCAGACUCAUGUUUCAUAUCCuCUUUACAGUAGUGCCAUUCcCAAGCUCA   count   length
Libraries combined    Precision: [ total = 93.3% | 5p-arm = 86.6% | 3p-arm = 96.9% ]
-----------------------------------------------------------------------------------------------------------------------------------------------------------------UCUUUACAGUAGUGCCAUUCC--------   94   21
----------AAUGGCACAACUGUAAAGAGG---------------------------------------------------------------------------------------------------------------------------------------------------------------   54   21
-----------------------------------------------------------------------------------------------------------------------------------------------------------------UCUUUACAGUAGUGCCAUUC---------   24   20
-------------------------AAGAGGAUAUGAAACAUGAGU------------------------------------------------------------------------------------------------------------------------------------------------   7   21
----------AAUGGCACAACUGUAAAGAGGAU-------------------------------------------------------------------------------------------------------------------------------------------------------------   3   23
-----------------------------------------------------------------------------------------------------------------------------------------------------------------UCUUUACAGUAGUGCCAUU----------   4   19
------------------------------------------------------------------------------------------------------------------------------------------------------------------CUUUACAGUAGUGCCAUUCCC-------   2   21
-----------------------------------------------------------------------------------------------------------------------------------------------------------------UCUUUACAGUAGUGCCAU-----------   1   18
----------AAUGGCACAACUGUAAAGAGGA--------------------------------------------------------------------------------------------------------------------------------------------------------------   1   22
-----------AUGGCACAACUGUAAAGAGGA--------------------------------------------------------------------------------------------------------------------------------------------------------------   1   21
--------------------------------UAUGAAACAUGAGUUGGUCAU-----------------------------------------------------------------------------------------------------------------------------------------   1   21
----------------------------------------------------------------------------------------------------------------------------------------CACAUCAGACUCAUGUUUCAUAUC------------------------------   1   24
-------------------------------------------------------------------------------------------------------------------------------------------------------------------UUUACAGUAGUGCCAUUCC--------   1   19
Library = 1           Precision: [ total = 95.0% | 5p-arm = 93.0% | 3p-arm = 96.5% ]
-----------------------------------------------------------------------------------------------------------------------------------------------------------------UCUUUACAGUAGUGCCAUUCC--------   42   21
----------AAUGGCACAACUGUAAAGAGG---------------------------------------------------------------------------------------------------------------------------------------------------------------   38   21
-----------------------------------------------------------------------------------------------------------------------------------------------------------------UCUUUACAGUAGUGCCAUUC---------   10   20
-------------------------AAGAGGAUAUGAAACAUGAGU------------------------------------------------------------------------------------------------------------------------------------------------   3   21
----------AAUGGCACAACUGUAAAGAGGAU-------------------------------------------------------------------------------------------------------------------------------------------------------------   2   23
-----------------------------------------------------------------------------------------------------------------------------------------------------------------UCUUUACAGUAGUGCCAUU----------   2   19
------------------------------------------------------------------------------------------------------------------------------------------------------------------CUUUACAGUAGUGCCAUUCCC-------   2   21
-----------------------------------------------------------------------------------------------------------------------------------------------------------------UCUUUACAGUAGUGCCAU-----------   1   18
Library = 2           Precision: [ total = 89.5% | 5p-arm = 70.0% | 3p-arm = 96.4% ]
-----------------------------------------------------------------------------------------------------------------------------------------------------------------UCUUUACAGUAGUGCCAUUCC--------   40   21
----------AAUGGCACAACUGUAAAGAGG---------------------------------------------------------------------------------------------------------------------------------------------------------------   13   21
-----------------------------------------------------------------------------------------------------------------------------------------------------------------UCUUUACAGUAGUGCCAUUC---------   13   20
-------------------------AAGAGGAUAUGAAACAUGAGU------------------------------------------------------------------------------------------------------------------------------------------------   4   21
----------AAUGGCACAACUGUAAAGAGGA--------------------------------------------------------------------------------------------------------------------------------------------------------------   1   22
-----------AUGGCACAACUGUAAAGAGGA--------------------------------------------------------------------------------------------------------------------------------------------------------------   1   21
--------------------------------UAUGAAACAUGAGUUGGUCAU-----------------------------------------------------------------------------------------------------------------------------------------   1   21
----------------------------------------------------------------------------------------------------------------------------------------CACAUCAGACUCAUGUUUCAUAUC------------------------------   1   24
-----------------------------------------------------------------------------------------------------------------------------------------------------------------UCUUUACAGUAGUGCCAUU----------   1   19
-------------------------------------------------------------------------------------------------------------------------------------------------------------------UUUACAGUAGUGCCAUUCC--------   1   19
Library = 3           Precision: [ total = 100.0% | 5p-arm = 100.0% | 3p-arm = 100.0% ]
-----------------------------------------------------------------------------------------------------------------------------------------------------------------UCUUUACAGUAGUGCCAUUCC--------   12   21
----------AAUGGCACAACUGUAAAGAGG---------------------------------------------------------------------------------------------------------------------------------------------------------------   3   21
----------AAUGGCACAACUGUAAAGAGGAU-------------------------------------------------------------------------------------------------------------------------------------------------------------   1   23
-----------------------------------------------------------------------------------------------------------------------------------------------------------------UCUUUACAGUAGUGCCAUUC---------   1   20
-----------------------------------------------------------------------------------------------------------------------------------------------------------------UCUUUACAGUAGUGCCAUU----------   1   19
```

```
                  A                            G---    U   U  UCUCCAA    UU
UGAGCUUGGGaAUGGCAC ACUGUAAAGAGgAUAUGAAACAUGAGUU    GUCA UCA UC       GUUA  A
ACUCGAACcCUUACCGUG UGACAUUUCuCCUAUACUUUGUACUCAG\  /UAGU AGU AG       CAGU  C
                  A                             AU     U   U  UUACAG-    UA
                                                CG
                                                UA             C    UACU
                                                A GUGA------GCG GCGA    A
                                                 \CACU      CGC CGCU    /
                                                      AGUAAC   C    CCCA
```

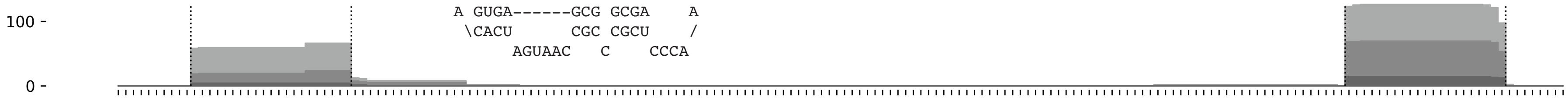


```
((((((((((((((((((.((((((((((((((((((((((((((((.((((.(((.((.......((((......))))......)).))).))))((((((((((.((((.........)))).)))......)))).))))))))))))))))))))))))))))))).))))))))))))))))))
UGAGCUUGGGAAUGGCACAACUGUAAAGAGGAUAUGAAACAUGAGUUGGUCAUUCAUUCUCUCCAAGUUAUUACAUUGACGACAUUGAUUGAUUGAUUGAGUGAGCGCGCGAUACUAACCCUCGCCCGCCAAUGAUCACAUCAGACUCAUGUUUCAUAUCCUCUUUACAGUAGUGCCAUUCCCAAGCUCA
```

A. subglobosum asu-mir-1216

```
.....(.(((((((.((.((((((((((.(..(.(((((((((((((........))))))))))))).)..).)))))))))).)).))))))).)..... -65.75 kcal/mol
UCUACCCAGCcAAUCACCUAGCUACACCCCaCCUGGCCAAUGGCACAGCUACCUGUGUGUCAUUGGCUUGACGaGGUGUAGCUAUGUCAUUGGcUUGAUGAG count    length
Libraries combined          Precision: [ total = 99.7% | 5p-arm = 59.4% | 3p-arm = 99.7% ]
-------------------------------------------------------------------------AGGUGUAGCUAUGUCAUUGGC-------- 97242    21
-------------------------------------------------------------------------AGGUGUAGCUAUGUCAUUGG--------- 17621    20
-------------------------------------------------------------------------AGGUGUAGCUAUGUCAUUG---------- 4455     19
-------------------------------------------------------------------------AGGUGUAGCUAUGUCAUU----------- 273      18
-------------------------------------------------------------------------AGGUGUAGCUAUGUCAUUGGCU------- 196      22
--------------------------------------------------------------------------GGUGUAGCUAUGUCAUUGGC-------- 99       20
-----------------------------------------------------------------------CGAGGUGUAGCUAUGUCAUUG---------- 83       21
----------------------------------------------------CUGUGUGUCAUUGGCUUGACG----------------------------- 43       21
-------------------------------------------------------------------------AGGUGUAGCUAUGUCAUUGGCUU------ 30       23
-----------------------------------------------------------------------CGAGGUGUAGCUAUGUCAUUGG--------- 25       22
----------------------------------------------------CUGUGUGUCAUUGGCUUGA------------------------------- 17       19
-----------------------------------------------------------------------CGAGGUGUAGCUAUGUCAUU----------- 16       20
----------CAAUCACCUAGCUACACCCCA----------------------------------------------------------------------- 13       21
--------------------------------------------------------------------------GGUGUAGCUAUGUCAUUGG--------- 8        19
----------------------------------------------------CUGUGUGUCAUUGGCUUGAC------------------------------ 6        20
----------------------------------------------------CUGUGUGUCAUUGGCUUG-------------------------------- 5        18
------------------------------------------------------------------------GAGGUGUAGCUAUGUCAUUGG--------- 4        21
----------------------------------------------------------------------------UGUAGCUAUGUCAUUGGC-------- 7        18
----------CAAUCACCUAGCUACACCCC------------------------------------------------------------------------ 5        20
------------AUCACCUAGCUACACCCCACC--------------------------------------------------------------------- 2        21
-------------------------------CCUGGCCAAUGGCACAGCU---------------------------------------------------- 3        19
------------------------------------------------------------------------GAGGUGUAGCUAUGUCAUUG---------- 3        20
--------------------------------------------------------------------------GGUGUAGCUAUGUCAUUGGCU------- 4        21
---------------------------------------------------------------------------GUGUAGCUAUGUCAUUGGC-------- 2        19
--------------------------------------------------------------------------------GCUAUGUCAUUGGCUUGAU--- 2        19
---------CCAAUCACCUAGCUACACCCC------------------------------------------------------------------------ 2        21
-----------------------ACACCCCACCUGGCCAAUGGC---------------------------------------------------------- 1        21
--------------------------CCCCACCUGGCCAAUGGCAC-------------------------------------------------------- 1        20
-----------------------------------------------------UGUGUGUCAUUGGCUUGACG----------------------------- 1        20
-----------------------------------------------------UGUGUGUCAUUGGCUUGAC------------------------------ 1        19
--------------------------------------------------------------------------GGUGUAGCUAUGUCAUUG---------- 4        18
----------------------------------------------------------------------------UGUAGCUAUGUCAUUGGCUUG----- 1        21
----------CAAUCACCUAGCUACACCC------------------------------------------------------------------------- 1        19
--------------CACCUAGCUACACCCCACCUGG------------------------------------------------------------------ 2        22
----------------------UACACCCCACCUGGCCAAUGG----------------------------------------------------------- 2        21
---------------------------------------------------CCUGUGUGUCAUUGGCUUGACG----------------------------- 3        22
------------------------------------------------------------------------GAGGUGUAGCUAUGUCAUUGGC-------- 2        22
-------------------------------------------------------------------------AGGUGUAGCUAUGUCAUUGGCUUG----- 1        24
----------------------------------------------------------------------------------UAUGUCAUUGGCUUGAUG-- 1        18
```

```
UCUAC C       C  C          C aC U             GCU
     C AGCcAAU AC UAGCUACACC C  C GGCCAAUGGCACA   A
     G UcGGUUA UG AUCGAUGUGG G  G UCGGUUACUGUGU   C
GAGUA U       C  U          a CA U             GUC
```

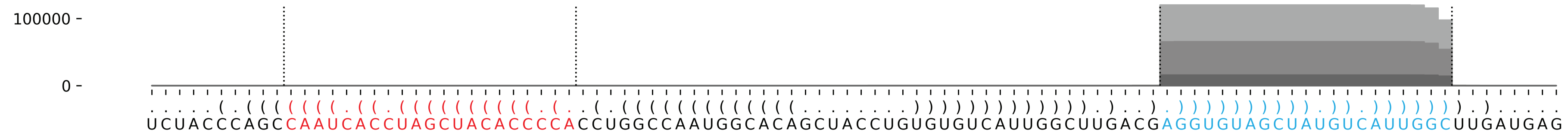

A. subglobosum asu-mir-1217

```
.......(((((((.(((.((((((((((((((((...............(((((((((....))))))))))))))))))))))))).))).))))))).......   -55.33 kcal/mol
UAAUUAAUGAaCGUAUCACCUAUGUCUGAUaCUCAUUCUAAAUUAAUCUUAUAUUGAUCAUUAGAUUAAUAUUGAGUAuCAGACAUAGUUGACACGUUcACUCAUUC   count    length
Libraries combined          Precision: [ total = 98.7% | 5p-arm = 55.6% | 3p-arm = 98.7% ]
------------------------------------------------------------------------------UCAGACAUAGUUGACACGUUC--------   11779    21
------------------------------------------------------------------------------UCAGACAUAGUUGACACGUU---------   206      20
-----------------------------------------------------------------------------AUCAGACAUAGUUGACACGUU---------   62       21
------------------------------------------------------------------------------UCAGACAUAGUUGACACGUUCACU-----   41       24
------------------------------------------------------------------------------UCAGACAUAGUUGACACGU----------   41       19
------------------------------------------------------------------------------UCAGACAUAGUUGACACGUUCACUC----   11       25
------------------------------------------------------------------------------UCAGACAUAGUUGACACGUUCA-------   6        22
------------------------------------------------------------------------------UCAGACAUAGUUGACACG-----------   9        18
---------------------------------------------------------------------------------GACAUAGUUGACACGUUC--------   4        18
----------ACGUAUCACCUAUGUCUGAUA----------------------------------------------------------------------------   8        21
-----------------------------------------------------------------------------AUCAGACAUAGUUGACACGU----------   4        20
--------------------------------------------------------------------------------AGACAUAGUUGACACGUUC--------   7        19
---------AACGUAUCACCUAUGUCUGAU-----------------------------------------------------------------------------   6        21
----------ACGUAUCACCUAUGUCUGAU-----------------------------------------------------------------------------   2        20
--------------------------------------------------------------------AUAUUGAGUAUCAGACAUAGU------------------   3        21
--------------------------------------------------------------------------AGUAUCAGACAUAGUUGACACG-----------   6        22
--------------------------------------------------------------------------------AGACAUAGUUGACACGUUCACUC----   2        23
---------AACGUAUCACCUAUGUCUGA------------------------------------------------------------------------------   1        20
--------------------------------------------------------------------AUAUUGAGUAUCAGACAUAG-------------------   1        20
--------------------------------------------------------------------------AGUAUCAGACAUAGUUGACAC------------   3        21
----------------------------------------------------------------------------UAUCAGACAUAGUUGACACGU----------   2        21
----------------------------------------------------------------------------UAUCAGACAUAGUUGACACGUUCACU-----   1        26
-----------------------------------------------------------------------------AUCAGACAUAGUUGACACG-----------   2        19
-------------------------------------------------------------------------------CAGACAUAGUUGACACGUUCA-------   2        21
-------------------------------------------------------------------------------CAGACAUAGUUGACACGUUCACUC----   1        24
--------------AUCACCUAUGUCUGAUACUC-------------------------------------------------------------------------   1        20
-------------------------------------------------------------------------------CAGACAUAGUUGACACGUUCACU-----   1        23
-------------------------------------------------------------------------------CAGACAUAGUUGACACGUUC--------   1        20
UAAUUAA       A   C                   UUCUAAAUUAAUCUU         A
       UGAaCGU UCA CUAUGUCUGAUaCUCA                AUAUUGAUC U
       AcUUGCA AGU GAUACAGACuAUGAGU----------------UAUAAUUAG U
CUUACUC       C   U                                         A
```

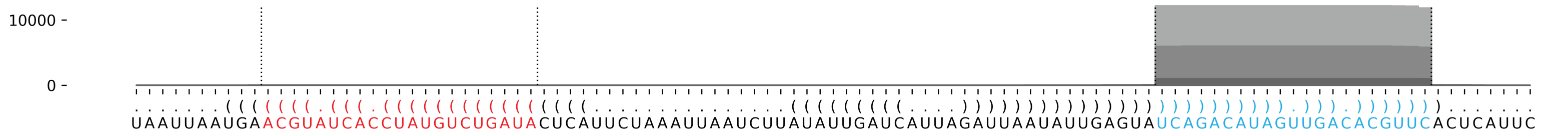

D. fasciculatum dfa-mir-1218

```
((((..((((((((((.((((((((((((((((((((((.((((.(.((..(....(((......)))....)..))).)))))))))))))))))))))))))).)))))))))).)))).    -64.67 kcal/mol
UGGACUGUUCuUUUCACCAUUUAUCUCUUCcAAUAAGAUCAUUUUCUCUCUUUCUCUGGCUUCAACCAAAUUAUAAGGUAGAUAUCUUAUUGGaAGAGAUGAAUGCUGAAAAGaAUUUUCAA    count    length
Libraries combined         Precision: [ total = 95.3% | 5p-arm = 100.0% | 3p-arm = 94.6% ]
---------------------------------------------------------------------------------------------AAGAGAUGAAUGCUGAAAAGA--------    26       21
---------------------------------------------------------------------------------------------AAGAGAUGAAUGCUGAAAAGAA-------    7        22
----------UUUUCACCAUUUAUCUCUUCC-------------------------------------------------------------------------------------------    5        21
----------UUUUCACCAUUUAUCUCUUC--------------------------------------------------------------------------------------------    1        20
---------------------------------------------------------------------------------------UAUUGGAAGAGAUGAAUGCUG--------------    1        21
--------------------------------------------------------------------------------------------GAAGAGAUGAAUGCUGAAAAG---------    1        21
---------------------------------------------------------------------------------------------AAGAGAUGAAUGCUGAAAAGAAU------    2        23
Library = 1        Precision: [ total = 100.0% | 5p-arm = 0% | 3p-arm = 100.0% ]
---------------------------------------------------------------------------------------------AAGAGAUGAAUGCUGAAAAGA--------    9        21
---------------------------------------------------------------------------------------------AAGAGAUGAAUGCUGAAAAGAA-------    3        22
Library = 2        Precision: [ total = 90.9% | 5p-arm = 100.0% | 3p-arm = 87.5% ]
----------UUUUCACCAUUUAUCUCUUCC-------------------------------------------------------------------------------------------    3        21
---------------------------------------------------------------------------------------------AAGAGAUGAAUGCUGAAAAGA--------    3        21
---------------------------------------------------------------------------------------------AAGAGAUGAAUGCUGAAAAGAA-------    3        22
--------------------------------------------------------------------------------------------GAAGAGAUGAAUGCUGAAAAG---------    1        21
---------------------------------------------------------------------------------------------AAGAGAUGAAUGCUGAAAAGAAU------    1        23
Library = 3        Precision: [ total = 95.0% | 5p-arm = 100.0% | 3p-arm = 94.1% ]
---------------------------------------------------------------------------------------------AAGAGAUGAAUGCUGAAAAGA--------    14       21
----------UUUUCACCAUUUAUCUCUUCC-------------------------------------------------------------------------------------------    2        21
----------UUUUCACCAUUUAUCUCUUC--------------------------------------------------------------------------------------------    1        20
---------------------------------------------------------------------------------------UAUUGGAAGAGAUGAAUGCUG--------------    1        21
---------------------------------------------------------------------------------------------AAGAGAUGAAUGCUGAAAAGAA-------    1        22
---------------------------------------------------------------------------------------------AAGAGAUGAAUGCUGAAAAGAAU------    1        23
```

```
    CU          C                      C    U U  CU UCUC   CU
UGGA  GUUCuUUUCA CAUUUAUCUCUUCcAAUAAGAU AUUU C CU  U    UGG  U
 ACUU UAaGAAAAGU GUAAGUAGAGAaGGUUAUUCUA-UAGA G-GA  A    ACC  C
A    U          C                           U    AU UUAA   AA
```

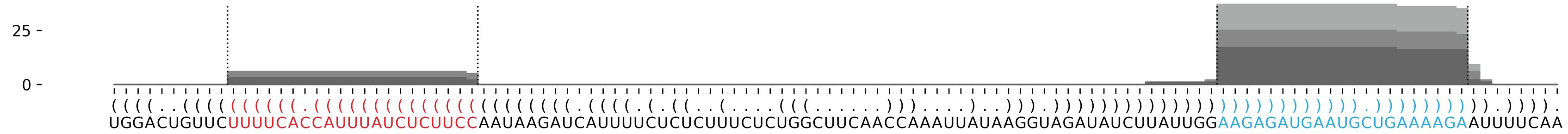

D. fasciculatum dfa-mir-1219

```
(((((((((((((((((.((((((((.((((((((.((((((((..((((...))))...))))))))).........(((.((((.....)))).)))...(((((((.(((...)))))))))))))))))))))).)))))))).)))))))))))))))))   -84.55 kcal/mol
UGCAAUGUUAuAAUGAUCAACGAGUUCAAUaCCUCAAAUUGAAUUCGAUUGUUGGUCUAUGUUCAAUUCUCUUUUGCCAAGCAUUUUGAUGAUGGUUGUUCACCUUUGACGUGUGGCGCAGAGGUGAGGUAuUAAACUCGUUUAUCAUUAUaACAUUGUA   count    length
Libraries combined         Precision: [ total = 92.8% | 5p-arm = 87.7% | 3p-arm = 95.8% ]
-----------------------------------------------------------------------------------------------------------------------------------UUAAACUCGUUUAUCAUUAUA--------   512      21
----------UAAUGAUCAACGAGUUCAAUA---------------------------------------------------------------------------------------------------------------------------------   250      21
----------UAAUGAUCAACGAGUUCAAU----------------------------------------------------------------------------------------------------------------------------------   21       20
----------UAAUGAUCAACGAGUUCAAUAC--------------------------------------------------------------------------------------------------------------------------------   10       22
------------AUGAUCAACGAGUUCAAUACC-------------------------------------------------------------------------------------------------------------------------------   6        21
-------------UGAUCAACGAGUUCAAUACCU------------------------------------------------------------------------------------------------------------------------------   7        21
----------------------------------------------------------------------------------------------------------UGACGUGUGGCGCAGAGGU-----------------------------------   2        19
-----------------------------------------------------------------------------------------------------------------------------------UUAAACUCGUUUAUCAUUAU---------   6        20
UGCAAUGUUAUAAUGAUCAAC-------------------------------------------------------------------------------------------------------------------------------------------   4        21
----------UAAUGAUCAACGAGUUCAA-----------------------------------------------------------------------------------------------------------------------------------   3        19
-----------AAUGAUCAACGAGUUCAAUAC--------------------------------------------------------------------------------------------------------------------------------   6        21
----------------------------------------------------------------------------------------------------------------------CAGAGGUGAGGUAUUAAACUC---------------------   5        21
---------------------------------------------------------------------------------------------------------------------------------UAUUAAACUCGUUUAUCAUUA----------   7        21
-------UUAUAAUGAUCAACGAGUUC-------------------------------------------------------------------------------------------------------------------------------------   4        20
-------UUAUAAUGAUCAACGAGUUCA------------------------------------------------------------------------------------------------------------------------------------   6        21
----------UAAUGAUCAACGAGUUCAAUACC-------------------------------------------------------------------------------------------------------------------------------   1        23
--------------GAUCAACGAGUUCAAUACCUC-----------------------------------------------------------------------------------------------------------------------------   1        21
------------------------------ACCUCAAAUUGAAUUCGAUUG-------------------------------------------------------------------------------------------------------------   1        21
------------------------------------AAUUGAAUUCGAUUGUUGGUC-------------------------------------------------------------------------------------------------------   1        21
-------------------------------------AUUGAAUUCGAUUGUUGGUCU------------------------------------------------------------------------------------------------------   1        21
--------------------------------------UUGAAUUCGAUUGUUGGUCUA-----------------------------------------------------------------------------------------------------   1        21
----------------------------------------------------------AUGUUCAAUUCUCUUUUGCCA---------------------------------------------------------------------------------   2        21
--------------------------------------------------------------------------------------------------------------------CGCAGAGGUGAGGUAUUAAAC-----------------------   2        21
----------------------------------------------------------------------------------------------------------------------CAGAGGUGAGGUAUUAAACU----------------------   1        20
-----------------------------------------------------------------------------------------------------------------------AGAGGUGAGGUAUUAAAC-----------------------   1        18
-----------------------------------------------------------------------------------------------------------------------AGAGGUGAGGUAUUAAACUC---------------------   1        20
---------------------------------------------------------------------------------------------------------------------------------UAUUAAACUCGUUUAUCAUUAU---------   1        22
---------------------------------------------------------------------------------------------------------------------------------UAUUAAACUCGUUUAUCA-------------   1        18
----------------------------------------------------------------------------------------------------------------------------------AUUAAACUCGUUUAUCAUUAU---------   1        21
-----------------------------------------------------------------------------------------------------------------------------------UUAAACUCGUUUAUCAUUA----------   8        19
-----------------------------------------------------------------------------------------------------------------------------------UUAAACUCGUUUAUCAUUAUAA-------   1        22
------------------------------------------------------------------------------------------------------------------------------------UAAACUCGUUUAUCAUUAUAAC------   1        22
Library = 1       Precision: [ total = 94.4% | 5p-arm = 92.8% | 3p-arm = 95.7% ]
-----------------------------------------------------------------------------------------------------------------------------------UUAAACUCGUUUAUCAUUAUA--------   88       21
----------UAAUGAUCAACGAGUUCAAUA---------------------------------------------------------------------------------------------------------------------------------   67       21
----------UAAUGAUCAACGAGUUCAAU----------------------------------------------------------------------------------------------------------------------------------   6        20
----------UAAUGAUCAACGAGUUCAAUAC--------------------------------------------------------------------------------------------------------------------------------   3        22
------------AUGAUCAACGAGUUCAAUACC-------------------------------------------------------------------------------------------------------------------------------   2        21
-------------UGAUCAACGAGUUCAAUACCU------------------------------------------------------------------------------------------------------------------------------   2        21
----------------------------------------------------------------------------------------------------------UGACGUGUGGCGCAGAGGU-----------------------------------   2        19
-----------------------------------------------------------------------------------------------------------------------------------UUAAACUCGUUUAUCAUUAU---------   2        20
UGCAAUGUUAUAAUGAUCAAC-------------------------------------------------------------------------------------------------------------------------------------------   1        21
----------UAAUGAUCAACGAGUUCAA-----------------------------------------------------------------------------------------------------------------------------------   1        19
-----------AAUGAUCAACGAGUUCAAUAC--------------------------------------------------------------------------------------------------------------------------------   1        21
----------------------------------------------------------------------------------------------------------------------CAGAGGUGAGGUAUUAAACUC---------------------   1        21
---------------------------------------------------------------------------------------------------------------------------------UAUUAAACUCGUUUAUCAUUA----------   1        21
Library = 2       Precision: [ total = 86.5% | 5p-arm = 77.2% | 3p-arm = 92.6% ]
-----------------------------------------------------------------------------------------------------------------------------------UUAAACUCGUUUAUCAUUAUA--------   105      21
----------UAAUGAUCAACGAGUUCAAUA---------------------------------------------------------------------------------------------------------------------------------   56       21
----------UAAUGAUCAACGAGUUCAAU----------------------------------------------------------------------------------------------------------------------------------   4        20
----------------------------------------------------------------------------------------------------------------------CAGAGGUGAGGUAUUAAACUC---------------------   4        21
-----------------------------------------------------------------------------------------------------------------------------------UUAAACUCGUUUAUCAUUA----------   4        19
------------AUGAUCAACGAGUUCAAUACC-------------------------------------------------------------------------------------------------------------------------------   3        21
```

D. fasciculatum dfa-mir-1219 continued

```
(((((((((((((((((.((((((((.((((((((.((((((((..((((...))))...))))))))).........(((.((((.....)))).)))...(((((((.(((...)))))))))))))))))))).))))))))).)))))))))))))))))   -84.55 kcal/mol
UGCAAUGUUAuAAUGAUCAACGAGUUCAAUaCCUCAAAUUGAAUUCGAUUGUUGGUCUAUGUUCAAUUCUCUUUUGCCAAGCAUUUUGAUGAUGGUUGUUCACCUUUGACGUGUGGCGCAGAGGUGAGGUAuUAAACUCGUUUAUCAUUAUaACAUUGUA   count   length
UGCAAUGUUAUAAUGAUCAAC-------------------------------------------------------------------------------------------------------------------------------------------   2   21
-------UUAUAAUGAUCAACGAGUUC-------------------------------------------------------------------------------------------------------------------------------------   2   20
-----------AAUGAUCAACGAGUUCAAUAC--------------------------------------------------------------------------------------------------------------------------------   2   21
----------------------------------------------------------AUGUUCAAUUCUCUUUUGCCA---------------------------------------------------------------------------------   2   21
--------------------------------------------------------------------------------------------------------------------CGCAGAGGUGAGGUAUUAAAC-----------------------   2   21
-----------------------------------------------------------------------------------------------------------------------------------UUAAACUCGUUUAUCAUUAU---------   2   20
-------UUAUAAUGAUCAACGAGUUCA------------------------------------------------------------------------------------------------------------------------------------   1   21
----------UAAUGAUCAACGAGUUCAAUAC--------------------------------------------------------------------------------------------------------------------------------   1   22
-------------UGAUCAACGAGUUCAAUACCU------------------------------------------------------------------------------------------------------------------------------   1   21
--------------GAUCAACGAGUUCAAUACCUC-----------------------------------------------------------------------------------------------------------------------------   1   21
------------------------------ACCUCAAAUUGAAUUCGAUUG-------------------------------------------------------------------------------------------------------------   1   21
------------------------------------AAUUGAAUUCGAUUGUUGGUC-------------------------------------------------------------------------------------------------------   1   21
-------------------------------------AUUGAAUUCGAUUGUUGGUCU------------------------------------------------------------------------------------------------------   1   21
--------------------------------------UUGAAUUCGAUUGUUGGUCUA-----------------------------------------------------------------------------------------------------   1   21
-----------------------------------------------------------------------------------------------------------------------AGAGGUGAGGUAUUAAAC-----------------------   1   18
---------------------------------------------------------------------------------------------------------------------------------UAUUAAACUCGUUUAUCAUUA----------   1   21
----------------------------------------------------------------------------------------------------------------------------------AUUAAACUCGUUUAUCAUUAU---------   1   21
-----------------------------------------------------------------------------------------------------------------------------------UUAAACUCGUUUAUCAUUAUAA-------   1   22
Library = 3        Precision: [ total = 94.8% | 5p-arm = 90.2% | 3p-arm = 97.0% ]
-----------------------------------------------------------------------------------------------------------------------------------UUAAACUCGUUUAUCAUUAUA--------   319   21
----------UAAUGAUCAACGAGUUCAAUA---------------------------------------------------------------------------------------------------------------------------------   127   21
----------UAAUGAUCAACGAGUUCAAU----------------------------------------------------------------------------------------------------------------------------------   11   20
----------UAAUGAUCAACGAGUUCAAUAC--------------------------------------------------------------------------------------------------------------------------------   6   22
-------UUAUAAUGAUCAACGAGUUCA------------------------------------------------------------------------------------------------------------------------------------   5   21
---------------------------------------------------------------------------------------------------------------------------------UAUUAAACUCGUUUAUCAUUA----------   5   21
-------------UGAUCAACGAGUUCAAUACCU------------------------------------------------------------------------------------------------------------------------------   4   21
-----------------------------------------------------------------------------------------------------------------------------------UUAAACUCGUUUAUCAUUA----------   4   19
-----------AAUGAUCAACGAGUUCAAUAC--------------------------------------------------------------------------------------------------------------------------------   3   21
-------UUAUAAUGAUCAACGAGUUC-------------------------------------------------------------------------------------------------------------------------------------   2   20
----------UAAUGAUCAACGAGUUCAA-----------------------------------------------------------------------------------------------------------------------------------   2   19
-----------------------------------------------------------------------------------------------------------------------------------UUAAACUCGUUUAUCAUUAU---------   2   20
UGCAAUGUUAUAAUGAUCAAC-------------------------------------------------------------------------------------------------------------------------------------------   1   21
----------UAAUGAUCAACGAGUUCAAUACC-------------------------------------------------------------------------------------------------------------------------------   1   23
------------AUGAUCAACGAGUUCAAUACC-------------------------------------------------------------------------------------------------------------------------------   1   21
----------------------------------------------------------------------------------------------------------------------CAGAGGUGAGGUAUUAAACU----------------------   1   20
-----------------------------------------------------------------------------------------------------------------------AGAGGUGAGGUAUUAAACUC---------------------   1   20
---------------------------------------------------------------------------------------------------------------------------------UAUUAAACUCGUUUAUCAUUAU---------   1   22
---------------------------------------------------------------------------------------------------------------------------------UAUUAAACUCGUUUAUCA-------------   1   18
------------------------------------------------------------------------------------------------------------------------------------UAAACUCGUUUAUCAUUAUAAC------   1   22
```

```
                                           CU-    G
                                     /-GAAU   GAUU U
                                     UACUUG   CUGG /
                                     UA    UAU    U
                                     AU
                 C        C         AAUCUCUUUUGC    G    UU
UGCAAUGUUAuAAUGAU AACGAGUU AAUaCCUC/            \CAA CAUU  G
AUGUUACAaUAUUACUA UUGCUCAA UuAUGGAG-\      /------GUU GUAG  /
                 U        A          UACUU          G    UA
                                     GC
                                     GC   A   G
                                     AUUUG CGU U
                                     \-GAC-GCG /
                                              G
```

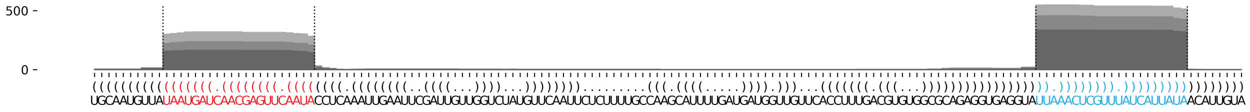

D. fasciculatum dfa-mir-1220-P1

```
.....(.((((((((.((((((((((..((.(((((((..(((((((..........((.(((((..((((((...(((..............))).))))))..))))).))........)))))))..))))))).))..))))))))))).)))))))).).....   -61.47 kcal/mol
AUACUCUAGAuCAUUCCCAUUGUCACCGAAaUCAUUAUUUAAUGAAUCACAAACCAUGCCAUUGUCAAGAAGAAACCCAACCAAUCAAUCAACUGGCUCUUUUAAAUAAUCGCUUCAUCUCAUUCAUUUUAUAAUGACUuAAGUGAUGAUGGUAAUGAUcUUGUUUUA   count   length
Libraries combined            Precision: [ total = 99.9% | 5p-arm = 95.2% | 3p-arm = 99.9% ]
-------------------------------------------------------------------------------------------------------------------------------------------UAAGUGAUGAUGGUAAUGAUC--------   77764   21
-------------------------------------------------------------------------------------------------------------------------------------------UAAGUGAUGAUGGUAAUGAU---------   31349   20
-------------------------------------------------------------------------------------------------------------------------------------------UAAGUGAUGAUGGUAAUGAUCU-------   2904    22
-------------------------------------------------------------------------------------------------------------------------------------------UAAGUGAUGAUGGUAAUGA----------   1326    19
-------------------------------------------------------------------------------------------------------------------------------------------UAAGUGAUGAUGGUAAUG-----------   620     18
-------------------------------------------------------------------------------------------------------------------------------------------UAAGUGAUGAUGGUAAUGAUCUU------   681     23
------------------------------------------------------------------------------------------------------------------------------------------UUAAGUGAUGAUGGUAAUGAUC--------   24      22
--------------------------------------------------------------------------------------------------------------------------------------------AAGUGAUGAUGGUAAUGAUC--------   10      20
----------UCAUUCCCAUUGUCACCG--------------------------------------------------------------------------------------------------------------------------------------------   7       18
-----------------------------------------------------------------------------------------------------------------------------------------CUUAAGUGAUGAUGGUAAUGA----------   7       21
-----------------------------------------------------------------------------------------------------------------------------------------CUUAAGUGAUGAUGGUAAUGAUC--------   9       23
------------------------------------------------------------------------------------------------------------------------------------------UUAAGUGAUGAUGGUAAUGAU---------   36      21
--------------------------------------------------------------------------------------------------------------------------------------------AAGUGAUGAUGGUAAUGAUCU-------   3       21
----------UCAUUCCCAUUGUCACCGAAA-----------------------------------------------------------------------------------------------------------------------------------------   13      21
----------------------------------------------------------------------------------------------------------------------------------------ACUUAAGUGAUGAUGGUAAUGAU---------   3       23
-----------------------------------------------------------------------------------------------------------------------------------------CUUAAGUGAUGAUGGUAAUGAU---------   3       22
-----------------------------------------------------------------------------------------------------------------------------------------CUUAAGUGAUGAUGGUAAUG-----------   5       20
-----------------------------------------------------------------------------------------------------------------------------------------CUUAAGUGAUGAUGGUAAUGAUCU-------   1       24
----------------------------------------------------------------------------------------------------------------------------------------------GUGAUGAUGGUAAUGAUC--------   1       18
--------------------------------------UUAAUGAAUCACAAACCAUG--------------------------------------------------------------------------------------------------------------   1       20
----------------------------------------------------------------------------------------------------------------CUUCAUCUCAUUCAUUUUAUA-----------------------------------   1       21
--------------------------------------------------------------------------------------------------------------------------------UUAUAAUGACUUAAGUGAUG--------------------   1       20
--------------------------------------------------------------------------------------------------------------------------------UUAUAAUGACUUAAGUGAU---------------------   2       19
------------------------------------------------------------------------------------------------------------------------------------AAUGACUUAAGUGAUGAUGGUAAUGAUC--------   1       28
----------------------------------------------------------------------------------------------------------------------------------------ACUUAAGUGAUGAUGGUAAUGA----------   1       22
----------------------------------------------------------------------------------------------------------------------------------------ACUUAAGUGAUGAUGGUAAUG-----------   1       21
------------------------------------------------------------------------------------------------------------------------------------------UUAAGUGAUGAUGGUAAUGAUCU-------   3       23
------------------------------------------------------------------------------------------------------------------------------------------UUAAGUGAUGAUGGUAAUG-----------   1       19
-------------------------------------------------------------------------------------------------------------------------------------------UAAGUGAUGAUGGUAAUGAUCUUGU----   1       25
--------------------------------------------------------------------------------------------------------------------------------------------AAGUGAUGAUGGUAAUGAU---------   8       19
---------------------------------------------------------------------------------------------------------------------------------------------AGUGAUGAUGGUAAUGAU---------   1       18
---------------------------------------------------------------------------------------------------------------------------------------------AGUGAUGAUGGUAAUGAUC--------   1       19
```

```
AUACU U         C           CG  a        UU        CACAAACCAU  C      CA       AAC    ACCAAU
     C AGAuCAUU CCAUUGUCAC  AA UCAUUAU  AAUGAAU              GC AUUGU  AGAAGA   CCA       C
     G UcUAGUAA GGUAGUAGUG  uU AGUAAAUA UUACUUA              CG UAAUA  UUUUCU   GGU       A
AUUUU U         U           AA  C        UU        CUCUACUU--  C      AA       C--    CAACUA
```

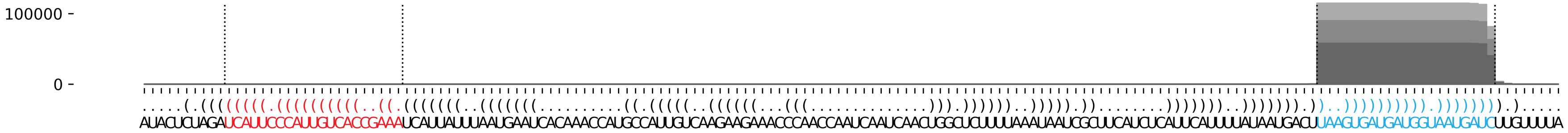

D. fasciculatum dfa-mir-1220-P2

```
.....(.((((((((.((((((((((..(((((((.(((..((((((((...((((.......((.((((.((((..((((((((....)))))))).)))).)))))).))))..)))))))).))).)))))))..)))))))))).)))))))).).....   -66.48 kcal/mol
AUACUCUAGAuCAUUCCCAUUGUCACCCAAaUCAUCAUUAUUUAUUUAUUUAUGUCUCUCAUCAGUGUGUCGACACAGUAUAUAUACAUAUAUAUAUAUGUUUACACCUCGGCAGUGUGGAUAACGAUCAUGAUUuAAGUGAUGAUGGUAAUGAUcUUGUUUUA   count   length
Libraries combined            Precision: [ total = 99.9% | 5p-arm = 87.5% | 3p-arm = 99.9% ]
---------------------------------------------------------------------------------------------------------------------------------------UAAGUGAUGAUGGUAAUGAUC--------   77764   21
---------------------------------------------------------------------------------------------------------------------------------------UAAGUGAUGAUGGUAAUGAU---------   31349   20
---------------------------------------------------------------------------------------------------------------------------------------UAAGUGAUGAUGGUAAUGAUCU-------   2904    22
---------------------------------------------------------------------------------------------------------------------------------------UAAGUGAUGAUGGUAAUGA----------   1326    19
---------------------------------------------------------------------------------------------------------------------------------------UAAGUGAUGAUGGUAAUG-----------   620     18
---------------------------------------------------------------------------------------------------------------------------------------UAAGUGAUGAUGGUAAUGAUCUU------   681     23
--------------------------------------------------------------------------------------------------------------------------------------UUAAGUGAUGAUGGUAAUGAUC--------   24      22
----------------------------------------------------------------------------------------------------------------------------------------AAGUGAUGAUGGUAAUGAUC--------   10      20
--------------------------------------------------------------------------------------------------------------------------------------UUAAGUGAUGAUGGUAAUGAU---------   36      21
----------------------------------------------------------------------------------------------------------------------------------------AAGUGAUGAUGGUAAUGAUCU-------   3       21
----------UCAUUCCCAUUGUCACCCAAA-------------------------------------------------------------------------------------------------------------------------------------   13      21
--------------------------------------------------------------------------------------------UAUAUAUGUUUACACCUCGGC---------------------------------------------------   1       21
------------------------------------------------------------------------------------------------------------UCGGCAGUGUGGAUAACGAUC-----------------------------------   8       21
------------------------------------------------------------------------------------------------------------------GUGUGGAUAACGAUCAUGAUU-----------------------------   1       21
-------------------------------------------------------------------------------------------------------------------UGUGGAUAACGAUCAUGAUUU----------------------------   1       21
------------------------------------------------------------------------------------------------------------------------------------AUUUAAGUGAUGAUGGUAAUGAU---------   1       23
-------------------------------------------------------------------------------------------------------------------------------------UUUAAGUGAUGAUGGUAAUG-----------   1       20
------------------------------------------------------------------------------------------------------------------------------------------GUGAUGAUGGUAAUGAUC--------   1       18
----------UCAUUCCCAUUGUCACCCAAAU------------------------------------------------------------------------------------------------------------------------------------   1       22
-------------UUCCCAUUGUCACCCAAAUCA----------------------------------------------------------------------------------------------------------------------------------   1       21
----------------------------------------------------UGUCUCUCAUCAGUGUGUCGA-------------------------------------------------------------------------------------------   1       21
----------------------------------------------------------------------------------------------UAUAUGUUUACACCUCGGCAG-------------------------------------------------   2       21
---------------------------------------------------------------------------------------------------------ACCUCGGCAGUGUGGAUAACGAUCAUGAU------------------------------   1       29
-----------------------------------------------------------------------------------------------------------CUCGGCAGUGUGGAUAACGAUCAUGAUU-----------------------------   1       28
------------------------------------------------------------------------------------------------------------UCGGCAGUGUGGAUAACGAUCAUGAU------------------------------   1       26
-------------------------------------------------------------------------------------------------------------CGGCAGUGUGGAUAACGAUCA----------------------------------   3       21
-------------------------------------------------------------------------------------------------------------------------------------UUUAAGUGAUGAUGGUAAUGAU---------   2       22
-------------------------------------------------------------------------------------------------------------------------------------UUUAAGUGAUGAUGGUAAUGAUCU-------   1       24
-------------------------------------------------------------------------------------------------------------------------------------UUUAAGUGAUGAUGGUAAUGAUC--------   5       23
-------------------------------------------------------------------------------------------------------------------------------------UUUAAGUGAUGAUGGUAAUGA----------   1       21
--------------------------------------------------------------------------------------------------------------------------------------UUAAGUGAUGAUGGUAAUGAUCU-------   3       23
--------------------------------------------------------------------------------------------------------------------------------------UUAAGUGAUGAUGGUAAUG-----------   1       19
---------------------------------------------------------------------------------------------------------------------------------------UAAGUGAUGAUGGUAAUGAUCUUGU----   1       25
----------------------------------------------------------------------------------------------------------------------------------------AAGUGAUGAUGGUAAUGAU---------   8       19
-----------------------------------------------------------------------------------------------------------------------------------------AGUGAUGAUGGUAAUGAU---------   1       18
-----------------------------------------------------------------------------------------------------------------------------------------AGUGAUGAUGGUAAUGAUC--------   1       19
```

```
AUACU U         C          CC        C   AU        UUA     UCUCAUC  U     C    CA         A
     C AGAuCAUU CCAUUGUCAC   AAaUCAU AUU  UUAUUUAU   UGUC       AG GUGU GACA  GUAUAUAU C
     G UcUAGUAA GGUAGUAGUG   uUUAGUA UAG  AAUAGGUG   ACGG       UC-CACA UUGU  UAUAUAUA A
AUUUU U         U          AA        C   C-        UG-     C------        U    A-         U
```

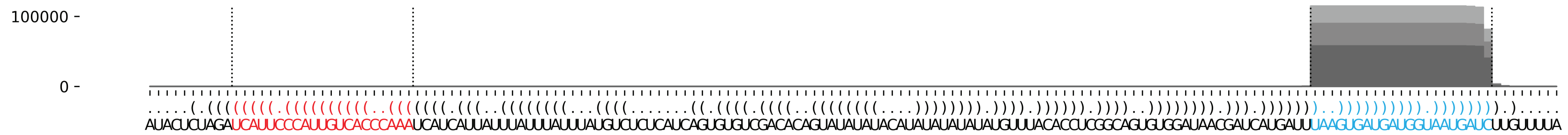

P. polycephalum ppo-mir-1221

```
(((((((((((((((((((((((.(((((((((((((((((.((((....................(((.....................)))....)))).))))))))))))))))).)))))))))))))))))))))))   -81.92 kcal/mol
CUUCUUUUGGaGGAUUUCAAAGUGUAUGACgAGUAUCACUACCUAAUUUUCAAACUUACAAACAAACCUAAUUACUUCUGCGCACAAUAUGGUACACUAGGAAGUGAUACUCGuCAUAAACUUUGAAAUCCUCCcAAAAGGAG   count    length
Libraries combined         Precision: [ total = 99.5% | 5p-arm = 97.7% | 3p-arm = 99.6% ]
-----------------------------------------------------------------------------------------------------------------UCAUAAACUUUGAAAUCCUCC---------   2979     21
-----------------------------------------------------------------------------------------------------------------UCAUAAACUUUGAAAUCCU-----------   1272     19
-----------------------------------------------------------------------------------------------------------------UCAUAAACUUUGAAAUCCUC----------   118      20
----------AGGAUUUCAAAGUGUAUGACG----------------------------------------------------------------------------------------------------------------   28       21
-------------------------------------------------------------------------------------------------------------------AUAAACUUUGAAAUCCUCC---------   9        19
----------AGGAUUUCAAAGUGUAUGAC-----------------------------------------------------------------------------------------------------------------   14       20
---------------------------------------------------------------------------------------------ACACUAGGAAGUGAUACUCG------------------------------   2        20
-----------------------------------------------------------------------------------------------------------------UCAUAAACUUUGAAAUCC------------   10       18
--------------------------------------------------------------------------------------------------------------------UAAACUUUGAAAUCCUCC---------   4        18
------------------------------------------------------------------------------------------------------------------CAUAAACUUUGAAAUCCUCC---------   3        20
------------------------------------------------------------------------------------------------------------------CAUAAACUUUGAAAUCCU-----------   1        18
-----UUUGGAGGAUUUCAAAGUG-----------------------------------------------------------------------------------------------------------------------   1        19
-----------------------------------------------------------------------------------------------------------------UCAUAAACUUUGAAAUCCUCCA--------   1        22
Library = 1       Precision: [ total = 99.4% | 5p-arm = 100.0% | 3p-arm = 99.4% ]
-----------------------------------------------------------------------------------------------------------------UCAUAAACUUUGAAAUCCUCC---------   1107     21
-----------------------------------------------------------------------------------------------------------------UCAUAAACUUUGAAAUCCU-----------   409      19
-----------------------------------------------------------------------------------------------------------------UCAUAAACUUUGAAAUCCUC----------   46       20
----------AGGAUUUCAAAGUGUAUGACG----------------------------------------------------------------------------------------------------------------   8        21
-------------------------------------------------------------------------------------------------------------------AUAAACUUUGAAAUCCUCC---------   3        19
----------AGGAUUUCAAAGUGUAUGAC-----------------------------------------------------------------------------------------------------------------   2        20
---------------------------------------------------------------------------------------------ACACUAGGAAGUGAUACUCG------------------------------   2        20
-----------------------------------------------------------------------------------------------------------------UCAUAAACUUUGAAAUCC------------   2        18
--------------------------------------------------------------------------------------------------------------------UAAACUUUGAAAUCCUCC---------   2        18
------------------------------------------------------------------------------------------------------------------CAUAAACUUUGAAAUCCUCC---------   1        20
------------------------------------------------------------------------------------------------------------------CAUAAACUUUGAAAUCCU-----------   1        18
Library = 2       Precision: [ total = 99.6% | 5p-arm = 100.0% | 3p-arm = 99.6% ]
-----------------------------------------------------------------------------------------------------------------UCAUAAACUUUGAAAUCCUCC---------   1579     21
-----------------------------------------------------------------------------------------------------------------UCAUAAACUUUGAAAUCCU-----------   746      19
-----------------------------------------------------------------------------------------------------------------UCAUAAACUUUGAAAUCCUC----------   66       20
----------AGGAUUUCAAAGUGUAUGACG----------------------------------------------------------------------------------------------------------------   16       21
----------AGGAUUUCAAAGUGUAUGAC-----------------------------------------------------------------------------------------------------------------   9        20
-----------------------------------------------------------------------------------------------------------------UCAUAAACUUUGAAAUCC------------   8        18
-------------------------------------------------------------------------------------------------------------------AUAAACUUUGAAAUCCUCC---------   5        19
------------------------------------------------------------------------------------------------------------------CAUAAACUUUGAAAUCCUCC---------   2        20
--------------------------------------------------------------------------------------------------------------------UAAACUUUGAAAUCCUCC---------   2        18
-----------------------------------------------------------------------------------------------------------------UCAUAAACUUUGAAAUCCUCCA--------   1        22
Library = 3       Precision: [ total = 99.5% | 5p-arm = 87.5% | 3p-arm = 99.8% ]
-----------------------------------------------------------------------------------------------------------------UCAUAAACUUUGAAAUCCUCC---------   293      21
-----------------------------------------------------------------------------------------------------------------UCAUAAACUUUGAAAUCCU-----------   117      19
-----------------------------------------------------------------------------------------------------------------UCAUAAACUUUGAAAUCCUC----------   6        20
----------AGGAUUUCAAAGUGUAUGACG----------------------------------------------------------------------------------------------------------------   4        21
----------AGGAUUUCAAAGUGUAUGAC-----------------------------------------------------------------------------------------------------------------   3        20
-----UUUGGAGGAUUUCAAAGUG-----------------------------------------------------------------------------------------------------------------------   1        19
-------------------------------------------------------------------------------------------------------------------AUAAACUUUGAAAUCCUCC---------   1        19
```

```
                       G                A    AUUUUCAAACUUACAAACAA   UAAUUACUUC
CUUCUUUUGGaGGAUUUCAAAGU UAUGACgAGUAUCACU CCUA                    ACC          U
GAGGAAAAcCUCCUAAAGUUUCA AUACuGCUCAUAGUGA GGAU    ----------------UGG          G
                       A                A    CACA                   UAUAACACGC
```

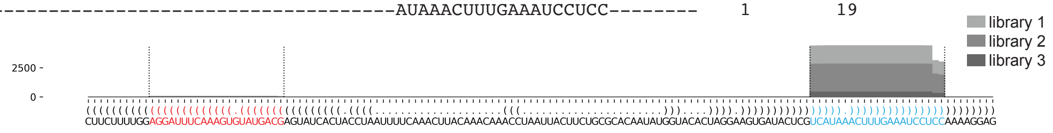

P. polycephalum ppo-mir-1222

```
.((((((((((.(((((.(.(((.((((((((((((((((.((...((((((((((.(((((((((((((.(((((((((((..((.....((((((...((....))....))))))).....)).))))))))))).))).))))).....))).))))))))))))...))..))))))))))))))))).))).).))))).)))))))))). -128.63 kcal/mol
AGUUGUUCCCuCAGGUUGAGUUGUCCACAGCuGUUGCACUCCUGAAUCUGcACUUGACUACUUGUAGAAAAAUACUUUGCUGAUGGAUAGAUAGUGCCAAGGUUGGCCAUAAGCACUACAAAGCCCUAGCAAAGUAUCUUUCCUACAGCCCAAGUCAGCAAGUGUgGAAUAAGAAAGUGCAACAGcuGUGGCCAAGUGAAUCUAAGgGAACAAUU count length
Libraries combined      Precision: [ total = 98.1% | 5p-arm = 98.5% | 3p-arm = 92.6% ]
----------UCAGGUUGAGUUGUCCACAGC---------------------------------------------------------------------------------------------------------------------------------------------------------------------------------- 241 21
----------UCAGGUUGAGUUGUCCACA------------------------------------------------------------------------------------------------------------------------------------------------------------------------------------ 41 19
-------------------------------UGUUGCACUCCUGAAUCUGC-------------------------------------------------------------------------------------------------------------------------------------------------------------------- 58 20
------------------------------------------------------------------------------------------------------------------------------------------------------------------------------------------UGUGGCCAAGUGAAUCUAAG--------- 22 20
----------UCAGGUUGAGUUGUCCAC------------------------------------------------------------------------------------------------------------------------------------------------------------------------------------------- 17 18
----------UCAGGUUGAGUUGUCCACAG----------------------------------------------------------------------------------------------------------------------------------------------------------------------------------- 26 20
----------UCAGGUUGAGUUGUCCACAGCU--------------------------------------------------------------------------------------------------------------------------------------------------------------------------------- 4 22
-------------------------------UGUUGCACUCCUGAAUCUG--------------------------------------------------------------------------------------------------------------------------------------------------------------------- 3 19
------------------------------------------------------------------------------------------------------------------------------------------------------------------UGUGGAAUAAGAAAGUGCAACAG------------------------------ 1 23
------------------------------------------------------------------------------------------------------------------------------------------------------------------------------------------UGUGGCCAAGUGAAUCUAA---------- 1 19
------------------------------------------------------------------------------------------------------------------------------------------------------------------------------------------UGUGGCCAAGUGAAUCUAAGG-------- 1 21
-----------CAGGUUGAGUUGUCCACAGC---------------------------------------------------------------------------------------------------------------------------------------------------------------------------------- 2 20
---------------------------------UUGCACUCCUGAAUCUGCAC------------------------------------------------------------------------------------------------------------------------------------------------------------------ 1 20
---------------------------------------------------ACUUGACUACUUGUAGAAAAAUACUUUGCUGA------------------------------------------------------------------------------------------------------------------------------------ 3 32
---------------------------------------------------------------------------------------------------------------------------------------------------------------------GGAAUAAGAAAGUGCAACAGC----------------------------- 1 21
----------------------------------------------------------------------------------------------------------------------------------------------------------------------GAAUAAGAAAGUGCAACAGC----------------------------- 1 20
Library = 1 Precision: [ total = 99.3% | 5p-arm = 100.0% | 3p-arm = 92.9% ]
----------UCAGGUUGAGUUGUCCACAGC---------------------------------------------------------------------------------------------------------------------------------------------------------------------------------- 81 21
----------UCAGGUUGAGUUGUCCACA------------------------------------------------------------------------------------------------------------------------------------------------------------------------------------ 18 19
-------------------------------UGUUGCACUCCUGAAUCUGC-------------------------------------------------------------------------------------------------------------------------------------------------------------------- 18 20
------------------------------------------------------------------------------------------------------------------------------------------------------------------------------------------UGUGGCCAAGUGAAUCUAAG--------- 11 20
----------UCAGGUUGAGUUGUCCAC------------------------------------------------------------------------------------------------------------------------------------------------------------------------------------------- 10 18
----------UCAGGUUGAGUUGUCCACAG----------------------------------------------------------------------------------------------------------------------------------------------------------------------------------- 8 20
----------UCAGGUUGAGUUGUCCACAGCU--------------------------------------------------------------------------------------------------------------------------------------------------------------------------------- 2 22
-------------------------------UGUUGCACUCCUGAAUCUG--------------------------------------------------------------------------------------------------------------------------------------------------------------------- 1 19
------------------------------------------------------------------------------------------------------------------------------------------------------------------UGUGGAAUAAGAAAGUGCAACAG------------------------------ 1 23
------------------------------------------------------------------------------------------------------------------------------------------------------------------------------------------UGUGGCCAAGUGAAUCUAA---------- 1 19
------------------------------------------------------------------------------------------------------------------------------------------------------------------------------------------UGUGGCCAAGUGAAUCUAAGG-------- 1 21
Library = 2 Precision: [ total = 97.9% | 5p-arm = 98.2% | 3p-arm = 92.3% ]
----------UCAGGUUGAGUUGUCCACAGC---------------------------------------------------------------------------------------------------------------------------------------------------------------------------------- 135 21
-------------------------------UGUUGCACUCCUGAAUCUGC-------------------------------------------------------------------------------------------------------------------------------------------------------------------- 36 20
----------UCAGGUUGAGUUGUCCACA------------------------------------------------------------------------------------------------------------------------------------------------------------------------------------ 20 19
----------UCAGGUUGAGUUGUCCACAG----------------------------------------------------------------------------------------------------------------------------------------------------------------------------------- 17 20
------------------------------------------------------------------------------------------------------------------------------------------------------------------------------------------UGUGGCCAAGUGAAUCUAAG--------- 11 20
----------UCAGGUUGAGUUGUCCAC------------------------------------------------------------------------------------------------------------------------------------------------------------------------------------------- 5 18
---------------------------------------------------ACUUGACUACUUGUAGAAAAAUACUUUGCUGA------------------------------------------------------------------------------------------------------------------------------------ 3 32
----------UCAGGUUGAGUUGUCCACAGCU--------------------------------------------------------------------------------------------------------------------------------------------------------------------------------- 2 22
-------------------------------UGUUGCACUCCUGAAUCUG--------------------------------------------------------------------------------------------------------------------------------------------------------------------- 2 19
---------------------------------UUGCACUCCUGAAUCUGCAC------------------------------------------------------------------------------------------------------------------------------------------------------------------ 1 20
---------------------------------------------------------------------------------------------------------------------------------------------------------------------GGAAUAAGAAAGUGCAACAGC----------------------------- 1 21
----------------------------------------------------------------------------------------------------------------------------------------------------------------------GAAUAAGAAAGUGCAACAGC----------------------------- 1 20
Library = 3 Precision: [ total = 94.6% | 5p-arm = 94.6% | 3p-arm = 0% ]
----------UCAGGUUGAGUUGUCCACAGC---------------------------------------------------------------------------------------------------------------------------------------------------------------------------------- 25 21
-------------------------------UGUUGCACUCCUGAAUCUGC-------------------------------------------------------------------------------------------------------------------------------------------------------------------- 4 20
----------UCAGGUUGAGUUGUCCACA------------------------------------------------------------------------------------------------------------------------------------------------------------------------------------ 3 19
----------UCAGGUUGAGUUGUCCAC------------------------------------------------------------------------------------------------------------------------------------------------------------------------------------------- 2 18
-----------CAGGUUGAGUUGUCCACAGC---------------------------------------------------------------------------------------------------------------------------------------------------------------------------------- 2 20
----------UCAGGUUGAGUUGUCCACAG----------------------------------------------------------------------------------------------------------------------------------------------------------------------------------- 1 20
```

```
A          C     G G   U                C   GAA          A                    A           AU  AUAGA      CAA    U
 GUUGUUCCCu AGGUU A UUG CCACAGcuGUUGCACU -CU   UCUGcACUUG CU-ACU-----UGUAG-AAA AUACUUUGCUG  GG     UAGUGC   -GG U
 UAACAAGgGA UCUAA U AAC GGUGucGACAACGUGA  GA   AGgUGUGAAC-GA UGA     ACAUC UUU UAUGAAACGAU -CC     AUCACG    CC G
U          A     G G   C                AA  AUA             C   ACCCG     C   C           C   GAAAC      AAUA   G
```

P. polycephalum ppo-mir-1222 continued

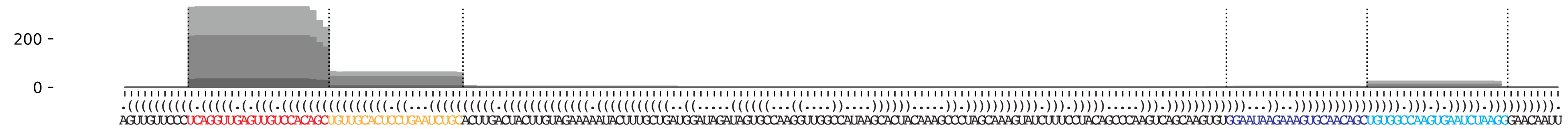

P. polycephalum ppo-mir-1223

```
..((((((.(((((((.((..(((.((.((((((((.....)).)))))).)).)))..)).))))))).))))))..        -32.06 kcal/mol
UAAUAUUUUGuGAAAAAUGCUGGCUGUCAgUGAGAAUCUAUUUCCUCACUaAUUGCUUCCACUUUUCACcAAAUGUGA        count    length
Libraries combined         Precision: [ total = 100.0% | 5p-arm = 100.0% | 3p-arm = 100.0% ]
----------UGAAAAAUGCUGGCUGUCAG------------------------------------------------        100      20
----------UGAAAAAUGCUGGCUGUCA-------------------------------------------------        6        19
----------UGAAAAAUGCUGGCUGUCAGU-----------------------------------------------        6        21
--------------------------------------------------AAUUGCUUCCACUUUUCACC--------        4        20
----------UGAAAAAUGCUGGCUGUC--------------------------------------------------        5        18
Library = 1        Precision: [ total = 100.0% | 5p-arm = 100.0% | 3p-arm = 100.0% ]
----------UGAAAAAUGCUGGCUGUCAG------------------------------------------------        42       20
----------UGAAAAAUGCUGGCUGUCA-------------------------------------------------        2        19
----------UGAAAAAUGCUGGCUGUCAGU-----------------------------------------------        2        21
--------------------------------------------------AAUUGCUUCCACUUUUCACC--------        2        20
----------UGAAAAAUGCUGGCUGUC--------------------------------------------------        1        18
Library = 2        Precision: [ total = 100.0% | 5p-arm = 100.0% | 3p-arm = 100.0% ]
----------UGAAAAAUGCUGGCUGUCAG------------------------------------------------        50       20
----------UGAAAAAUGCUGGCUGUCA-------------------------------------------------        4        19
----------UGAAAAAUGCUGGCUGUCAGU-----------------------------------------------        4        21
----------UGAAAAAUGCUGGCUGUC--------------------------------------------------        3        18
--------------------------------------------------AAUUGCUUCCACUUUUCACC--------        2        20
Library = 3        Precision: [ total = 100.0% | 5p-arm = 100.0% | 3p-arm = 0% ]
----------UGAAAAAUGCUGGCUGUCAG------------------------------------------------        8        20
----------UGAAAAAUGCUGGCUGUC--------------------------------------------------        1        18

UA      U       A  CU   U  C          UC
  AUAUUU GuGAAAA UG  GGC GU AgUGAG-AA   \
  UGUAAA CACUUUU AC  UCG UA UCACUC UU   U
AG      c       C  CU   U  a       C  UA
```

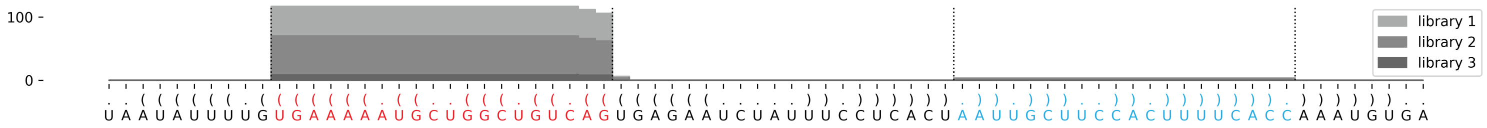

P. polycephalum ppo-mir-1224

```
...((((..((((((((((((((((((((((....(((.((((((((..((((((...))).)))..)))))))).)))....)))))))))))))))))))))).)))).... -68.01 kcal/mol
UCAGGUUUAGuUUAGAACAUGAACUCGGACuCACAGACGCGGGCUUUCAGGUCAAAGUUUGCACUAAGGGGUCCGAGUCCACAAGuCUGAGUUUGUGUUCUAAACcAACCCAGC count length
Libraries combined         Precision: [ total = 98.5% | 5p-arm = 98.4% | 3p-arm = 100.0% ]
----------UUUAGAACAUGAACUCGGACU-----------------------------------------------------------------------------------  124  21
-------------------------------------------------------------------------------------UCUGAGUUUGUGUUCUAAACC--------  9    21
------------UAGAACAUGAACUCGGACU-----------------------------------------------------------------------------------  2    19
----------UUUAGAACAUGAACUCGGA-------------------------------------------------------------------------------------  1    19
Library = 1        Precision: [ total = 98.1% | 5p-arm = 98.0% | 3p-arm = 100.0% ]
----------UUUAGAACAUGAACUCGGACU-----------------------------------------------------------------------------------  48   21
-------------------------------------------------------------------------------------UCUGAGUUUGUGUUCUAAACC--------  4    21
------------UAGAACAUGAACUCGGACU-----------------------------------------------------------------------------------  1    19
Library = 2        Precision: [ total = 98.6% | 5p-arm = 98.5% | 3p-arm = 100.0% ]
----------UUUAGAACAUGAACUCGGACU-----------------------------------------------------------------------------------  64   21
-------------------------------------------------------------------------------------UCUGAGUUUGUGUUCUAAACC--------  4    21
------------UAGAACAUGAACUCGGACU-----------------------------------------------------------------------------------  1    19
Library = 3        Precision: [ total = 100.0% | 5p-arm = 100.0% | 3p-arm = 100.0% ]
----------UUUAGAACAUGAACUCGGACU-----------------------------------------------------------------------------------  12   21
----------UUUAGAACAUGAACUCGGA-------------------------------------------------------------------------------------  1    19
-------------------------------------------------------------------------------------UCUGAGUUUGUGUUCUAAACC--------  1    21
```

```
UCA    UA                      CACA   G        CA       A
   GGUU  GuUUAGAACAUGAACUCGGACu    GAC CGGGCUUU  GGU-CAA \
    CCAA CAAAUCUUGUGUUUGAGUCuGA    CUG GCCUGGGG  UCA GUU G
CGAC    c                      ACAC   A        AA   C   U
```

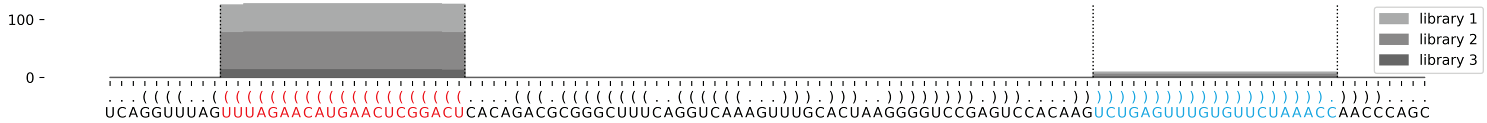

P. polycephalum ppo-mir-1225

```
((((..(((((((.((((.(((((.(((((((((((((((((((((((.(((((((...((.(((((.(((.((.....)).))).)))))))...))))))).))))).)))))))))))))))))).))))).)))).)))))))..)))) -98.02 kcal/mol
UUCAUAaGGAUGGAUGGUAUCGAGCAcaAUUAUGCCAGUGAACCUCUgUGUGUAUGAGCUGUCUCUUUAAAAAUCCAUAAUAUUUUGGGAGCAAUACAUACACCCAgAGUGUUCACUGGUAUAAUUGuaCUUGACACCAACCAUCCUAcUGAA count    length
Libraries combined         Precision: [ total = 94.4% | 5p-arm = 94.8% | 3p-arm = 87.5% ]
---------------------------AAUUAUGCCAGUGAACCUCUG--------------------------------------------------------------------------------------------------------- 153      21
---------------------------AAUUAUGCCAGUGAACCUC----------------------------------------------------------------------------------------------------------- 29       19
---------------------------AAUUAUGCCAGUGAACCUCU---------------------------------------------------------------------------------------------------------- 31       20
------AGGAUGGAUGGUAUCGAGCAC------------------------------------------------------------------------------------------------------------------------------ 22       21
---------------------------AAUUAUGCCAGUGAACCUCUGU-------------------------------------------------------------------------------------------------------- 16       22
--------------------------------------------------------------------------------------------------------------------------------ACUUGACACCAACCAUCCUAC---- 12       21
------AGGAUGGAUGGUAUCGAGC-------------------------------------------------------------------------------------------------------------------------------- 4        19
----------------------------------------------------------------------------------------------------------GAGUGUUCACUGGUAUAAUUGU------------------------- 2        22
-UCAUAAGGAUGGAUGGUAUCGAGCAC------------------------------------------------------------------------------------------------------------------------------ 1        26
----------------------------AUUAUGCCAGUGAACCUCUG--------------------------------------------------------------------------------------------------------- 3        20
---------------------------------------------------------------------------------------------------------AGAGUGUUCACUGGUAUAAUUGU------------------------- 1        23
UUCAUAAGGAUGGAUGGUAUCGAGC-------------------------------------------------------------------------------------------------------------------------------- 1        25
----UAAGGAUGGAUGGUAUCGAGCAC------------------------------------------------------------------------------------------------------------------------------ 1        23
----UAAGGAUGGAUGGUAUCGAGC-------------------------------------------------------------------------------------------------------------------------------- 1        21
---------------------------AAUUAUGCCAGUGAACCU------------------------------------------------------------------------------------------------------------ 1        18
---------------------------AAUUAUGCCAGUGAACCUCUGUGUGUA--------------------------------------------------------------------------------------------------- 1        27
-----------------------------UUAUGCCAGUGAACCUCUGUGUGUA--------------------------------------------------------------------------------------------------- 3        25
------------------------------UAUGCCAGUGAACCUCUGU-------------------------------------------------------------------------------------------------------- 1        19
------------------------------------------------UGUGUAUGAGCUGUCUCUUU------------------------------------------------------------------------------------- 2        20
----------------------------------------------------------------------------------------------------------------------------------UUGACACCAACCAUCCUACUG-- 1        21
Library = 1       Precision: [ total = 97.4% | 5p-arm = 98.2% | 3p-arm = 85.7% ]
---------------------------AAUUAUGCCAGUGAACCUCUG--------------------------------------------------------------------------------------------------------- 65       21
---------------------------AAUUAUGCCAGUGAACCUC----------------------------------------------------------------------------------------------------------- 15       19
---------------------------AAUUAUGCCAGUGAACCUCU---------------------------------------------------------------------------------------------------------- 12       20
------AGGAUGGAUGGUAUCGAGCAC------------------------------------------------------------------------------------------------------------------------------ 7        21
---------------------------AAUUAUGCCAGUGAACCUCUGU-------------------------------------------------------------------------------------------------------- 6        22
--------------------------------------------------------------------------------------------------------------------------------ACUUGACACCAACCAUCCUAC---- 4        21
------AGGAUGGAUGGUAUCGAGC-------------------------------------------------------------------------------------------------------------------------------- 3        19
----------------------------------------------------------------------------------------------------------GAGUGUUCACUGGUAUAAUUGU------------------------- 2        22
-UCAUAAGGAUGGAUGGUAUCGAGCAC------------------------------------------------------------------------------------------------------------------------------ 1        26
----------------------------AUUAUGCCAGUGAACCUCUG--------------------------------------------------------------------------------------------------------- 1        20
---------------------------------------------------------------------------------------------------------AGAGUGUUCACUGGUAUAAUUGU------------------------- 1        23
Library = 2       Precision: [ total = 91.6% | 5p-arm = 91.7% | 3p-arm = 88.9% ]
---------------------------AAUUAUGCCAGUGAACCUCUG--------------------------------------------------------------------------------------------------------- 78       21
---------------------------AAUUAUGCCAGUGAACCUCU---------------------------------------------------------------------------------------------------------- 18       20
------AGGAUGGAUGGUAUCGAGCAC------------------------------------------------------------------------------------------------------------------------------ 14       21
---------------------------AAUUAUGCCAGUGAACCUC----------------------------------------------------------------------------------------------------------- 12       19
---------------------------AAUUAUGCCAGUGAACCUCUGU-------------------------------------------------------------------------------------------------------- 10       22
--------------------------------------------------------------------------------------------------------------------------------ACUUGACACCAACCAUCCUAC---- 8        21
-----------------------------UUAUGCCAGUGAACCUCUGUGUGUA--------------------------------------------------------------------------------------------------- 3        25
----------------------------AUUAUGCCAGUGAACCUCUG--------------------------------------------------------------------------------------------------------- 2        20
------------------------------------------------UGUGUAUGAGCUGUCUCUUU------------------------------------------------------------------------------------- 2        20
UUCAUAAGGAUGGAUGGUAUCGAGC-------------------------------------------------------------------------------------------------------------------------------- 1        25
----UAAGGAUGGAUGGUAUCGAGCAC------------------------------------------------------------------------------------------------------------------------------ 1        23
----UAAGGAUGGAUGGUAUCGAGC-------------------------------------------------------------------------------------------------------------------------------- 1        21
---------------------------AAUUAUGCCAGUGAACCU------------------------------------------------------------------------------------------------------------ 1        18
---------------------------AAUUAUGCCAGUGAACCUCUGUGUGUA--------------------------------------------------------------------------------------------------- 1        27
------------------------------UAUGCCAGUGAACCUCUGU-------------------------------------------------------------------------------------------------------- 1        19
----------------------------------------------------------------------------------------------------------------------------------UUGACACCAACCAUCCUACUG-- 1        21
```

P. polycephalum ppo-mir-1225 continued

```
((((..(((((((.((((.(((((.(((((((((((((((((((((((.(((((((...((.(((((.(((.((.....)).))).)))))))...))))))).))))).)))))))))))))))))).))))).)))).)))))))..)))) -98.02 kcal/mol
UUCAUAaGGAUGGAUGGUAUCGAGCAcaAUUAUGCCAGUGAACCUCUgUGUGUAUGAGCUGUCUCUUUAAAAAUCCAUAAUAUUUUGGGAGCAAUACAUACACCCAgAGUGUUCACUGGUAUAAUUGuaCUUGACACCAACCAUCCUAcUGAA count    length
Library = 3       Precision: [ total = 100.0% | 5p-arm = 100.0% | 3p-arm = 0% ]
---------------------------AAUUAUGCCAGUGAACCUCUG-------------------------------------------------------------------------------------------------------- 10       21
---------------------------AAUUAUGCCAGUGAACCUC---------------------------------------------------------------------------------------------------------- 2        19
------AGGAUGGAUGGUAUCGAGCAC----------------------------------------------------------------------------------------------------------------------------- 1        21
------AGGAUGGAUGGUAUCGAGC------------------------------------------------------------------------------------------------------------------------------- 1        19
---------------------------AAUUAUGCCAGUGAACCUCU--------------------------------------------------------------------------------------------------------- 1        20

    UA       A    A     C                         U       AGC  U     U   A  CC
UUCA  aGGAUGG UGGU UCGAG AcaAUUAUGCCAGUGAAC-CUCUg GUGUAUG   UG CUCUU AAA AU  \
AAGU  UCCUACC ACCA AGUUC uGUUAAUAUGGUCACUUG GAgAC CACAUAC   AC-GAGGG UUU UA  A
    cA       A    C     a                  U      C       AUA        U   A  AU
```

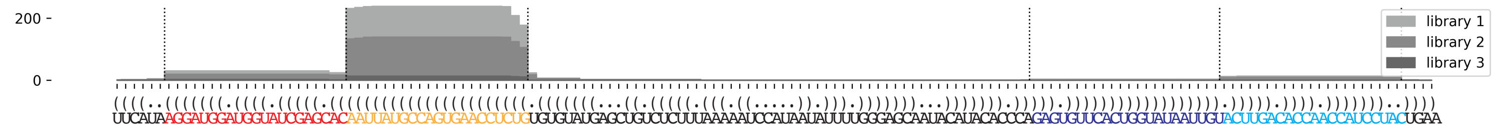

P. polycephalum ppo-mir-1226

..((((((((((.((((((((((((((((((.(((.(((.(.(((((((.((((((..((((((((((((((((((((((((((((((.(.(..((((.(((((((((..((((.(((.(((((((((.(((((((....)))))))..)))..))))))).))).))))..))))..................))))).)))).).).))))))))))))))))))..))).)))))).))))))))))))).).))).))))))))))))))))))))).))))))..))))) −184.64 kcal/mol

CCCCCUCUCAaAGAGUACCUUACUUCAGACcCGAUUGCGCGCGGUACUGAGGUAUGUCUCGUGCGGUACAUGCGAGCGCGUCGCGGUCGGUUGCGGUCGUCGUGUUAUGAUGAACUUCAUGUAUCGUAUGUCGCGUACUCGUGACACAACGCAAUGCGUGCAGUCGCGUUCAACAAUAUACUCACUUCAGCUGACAGUGCGUCUCUUGCGUGUGUGCUCGUGUGCUUGCGCGCGCGUAUGUACCCAGUGGCACACGCUAUCGGuCUGAAGUAAGGUACCCUUUgAAAGACGG count length

Libraries combined Precision: [total = 98.0% | 5p-arm = 47.6% | 3p-arm = 99.2%]

Read	count	length
UCUGAAGUAAGGUACCCUUUG	2153	21
UCUGAAGUAAGGUACCCUUU	557	20
UCUGAAGUAAGGUACCCU	420	18
UCUGAAGUAAGGUACCCUU	399	19
UCUGAAGUAAGGUACCCUUUGA	61	22
AAGAGUACCUUACUUCAGACC	26	21
UUGCGCGCGGUACUGAGGU	14	19
AAGAGUACCUUACUUCAGACCC	10	22
UGAAGUAAGGUACCCUUUG	7	19
UUGCGCGCGGUACUGAGGUA	13	20
UUGCGCGCGGUACUGAGG	4	18
AUGUACCCAGUGGCACACG	2	19
CCCAGUGGCACACGCUAUCGG	6	21
CAGUGGCACACGCUAUCGGUC	3	21
AAGAGUACCUUACUUCAGAC	2	20
UCGUGCGGUACAUGCGAGC	1	19
UUGCGCGCGCGUAUGUACC	1	19
CCAGUGGCACACGCUAUCGGU	2	21
AGUGGCACACGCUAUCGGUC	3	20
GUGGCACACGCUAUCGGUC	3	19
UGAAGUAAGGUACCCUUU	1	18
AAGAGUACCUUACUUCAGA	2	19
CCCGAUUGCGCGCGGUACUGAGG	1	23
CGAUUGCGCGCGGUACUGA	1	19
GAUUGCGCGCGGUACUGAGGUA	2	22
UUGCGCGCGGUACUGAGGUAU	1	21
UAUGUCUCGUGCGGUACAUG	1	20
UAUGUCUCGUGCGGUACAUGCGA	1	23
AUGUCUCGUGCGGUACAUGCGAG	1	23
UGUCUCGUGCGGUACAUG	2	18
UGUCUCGUGCGGUACAUGCGAGCGC	1	25
UGUCUCGUGCGGUACAUGCG	1	20
UGCUUGCGCGCGCGUAUGUA	1	20
CACACGCUAUCGGUCUGAAG	1	20
UCUGAAGUAAGGUACCCUUUGAA	3	23

Library = 1 Precision: [total = 97.8% | 5p-arm = 53.1% | 3p-arm = 98.9%]

Read	count	length
UCUGAAGUAAGGUACCCUUUG	763	21
UCUGAAGUAAGGUACCCUUU	218	20
UCUGAAGUAAGGUACCCU	201	18
UCUGAAGUAAGGUACCCUU	172	19
UCUGAAGUAAGGUACCCUUUGA	23	22
AAGAGUACCUUACUUCAGACC	10	21
UUGCGCGCGGUACUGAGGU	8	19
AAGAGUACCUUACUUCAGACCC	6	22
UGAAGUAAGGUACCCUUUG	5	19
UUGCGCGCGGUACUGAGGUA	3	20
UUGCGCGCGGUACUGAGG	3	18
AUGUACCCAGUGGCACACG	2	19
CCCAGUGGCACACGCUAUCGG	2	21
CAGUGGCACACGCUAUCGGUC	2	21
AAGAGUACCUUACUUCAGAC	1	20
UCGUGCGGUACAUGCGAGC	1	19
UUGCGCGCGCGUAUGUACC	1	19
CCAGUGGCACACGCUAUCGGU	1	21
AGUGGCACACGCUAUCGGUC	1	20
GUGGCACACGCUAUCGGUC	1	19
UGAAGUAAGGUACCCUUU	1	18

Library = 2 Precision: [total = 98.2% | 5p-arm = 38.1% | 3p-arm = 99.5%]

Read	count	length
UCUGAAGUAAGGUACCCUUUG	1192	21
UCUGAAGUAAGGUACCCUUU	299	20
UCUGAAGUAAGGUACCCU	193	18
UCUGAAGUAAGGUACCCUU	186	19
UCUGAAGUAAGGUACCCUUUGA	32	22

P. polycephalum ppo-mir-1226 continued

```
..((((((((((.((((((((((((((((.(((.(((.(.((((((.((((((..((((((((((((((((((((((((((.(.(..((((.((((((((..((((.(((.((((((((((.(((((((....)))))))..)))..))))))).))).))))..))))................))))).)))).).).))))))))))))))))))..))).)))))).))))))))))))).).))).)))))))))))))))))))).))))))..)))))   -184.64 kcal/mol
CCCCCUCUCAaAGAGUACCUUACUUCAGACcCGAUUGCGCGGGUUACUGAGGUAUGUCUCGUGCGGUACAUGCGAGCGCGUGCGCGUGCGUUUGCGGUGUCGUGUUAUGAUGAACUUCAUGUAUCGUAUGUCGCGUACUCGUGACACAACGCAAUGCGUGCAGUGCGUUCAACAAUAUACCUCACUUCAGCUGACAGUGCGUCUCUUGCGUGUGUGCUCGUGUGCUUGCGCGCGCGUAUGUACCCAGUGGCACACGCUAUCGGuCUGAAGUAAGGUACCCUUUgAAAGAGGG   count   length
```

Libraries combined Precision: [total = 98.0% | 5p-arm = 47.6% | 3p-arm = 99.2%]

Read	count	length
AAGAGUACCUUACUUCAGACC	11	21
UUGCGCGGGUUACUGAGGUA	8	20
UUGCGCGGGUUACUGAGGU	5	19
AAGAGUACCUUACUUCAGACCC	3	22
UCUGAAGUAAGGUACCCUUUGAA	3	23
GAUUGCGCGGGUUACUGAGGUA	2	22
UGUCUCGUGCGGUACAUG	2	18
AGUGGCACACGCUAUCGGUC	2	20
UGAAGUAAGGUACCCUUUG	2	19
AAGAGUACCUUACUUCAGAC	1	20
AAGAGUACCUUACUUCAGA	1	19
CCCGAUUGCGCGGGUUACUGAGG	1	23
CGAUUGCGCGGGUUACUGA	1	19
UUGCGCGGGUUACUGAGG	1	18
UUGCGCGGGUUACUGAGGUAU	1	21
UAUGUCUCGUGCGGUACAUG	1	20
UAUGUCUCGUGCGGUACAUGCGA	1	23
AUGUCUCGUGCGGUACAUGCGAG	1	23
UGUCUCGUGCGGUACAUGCGAGCGC	1	25
UGUCUCGUGCGGUACAUGCG	1	20
UGCUUGCGCGCGCGUAUGUA	1	20
CCCAGUGGCACACGCUAUCGG	1	21
CCAGUGGCACACGCUAUCGGU	1	21
CAGUGGCACACGCUAUCGGUC	1	21
GUGGCACACGCUAUCGGUC	1	19
CACACGCUAUCGGUCUGAAG	1	20

Library = 3 Precision: [total = 97.8% | 5p-arm = 70.0% | 3p-arm = 98.7%]

Read	count	length
UCUGAAGUAAGGUACCCUUUG	198	21
UCUGAAGUAAGGUACCCUU	41	19
UCUGAAGUAAGGUACCCUUU	40	20
UCUGAAGUAAGGUACCCU	26	18
UCUGAAGUAAGGUACCCUUUGA	6	22
AAGAGUACCUUACUUCAGACC	5	21
CCCAGUGGCACACGCUAUCGG	3	21
UUGCGCGGGUUACUGAGGUA	2	20
AAGAGUACCUUACUUCAGACCC	1	22
AAGAGUACCUUACUUCAGA	1	19
UUGCGCGGGUUACUGAGGU	1	19
GUGGCACACGCUAUCGGUC	1	19

```
CC               A                  C   U   C G       A       CU                                  U C UU    G                                    AU    A   U           A        U
  CCCUC--UCAaAG GUACCUUACUUCAGACc GAU GCG G GUUACUG GGUAUGU  CGUGCG-GUA--CAUGCGAGCGCGUGCGCG G G  UGCG UGUCG-----------------UGUU  GAUG ACU CAUGUAU--CGU -UGUCGCG A
  GGGAG  AgUUUC CAUGGAAUGAAGUCuGG-CUA CGC C CGGUGAC-CCAUGUA -GCGCGC CGU  GUGUGCUCGUGUGUGCGU C C -GCGU ACAGU                 ACAA  UUGC UGA GUGCGUA  GCA  ACAGUGC C
       AA        C                  U   A A                U       G  UC                   U U U     G     CGACUUCACUCCAUAUA  CU    G   C       AC   AC       U
```

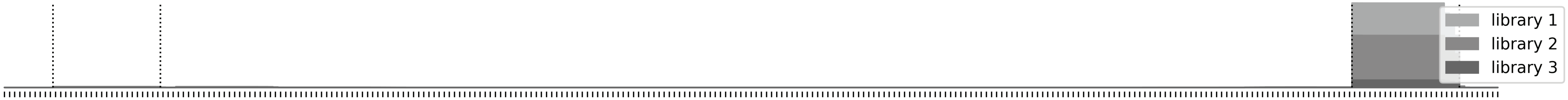


```
..((((((((((.((((((((((((((((.(((.(((.(.((((((.((((((..((((((((((((((((((((((((((.(.(..((((.((((((((..((((.(((.((((((((((.(((((((....)))))))..)))..))))))).))).))))..))))................))))).)))).).).))))))))))))))))))..))).)))))).))))))))))))).).))).)))))))))))))))))))).))))))..)))))
CCCCCUCUCAAAGAGUACCUUACUUCAGACCCGAUUGCGCGGGUUACUGAGGUAUGUCUCGUGCGGUACAUGCGAGCGCGUGCGCGUGCGUUUGCGGUGUCGUGUUAUGAUGAACUUCAUGUAUCGUAUGUCGCGUACUCGUGACACAACGCAAUGCGUGCAGUGCGUUCAACAAUAUACCUCACUUCAGCUGACAGUGCGUCUCUUGCGUGUGUGCUCGUGUGCUUGCGCGCGCGUAUGUACCCAGUGGCACACGCUAUCGGUCUGAAGUAAGGUACCCUUUGAAAGAGGG
```

P. polycephalum ppo-mir-1227

```
..((((((.((((((.(((((((.((((((((((.......(((((.(((((((.(..((((.(((.((.(((........))).)))))...))))..).)).))))).)))))...)))))))))).))))))).)))))).))..))))    -64.91 kcal/mol
AGACAGUGAGuGAGUAUUUGUGAGGUGCACaGACCAAACAACAAAAACAAAAAAAGGGCGCCAACGUGGUUUUGAAAAUUGAAGCAGUUCCCGCGCAAUAUUCUUUGUGUUUGUGCUUCUGuGCACAUCAUGGACACUCACcCAGAUUGU    count    length
Libraries combined          Precision: [ total = 97.3% | 5p-arm = 100.0% | 3p-arm = 97.2% ]
-------------------------------------------------------------------------------------------------------------------------UGCACAUCAUGGACACUCACC--------    374      21
----------UGAGUAUUUGUGAGGUGC--------------------------------------------------------------------------------------------------------------------------    13       18
--------------------------------------------------------------------------------------------------------------------------GCACAUCAUGGACACUCACC--------    9        20
----------UGAGUAUUUGUGAGGUGCACA-----------------------------------------------------------------------------------------------------------------------    3        21
----------UGAGUAUUUGUGAGGUGCA-------------------------------------------------------------------------------------------------------------------------    2        19
----------UGAGUAUUUGUGAGGUGCAC------------------------------------------------------------------------------------------------------------------------    3        20
----------UGAGUAUUUGUGAGGUGCACAG----------------------------------------------------------------------------------------------------------------------    1        22
-------------------------------------------------------------------------------------------------------------------------UGCACAUCAUGGACACUCAC---------    2        20
-----------------------------------------------------------------------------------------------------------------------UGUGCACAUCAUGGACACU------------    1        19
-----------------------------------------------------------------------------------------------------------------------UGUGCACAUCAUGGACACUC-----------    1        20
Library = 1        Precision: [ total = 98.1% | 5p-arm = 100.0% | 3p-arm = 97.9% ]
-------------------------------------------------------------------------------------------------------------------------UGCACAUCAUGGACACUCACC--------    188      21
----------UGAGUAUUUGUGAGGUGC--------------------------------------------------------------------------------------------------------------------------    9        18
--------------------------------------------------------------------------------------------------------------------------GCACAUCAUGGACACUCACC--------    4        20
----------UGAGUAUUUGUGAGGUGCACA-----------------------------------------------------------------------------------------------------------------------    2        21
----------UGAGUAUUUGUGAGGUGCA-------------------------------------------------------------------------------------------------------------------------    1        19
----------UGAGUAUUUGUGAGGUGCAC------------------------------------------------------------------------------------------------------------------------    1        20
----------UGAGUAUUUGUGAGGUGCACAG----------------------------------------------------------------------------------------------------------------------    1        22
-------------------------------------------------------------------------------------------------------------------------UGCACAUCAUGGACACUCAC---------    1        20
Library = 2        Precision: [ total = 96.7% | 5p-arm = 100.0% | 3p-arm = 96.5% ]
-------------------------------------------------------------------------------------------------------------------------UGCACAUCAUGGACACUCACC--------    165      21
----------UGAGUAUUUGUGAGGUGC--------------------------------------------------------------------------------------------------------------------------    4        18
--------------------------------------------------------------------------------------------------------------------------GCACAUCAUGGACACUCACC--------    4        20
----------UGAGUAUUUGUGAGGUGCAC------------------------------------------------------------------------------------------------------------------------    2        20
----------UGAGUAUUUGUGAGGUGCACA-----------------------------------------------------------------------------------------------------------------------    1        21
----------UGAGUAUUUGUGAGGUGCA-------------------------------------------------------------------------------------------------------------------------    1        19
-----------------------------------------------------------------------------------------------------------------------UGUGCACAUCAUGGACACU------------    1        19
-----------------------------------------------------------------------------------------------------------------------UGUGCACAUCAUGGACACUC-----------    1        20
-------------------------------------------------------------------------------------------------------------------------UGCACAUCAUGGACACUCAC---------    1        20
Library = 3        Precision: [ total = 95.5% | 5p-arm = 0% | 3p-arm = 95.5% ]
-------------------------------------------------------------------------------------------------------------------------UGCACAUCAUGGACACUCACC--------    21       21
--------------------------------------------------------------------------------------------------------------------------GCACAUCAUGGACACUCACC--------    1        20
```

```
AG         A       A        G         CCAAACA      A         A GG     C      G   G    UGA
  ACAG--UG GuGAGU UUUGUGA GUGCACaGA       ACAAA ACAAA-AA A  GCGC --AAC UG UUU    A
  UGUU   AC CACUCA AGGUACU CACGuGUCU    ----UGUUU UGUUU UU U  CGCG   UUG-AC AAG    A
      AG  c       C        A         UCG          G      C  A AA     CCC       G    UUA
```

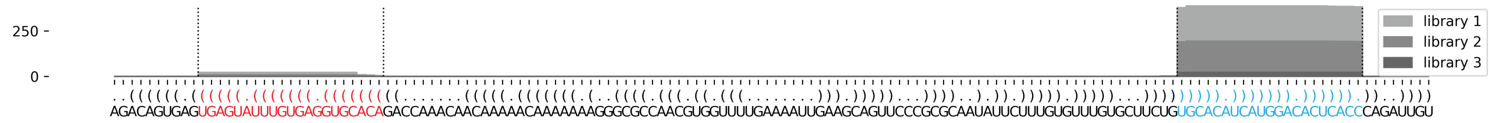

A. castellanii aca-mir-1228

```
.....((((((((((((.(((((.((((((((.((...((....)).)).)))))))).))))).)))))))))))).....    -56.3 kcal/mol
CCAUUUCAUGUUUAGUGCCUAUGUCCCCUUCUGUCAUACCUAUUGGGGAGAGAAGGGGUCAUAGACACUAAACAUGACUCUG    count    length
Libraries combined          Precision: [ total = 94.8% | 5p-arm = 94.8% | 3p-arm = 94.9% ]
----------UUUAGUGCCUAUGUCCCCUUC---------------------------------------------------    87       21
-----------------------------------------------------AGGGGUCAUAGACACUAAACA--------    40       21
-----------------------------------------------------AGGGGUCAUAGACACUAAAC---------    16       20
---------GUUUAGUGCCUAUGUCCCCUUCU--------------------------------------------------    1        23
---------GUUUAGUGCCUAUGUCCCCUUC---------------------------------------------------    1        22
----------UUUAGUGCCUAUGUCCCCUU----------------------------------------------------    2        20
----------UUUAGUGCCUAUGUCCCCUUCUGUCAUACCUA----------------------------------------    1        32
----------UUUAGUGCCUAUGUCCCCUUCUGUCAUACC------------------------------------------    1        30
----------UUUAGUGCCUAUGUCCCCUUCUG-------------------------------------------------    2        23
-----------UUAGUGCCUAUGUCCCCUUC---------------------------------------------------    1        20
-------------------------------------ACCUAUUGGGGAGAGAAGGGGUCAUAGACAC--------------    1        31
-----------------------------------------AUUGGGGAGAGAAGGGGUCAUAGACACUAAACA--------    1        33
------------------------------------------UUGGGGAGAGAAGGGGUCAUAGACACUAAACA--------    1        32
Library = 1        Precision: [ total = 93.0% | 5p-arm = 92.3% | 3p-arm = 93.9% ]
----------UUUAGUGCCUAUGUCCCCUUC---------------------------------------------------    58       21
-----------------------------------------------------AGGGGUCAUAGACACUAAACA--------    32       21
-----------------------------------------------------AGGGGUCAUAGACACUAAAC---------    14       20
---------GUUUAGUGCCUAUGUCCCCUUCU--------------------------------------------------    1        23
---------GUUUAGUGCCUAUGUCCCCUUC---------------------------------------------------    1        22
----------UUUAGUGCCUAUGUCCCCUU----------------------------------------------------    2        20
----------UUUAGUGCCUAUGUCCCCUUCUGUCAUACCUA----------------------------------------    1        32
----------UUUAGUGCCUAUGUCCCCUUCUGUCAUACC------------------------------------------    1        30
-----------UUAGUGCCUAUGUCCCCUUC---------------------------------------------------    1        20
-------------------------------------ACCUAUUGGGGAGAGAAGGGGUCAUAGACAC--------------    1        31
-----------------------------------------AUUGGGGAGAGAAGGGGUCAUAGACACUAAACA--------    1        33
------------------------------------------UUGGGGAGAGAAGGGGUCAUAGACACUAAACA--------    1        32
Library = 2        Precision: [ total = 100.0% | 5p-arm = 100.0% | 3p-arm = 100.0% ]
----------UUUAGUGCCUAUGUCCCCUUC---------------------------------------------------    26       21
-----------------------------------------------------AGGGGUCAUAGACACUAAACA--------    5        21
-----------------------------------------------------AGGGGUCAUAGACACUAAAC---------    1        20
----------UUUAGUGCCUAUGUCCCCUUCUG-------------------------------------------------    2        23
Library = 3        Precision: [ total = 100.0% | 5p-arm = 100.0% | 3p-arm = 100.0% ]
----------UUUAGUGCCUAUGUCCCCUUC---------------------------------------------------    3        21
-----------------------------------------------------AGGGGUCAUAGACACUAAACA--------    3        21
-----------------------------------------------------AGGGGUCAUAGACACUAAAC---------    1        20
```

```
CCAUU                C      U          G   AUA   U
     UCAUGUUUAGUG CUAUG CCCCUUCU UC    CC  A
     AGUACAAAUCAC GAUAC GGGGAAGA AG --GG  U
GUCUC                A      U          G   G     U
```

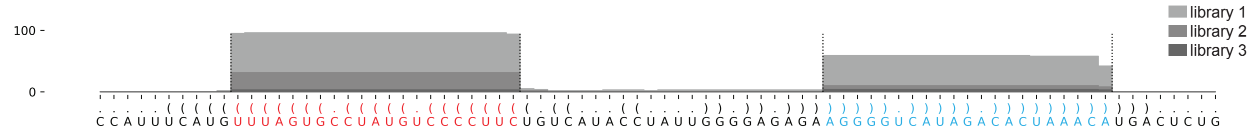

A. lenticulata ale-mir-1228-P1

```
.....((((((((((((.(((((.((((((((((..((.((((........)))))).)))))))))).))))).))))))))))))).....   -58.42 kcal/mol
AUUCUUCAUGUUUAGUGCCUAUGUCCUCUUCACUCAUGAUCGCCAUCACGAGCGGCACAGUGAAGAGGUCAUAGACACUAAACAUGACUUUU   count    length
Libraries combined        Precision: [ total = 99.4% | 5p-arm = 99.3% | 3p-arm = 100.0% ]
----------UUUAGUGCCUAUGUCCUCUUCA------------------------------------------------------------   101      22
----------UUUAGUGCCUAUGUCCUCUUC-------------------------------------------------------------   116      21
----------UUUAGUGCCUAUGUCCUC----------------------------------------------------------------   91       18
----------UUUAGUGCCUAUGUCCUCUU--------------------------------------------------------------   94       20
----------UUUAGUGCCUAUGUCCUCU---------------------------------------------------------------   48       19
--------------------------------------------------------------AAGAGGUCAUAGACACUAAACA--------   9        22
-----------UUAGUGCCUAUGUCCUCUUCA------------------------------------------------------------   2        21
-----------UUAGUGCCUAUGUCCUCUUC-------------------------------------------------------------   1        20
--------------------------------------------------------------AAGAGGUCAUAGACACUAAAC---------   4        21
Library = 1        Precision: [ total = 100.0% | 5p-arm = 100.0% | 3p-arm = 100.0% ]
----------UUUAGUGCCUAUGUCCUCUUCA------------------------------------------------------------   39       22
----------UUUAGUGCCUAUGUCCUCUUC-------------------------------------------------------------   31       21
----------UUUAGUGCCUAUGUCCUC----------------------------------------------------------------   25       18
----------UUUAGUGCCUAUGUCCUCUU--------------------------------------------------------------   22       20
----------UUUAGUGCCUAUGUCCUCU---------------------------------------------------------------   10       19
--------------------------------------------------------------AAGAGGUCAUAGACACUAAACA--------   3        22
Library = 2        Precision: [ total = 98.6% | 5p-arm = 98.6% | 3p-arm = 100.0% ]
----------UUUAGUGCCUAUGUCCUCUUC-------------------------------------------------------------   59       21
----------UUUAGUGCCUAUGUCCUCUU--------------------------------------------------------------   45       20
----------UUUAGUGCCUAUGUCCUC----------------------------------------------------------------   40       18
----------UUUAGUGCCUAUGUCCUCUUCA------------------------------------------------------------   37       22
----------UUUAGUGCCUAUGUCCUCU---------------------------------------------------------------   28       19
-----------UUAGUGCCUAUGUCCUCUUCA------------------------------------------------------------   2        21
--------------------------------------------------------------AAGAGGUCAUAGACACUAAACA--------   2        22
--------------------------------------------------------------AAGAGGUCAUAGACACUAAAC---------   2        21
-----------UUAGUGCCUAUGUCCUCUUC-------------------------------------------------------------   1        20
Library = 3        Precision: [ total = 100.0% | 5p-arm = 100.0% | 3p-arm = 100.0% ]
----------UUUAGUGCCUAUGUCCUCUU--------------------------------------------------------------   27       20
----------UUUAGUGCCUAUGUCCUC----------------------------------------------------------------   26       18
----------UUUAGUGCCUAUGUCCUCUUC-------------------------------------------------------------   26       21
----------UUUAGUGCCUAUGUCCUCUUCA------------------------------------------------------------   25       22
----------UUUAGUGCCUAUGUCCUCU---------------------------------------------------------------   10       19
--------------------------------------------------------------AAGAGGUCAUAGACACUAAACA--------   4        22
--------------------------------------------------------------AAGAGGUCAUAGACACUAAAC---------   2        21
AUUCU           C     U          CA  A    CAU
     UCAUGUUUAGUG CUAUG CCUCUUCACU  UG UCGC   C
     AGUACAAAUCAC GAUAC GGAGAAGUGA -AC-GGCG   A
UUUUC           A     U          C        AGC
```

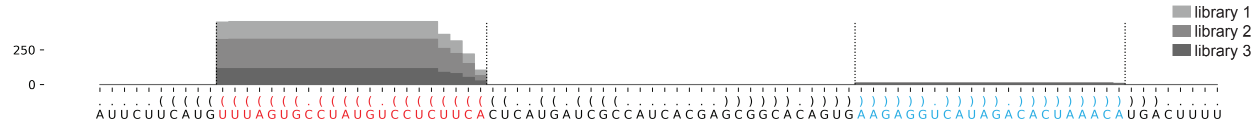

A. lenticulata ale-mir-1229

```
..(.((.(((((((((((((((((.((((((((............(((...(.((.(((((((((((((.((.......)).))))))))).)))).)).)...)))...)))))))).))))))))))))))))).)).)..   -96.8 kcal/mol
ACGAGGAGAGaGCGGGCUGGAGAUGAAGCGGaCGACAGAAGAGAAGACGACGACGCCAGCGUGGCGAUGAUGAGGACGACGACAUCGCCGCUGCUGCCGCCACAGUCCGAGUCcGCUUGGUCUUCAGCUCGCUCuCACCGCAA   count   length
Libraries combined          Precision: [ total = 96.1% | 5p-arm = 95.9% | 3p-arm = 100.0% ]
----------AGCGGGCUGGAGAUGAAGCGGA---------------------------------------------------------------------------------------------------------------   55   22
----------AGCGGGCUGGAGAUGAAGCGG----------------------------------------------------------------------------------------------------------------   6    21
----------AGCGGGCUGGAGAUGAAGC------------------------------------------------------------------------------------------------------------------   6    19
----------AGCGGGCUGGAGAUGAAGCGGAC--------------------------------------------------------------------------------------------------------------   2    23
-------GAGAGCGGGCUGGAGAUGAAGCGGACGAC-----------------------------------------------------------------------------------------------------------   1    29
-------GAGAGCGGGCUGGAGAUGAAGCGGACG-------------------------------------------------------------------------------------------------------------   1    27
----------AGCGGGCUGGAGAUGAAGCG-----------------------------------------------------------------------------------------------------------------   1    20
-----------GCGGGCUGGAGAUGAAGCGG----------------------------------------------------------------------------------------------------------------   1    20
-----------------------------------------------------------------------------------------------------------------CGCUUGGUCUUCAGCUCGCUCU--------   3    22
Library = 1        Precision: [ total = 100.0% | 5p-arm = 100.0% | 3p-arm = 0% ]
----------AGCGGGCUGGAGAUGAAGCGGA---------------------------------------------------------------------------------------------------------------   23   22
----------AGCGGGCUGGAGAUGAAGCGG----------------------------------------------------------------------------------------------------------------   2    21
----------AGCGGGCUGGAGAUGAAGC------------------------------------------------------------------------------------------------------------------   1    19
----------AGCGGGCUGGAGAUGAAGCGGAC--------------------------------------------------------------------------------------------------------------   1    23
Library = 2        Precision: [ total = 92.0% | 5p-arm = 91.7% | 3p-arm = 100.0% ]
----------AGCGGGCUGGAGAUGAAGCGGA---------------------------------------------------------------------------------------------------------------   16   22
----------AGCGGGCUGGAGAUGAAGCGG----------------------------------------------------------------------------------------------------------------   3    21
----------AGCGGGCUGGAGAUGAAGC------------------------------------------------------------------------------------------------------------------   2    19
-------GAGAGCGGGCUGGAGAUGAAGCGGACGAC-----------------------------------------------------------------------------------------------------------   1    29
-------GAGAGCGGGCUGGAGAUGAAGCGGACG-------------------------------------------------------------------------------------------------------------   1    27
----------AGCGGGCUGGAGAUGAAGCG-----------------------------------------------------------------------------------------------------------------   1    20
-----------------------------------------------------------------------------------------------------------------CGCUUGGUCUUCAGCUCGCUCU--------   1    22
Library = 3        Precision: [ total = 95.8% | 5p-arm = 95.5% | 3p-arm = 100.0% ]
----------AGCGGGCUGGAGAUGAAGCGGA---------------------------------------------------------------------------------------------------------------   16   22
----------AGCGGGCUGGAGAUGAAGC------------------------------------------------------------------------------------------------------------------   3    19
-----------------------------------------------------------------------------------------------------------------CGCUUGGUCUUCAGCUCGCUCU--------   2    22
----------AGCGGGCUGGAGAUGAAGCGGAC--------------------------------------------------------------------------------------------------------------   1    23
----------AGCGGGCUGGAGAUGAAGCGG----------------------------------------------------------------------------------------------------------------   1    21
-----------GCGGGCUGGAGAUGAAGCGG----------------------------------------------------------------------------------------------------------------   1    20

AC A  A                 G        GACAGAAGAGAA   GAC A  C              A  AGG
  G GG GAGaGCGGGCUGGAGAU AAGCGGaC            GAC   G CG CAGC-GUGGCGAUG UG   \
  C CC CuCUCGCUCGACUUCUG UUCGcCUG   ---------CUG   C GC GUCG CGCCGCUAC GC   A
AA G  A                 G        AGC            ACA C  C    U         A  AGC
```

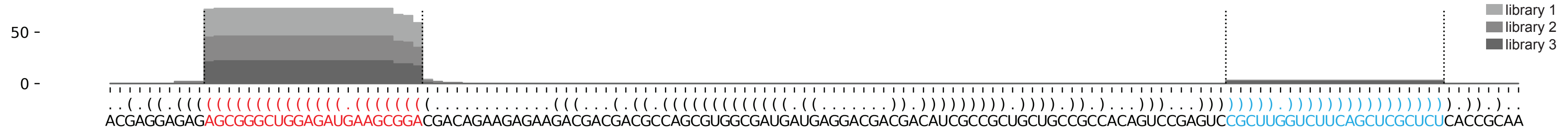

A. lenticulata ale-mir-1228-P2

```
...((.(((((((((((.(((((.((((((((((..((.((((........)))))).))))))))))).))))).)))))))))))))....   -58.46 kcal/mol
AUUGUUCAUGuUUAGUGCCUAUGUCCUCUUCaCUCAUGAUCGCCAUCACGAGCGGCACAGUGaAGAGGUCAUAGACACUAAACaUGACUUUU   count    length
Libraries combined          Precision: [ total = 99.4% | 5p-arm = 99.3% | 3p-arm = 100.0% ]
----------UUUAGUGCCUAUGUCCUCUUCA------------------------------------------------------------   101      22
----------UUUAGUGCCUAUGUCCUCUUC-------------------------------------------------------------   116      21
----------UUUAGUGCCUAUGUCCUC----------------------------------------------------------------   91       18
----------UUUAGUGCCUAUGUCCUCUU--------------------------------------------------------------   94       20
----------UUUAGUGCCUAUGUCCUCU---------------------------------------------------------------   48       19
--------------------------------------------------------------AAGAGGUCAUAGACACUAAACA--------   9        22
-----------UUAGUGCCUAUGUCCUCUUCA------------------------------------------------------------   2        21
-----------UUAGUGCCUAUGUCCUCUUC-------------------------------------------------------------   1        20
--------------------------------------------------------------AAGAGGUCAUAGACACUAAAC---------   4        21
Library = 1        Precision: [ total = 100.0% | 5p-arm = 100.0% | 3p-arm = 100.0% ]
----------UUUAGUGCCUAUGUCCUCUUCA------------------------------------------------------------   39       22
----------UUUAGUGCCUAUGUCCUCUUC-------------------------------------------------------------   31       21
----------UUUAGUGCCUAUGUCCUC----------------------------------------------------------------   25       18
----------UUUAGUGCCUAUGUCCUCUU--------------------------------------------------------------   22       20
----------UUUAGUGCCUAUGUCCUCU---------------------------------------------------------------   10       19
--------------------------------------------------------------AAGAGGUCAUAGACACUAAACA--------   3        22
Library = 2        Precision: [ total = 98.6% | 5p-arm = 98.6% | 3p-arm = 100.0% ]
----------UUUAGUGCCUAUGUCCUCUUC-------------------------------------------------------------   59       21
----------UUUAGUGCCUAUGUCCUCUU--------------------------------------------------------------   45       20
----------UUUAGUGCCUAUGUCCUC----------------------------------------------------------------   40       18
----------UUUAGUGCCUAUGUCCUCUUCA------------------------------------------------------------   37       22
----------UUUAGUGCCUAUGUCCUCU---------------------------------------------------------------   28       19
-----------UUAGUGCCUAUGUCCUCUUCA------------------------------------------------------------   2        21
--------------------------------------------------------------AAGAGGUCAUAGACACUAAACA--------   2        22
--------------------------------------------------------------AAGAGGUCAUAGACACUAAAC---------   2        21
-----------UUAGUGCCUAUGUCCUCUUC-------------------------------------------------------------   1        20
Library = 3        Precision: [ total = 100.0% | 5p-arm = 100.0% | 3p-arm = 100.0% ]
----------UUUAGUGCCUAUGUCCUCUU--------------------------------------------------------------   27       20
----------UUUAGUGCCUAUGUCCUC----------------------------------------------------------------   26       18
----------UUUAGUGCCUAUGUCCUCUUC-------------------------------------------------------------   26       21
----------UUUAGUGCCUAUGUCCUCUUCA------------------------------------------------------------   25       22
----------UUUAGUGCCUAUGUCCUCU---------------------------------------------------------------   10       19
--------------------------------------------------------------AAGAGGUCAUAGACACUAAACA--------   4        22
--------------------------------------------------------------AAGAGGUCAUAGACACUAAAC---------   2        21
```

```
 AUU   U              C      U           CA   A     CAU
    GU CAUGuUUAGUG CUAUG CCUCUUCaCU  UG UCGC    C
    CA-GUaCAAAUCAC GAUAC GGAGAaGUGA -AC-GGCG    A
UUUU                A      U           C          AGC
```

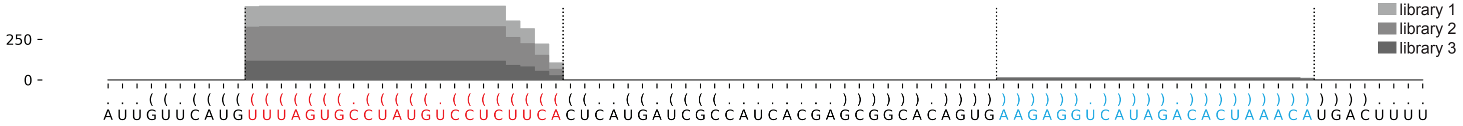

A. lenticulata ale-mir-1230-P1

```
..((((((((.(((..((((((((((((.(((((((.((.((....).))))))))).)))))))))))..))).)))))..)))   -58.43 kcal/mol
CACCCUUUCCcACGCUCUCGGGAGACGAAGcACAAAGCGCAUAAGGGCUUGUGCuCCGUCUCUUGAGCUCGUCGgAAACUGGG   count    length
Libraries combined          Precision: [ total = 93.4% | 5p-arm = 99.2% | 3p-arm = 61.1% ]
----------CACGCUCUCGGGAGACGAAGC----------------------------------------------------   398      21
----------CACGCUCUCGGGAGACGAAG-----------------------------------------------------   158      20
----------CACGCUCUCGGGAGACGAAGCA---------------------------------------------------   61       22
------------------------------------------------------UCCGUCUCUUGAGCUCGUCGG--------   56       21
-----------------------------------------------------CUCCGUCUCUUGAGCUCGUCGG--------   16       22
-----------------------------------------------------CUCCGUCUCUUGAGCUCGUC----------   13       20
------------------------------------------------------UCCGUCUCUUGAGCUCGUCG---------   8        20
----------CACGCUCUCGGGAGACGA-------------------------------------------------------   4        18
-----------------------------------------------------CUCCGUCUCUUGAGCUCGUCG---------   3        21
-----------------------------------------------------CUCCGUCUCUUGAGCUCGU-----------   4        19
----------CACGCUCUCGGGAGACGAAGCAC--------------------------------------------------   2        23
----------------------------------------AUAAGGGCUUGUGCUCCGUCUCU--------------------   1        23
------------------------------------------AAGGGCUUGUGCUCCGUCUCUUGAGC---------------   1        26
------------------------------------------------UUGUGCUCCGUCUCUUGAGCUCGUC----------   1        25
-----------------------------------------------------CUCCGUCUCUUGAGCUCGUCGGA-------   2        23
------------------------------------------------------UCCGUCUCUUGAGCUCGU-----------   2        18
----------CACGCUCUCGGGAGACGAA------------------------------------------------------   7        19
-----------ACGCUCUCGGGAGACGAAGC----------------------------------------------------   4        20
----------------CUCGGGAGACGAAGCACA-------------------------------------------------   1        18
-------------------------------ACAAAGCGCAUAAGGGCUUGUGCUCCGUCUCU--------------------   1        32
----------------------------------------AUAAGGGCUUGUGCUCCGUCUCUUGAGCUCGUC----------   1        33
-------------------------------------------------UGUGCUCCGUCUCUUGAGCUC-------------   1        21
------------------------------------------------------UCCGUCUCUUGAGCUCGUC----------   3        19
Library = 1        Precision: [ total = 92.1% | 5p-arm = 100.0% | 3p-arm = 48.6% ]
----------CACGCUCUCGGGAGACGAAGC----------------------------------------------------   140      21
----------CACGCUCUCGGGAGACGAAG-----------------------------------------------------   44       20
----------CACGCUCUCGGGAGACGAAGCA---------------------------------------------------   17       22
------------------------------------------------------UCCGUCUCUUGAGCUCGUCGG--------   13       21
-----------------------------------------------------CUCCGUCUCUUGAGCUCGUCGG--------   6        22
-----------------------------------------------------CUCCGUCUCUUGAGCUCGUC----------   5        20
------------------------------------------------------UCCGUCUCUUGAGCUCGUCG---------   4        20
----------CACGCUCUCGGGAGACGA-------------------------------------------------------   2        18
-----------------------------------------------------CUCCGUCUCUUGAGCUCGUCG---------   2        21
-----------------------------------------------------CUCCGUCUCUUGAGCUCGU-----------   2        19
----------CACGCUCUCGGGAGACGAAGCAC--------------------------------------------------   1        23
----------------------------------------AUAAGGGCUUGUGCUCCGUCUCU--------------------   1        23
------------------------------------------AAGGGCUUGUGCUCCGUCUCUUGAGC---------------   1        26
------------------------------------------------UUGUGCUCCGUCUCUUGAGCUCGUC----------   1        25
-----------------------------------------------------CUCCGUCUCUUGAGCUCGUCGGA-------   1        23
------------------------------------------------------UCCGUCUCUUGAGCUCGU-----------   1        18
Library = 2        Precision: [ total = 95.0% | 5p-arm = 98.6% | 3p-arm = 68.4% ]
----------CACGCUCUCGGGAGACGAAGC----------------------------------------------------   164      21
----------CACGCUCUCGGGAGACGAAG-----------------------------------------------------   77       20
----------CACGCUCUCGGGAGACGAAGCA---------------------------------------------------   34       22
------------------------------------------------------UCCGUCUCUUGAGCUCGUCGG--------   21       21
-----------------------------------------------------CUCCGUCUCUUGAGCUCGUCGG--------   5        22
----------CACGCUCUCGGGAGACGAA------------------------------------------------------   4        19
-----------------------------------------------------CUCCGUCUCUUGAGCUCGUC----------   4        20
-----------ACGCUCUCGGGAGACGAAGC----------------------------------------------------   3        20
------------------------------------------------------UCCGUCUCUUGAGCUCGUC----------   3        19
----------CACGCUCUCGGGAGACGAAGCAC--------------------------------------------------   1        23
----------CACGCUCUCGGGAGACGA-------------------------------------------------------   1        18
```

A. lenticulata ale-mir-1230-P1 continued

```
..((((((((.(((..(((((((((((.(((((((.((.(....).))))))))).)))))))))))..))).)))))..)))   -58.43 kcal/mol
CACCCUUUCCcACGCUCUCGGGAGACGAAGcACAAAGCGCAUAAGGGCUUGUGCuCCGUCUCUUGAGCUCGUCGgAAACUGGG   count    length
----------------CUCGGGAGACGAAGCACA-------------------------------------------------   1        18
-------------------------------------------------UGUGCUCCGUCUCUUGAGCUC-------------   1        21
-----------------------------------------------------CUCCGUCUCUUGAGCUCGUCG---------   1        21
-----------------------------------------------------CUCCGUCUCUUGAGCUCGUCGGA-------   1        23
------------------------------------------------------UCCGUCUCUUGAGCUCGUCG---------   1        20
------------------------------------------------------UCCGUCUCUUGAGCUCGU-----------   1        18
Library = 3        Precision: [ total = 92.4% | 5p-arm = 99.3% | 3p-arm = 65.8% ]
----------CACGCUCUCGGGAGACGAAGC----------------------------------------------------   94       21
----------CACGCUCUCGGGAGACGAAG-----------------------------------------------------   37       20
------------------------------------------------------UCCGUCUCUUGAGCUCGUCGG--------   22       21
----------CACGCUCUCGGGAGACGAAGCA---------------------------------------------------   10       22
-----------------------------------------------------CUCCGUCUCUUGAGCUCGUCGG--------   5        22
-----------------------------------------------------CUCCGUCUCUUGAGCUCGUC----------   4        20
----------CACGCUCUCGGGAGACGAA------------------------------------------------------   3        19
------------------------------------------------------UCCGUCUCUUGAGCUCGUCG---------   3        20
-----------------------------------------------------CUCCGUCUCUUGAGCUCGU-----------   2        19
----------CACGCUCUCGGGAGACGA-------------------------------------------------------   1        18
-----------ACGCUCUCGGGAGACGAAGC----------------------------------------------------   1        20
-------------------------------ACAAAGCGCAUAAGGGCUUGUGCUCCGUCUCU--------------------   1        32
----------------------------------------AUAAGGGCUUGUGCUCCGUCUCUUGAGCUCGUC----------   1        33
```

```
CA          c   CU           A       A  G A
  CCC--UUUCC ACG  CUCGGGAGACG AGcACAA GC C U
  GGG  AAAgG UGC  GAGUUCUCUGC uCGUGUU-CG G A
     UC     C   UC           C          G A
```

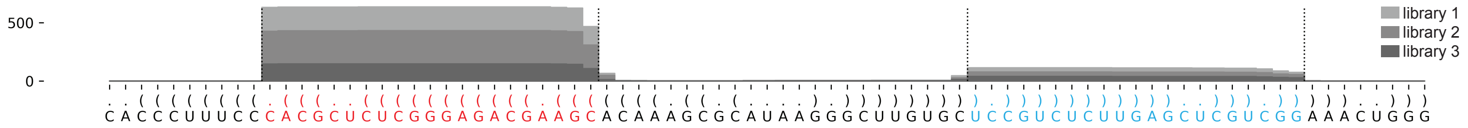

A. lenticulata ale-mir-1230-P2

```
..((((((((.(((..(((((((((((.(((((((.((((((....))))))))))))).))))))))))).))).)))))..)))       -65.24 kcal/mol
CACCCUUUCCcACGCUCUCGGGAGACGAAGcACAAAGCCCUUAUAUAAGGGCUUGUGCuCCGUCUCUUGAGCUCGUCGgAAACUGGG       count    length
Libraries combined         Precision: [ total = 93.3% | 5p-arm = 98.9% | 3p-arm = 61.6% ]
----------CACGCUCUCGGGAGACGAAGC--------------------------------------------------------       398      21
----------CACGCUCUCGGGAGACGAAG---------------------------------------------------------       158      20
----------CACGCUCUCGGGAGACGAAGCA-------------------------------------------------------       61       22
----------------------------------------------------------UCCGUCUCUUGAGCUCGUCGG--------       56       21
---------------------------------------------------------CUCCGUCUCUUGAGCUCGUCGG--------       16       22
---------------------------------------------------------CUCCGUCUCUUGAGCUCGUC----------       13       20
----------------------------------------------------------UCCGUCUCUUGAGCUCGUCG---------       8        20
----------CACGCUCUCGGGAGACGA-----------------------------------------------------------       4        18
---------------------------------------------------------CUCCGUCUCUUGAGCUCGUCG---------       3        21
---------------------------------------------------------CUCCGUCUCUUGAGCUCGU-----------       4        19
----------CACGCUCUCGGGAGACGAAGCAC------------------------------------------------------       2        23
--------------------------------------------AUAAGGGCUUGUGCUCCGUCUCU--------------------       1        23
----------------------------------------------AAGGGCUUGUGCUCCGUCUCUUGAGC---------------       1        26
----------------------------------------------------UUGUGCUCCGUCUCUUGAGCUCGUC----------       1        25
---------------------------------------------------------CUCCGUCUCUUGAGCUCGUCGGA-------       2        23
----------------------------------------------------------UCCGUCUCUUGAGCUCGU-----------       2        18
----------CACGCUCUCGGGAGACGAA----------------------------------------------------------       7        19
-----------ACGCUCUCGGGAGACGAAGC--------------------------------------------------------       4        20
----------------CUCGGGAGACGAAGCACA-----------------------------------------------------       1        18
----------------CUCGGGAGACGAAGCACAAAGCCC-----------------------------------------------       1        24
---------------------GAGACGAAGCACAAAGCCC-----------------------------------------------       1        19
--------------------------------------------AUAAGGGCUUGUGCUCCGUCUCUUGAGCUCGUC----------       1        33
-----------------------------------------------------UGUGCUCCGUCUCUUGAGCUC-------------       1        21
----------------------------------------------------------UCCGUCUCUUGAGCUCGUC----------       3        19
Library = 1        Precision: [ total = 92.1% | 5p-arm = 100.0% | 3p-arm = 48.6% ]
----------CACGCUCUCGGGAGACGAAGC--------------------------------------------------------       140      21
----------CACGCUCUCGGGAGACGAAG---------------------------------------------------------       44       20
----------CACGCUCUCGGGAGACGAAGCA-------------------------------------------------------       17       22
----------------------------------------------------------UCCGUCUCUUGAGCUCGUCGG--------       13       21
---------------------------------------------------------CUCCGUCUCUUGAGCUCGUCGG--------       6        22
---------------------------------------------------------CUCCGUCUCUUGAGCUCGUC----------       5        20
----------------------------------------------------------UCCGUCUCUUGAGCUCGUCG---------       4        20
----------CACGCUCUCGGGAGACGA-----------------------------------------------------------       2        18
---------------------------------------------------------CUCCGUCUCUUGAGCUCGUCG---------       2        21
---------------------------------------------------------CUCCGUCUCUUGAGCUCGU-----------       2        19
----------CACGCUCUCGGGAGACGAAGCAC------------------------------------------------------       1        23
--------------------------------------------AUAAGGGCUUGUGCUCCGUCUCU--------------------       1        23
----------------------------------------------AAGGGCUUGUGCUCCGUCUCUUGAGC---------------       1        26
----------------------------------------------------UUGUGCUCCGUCUCUUGAGCUCGUC----------       1        25
---------------------------------------------------------CUCCGUCUCUUGAGCUCGUCGGA-------       1        23
----------------------------------------------------------UCCGUCUCUUGAGCUCGU-----------       1        18
Library = 2        Precision: [ total = 94.8% | 5p-arm = 98.3% | 3p-arm = 68.4% ]
----------CACGCUCUCGGGAGACGAAGC--------------------------------------------------------       164      21
----------CACGCUCUCGGGAGACGAAG---------------------------------------------------------       77       20
----------CACGCUCUCGGGAGACGAAGCA-------------------------------------------------------       34       22
----------------------------------------------------------UCCGUCUCUUGAGCUCGUCGG--------       21       21
---------------------------------------------------------CUCCGUCUCUUGAGCUCGUCGG--------       5        22
----------CACGCUCUCGGGAGACGAA----------------------------------------------------------       4        19
---------------------------------------------------------CUCCGUCUCUUGAGCUCGUC----------       4        20
-----------ACGCUCUCGGGAGACGAAGC--------------------------------------------------------       3        20
----------------------------------------------------------UCCGUCUCUUGAGCUCGUC----------       3        19
----------CACGCUCUCGGGAGACGAAGCAC------------------------------------------------------       1        23
----------CACGCUCUCGGGAGACGA-----------------------------------------------------------       1        18
```

A. lenticulata ale-mir-1230-P2 continued

```
..((((((((.(((..(((((((((((.(((((((.((((((....))))))))))))).)))))))))))..))).)))))..)))      -65.24 kcal/mol
CACCCUUUCCcACGCUCUCGGGAGACGAAGcACAAAGCCCUUAUAUAAGGGCUUGUGCuCCGUCUCUUGAGCUCGUCGgAAACUGGG       count    length
----------------CUCGGGAGACGAAGCACA-----------------------------------------------------      1        18
----------------CUCGGGAGACGAAGCACAAAGCCC-----------------------------------------------      1        24
-----------------------------------------------------UGUGCUCCGUCUCUUGAGCUC-------------      1        21
---------------------------------------------------------CUCCGUCUCUUGAGCUCGUCG---------      1        21
---------------------------------------------------------CUCCGUCUCUUGAGCUCGUCGGA-------      1        23
----------------------------------------------------------UCCGUCUCUUGAGCUCGUCG---------      1        20
----------------------------------------------------------UCCGUCUCUUGAGCUCGU-----------      1        18
Library = 3        Precision: [ total = 92.4% | 5p-arm = 98.6% | 3p-arm = 67.6% ]
----------CACGCUCUCGGGAGACGAAGC--------------------------------------------------------      94       21
----------CACGCUCUCGGGAGACGAAG---------------------------------------------------------      37       20
----------------------------------------------------------UCCGUCUCUUGAGCUCGUCGG--------      22       21
----------CACGCUCUCGGGAGACGAAGCA-------------------------------------------------------      10       22
---------------------------------------------------------CUCCGUCUCUUGAGCUCGUCGG--------      5        22
---------------------------------------------------------CUCCGUCUCUUGAGCUCGUC----------      4        20
----------CACGCUCUCGGGAGACGAA----------------------------------------------------------      3        19
----------------------------------------------------------UCCGUCUCUUGAGCUCGUCG---------      3        20
---------------------------------------------------------CUCCGUCUCUUGAGCUCGU-----------      2        19
----------CACGCUCUCGGGAGACGA-----------------------------------------------------------      1        18
-----------ACGCUCUCGGGAGACGAAGC--------------------------------------------------------      1        20
---------------------GAGACGAAGCACAAAGCCC-----------------------------------------------      1        19
--------------------------------------------AUAAGGGCUUGUGCUCCGUCUCUUGAGCUCGUC----------      1        33
```

```
CA          c   CU          A       A      A
  CCC--UUUCC ACG  CUCGGGAGACG AGcACAA GCCCUU U
  GGG  AAAgG UGC  GAGUUCUCUGC uCGUGUU-CGGGAA A
     UC     C   UC          C              U
```

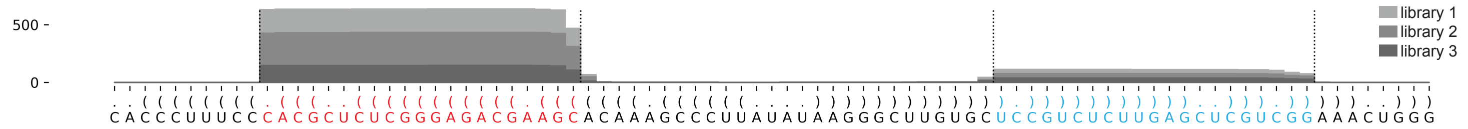

D. firmibasis dfi-mir-1177

```
....((((((.(((..((((((((((((((((((((.((((((((((((((.((((.((((......(((((.(((((..(((....))).))))))))))))))..))))))))))))))))))))))))))))))))))))))..))).))))))....    -69.66 kcal/mol
UAAAAAUAAAcCAGUUAGGGUUUAAUGGUUcUUGUUUAAAUUUUUUUUUUUUGGAUUCAAAAUUUAUUAAUCGAUUUUCCUAAUAAUUUAAAAAAUGAUUAUUUGUUAUCCAAAAAAAAGAAUUUAACAAGAgCCGUUGAGUCCUUUCUGAUuUAUUAAUU    count   length
Libraries combined           Precision: [ total = 96.2% | 5p-arm = 96.4% | 3p-arm = 95.7% ]
----------CCAGUUAGGGUUUAAUGGUUC------------------------------------------------------------------------------------------------------------------------------------    650     21
------------------------------------------------------------------------------------------------------------------------------------GCCGUUGAGUCCUUUCUGAU---------    191     20
------------------------------------------------------------------------------------------------------------------------------------GCCGUUGAGUCCUUUCUGAUU--------    137     21
------------------------------------------------------------------------------------------------------------------------------------GCCGUUGAGUCCUUUCUGA----------    41      19
----------CCAGUUAGGGUUUAAUGGUUCU---------------------------------------------------------------------------------------------------------------------------------    20      22
------------AGUUAGGGUUUAAUGGUUCUUG-------------------------------------------------------------------------------------------------------------------------------    17      22
------------------------------------------------------------------------------------------------------------------------------------GCCGUUGAGUCCUUUCUG-----------    26      18
-----------CAGUUAGGGUUUAAUGGUUC------------------------------------------------------------------------------------------------------------------------------------    3       20
----------------------------------------------------------------------------------------------------------------------------UAACAAGAGCCGUUGAGUCCU----------------    8       21
--------------------------------------------------------------------------------------------------------------------------------------CGUUGAGUCCUUUCUGAUUUA------    3       21
----------CCAGUUAGGGUUUAAUGGUUCUU--------------------------------------------------------------------------------------------------------------------------------    1       23
----------CCAGUUAGGGUUUAAUGGU------------------------------------------------------------------------------------------------------------------------------------    1       19
------------AGUUAGGGUUUAAUGGUUCUU--------------------------------------------------------------------------------------------------------------------------------    1       21
-----------------------------------------------------------------------------------------------------------------------GAAUUUAACAAGAGCCGUUG----------------------    1       20
-----------------------------------------------------------------------------------------------------------------------------AACAAGAGCCGUUGAGUCCU----------------    1       20
------------------------------------------------------------------------------------------------------------------------------------GCCGUUGAGUCCUUUCUGAUUU-------    4       22
----------CCAGUUAGGGUUUAAUGGUU-------------------------------------------------------------------------------------------------------------------------------------    3       20
-----------CAGUUAGGGUUUAAUGGUUCU---------------------------------------------------------------------------------------------------------------------------------    1       21
-------------------------------UUGUUUAAAUUUUUUUUUUUUGG-----------------------------------------------------------------------------------------------------------    3       23
------------------------------------------------------------------------------------------------------------------------------ACAAGAGCCGUUGAGUCCU----------------    1       19
---------------------------------------------------------------------------------------------------------------------------------AGAGCCGUUGAGUCCUUUCUG-----------    1       21
-----------------------------------------------------------------------------------------------------------------------------------AGCCGUUGAGUCCUUUCUGAU---------    1       21
-----------------------------------------------------------------------------------------------------------------------------------AGCCGUUGAGUCCUUUCUGAUU--------    1       22
---------------------------------------------------------------------------------------------------------------------------------------GUUGAGUCCUUUCUGAUU--------    1       18
Library = 1        Precision: [ total = 95.8% | 5p-arm = 96.1% | 3p-arm = 95.3% ]
----------CCAGUUAGGGUUUAAUGGUUC------------------------------------------------------------------------------------------------------------------------------------    238     21
------------------------------------------------------------------------------------------------------------------------------------GCCGUUGAGUCCUUUCUGAU---------    59      20
------------------------------------------------------------------------------------------------------------------------------------GCCGUUGAGUCCUUUCUGAUU--------    41      21
------------------------------------------------------------------------------------------------------------------------------------GCCGUUGAGUCCUUUCUGA----------    13      19
----------CCAGUUAGGGUUUAAUGGUUCU---------------------------------------------------------------------------------------------------------------------------------    8       22
------------AGUUAGGGUUUAAUGGUUCUUG-------------------------------------------------------------------------------------------------------------------------------    7       22
------------------------------------------------------------------------------------------------------------------------------------GCCGUUGAGUCCUUUCUG-----------    7       18
-----------CAGUUAGGGUUUAAUGGUUC------------------------------------------------------------------------------------------------------------------------------------    2       20
----------------------------------------------------------------------------------------------------------------------------UAACAAGAGCCGUUGAGUCCU----------------    2       21
--------------------------------------------------------------------------------------------------------------------------------------CGUUGAGUCCUUUCUGAUUUA------    2       21
----------CCAGUUAGGGUUUAAUGGUUCUU--------------------------------------------------------------------------------------------------------------------------------    1       23
----------CCAGUUAGGGUUUAAUGGU------------------------------------------------------------------------------------------------------------------------------------    1       19
------------AGUUAGGGUUUAAUGGUUCUU--------------------------------------------------------------------------------------------------------------------------------    1       21
-----------------------------------------------------------------------------------------------------------------------GAAUUUAACAAGAGCCGUUG----------------------    1       20
-----------------------------------------------------------------------------------------------------------------------------AACAAGAGCCGUUGAGUCCU----------------    1       20
------------------------------------------------------------------------------------------------------------------------------------GCCGUUGAGUCCUUUCUGAUUU-------    1       22
Library = 2        Precision: [ total = 95.8% | 5p-arm = 96.5% | 3p-arm = 94.5% ]
----------CCAGUUAGGGUUUAAUGGUUC------------------------------------------------------------------------------------------------------------------------------------    209     21
------------------------------------------------------------------------------------------------------------------------------------GCCGUUGAGUCCUUUCUGAU---------    61      20
------------------------------------------------------------------------------------------------------------------------------------GCCGUUGAGUCCUUUCUGAUU--------    38      21
------------------------------------------------------------------------------------------------------------------------------------GCCGUUGAGUCCUUUCUGA----------    17      19
----------CCAGUUAGGGUUUAAUGGUUCU---------------------------------------------------------------------------------------------------------------------------------    8       22
----------------------------------------------------------------------------------------------------------------------------UAACAAGAGCCGUUGAGUCCU----------------    4       21
------------------------------------------------------------------------------------------------------------------------------------GCCGUUGAGUCCUUUCUG-----------    4       18
------------AGUUAGGGUUUAAUGGUUCUUG-------------------------------------------------------------------------------------------------------------------------------    3       22
-------------------------------UUGUUUAAAUUUUUUUUUUUUGG-----------------------------------------------------------------------------------------------------------    3       23
----------CCAGUUAGGGUUUAAUGGUU-------------------------------------------------------------------------------------------------------------------------------------    1       20
-----------CAGUUAGGGUUUAAUGGUUC------------------------------------------------------------------------------------------------------------------------------------    1       20
```

D. firmibasis dfi-mir-1177 continued

```
....(((((.(((..(((((((((((((((((((.(((((((((((((.((((.((((......(((((.(((((..(((....))).))))))))))))))..))))))))))))))))))))))))))))))))))))..))).))))))....    -69.66 kcal/mol
UAAAAAUAAAcCAGUUAGGGUUUAAUGGUUcUUGUUUAAAUUUUUUUUUUUUUGGAUUCAAAAUUUAUUAAUCGAUUUUCCUAAUAAUUUAAAAAAUGAUUAUUUGUUAUCCAAAAAAAAGAAUUUAACAAGAgCCGUUGAGUCCUUUCUGAUuUAUUAAUU    count    length
-----------CAGUUAGGGUUUAAUGGUUCU----------------------------------------------------------------------------------------------------------------------------------    1    21
------------------------------------------------------------------------------------------------------------------------------------AGCCGUUGAGUCCUUUCUGAU---------    1    21
-------------------------------------------------------------------------------------------------------------------------------------GCCGUUGAGUCCUUUCUGAUUU-------    1    22
---------------------------------------------------------------------------------------------------------------------------------------CGUUGAGUCCUUUCUGAUUUA------    1    21
----------------------------------------------------------------------------------------------------------------------------------------GUUGAGUCCUUUCUGAUU--------    1    18
Library = 3         Precision: [ total = 96.8% | 5p-arm = 96.8% | 3p-arm = 96.9% ]
----------CCAGUUAGGGUUUAAUGGUUC-----------------------------------------------------------------------------------------------------------------------------------    203    21
-------------------------------------------------------------------------------------------------------------------------------------GCCGUUGAGUCCUUUCUGAU---------    71    20
-------------------------------------------------------------------------------------------------------------------------------------GCCGUUGAGUCCUUUCUGAUU--------    58    21
-------------------------------------------------------------------------------------------------------------------------------------GCCGUUGAGUCCUUUCUG-----------    15    18
-------------------------------------------------------------------------------------------------------------------------------------GCCGUUGAGUCCUUUCUGA----------    11    19
------------AGUUAGGGUUUAAUGGUUCUUG--------------------------------------------------------------------------------------------------------------------------------    7    22
----------CCAGUUAGGGUUUAAUGGUUCU----------------------------------------------------------------------------------------------------------------------------------    4    22
----------CCAGUUAGGGUUUAAUGGUU------------------------------------------------------------------------------------------------------------------------------------    2    20
-----------------------------------------------------------------------------------------------------------------------------UAACAAGAGCCGUUGAGUCCU----------------    2    21
-------------------------------------------------------------------------------------------------------------------------------------GCCGUUGAGUCCUUUCUGAUUU-------    2    22
-------------------------------------------------------------------------------------------------------------------------------ACAAGAGCCGUUGAGUCCU----------------    1    19
-----------------------------------------------------------------------------------------------------------------------------------AGAGCCGUUGAGUCCUUUCUG----------    1    21
------------------------------------------------------------------------------------------------------------------------------------AGCCGUUGAGUCCUUUCUGAUU--------    1    22
```

```
UAAA       c    UU                      U               U     U-     AUUUAU      G      CC    U
    AAUAAA CAG   AGGGUUUAAUGGUUcUUGUU AAAUUUUUUUUUUUU GGAU  CAAA       UAAUC AUUUU  UAA A
    UUAUuU GUC   UCCUGAGUUGCCgAGAACAA-UUUAAGAAAAAAAAA-CCUA  GUUU------AUUAG-UAAAA  AUU A
UUAA       A    UU                                     UU                     A-    U
```

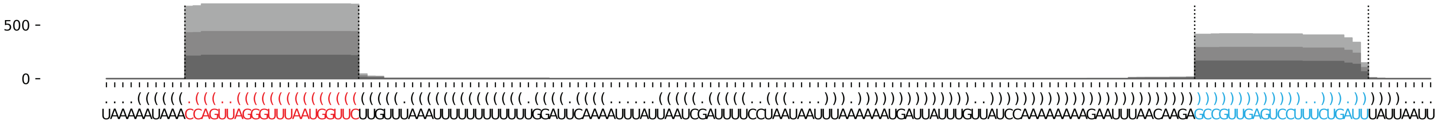

D. firmibasis dfi-mir-1189

```
(((((((((((((((((((((((((((((((((((((((((((((((((......)))))))))))))))))))))))))))))))))))))))))))))))))    -108.21 kcal/mol
GGAAGUAAAGcAUGGAAGAUUAAUGACCGCcUACCAAAGACAAAUAGGAAUCAAAUCCUAUUUGUCUUUGGUAGGcGGUCAUUAAUCUUCCAUGCuUUACUUCC    count    length
Libraries combined         Precision: [ total = 76.3% | 5p-arm = 72.2% | 3p-arm = 96.4% ]
----------CAUGGAAGAUUAAUGACCGCC-------------------------------------------------------------------------    225704    21
-----------AUGGAAGAUUAAUGACCGCCU------------------------------------------------------------------------    84956    21
---------------------------------------------------------------------------CGGUCAUUAAUCUUCCAUGCU--------    63851    21
----------CAUGGAAGAUUAAUGACCGCCU------------------------------------------------------------------------    16011    22
-----------AUGGAAGAUUAAUGACCGCC-------------------------------------------------------------------------    4437    20
---------GCAUGGAAGAUUAAUGACCGCC-------------------------------------------------------------------------    1197    22
---------GCAUGGAAGAUUAAUGACCGC--------------------------------------------------------------------------    743    21
---------------------------------------------------------------------------CGGUCAUUAAUCUUCCAUGCUU-------    1266    22
---------------------------------------------------------------------------CGGUCAUUAAUCUUCCAUGC---------    962    20
----------CAUGGAAGAUUAAUGACCG---------------------------------------------------------------------------    562    19
--------AGCAUGGAAGAUUAAUGACCGC--------------------------------------------------------------------------    520    22
----------------------------------------------------------------------------GGUCAUUAAUCUUCCAUGCUU-------    844    21
--------------------------------------------------------------------------GCGGUCAUUAAUCUUCCAUGC---------    573    21
--------AGCAUGGAAGAUUAAUGACCGCC-------------------------------------------------------------------------    411    23
----------CAUGGAAGAUUAAUGACCGC--------------------------------------------------------------------------    470    20
--------AGCAUGGAAGAUUAAUGACCG---------------------------------------------------------------------------    367    21
--------------------UAAUGACCGCCUACCAAAGAC---------------------------------------------------------------    145    21
----------------------------------------------------------------------------GGUCAUUAAUCUUCCAUGCU--------    223    20
---------GCAUGGAAGAUUAAUGACCGCCU------------------------------------------------------------------------    121    23
```

D. firmibasis dfi-mir-1189 continued

```
(((((((((((((((((((((((((((((((((((((((((((((((((......)))))))))))))))))))))))))))))))))))))))))))))))))    -108.21 kcal/mol
GGAAGUAAAGcAUGGAAGAUUAAUGACCGCcUACCAAAGACAAAUAGGAAUCAAAUCCUAUUUGUCUUUGGUAGGcGGUCAUUAAUCUUCCAUGCuUUACUUCC    count    length
-------------------------------UACCAAAGACAAAUAGGAAUCAAAUCCUAUUU-----------------------------------------    54       32
---------------------------------------------------------CUAUUUGUCUUUGGUAGGC----------------------------    87       19
--------------GAAGAUUAAUGACCGCCUACC---------------------------------------------------------------------    143      21
----------CAUGGAAGAUUAAUGACC----------------------------------------------------------------------------    57       18
-------AAGCAUGGAAGAUUAAUGACC----------------------------------------------------------------------------    63       21
-----------------------------------------------------------------------UAGGCGGUCAUUAAUCUUCCA------------    82       21
--------AGCAUGGAAGAUUAAUGACCGCCU------------------------------------------------------------------------    63       24
---------GCAUGGAAGAUUAAUGACCG---------------------------------------------------------------------------    56       20
-------------------------------------------------------UCCUAUUUGUCUUUGGUAGGC----------------------------    46       21
--------------------------------------------------------------------------GCGGUCAUUAAUCUUCCAUG----------    58       20
-----------AUGGAAGAUUAAUGACCGC--------------------------------------------------------------------------    37       19
-------------------------------UACCAAAGACAAAUAGGAAUCAAAUCCU---------------------------------------------    55       28
-----------------------------------------------------------------------UAGGCGGUCAUUAAUCUUCC-------------    67       20
-----------------------------------------------------------------------------GUCAUUAAUCUUCCAUGCU--------    39       19
------------------AUUAAUGACCGCCUACCAAAG-----------------------------------------------------------------    40       21
-------------------------------UACCAAAGACAAAUAGGAAUCAAAUC-----------------------------------------------    29       26
---------------------------------------------------------------------------CGGUCAUUAAUCUUCCAUG----------    41       19
-------------------------------UACCAAAGACAAAUAGGAAUCAAAUCCUAU-------------------------------------------    46       30
------------------------------------------------------------------UUUGGUAGGCGGUCAUUAAUC-----------------    21       21
--------------GAAGAUUAAUGACCGCCUAC----------------------------------------------------------------------    32       20
-----------------------------------------------------AAUCCUAUUUGUCUUUGGUAG------------------------------    27       21
------------------------------------------------------------------------AGGCGGUCAUUAAUCUUCCAUG----------    61       22
--------------------------------------------------------------------------GCGGUCAUUAAUCUUCCAUGCU--------    42       22
-----------------------------------------------------------AUUUGUCUUUGGUAGGCGGUC------------------------    15       21
-----------AUGGAAGAUUAAUGACCGCCUA-----------------------------------------------------------------------    24       22
-------AAGCAUGGAAGAUUAAUGACCG---------------------------------------------------------------------------    21       22
--------------GAAGAUUAAUGACCGCCU------------------------------------------------------------------------    16       18
------AAAGCAUGGAAGAUUAAUGAC-----------------------------------------------------------------------------    19       21
------------------------------------------------------------------------------UCAUUAAUCUUCCAUGCU--------    8        18
----GUAAAGCAUGGAAGAUUAAUG-------------------------------------------------------------------------------    5        21
-------------------------------UACCAAAGACAAAUAGGAAUCAAAUCCUA--------------------------------------------    9        29
--------------------------------------------------------CCUAUUUGUCUUUGGUAGGCG---------------------------    10       21
---------------------------------------------------------CUAUUUGUCUUUGGUAGGCGG--------------------------    7        21
---------------------------------------------------------------------GGUAGGCGGUCAUUAAUCUUC--------------    10       21
-----------------------------------------------------------------------------------AAUCUUCCAUGCUUUACUUCC    17       21
-------AAGCAUGGAAGAUUAAUGACCGCC-------------------------------------------------------------------------    5        24
------------------------------------------------------------------------AGGCGGUCAUUAAUCUUCCA------------    7        20
----------------------------------------------------------------------------GGUCAUUAAUCUUCCAUGCUUU------    41       22
-----------------------------------------------------------------------------GUCAUUAAUCUUCCAUGCUUU------    6        21
--------------------------------------------------------------------------------AUUAAUCUUCCAUGCUUUACU---    19       21
--------AGCAUGGAAGAUUAAUGACC----------------------------------------------------------------------------    9        20
---------GCAUGGAAGAUUAAUGACC----------------------------------------------------------------------------    5        19
-------------------------------UACCAAAGACAAAUAGGAAUCAAAUCCUAUU------------------------------------------    4        31
-------------------------------------------------------UCCUAUUUGUCUUUGGUA-------------------------------    4        18
GGAAGUAAAGCAUGGAAGAUU-----------------------------------------------------------------------------------    3        21
------AAAGCAUGGAAGAUUAAUGACCG---------------------------------------------------------------------------    5        23
-------AAGCAUGGAAGAUUAAUGAC-----------------------------------------------------------------------------    6        20
----------CAUGGAAGAUUAAUGACCGCCUA-----------------------------------------------------------------------    3        23
-----------AUGGAAGAUUAAUGACCG---------------------------------------------------------------------------    7        18
-------------------UUAAUGACCGCCUACCAAAGA----------------------------------------------------------------    5        21
-------------------UUAAUGACCGCCUACCAAAG-----------------------------------------------------------------    6        20
-------------------------------UACCAAAGACAAAUAGGAAUCAAAUCC----------------------------------------------    17       27
-------------------------------------------------------UCCUAUUUGUCUUUGGUAG------------------------------    4        19
----------------------------------------------------------UAUUUGUCUUUGGUAGGCGG--------------------------    2        20
```

D. firmibasis dfi-mir-1189 continued

```
(((((((((((((((((((((((((((((((((((((((((((((((((......)))))))))))))))))))))))))))))))))))))))))))))))))   -108.21 kcal/mol
GGAAGUAAAGcAUGGAAGAUUAAUGACCGCcUACCAAAGACAAAUAGGAAUCAAAUCCUAUUUGUCUUUGGUAGGcGGUCAUUAAUCUUCCAUGCuUUACUUCC   count   length
-----------------------------------------------------------AUUUGUCUUUGGUAGGCGGU-------------------------   2       20
----------------------------------------------------------------UCUUUGGUAGGCGGUCAUUAA-------------------   3       21
----------------------------------------------------------------UCUUUGGUAGGCGGUCAUUA--------------------   3       20
----------------------------------------------------------------UCUUUGGUAGGCGGUCAUUAAU------------------   3       22
-----------------------------------------------------------------------UAGGCGGUCAUUAAUCUUC--------------   3       19
------------------------------------------------------------------------AGGCGGUCAUUAAUCUUCCAU-----------   6       21
------------------------------------------------------------------------AGGCGGUCAUUAAUCUUCC-------------   8       19
-----UAAAGCAUGGAAGAUUAAUGAC-----------------------------------------------------------------------------   1       22
-----UAAAGCAUGGAAGAUUAAUGACCG---------------------------------------------------------------------------   3       24
------AAAGCAUGGAAGAUUAAUGACC----------------------------------------------------------------------------   3       22
---------GCAUGGAAGAUUAAUGAC-----------------------------------------------------------------------------   1       18
-------------GGAAGAUUAAUGACCGCCUAC----------------------------------------------------------------------   4       21
-------------GGAAGAUUAAUGACCGCCUACC---------------------------------------------------------------------   2       22
-------------GGAAGAUUAAUGACCGCCUA-----------------------------------------------------------------------   2       20
-------------------------------UACCAAAGACAAAUAGGAAUC----------------------------------------------------   1       21
-------------------------------UACCAAAGACAAAUAGGAAUCAAAU------------------------------------------------   4       25
--------------------------------ACCAAAGACAAAUAGGAAUCAAAUCCUAUU------------------------------------------   1       30
-------------------------------------------AUAGGAAUCAAAUCCUAUUUGUCUUUGGUAGG-----------------------------   1       32
--------------------------------------------------------CCUAUUUGUCUUUGGUAG------------------------------   2       18
---------------------------------------------------------CUAUUUGUCUUUGGUAGG-----------------------------   3       18
----------------------------------------------------------UAUUUGUCUUUGGUAGGC----------------------------   1       18
--------------------------------------------------------------UGUCUUUGGUAGGCGGUC------------------------   1       18
--------------------------------------------------------------UGUCUUUGGUAGGCGGUCAUU---------------------   6       21
--------------------------------------------------------------UGUCUUUGGUAGGCGGUCAUUA--------------------   1       22
-----------------------------------------------------------------CUUUGGUAGGCGGUCAUUAAU------------------   7       21
-------------------------------------------------------------------UUGGUAGGCGGUCAUUAAUC-----------------   1       20
--------------------------------------------------------------------UGGUAGGCGGUCAUUAAUCUUC--------------   4       22
------------------------------------------------------------------------AGGCGGUCAUUAAUCUUC--------------   6       18
-------------------------------------------------------------------------GGCGGUCAUUAAUCUUCCAUG----------   3       21
----------------------------------------------------------------------------GGUCAUUAAUCUUCCAUG----------   9       18
---------------------------------------------------------------------------------UUAAUCUUCCAUGCUUUACUU--   1       21
-----UAAAGCAUGGAAGAUUAAUGA------------------------------------------------------------------------------   1       21
-----UAAAGCAUGGAAGAUUAAUG-------------------------------------------------------------------------------   1       20
-------AAGCAUGGAAGAUUAAUGACCGC--------------------------------------------------------------------------   2       23
-------AAGCAUGGAAGAUUAAUGACCGCCU------------------------------------------------------------------------   1       25
--------AGCAUGGAAGAUUAAUGAC-----------------------------------------------------------------------------   1       19
----------CAUGGAAGAUUAAUGACCGCCUACC---------------------------------------------------------------------   1       25
-----------AUGGAAGAUUAAUGACCGCCUACC---------------------------------------------------------------------   2       24
------------UGGAAGAUUAAUGACCGCCU------------------------------------------------------------------------   2       20
-------------GGAAGAUUAAUGACCGCC-------------------------------------------------------------------------   1       18
---------------AAGAUUAAUGACCGCCUAC----------------------------------------------------------------------   1       19
---------------------AAUGACCGCCUACCAAAGACA--------------------------------------------------------------   2       21
---------------------AAUGACCGCCUACCAAAGAC---------------------------------------------------------------   1       20
------------------------------CUACCAAAGACAAAUAGGAAU-----------------------------------------------------   1       21
--------------------------------ACCAAAGACAAAUAGGAAUCAAAU------------------------------------------------   1       24
--------------------------------ACCAAAGACAAAUAGGAAUCAAAUCCUAU-------------------------------------------   1       29
--------------------------------------------UAGGAAUCAAAUCCUAUUUGUCUUUGGUAGG-----------------------------   2       31
---------------------------------------------AGGAAUCAAAUCCUAUUUGUCUUUGGUAGG-----------------------------   2       30
----------------------------------------------GGAAUCAAAUCCUAUUUGUCUUUGGUAGG-----------------------------   1       29
-----------------------------------------------------AAUCCUAUUUGUCUUUGGUA-------------------------------   4       20
-----------------------------------------------------AAUCCUAUUUGUCUUUGG---------------------------------   1       18
------------------------------------------------------AUCCUAUUUGUCUUUGGUAG------------------------------   2       20
-------------------------------------------------------UCCUAUUUGUCUUUGGUAGG-----------------------------   9       20
----------------------------------------------------------UAUUUGUCUUUGGUAGGCGGU-------------------------   5       21
```

D. firmibasis dfi-mir-1189 continued

```
(((((((((((((((((((((((((((((((((((((((((((((((((......)))))))))))))))))))))))))))))))))))))))))))))))))         -108.21 kcal/mol
GGAAGUAAAGcAUGGAAGAUUAAUGACCGCcUACCAAAGACAAAUAGGAAUCAAAUCCUAUUUGUCUUUGGUAGGcGGUCAUUAAUCUUCCAUGCuUUACUUCC         count    length
------------------------------------------------------------UUUGUCUUUGGUAGGCGGUC------------------------         1        20
------------------------------------------------------------UUUGUCUUUGGUAGGCGGUCA-----------------------         1        21
------------------------------------------------------------UUUGUCUUUGGUAGGCGGUCAU----------------------         1        22
-------------------------------------------------------------UUGUCUUUGGUAGGCGGUCAU----------------------         2        21
--------------------------------------------------------------UGUCUUUGGUAGGCGGUCAU----------------------         1        20
---------------------------------------------------------------GUCUUUGGUAGGCGGUCAUU---------------------         2        20
----------------------------------------------------------------UCUUUGGUAGGCGGUCAUU---------------------         2        19
------------------------------------------------------------------UUUGGUAGGCGGUCAUUAAUCU----------------         3        22
------------------------------------------------------------------UUUGGUAGGCGGUCAUUAAUCUUC--------------         2        24
------------------------------------------------------------------UUUGGUAGGCGGUCAUUAAU------------------         2        20
--------------------------------------------------------------------UGGUAGGCGGUCAUUAAUCUU---------------         3        21
----------------------------------------------------------------------GUAGGCGGUCAUUAAUCUUCC-------------         1        21
-----------------------------------------------------------------------UAGGCGGUCAUUAAUCUU---------------         1        18
------------------------------------------------------------------------AGGCGGUCAUUAAUCUUCCAUGC---------         3        23
------------------------------------------------------------------------AGGCGGUCAUUAAUCUUCCAUGCUU-------         1        25
--------------------------------------------------------------------------GCGGUCAUUAAUCUUCCA------------         1        18
---------------------------------------------------------------------------CGGUCAUUAAUCUUCCAUGCUUU------         12       23
----------------------------------------------------------------------------GGUCAUUAAUCUUCCAUGCUUUA-----         1        23
----------------------------------------------------------------------------GGUCAUUAAUCUUCCAUGC---------         4        19
-----------------------------------------------------------------------------GUCAUUAAUCUUCCAUGC---------         3        18
------------------------------------------------------------------------------UCAUUAAUCUUCCAUGCUUU------         1        20
-------------------------------------------------------------------------------CAUUAAUCUUCCAUGCUUUAC----         1        21
-------------------------------------------------------------------------------CAUUAAUCUUCCAUGCUUUACU---         1        22
-------------------------------------------------------------------------------CAUUAAUCUUCCAUGCUU-------         1        18
----------------------------------------------------------------------------------UAAUCUUCCAUGCUUUACUUC-         1        21
```

```
                                                 AU
GGAAGUAAAGcAUGGAAGAUUAAUGACCGCcUACCAAAGACAAAUAGGA   C
CCUUCAUUuCGUACCUUCUAAUUACUGGcGGAUGGUUUCUGUUUAUCCU   A
                                                 AA
```

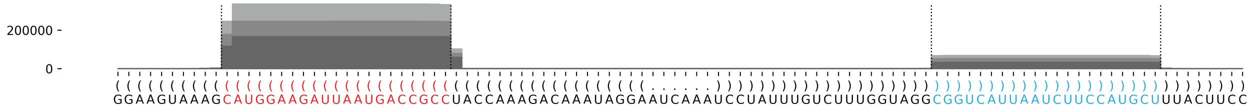

D. firmibasis dfi-mir-1190

(((.(((((((((((((((...(((((..........(((((((((.......((((((((...))))))))))(((((.(((((.....(((((.......))))))))))).))))))))))))))))).......)))))..))))))))))))))).))) −142.84 kcal/mol

CAACCAAAUCaAAAUUAUCAAAUGUCCUCAGcAAGAUAAUUCACAUAAUUCUUUAUUAAAUUCUUUUUAAAUGGGUUAACUCUGAUCUAAAAUAUUAACAAAAUUAUUACUAUAAUAAUUUAUAAAUUGACCAUAAUCAUUUCUUUUUAAAUGGGUUAUUUUAUAUUUAGAUCCAAAUAAUAUUUUUAAAGAAUUUAAUAAACAAUUAUGUGAAUUAUCUUGCuGAGGACAUUUGAUAAUUUUGaUUUGGUUG

Libraries combined Precision: [total = 76.9% | 5p-arm = 51.6% | 3p-arm = 78.1%]

read	count	length
UGAGGACAUUUGAUAAUUUUGA	59509	22
UGAGGACAUUUGAUAAUUUUG	30890	21
CUGAGGACAUUUGAUAAUUUU	9961	21
UGAGGACAUUUGAUAAUUUUGAU	5278	23
UGCUGAGGACAUUUGAUAAUU	4367	21
AAAAUUAUCAAAUGUCCUCAGC	2990	22
GCUGAGGACAUUUGAUAAUUU	2755	21
UUGCUGAGGACAUUUGAUAAU	2676	21
AAAUUAUCAAAUGUCCUCAGC	2111	21
UGAGGACAUUUGAUAAUUUU	1774	20
CUGAGGACAUUUGAUAAUUU	1633	20
CAAUUAUGUGAAUUAUCUUGC	996	21
CUGAGGACAUUUGAUAAUUUUG	768	22
UGAGGACAUUUGAUAAUUU	657	19
UGCUGAGGACAUUUGAUAAUUU	568	22
AUCUUGCUGAGGACAUUUGAU	453	21
UCUUGCUGAGGACAUUUGAUA	370	21
AAUUAUCAAAUGUCCUCAGC	388	20
UGAGGACAUUUGAUAAUUUUGAUU	392	24
ACAAUUAUGUGAAUUAUCUUGC	231	22
UUGCUGAGGACAUUUGAUAAUU	337	22
UGAGGACAUUUGAUAAUU	208	18
UGCUGAGGACAUUUGAUAAU	254	20
AAUUAUCAAAUGUCCUCAGCA	230	21
GAGGACAUUUGAUAAUUUUGA	206	21
GCUGAGGACAUUUGAUAAUU	221	20
CUGAGGACAUUUGAUAAUU	142	19
ACAAUUAUGUGAAUUAUCUUG	152	21
CUGAGGACAUUUGAUAAUUUUGA	133	23
AAAAUUAUCAAAUGUCCUCAG	127	21
AUCUUGCUGAGGACAUUUGAUA	97	22
UGAAUUAUCUUGCUGAGGAC	112	20
GAGGACAUUUGAUAAUUUUG	115	20
AGGACAUUUGAUAAUUUUGAU	78	21
AAAUUAUCAAAUGUCCUCAG	61	20
AAUUAUCUUGCUGAGGACAUU	75	21
GUGAAUUAUCUUGCUGAGGAC	64	21
CAAUUAUGUGAAUUAUCUUGCU	72	22
AGGACAUUUGAUAAUUUUGAUU	53	22
UGAAUUAUCUUGCUGAGGACA	61	21
UCUUGCUGAGGACAUUUGAUAA	45	22
AUCUUGCUGAGGACAUUUGA	21	20
CUGAGGACAUUUGAUAAUUUUGAU	23	24
AUUAUCAAAUGUCCUCAGC	30	19
AUCUUGCUGAGGACAUUUGAUAA	29	23
UCUUGCUGAGGACAUUUGAU	29	20
UUGCUGAGGACAUUUGAUAA	43	20
AAUUAUGUGAAUUAUCUUGCU	26	21
AUUAUCUUGCUGAGGACAUU	24	20
UUGCUGAGGACAUUUGAUAAUUU	24	23
CAAUUAUGUGAAUUAUCUUG	34	20
AAAUUAUCAAAUGUCCUCAGCA	17	22
ACAAUUAUGUGAAUUAUCUUGCU	24	23
CUGAGGACAUUUGAUAAU	26	18
UAUCAAAUGUCCUCAGCAAGA	18	21
AUUAUCUUGCUGAGGACAUUU	13	21
UUAUCUUGCUGAGGACAUUUG	10	21
AAUUAUCAAAUGUCCUCAG	11	19
AAUUAUCAAAUGUCCUCAGCAA	8	22
AAUUAUGUGAAUUAUCUUGC	13	20
UAUCUUGCUGAGGACAUUUGA	10	21
UCUUGCUGAGGACAUUUGAUAAU	18	23
GCUGAGGACAUUUGAUAAUUUU	12	22

D. firmibasis dfi-mir-1190 continued

((.(((((((((((((((...(((((..........(((((((((.......((((((((...))))))))(((((.(((((.....(((((.......))))))))))).))))))))))))))).......)))))..)))))))))))))))).)) −142.84 kcal/mol

CAACCAAAUCaAAAUUAUCAAAUGUCCUCAGcAAGAUAAUUCACAUAAUUCUUUAUUAAAUUCUUUUUUAAAUGGGUUAACUCUGAUCUAAAAUAUUAACAAAAUUAUUACUAUAAUAAUUUAUAAAUUGACCAUAAUCAUUUCUUUUUUAAAUGGGUUAUUUUAUAUUUAGAUCCAAAUAAUAUUUUUAAAGAAUUUAAUAAACAAUUAUGUGAAUUAUCUUGCuGAGGACAUUUGAUAAUUUGaUUUGGUUG

Libraries combined   Precision: [total = 76.9% | 5p-arm = 51.6% | 3p-arm = 78.1%]

read	count	length
GCUGAGGACAUUUGAUAAU	11	19
AGGACAUUUGAUAAUUUGA	9	20
UCAAAUGUCCUCAGCAAGAUA	12	21
AUCUUGCUGAGGACAUUUGAUAAU	12	24
CUUGCUGAGGACAUUUGAUA	4	20
ACAUUUGAUAAUUUGAUUUG	3	21
AACAAUUAUGUGAAUUAUCUUGCU	6	24
CAAUUAUGUGAAUUAUCU	2	18
AUUAUGUGAAUUAUCUUGC	4	19
AUGUGAAUUAUCUUGCUGAGG	17	21
UGAAUUAUCUUGCUGAGGACAUU	5	23
GAAUUAUCUUGCUGAGGACAU	6	21
GAAUUAUCUUGCUGAGGACA	6	20
AAUUAUCUUGCUGAGGACAU	4	20
UGCUGAGGACAUUUGAUAA	6	19
GAGGACAUUUGAUAAUUUU	5	19
UUUGAUAAUUUGAUUUGGUU	5	21
UUGAUAAUUUGAUUUGGUUG	4	21
UCAAAAUUAUCAAAUGUCCUC	1	21
AAAAUUAUCAAAUGUCCUCAGCA	3	23
AAUUAUCAAAUGUCCUCAGCAAG	2	23
UUAUCAAAUGUCCUCAGC	1	18
UUAUCAAAUGUCCUCAGCA	3	19
UAUCAAAUGUCCUCAGCAAG	2	20
UCCUCAGCAAGAUAAUUCACA	2	21
AAGAUAAUUCACAUAAUUCUU	1	21
AAGAUAAUUCACAUAAUUCU	4	20
AAAUGGGUUAACUCUGAUCUA	1	21
AACAAUUAUGUGAAUUAUCUUGC	4	23
CAAUUAUGUGAAUUAUCUUGCUGA	1	24
AUUAUGUGAAUUAUCUUGCUG	7	21
AUGUGAAUUAUCUUGCUGAGGA	2	22
AUGUGAAUUAUCUUGCUGA	1	19
UGUGAAUUAUCUUGCUGAG	1	19
UGAAUUAUCUUGCUGAGGACAU	3	22
GAAUUAUCUUGCUGAGGACAUU	2	22
AAUUAUCUUGCUGAGGACA	2	19
AUUAUCUUGCUGAGGACAUUUGAU	1	24
UUAUCUUGCUGAGGACAUUUGAU	2	23
UAUCUUGCUGAGGACAUUUGAU	2	22
AUCUUGCUGAGGACAUUUG	1	19
CUUGCUGAGGACAUUUGAUAAU	3	22
UUGCUGAGGACAUUUGAUA	4	19
UGAGGACAUUUGAUAAUUUGAUUU	10	25
GAGGACAUUUGAUAAUUUGAUU	3	23
AGGACAUUUGAUAAUUUG	5	19
GGACAUUUGAUAAUUUGAUU	6	21
CAUUUGAUAAUUUGAUUUGG	1	21
UCAAAAUUAUCAAAUGUCCUCAGC	1	24
UCAAAAUUAUCAAAUGUCCU	1	20
AAAUUAUCAAAUGUCCUCAGCAA	1	23
AUUAUCAAAUGUCCUCAG	3	18
AUUAUCAAAUGUCCUCAGCAA	1	21
AUUAUCAAAUGUCCUCAGCA	2	20
UUAUCAAAUGUCCUCAGCAAG	4	21
AUCAAAUGUCCUCAGCAAGAUA	1	22
AUCAAAUGUCCUCAGCAAGAUAAUUCACA	1	29
UCAAAUGUCCUCAGCAAGAU	1	20
CAAAUGUCCUCAGCAAGAUA	3	20
UGUCCUCAGCAAGAUAAUUC	1	20
CUCAGCAAGAUAAUUCACAUA	1	21
CAAGAUAAUUCACAUAAUUCU	1	21
CACAUAAUUCUUUAUUAAAUU	1	21

D. firmibasis dfi-mir-1190 continued

```
((((((((((((((((((((((((((((((((((((((((((((((((((.(((((((((((((((...(((((..........((((((((((.......(((((((((...)))))))))(((((.(((((.....(((((.......))))))))))).))))))))))))))).......)))))..))))))))))))))).))))))))))))))))))))))))))))))))))))))))))))))))))   -142.84 kcal/mol
CAACCAAAUCaAAAUUAUCAAAUGUCCUCAGcAAGAUAAUUCACAUAAUUCUUUAUUAAAUUCUUUUUUAAAUGGGUUAACUCUGAUCUAAAAUAUUAACAAAAUUAUUACUAUAAUAAUUUAUAAAUUGACCAUAAUCAUUUCUUUUUUAAAUGGGUUAUUUUAUAUUUUAGAUCCAAAUAAUAUUUUUAAAGAAUUUAAUAAACAAUUAUGUGAAUUAUCUUGCuGAGGACAUUUGAUAAUUUUGaUUUGGUUG   count   length
Libraries combined          Precision: [ total = 76.9% | 5p-arm = 51.6% | 3p-arm = 78.1% ]
```

Read	count	length
CUUUUUUAAAUGGGUUAACUC	1	21
AACAAUUAUGUGAAUUAUCUUG	13	22
ACAAUUAUGUGAAUUAUC	1	18
CAAUUAUGUGAAUUAUCUUGCUG	1	23
UUAUGUGAAUUAUCUUGCUGA	1	21
UAUGUGAAUUAUCUUGCUGAG	2	21
UAUGUGAAUUAUCUUGCUGAGG	2	22
AUGUGAAUUAUCUUGCUGAG	1	20
GUGAAUUAUCUUGCUGAGGACA	1	22
UGAAUUAUCUUGCUGAGGA	1	19
GAAUUAUCUUGCUGAGGAC	1	19
AAUUAUCUUGCUGAGGACAUUU	1	22
AUUAUCUUGCUGAGGACAU	2	19
AUUAUCUUGCUGAGGACAUUUGA	1	23
UAUCUUGCUGAGGACAUUUGAUA	1	23
UAUCUUGCUGAGGACAUUUG	1	20
UCUUGCUGAGGACAUUUGAUAAUU	1	24
CUUGCUGAGGACAUUUGAUAA	6	21
UGCUGAGGACAUUUGAUAAUUUU	1	23
GCUGAGGACAUUUGAUAA	1	18
GAGGACAUUUGAUAAUUU	5	18
GAGGACAUUUGAUAAUUUUGAU	8	22
AUUUGAUAAUUUUGAUUUGGU	1	21
AUUUGAUAAUUUUGAUUUGGUU	1	22
UUGAUAAUUUUGAUUUGGU	1	19

```
                                                 C                 UUU      GGUUAACUCU          AUUAACA          C
CAACCAAAUCaAAAUUAUCAAAUGUCCUCAGcAAGAUAAUUCACAUAAUU UUUAUUAAAUUCUUU    AAAUG          GAUCUAAAAU        AAAUUAUUA U
GUUGGUUUaGUUUUAAUAGUUUACAGGAGuCGUUCUAUUAAGUGUAUUAA AAAUAAUUUAAGAAA  -UUUAU        ---CUAGAUUUUA---\  AUUGGGUAAA /
                                                 C                 UU       AAUAAAC                 AU           A
                                                                                                    UA
                                                                                                    UA
                                                                                                    UA       AUAAU      CUU
                                                                                                  U   UUGACC      CAUUU    U
                                                                                                   \--AUUGG-----GUAAA    /
                                                                                                                    UUU
```

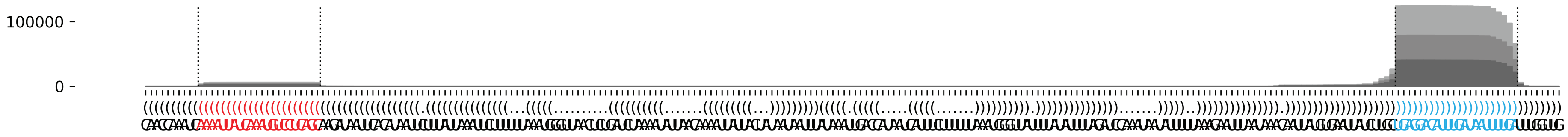

D. firmibasis dfi-mir-1191

```
((((((((((((((((.(((.(((((((((.((((((((((((((((((.(..(((.(((((.(.(((((((.((((((......)))))))......))))))).))))))...)))..)...)))))).....))))))))))))).))))))))).))).)))))))))))))))))  -95.3 kcal/mol
UCAUUAUGCAaACAAGUGCAAUCAGGAAAUaAAUACUAUAACUGAGCAAGUAUCAUUUCACAUCAAACAAACAAGAAGAAUUAAAAAGAGUUAGUUUAUUGGCUCUUGUUGGUUGAUCAUUGAUUUUUAAUACUGCGGUUGUUCAGUUAUAGUUUUUAuUUCCUCGUUACACUUGUUUgCAUAAUGA  count  length
Libraries combined    Precision: [ total = 97.1% | 5p-arm = 44.6% | 3p-arm = 97.8% ]
```

Read	count	length
UUUCCUCGUUACACUUGUUUG	74616	21
UUUCCUCGUUACACUUGUUUGC	11194	22
UUUCCUCGUUACACUUGUUUGCA	5823	23
AACAAGUGCAAUCAGGAAAUA	464	21
UUUCCUCGUUACACUUGUUU	634	20
CAAACAAGUGCAAUCAGGAAAUA	328	23
UUUCCUCGUUACACUUGUUUGCAU	526	24
UAUUUCCUCGUUACACUUGUU	334	21
AAACAAGUGCAAUCAGGAAAUA	228	22
UAUUUCCUCGUUACACUUGUUU	337	22
UUUCCUCGUUACACUUGUU	281	19
AACAAGUGCAAUCAGGAAAU	89	20
UUCCUCGUUACACUUGUUUGCA	156	22
UCCUCGUUACACUUGUUUGCA	119	21
CAAACAAGUGCAAUCAGGAAAU	83	22
UUCCUCGUUACACUUGUUUG	89	20
UUAUUUCCUCGUUACACUUGU	76	21
UUCCUCGUUACACUUGUUUGC	81	21
UUUUAUUUCCUCGUUACACUUG	45	22
AUUUCCUCGUUACACUUGUUU	51	21
AAACAAGUGCAAUCAGGAAAU	30	21
UCCUCGUUACACUUGUUUGC	37	20
UUUUAUUUCCUCGUUACACUU	20	21
UUUUAUUUCCUCGUUACACU	31	20
UUUCCUCGUUACACUUGU	30	18
UAUUUCCUCGUUACACUUGUUUG	7	23
UCCUCGUUACACUUGUUUG	29	19
UUUCCUCGUUACACUUGUUUGCAUA	19	25
CAAACAAGUGCAAUCAGGAAA	7	21
AACAAGUGCAAUCAGGAAA	4	19
UUAUUUCCUCGUUACACUUGUU	10	22
UAUUUCCUCGUUACACUUGU	18	20
UUCCUCGUUACACUUGUU	3	18
ACAAGUGCAAUCAGGAAAUA	8	20
UUCCUCGUUACACUUGUUUGCAU	11	23
GCAAACAAGUGCAAUCAGGAAAU	2	23
AACAAGUGCAAUCAGGAAAUAA	3	22
ACAAGUGCAAUCAGGAAAU	1	19
UCAGGAAAUAAAUACUAUAAC	1	21
UAUUGGCUCUUGUUGGUUGAUCAUU	1	25
UGCGGUUGUUCAGUUAUAGUU	4	21
CGGUUGUUCAGUUAUAGUUU	1	20
AGUUAUAGUUUUUAUUUCCUC	1	21
UUAUAGUUUUUAUUUCCUCGUU	1	22
GUUUUUAUUUCCUCGUUACACU	6	22
UUUAUUUCCUCGUUACACUUG	1	21
UUAUUUCCUCGUUACACUUG	13	20
UAUUUCCUCGUUACACUUGUUUGC	1	24
UAUUUCCUCGUUACACUUG	3	19
UCCUCGUUACACUUGUUU	3	18
CCUCGUUACACUUGUUUGCA	5	20
CAAACAAGUGCAAUCAGGAAAUAA	1	24
AAACAAGUGCAAUCAGGAAAUAA	3	23

D. firmibasis dfi-mir-1191 continued

```
((((((((((((((((((.(((.((((((((((.(((((((((((((((((((.(..(((.(((((.(.(((((((.(((((((......))))))).......))))))).)))))...)))..)...))))).....)))))))))))))).)))))))))).))).))))))))))))))))))  -95.3 kcal/mol
UCAUUAUGCAaACAAGUGCAAUCAGGAAAUaAAUACUAUAACUGAGCAAGUAUCAUUUCACAUCAAACAAACAAGAAGAAUUAAAAAGAGUUAGUUUAUUGGCUCUUGUUGGUUGAUCAUUGAUUUUUAAUACUGCGGUUGUUCAGUUAUAGUUUUUAuUUCCUCGUUACACUUGUUUgCAUAAUGA  count      length
Libraries combined    Precision: [ total = 97.1% | 5p-arm = 44.6% | 3p-arm = 97.8% ]
---------AAACAAGUGCAAUCAGGAAA--------------------------------------------------------------------------------------------------------------------------------------------------------------  3          20
--------------AGUGCAAUCAGGAAAUAAAUA--------------------------------------------------------------------------------------------------------------------------------------------------------  1          21
----------------------------------------------------------------------------------AAAAAGAGUUAGUUUAUUGGC------------------------------------------------------------------------------------  1          21
-------------------------------------------------------------------------------------------------------------------------------------UGCGGUUGUUCAGUUAUAGUUU--------------------------------  1          22
--------------------------------------------------------------------------------------------------------------------------------------GCGGUUGUUCAGUUAUAGUUU--------------------------------  1          21
-----------------------------------------------------------------------------------------------------------------------------------------------------UAGUUUUUAUUUCCUCGUUAC-----------------  4          21
--------------------------------------------------------------------------------------------------------------------------------------------------------UUUUUAUUUCCUCGUUACACU--------------  1          21
---------------------------------------------------------------------------------------------------------------------------------------------------------UUUUAUUUCCUCGUUACAC---------------  1          19
----------------------------------------------------------------------------------------------------------------------------------------------------------UUUAUUUCCUCGUUACACUUGU-----------  1          22
-------------------------------------------------------------------------------------------------------------------------------------------------------------AUUUCCUCGUUACACUUGUUUG--------  10         22
-------------------------------------------------------------------------------------------------------------------------------------------------------------AUUUCCUCGUUACACUUGUU----------  3          20
--------------------------------------------------------------------------------------------------------------------------------------------------------------UUUCCUCGUUACACUUGUUUGCAUAA---  2          26
---------------------------------------------------------------------------------------------------------------------------------------------------------------UUCCUCGUUACACUUGUUU---------  2          19
---------------------------------------------------------------------------------------------------------------------------------------------------------------UUCCUCGUUACACUUGUUUGCAUA----  1          24
----------------------------------------------------------------------------------------------------------------------------------------------------------------UCCUCGUUACACUUGUUUGCAU-----  8          22
-----------------------------------------------------------------------------------------------------------------------------------------------------------------CCUCGUUACACUUGUUUG--------  3          18
-----------------------------------------------------------------------------------------------------------------------------------------------------------------CCUCGUUACACUUGUUUGC-------  1          19
-----------------------------------------------------------------------------------------------------------------------------------------------------------------CCUCGUUACACUUGUUUGCAU-----  4          21
--------------------------------------------------------------------------------------------------------------------------------------------------------------------CGUUACACUUGUUUGCAUAAU--  1          21
---------------------------------------------------------------------------------------------------------------------------------------------------------------------GUUACACUUGUUUGCAUAAUG-  2          21
```

```
                  C   C          U                        C-- UU   C       A A       A            AA
UCAUUAUGCAaACAAGUG AAU AGGAAAUaAA ACUAUAACUGAGCA-----AGUAU   A  UCA --AUCAA C AACAAGA -----GAAUUAA  A
AGUAAUACgUUUGUUCAC UUG UCCUUuAUUU UGAUAUUGACUUGU     UCAUA   U  AGU   UAGUU-G UUGUUCU      UUUGAUU  G
                  A   C          U              UGGCG     AUU UU   UAC       G       CGGUUA       GA
```

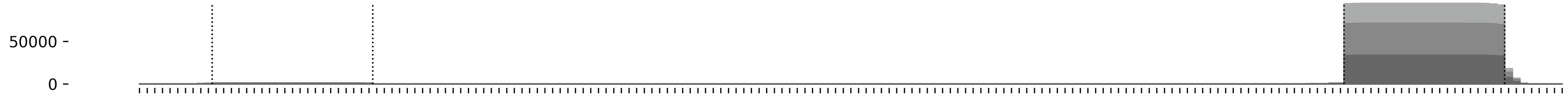

((((((((((((((((((.(((.((((((((((.(((((((((((((((((((.(..(((.(((((.(.(((((((.(((((((......))))))).......))))))).)))))...)))..)...))))).....)))))))))))))).)))))))))).))).))))))))))))))))))
UCAUUAUGCAAACAAGUGCAAUCAGGAAAUAAAUACUAUAACUGAGCAAGUAUCAUUUCACAUCAAACAAACAAGAAGAAUUAAAAAGAGUUAGUUUAUUGGCUCUUGUUGGUUGAUCAUUGAUUUUUAAUACUGCGGUUGUUCAGUUAUAGUUUUUAUUUCCUCGUUACACUUGUUUGCAUAAUGA

D. firmibasis dfi-mir-1192

```
((((((((.((.(((((((.((((((((((((((.((((...(((((((((........))))))))).)))).)))))))))))))).))))))).)).))))))))   -69.88 kcal/mol
UUUAUGCAGGuGUACCAAGACAAUCUCCAAgUACGUAUGGUAACUCAAUUGAAAAAUAAUGAUUGAGUACAUAAGUACUuGGAGAUUGGCUUGGUAAACaUGCAUAAA   count    length
Libraries combined         Precision: [ total = 84.5% | 5p-arm = 87.9% | 3p-arm = 76.9% ]
----------UGUACCAAGACAAUCUCCAAG-----------------------------------------------------------------------------   51       21
----UGCAGGUGUACCAAGACAAUC-----------------------------------------------------------------------------------   5        21
-------------------------------------------------------------------------------UGGAGAUUGGCUUGGUAAACA--------   9        21
--------------------------------------------------------------------------------GGAGAUUGGCUUGGUAAACAUG------   2        22
---------------------------------------------------------------------------UACUUGGAGAUUGGCUUGGUA------------   1        21
-------------------------------------------------------------------------------UGGAGAUUGGCUUGGUAAAC---------   10       20
--------------------------------------------------------------------------------GGAGAUUGGCUUGGUAAAC---------   1        19
------------UACCAAGACAAUCUCCAAGUA---------------------------------------------------------------------------   1        21
------------UACCAAGACAAUCUCCAAG-----------------------------------------------------------------------------   1        19
-----------------------------------------------------------------------------CUUGGAGAUUGGCUUGGUAAACA--------   2        23
-------------------------------------------------------------------------------UGGAGAUUGGCUUGGUAAA----------   1        19
Library = 1        Precision: [ total = 68.2% | 5p-arm = 80.0% | 3p-arm = 42.9% ]
----------UGUACCAAGACAAUCUCCAAG-----------------------------------------------------------------------------   12       21
----UGCAGGUGUACCAAGACAAUC-----------------------------------------------------------------------------------   3        21
-------------------------------------------------------------------------------UGGAGAUUGGCUUGGUAAACA--------   2        21
--------------------------------------------------------------------------------GGAGAUUGGCUUGGUAAACAUG------   2        22
---------------------------------------------------------------------------UACUUGGAGAUUGGCUUGGUA------------   1        21
-------------------------------------------------------------------------------UGGAGAUUGGCUUGGUAAAC---------   1        20
--------------------------------------------------------------------------------GGAGAUUGGCUUGGUAAAC---------   1        19
Library = 2        Precision: [ total = 92.0% | 5p-arm = 94.3% | 3p-arm = 86.7% ]
----------UGUACCAAGACAAUCUCCAAG-----------------------------------------------------------------------------   33       21
-------------------------------------------------------------------------------UGGAGAUUGGCUUGGUAAACA--------   7        21
-------------------------------------------------------------------------------UGGAGAUUGGCUUGGUAAAC---------   5        20
-----------------------------------------------------------------------------CUUGGAGAUUGGCUUGGUAAACA--------   2        23
----UGCAGGUGUACCAAGACAAUC-----------------------------------------------------------------------------------   1        21
------------UACCAAGACAAUCUCCAAGUA---------------------------------------------------------------------------   1        21
-------------------------------------------------------------------------------UGGAGAUUGGCUUGGUAAA----------   1        19
Library = 3        Precision: [ total = 83.3% | 5p-arm = 75.0% | 3p-arm = 100.0% ]
----------UGUACCAAGACAAUCUCCAAG-----------------------------------------------------------------------------   6        21
-------------------------------------------------------------------------------UGGAGAUUGGCUUGGUAAAC---------   4        20
----UGCAGGUGUACCAAGACAAUC-----------------------------------------------------------------------------------   1        21
------------UACCAAGACAAUCUCCAAG-----------------------------------------------------------------------------   1        19
```

```
        G  G       A              G    GUA         AAA
UUUAUGCA Gu UACCAAG CAAUCUCCAAgUAC UAUG   ACUCAAUUG   A
AAAUACGU CA AUGGUUC GUUAGAGGuUCAUG AUAC --UGAGUUAGU   A
        a  A       G              A    A           AAU
```

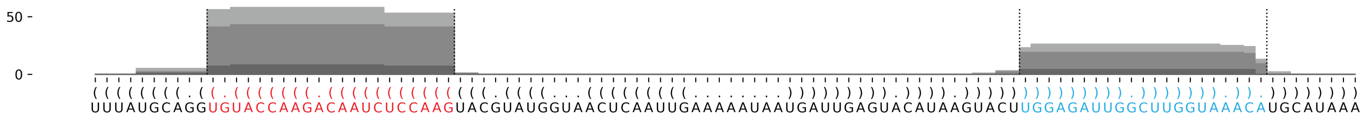

D. firmibasis dfi-mir-1193

```
...(((.((((((((.(((((((.((.(((((.((((.((((((((........)))))....))))))).))))).)).)))))))).))))))))).)))...    -42.09 kcal/mol
UUUUCUAUUUuUACUCCCAUUAUUAAGGAUgCAAAUUAAAUUUGUAUGCAAAAACACAUAUUUUUAAAUUAAAUUGCAcCCAUAAUAAUGAGAGUAAAaAAAGAUAA    count   length
Libraries combined          Precision: [ total = 99.6% | 5p-arm = 99.6% | 3p-arm = 100.0% ]
----------UUACUCCCAUUAUUAAGGAUG----------------------------------------------------------------------------    63315   21
---------UUUACUCCCAUUAUUAAGGAUG----------------------------------------------------------------------------    137     22
----------UUACUCCCAUUAUUAAGGAU-----------------------------------------------------------------------------    86      20
-----------UACUCCCAUUAUUAAGGAUG----------------------------------------------------------------------------    45      20
------------------------------------------------------------------------------CCCAUAAUAAUGAGAGUAAAA--------    25      21
----------UUACUCCCAUUAUUAAGGA------------------------------------------------------------------------------    14      19
-------------CUCCCAUUAUUAAGGAUG----------------------------------------------------------------------------    28      18
------------ACUCCCAUUAUUAAGGAUG----------------------------------------------------------------------------    17      19
------AUUUUUACUCCCAUUAUUAAGGAUG----------------------------------------------------------------------------    2       25
------------------------------------------------------------------------------CCCAUAAUAAUGAGAGUAAA---------    6       20
--------UUUUACUCCCAUUAUUAAGGAUG----------------------------------------------------------------------------    1       23
------------------------------------------------------------------------------CCCAUAAUAAUGAGAGUAAAAA-------    2       22
-------UUUUUACUCCCAUUAUUAAGGAUG----------------------------------------------------------------------------    1       24
----------UUACUCCCAUUAUUAAGG-------------------------------------------------------------------------------    1       18
Library = 1        Precision: [ total = 99.7% | 5p-arm = 99.7% | 3p-arm = 100.0% ]
----------UUACUCCCAUUAUUAAGGAUG----------------------------------------------------------------------------    25559   21
---------UUUACUCCCAUUAUUAAGGAUG----------------------------------------------------------------------------    54      22
----------UUACUCCCAUUAUUAAGGAU-----------------------------------------------------------------------------    32      20
-----------UACUCCCAUUAUUAAGGAUG----------------------------------------------------------------------------    15      20
------------------------------------------------------------------------------CCCAUAAUAAUGAGAGUAAAA--------    8       21
----------UUACUCCCAUUAUUAAGGA------------------------------------------------------------------------------    7       19
-------------CUCCCAUUAUUAAGGAUG----------------------------------------------------------------------------    7       18
------------ACUCCCAUUAUUAAGGAUG----------------------------------------------------------------------------    5       19
------AUUUUUACUCCCAUUAUUAAGGAUG----------------------------------------------------------------------------    2       25
------------------------------------------------------------------------------CCCAUAAUAAUGAGAGUAAA---------    2       20
--------UUUUACUCCCAUUAUUAAGGAUG----------------------------------------------------------------------------    1       23
------------------------------------------------------------------------------CCCAUAAUAAUGAGAGUAAAAA-------    1       22
Library = 2        Precision: [ total = 99.6% | 5p-arm = 99.6% | 3p-arm = 100.0% ]
----------UUACUCCCAUUAUUAAGGAUG----------------------------------------------------------------------------    17531   21
---------UUUACUCCCAUUAUUAAGGAUG----------------------------------------------------------------------------    36      22
----------UUACUCCCAUUAUUAAGGAU-----------------------------------------------------------------------------    22      20
-------------CUCCCAUUAUUAAGGAUG----------------------------------------------------------------------------    12      18
-----------UACUCCCAUUAUUAAGGAUG----------------------------------------------------------------------------    11      20
------------ACUCCCAUUAUUAAGGAUG----------------------------------------------------------------------------    6       19
------------------------------------------------------------------------------CCCAUAAUAAUGAGAGUAAAA--------    6       21
-------UUUUUACUCCCAUUAUUAAGGAUG----------------------------------------------------------------------------    1       24
----------UUACUCCCAUUAUUAAGGA------------------------------------------------------------------------------    1       19
------------------------------------------------------------------------------CCCAUAAUAAUGAGAGUAAA---------    1       20
Library = 3        Precision: [ total = 99.6% | 5p-arm = 99.6% | 3p-arm = 100.0% ]
----------UUACUCCCAUUAUUAAGGAUG----------------------------------------------------------------------------    20225   21
---------UUUACUCCCAUUAUUAAGGAUG----------------------------------------------------------------------------    47      22
----------UUACUCCCAUUAUUAAGGAU-----------------------------------------------------------------------------    32      20
-----------UACUCCCAUUAUUAAGGAUG----------------------------------------------------------------------------    19      20
------------------------------------------------------------------------------CCCAUAAUAAUGAGAGUAAAA--------    11      21
-------------CUCCCAUUAUUAAGGAUG----------------------------------------------------------------------------    9       18
----------UUACUCCCAUUAUUAAGGA------------------------------------------------------------------------------    6       19
------------ACUCCCAUUAUUAAGGAUG----------------------------------------------------------------------------    6       19
------------------------------------------------------------------------------CCCAUAAUAAUGAGAGUAAA---------    3       20
----------UUACUCCCAUUAUUAAGG-------------------------------------------------------------------------------    1       18
------------------------------------------------------------------------------CCCAUAAUAAUGAGAGUAAAAA-------    1       22
```

```
UUU    A          C         A  A      A     A              CAA
   UCU  UUUuUACUC  CAUUAUUA  GG  UgCAA  UUAA  UUU----GUAUG    A
   AGA  AaAAAUGAG  GUAAUAAU  CC  ACGUU  AAUU-AAA      UAUAC    A
AAU    A          A         A  c      A          UUUU      ACA
```

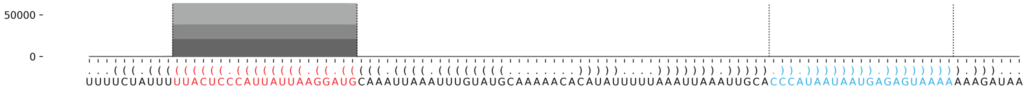

D. firmibasis dfi-mir-1194

```
((((((((((((((((((((((((((((.(((((((((((((.((.(((....))).)).))))))))))))).)))))))))))))))))))))))))))) -101.3 kcal/mol
UUAUUAAUGCuCGGAGCUCUGAUGCGAACUgUGGGUGGACGCUGGAAAUGUUGAUUGCCUGCGUCCACCCACAuUUCGCAUCAGAGCUCCGAGcAUUAAUAA count    length
Libraries combined         Precision: [ total = 98.8% | 5p-arm = 77.1% | 3p-arm = 98.8% ]
-------------------------------------------------------------------------UUUCGCAUCAGAGCUCCGAGC-------- 407849   21
--------------------------------------------------------------------------UUCGCAUCAGAGCUCCGAGC-------- 3289     20
-------------------------------------------------------------------------UUUCGCAUCAGAGCUCCGAG--------- 1908     20
-------------------------------------------------------------------------UUUCGCAUCAGAGCUCCGAGCA------- 1359     22
-------------------------------------------------------------------------UUUCGCAUCAGAGCUCCGAGCAU------ 557      23
----------------------------------------------------------------------------CGCAUCAGAGCUCCGAGCAUU----- 510      21
---------------------------------------------------------------------------UCGCAUCAGAGCUCCGAGC-------- 284      19
-------------------------------------------------------------------------UUUCGCAUCAGAGCUCCGAGCAUU----- 216      24
----------UCGGAGCUCUGAUGCGAACU------------------------------------------------------------------------ 203      20
---------------------------------------------------------------------------UCGCAUCAGAGCUCCGAGCAU------ 242      21
----------UCGGAGCUCUGAUGCGAACUG----------------------------------------------------------------------- 173      21
-------------------------------------------------------------------------UUUCGCAUCAGAGCUCCGA---------- 60       19
----------UCGGAGCUCUGAUGCGAAC------------------------------------------------------------------------- 51       19
-----------------------------------------------------------------------CAUUUCGCAUCAGAGCUCCGA---------- 41       21
---UUAAUGCUCGGAGCUCUGAUG------------------------------------------------------------------------------ 50       21
--------------------------------------------------------------------------UUCGCAUCAGAGCUCCGAGCA------- 43       21
---------------------------------------------------------------------------UCGCAUCAGAGCUCCGAGCAUU----- 39       22
----------------------------------------------------------------------------CGCAUCAGAGCUCCGAGCAU------ 45       20
--------------------------------------------------------------------------------UCAGAGCUCCGAGCAUUAAUA- 34       21
-------------------------------------------------------------------------UUUCGCAUCAGAGCUCCGAGCAUUAA--- 40       26
-------------------------------------------------------------------------UUUCGCAUCAGAGCUCCGAGCAUUA---- 15       25
-------------------------------------------------------------------------UUUCGCAUCAGAGCUCCGAGCAUUAAU-- 25       27
-----------------------------------------------------------------------------GCAUCAGAGCUCCGAGCAUUA---- 15       21
-------------------------------------------------------------------------------AUCAGAGCUCCGAGCAUUAAU-- 20       21
------------------------------------------------------------------------AUUUCGCAUCAGAGCUCCGAGC-------- 22       22
------------------------------------------------------------------------------CAUCAGAGCUCCGAGCAUUAA--- 6        21
--------------------------------------------------------------------------------UCAGAGCUCCGAGCAUUAAU-- 9        20
-----AAUGCUCGGAGCUCUGAUGCG---------------------------------------------------------------------------- 18       21
----------------------------------------------------------------------------CGCAUCAGAGCUCCGAGCAUUA---- 6        22
------AUGCUCGGAGCUCUGAUGCGA--------------------------------------------------------------------------- 3        21
----------UCGGAGCUCUGAUGCGAACUGU---------------------------------------------------------------------- 2        22
---------------------------------------------------------------------CACAUUUCGCAUCAGAGCUCC------------ 4        21
-----------------------------------------------------------------------CAUUUCGCAUCAGAGCUCCG----------- 8        20
--------------------------------------------------------------------------UUCGCAUCAGAGCUCCGAG--------- 4        19
---------------------------------------------------------------------------UCGCAUCAGAGCUCCGAGCAUUA---- 7        23
-----------------------------------------------------------------------------GCAUCAGAGCUCCGAGCAUUAA--- 2        22
------------------------------------------------------------------------------CAUCAGAGCUCCGAGCAUUAAUAA 2        24
------------------------------------------------------------------------------CAUCAGAGCUCCGAGCAUU----- 4        19
---UUAAUGCUCGGAGCUCUGAU------------------------------------------------------------------------------- 3        20
----UAAUGCUCGGAGCUCUGAUGC----------------------------------------------------------------------------- 5        21
-------UGCUCGGAGCUCUGAUGCGAA-------------------------------------------------------------------------- 4        21
-------UGCUCGGAGCUCUGAUGCGAACU------------------------------------------------------------------------ 12       23
--------GCUCGGAGCUCUGAUGCGAAC------------------------------------------------------------------------- 1        21
--------GCUCGGAGCUCUGAUGCGA--------------------------------------------------------------------------- 1        19
---------CUCGGAGCUCUGAUGCGAAC------------------------------------------------------------------------- 1        20
------------GGAGCUCUGAUGCGAACUGUG--------------------------------------------------------------------- 1        21
-------------------UGAUGCGAACUGUGGGUGGACG------------------------------------------------------------- 1        22
---------------------------------------------------UGAUUGCCUGCGUCCACCCA------------------------------- 1        20
-------------------------------------------------------------CGUCCACCCACAUUUCGCAUC-------------------- 1        21
---------------------------------------------------------------------CACAUUUCGCAUCAGAGCUCCG----------- 1        22
----------------------------------------------------------------------ACAUUUCGCAUCAGAGCUCCG----------- 4        21
------------------------------------------------------------------------AUUUCGCAUCAGAGCUCCGAG--------- 2        21
------------------------------------------------------------------------AUUUCGCAUCAGAGCUCCGA---------- 1        20
```

D. firmibasis dfi-mir-1194

```
((((((((((((((((((((((((((((.(((((((((((((.((.(((....))).)).))))))))))))).)))))))))))))))))))))))))))) -101.3 kcal/mol
UUAUUAAUGCuCGGAGCUCUGAUGCGAACUgUGGGUGGACGCUGGAAAUGUUGAUUGCCUGCGUCCACCCACAuUUCGCAUCAGAGCUCCGAGcAUUAAUAA count    length
Libraries combined          Precision: [ total = 98.8% | 5p-arm = 77.1% | 3p-arm = 98.8% ]
--------------------------------------------------------------------------UUCGCAUCAGAGCUCCGAGCAU------ 4       22
---------------------------------------------------------------------------UCGCAUCAGAGCUCCGAGCAUUAA--- 3       24
---------------------------------------------------------------------------UCGCAUCAGAGCUCCGAGCAUUAAU-- 1       25
---------------------------------------------------------------------------UCGCAUCAGAGCUCCGAG--------- 1       18
---------------------------------------------------------------------------UCGCAUCAGAGCUCCGAGCA------- 4       20
----------------------------------------------------------------------------CGCAUCAGAGCUCCGAGCAUUAA--- 2       23
-------------------------------------------------------------------------------AUCAGAGCUCCGAGCAUUAA--- 1       20
--------------------------------------------------------------------------------UCAGAGCUCCGAGCAUUAA--- 1       19
-UAUUAAUGCUCGGAGCUCUGAU------------------------------------------------------------------------------- 1       22
-UAUUAAUGCUCGGAGCUCUGA-------------------------------------------------------------------------------- 1       21
-UAUUAAUGCUCGGAGCUCUG--------------------------------------------------------------------------------- 1       20
--AUUAAUGCUCGGAGCUCUGAU------------------------------------------------------------------------------- 2       21
----UAAUGCUCGGAGCUCUGA-------------------------------------------------------------------------------- 2       18
-----AAUGCUCGGAGCUCUGAUGCGAACU------------------------------------------------------------------------ 1       25
-----AAUGCUCGGAGCUCUGAUGCGA--------------------------------------------------------------------------- 1       22
------AUGCUCGGAGCUCUGAUG------------------------------------------------------------------------------ 1       18
-------UGCUCGGAGCUCUGAUGCGA--------------------------------------------------------------------------- 1       20
--------GCUCGGAGCUCUGAUGCGAACU------------------------------------------------------------------------ 2       22
----------UCGGAGCUCUGAUGCGAACUGUG--------------------------------------------------------------------- 1       23
------------GGAGCUCUGAUGCGAACUG----------------------------------------------------------------------- 1       19
--------------AGCUCUGAUGCGAACUGUGGG------------------------------------------------------------------- 6       21
----------------CUCUGAUGCGAACUGUGGGUG----------------------------------------------------------------- 4       21
-----------------UCUGAUGCGAACUGUGGGUGG---------------------------------------------------------------- 2       21
----------------------UGCGAACUGUGGGUGGACGCU----------------------------------------------------------- 2       21
------------------------------------------------------------------ACCCACAUUUCGCAUCAGAGCU-------------- 1       22
-------------------------------------------------------------------------UUUCGCAUCAGAGCUCCG----------- 6       18
-------------------------------------------------------------------------UUUCGCAUCAGAGCUCCGAGCAUUAAUAA 2       29
--------------------------------------------------------------------------UUCGCAUCAGAGCUCCGAGCAUU----- 1       23
---------------------------------------------------------------------------UCGCAUCAGAGCUCCGAGCAUUAAUA- 1       26
----------------------------------------------------------------------------CGCAUCAGAGCUCCGAGC-------- 8       18
----------------------------------------------------------------------------CGCAUCAGAGCUCCGAGCA------- 2       19
-----------------------------------------------------------------------------GCAUCAGAGCUCCGAGCAUU----- 3       20
-----------------------------------------------------------------------------GCAUCAGAGCUCCGAGCAUUAAU-- 1       23
------------------------------------------------------------------------------CAUCAGAGCUCCGAGCAUUA---- 1       20
------------------------------------------------------------------------------CAUCAGAGCUCCGAGCAUUAAU-- 3       22
------------------------------------------------------------------------------CAUCAGAGCUCCGAGCAUUAAUA- 1       23
--------------------------------------------------------------------------------UCAGAGCUCCGAGCAUUAAUAA 5       22
---------------------------------------------------------------------------------CAGAGCUCCGAGCAUUAAU-- 1       19
```

```
                            C             U  A   G
UUAUUAAUGCuCGGAGCUCUGAUGCGAA UgUGGGUGGACGC GG AAU U
AAUAAUUAcGAGCCUCGAGACUACGCUU ACACCCACCUGCG CC UUA U
                            u             U  G   G
```

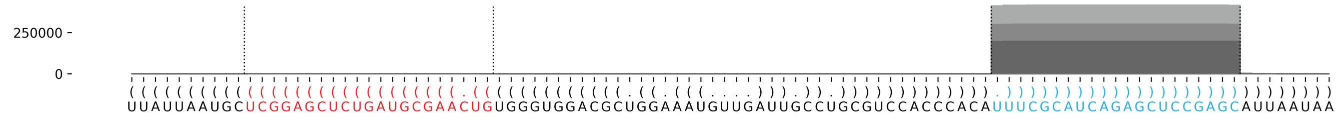

D. firmibasis dfi-mir-1195

```
(((.(((.(((((((((.(((((((((((((((((((..(((.(((((..((((((((....))))))))...........))))).)))..))))))))))))))))))).))))))))).))).)))          -69.84 kcal/mol
AGGUGAUCGUuGAUGUCGCCAAUGUAAUAAcAGAGGUUAAUUAUAUUAAAUAAUAUUUCAAUAAAUAUUAACAUUAACUUCUAAUACAAUAUACCUCUGUuAUUACAUUGGGGAUAUCAAcCGUCCCCU          count    length
Libraries combined          Precision: [ total = 97.7% | 5p-arm = 97.8% | 3p-arm = 34.5% ]
----------UGAUGUCGCCAAUGUAAUAAC--------------------------------------------------------------------------------------------------          530751   21
----------UGAUGUCGCCAAUGUAAUAA---------------------------------------------------------------------------------------------------          6469     20
---UGAUCGUUGAUGUCGCCAAUG---------------------------------------------------------------------------------------------------------          6127     21
----------UGAUGUCGCCAAUGUAAUAACA-------------------------------------------------------------------------------------------------          3143     22
---------UUGAUGUCGCCAAUGUAAUAAC--------------------------------------------------------------------------------------------------          1647     22
-----------GAUGUCGCCAAUGUAAUAAC--------------------------------------------------------------------------------------------------          1735     20
---------UUGAUGUCGCCAAUGUAAUAA---------------------------------------------------------------------------------------------------          701      21
------------AUGUCGCCAAUGUAAUAACAG------------------------------------------------------------------------------------------------          587      21
---------UUGAUGUCGCCAAUGUAAU-----------------------------------------------------------------------------------------------------          477      19
----------UGAUGUCGCCAAUGUAAU-----------------------------------------------------------------------------------------------------          483      18
-----------------------------------------------------------------------------------------------------AUUACAUUGGGGAUAUCAACC-------          398      21
------------AUGUCGCCAAUGUAAUAAC--------------------------------------------------------------------------------------------------          340      19
----------UGAUGUCGCCAAUGUAAUA----------------------------------------------------------------------------------------------------          305      19
----------------------------------------------------------------------------------------------------UAUUACAUUGGGGAUAUCAAC--------          188      21
---------UUGAUGUCGCCAAUGUAAUA----------------------------------------------------------------------------------------------------          165      20
-----------GAUGUCGCCAAUGUAAUAACA-------------------------------------------------------------------------------------------------          170      21
----------------------------------------------------------------------------------------------------UAUUACAUUGGGGAUAUCAA---------          77       20
------UCGUUGAUGUCGCCAAUGUA-------------------------------------------------------------------------------------------------------          68       20
-----------------------------------------------------------------------------------------------------AUUACAUUGGGGAUAUCAAC--------          55       20
-----------GAUGUCGCCAAUGUAAUAA---------------------------------------------------------------------------------------------------          59       19
-----AUCGUUGAUGUCGCCAAUGUA-------------------------------------------------------------------------------------------------------          39       21
----------UGAUGUCGCCAAUGUAAUAACAG------------------------------------------------------------------------------------------------          40       23
---UGAUCGUUGAUGUCGCCAA-----------------------------------------------------------------------------------------------------------          31       19
--------------GUCGCCAAUGUAAUAACAGAG----------------------------------------------------------------------------------------------          16       21
------------AUGUCGCCAAUGUAAUAACAGA-----------------------------------------------------------------------------------------------          27       22
-----------------------------------------------------------------------------------------------UCUGUUAUUACAUUGGGGAU--------------          12       20
---UGAUCGUUGAUGUCGCCAAU----------------------------------------------------------------------------------------------------------          20       20
-------------UGUCGCCAAUGUAAUAAC--------------------------------------------------------------------------------------------------          9        18
-----------------------------------------------------------------------------------------------------AUUACAUUGGGGAUAUCAA---------          18       19
---UGAUCGUUGAUGUCGCCA------------------------------------------------------------------------------------------------------------          9        18
----------------------------------------------------------------------------------------------------UAUUACAUUGGGGAUAUC-----------          9        18
-----AUCGUUGAUGUCGCCAAUG---------------------------------------------------------------------------------------------------------          12       19
-------------UGUCGCCAAUGUAAUAACAGAG----------------------------------------------------------------------------------------------          5        22
-----------GAUGUCGCCAAUGUAAUAACAG------------------------------------------------------------------------------------------------          8        22
--------------GUCGCCAAUGUAAUAACAG------------------------------------------------------------------------------------------------          5        19
--------------------------------------------------------------------------------------------------GUUAUUACAUUGGGGAUAUC-----------          4        20
---------------------------------------------------------------------------------------------------UUAUUACAUUGGGGAUAUCAA---------          4        21
-----------------------------------------------------------------------------------------------------AUUACAUUGGGGAUAUCAACCG------          7        22
---UGAUCGUUGAUGUCGCCAAUGU--------------------------------------------------------------------------------------------------------          11       22
------UCGUUGAUGUCGCCAAUG---------------------------------------------------------------------------------------------------------          2        18
----------UGAUGUCGCCAAUGUAAUAACAGA-----------------------------------------------------------------------------------------------          4        24
-----------GAUGUCGCCAAUGUAAUA----------------------------------------------------------------------------------------------------          6        18
------------AUGUCGCCAAUGUAAUAACA-------------------------------------------------------------------------------------------------          3        20
---------------UCGCCAAUGUAAUAACAGAGG---------------------------------------------------------------------------------------------          8        21
-------------------------------------------------------------------------------------------------UGUUAUUACAUUGGGGAUAUC-----------          8        21
----------------------------------------------------------------------------------------------------UAUUACAUUGGGGAUAUCAACC-------          5        22
------------------------------------------------------------------------------------------------------UUACAUUGGGGAUAUCAA---------          2        18
------------------------------------------------------------------------------------------------------UUACAUUGGGGAUAUCAAC--------          3        19
------------------------------------------------------------------------------------------------------UUACAUUGGGGAUAUCAACCG------          3        21
---UGAUCGUUGAUGUCGCCAAUGUA-------------------------------------------------------------------------------------------------------          3        23
----GAUCGUUGAUGUCGCCAAUG---------------------------------------------------------------------------------------------------------          7        20
------UCGUUGAUGUCGCCAAUGUAAU-----------------------------------------------------------------------------------------------------          1        22
-----------------------------------------------------------------------------------------------UCUGUUAUUACAUUGGGGAUA-------------          5        21
```

D. firmibasis dfi-mir-1195 continued

```
(((.(((.(((((((((.(((((((((((((((((((..(((.(((((..((((((((....))))))))...........))))).)))..))))))))))))))))))).))))))))).))).)))   -69.84 kcal/mol
AGGUGAUCGUuGAUGUCGCCAAUGUAAUAAcAGAGGUUAAUUAUAUUAAAUAAUAUUUCAAUAAAUAUUAACAUUAACUUCUAAUACAAUAUACCUCUGUuAUUACAUUGGGGAUAUCAAcCGUCCCCU   count   length
-----------------------------------------------------------------------------------------------------AUUACAUUGGGGAUAUCAACCGU-----   1   23
-----------------------------------------------------------------------------------------------------AUUACAUUGGGGAUAUCA----------   2   18
AGGUGAUCGUUGAUGUCGC--------------------------------------------------------------------------------------------------------------   1   19
--GUGAUCGUUGAUGUCGCCAAUG---------------------------------------------------------------------------------------------------------   1   22
-----AUCGUUGAUGUCGCCAAUGU--------------------------------------------------------------------------------------------------------   2   20
------UCGUUGAUGUCGCCAAUGUAA------------------------------------------------------------------------------------------------------   3   21
-------CGUUGAUGUCGCCAAUGUAAU-----------------------------------------------------------------------------------------------------   2   21
--------GUUGAUGUCGCCAAUGUAAU-----------------------------------------------------------------------------------------------------   1   20
--------GUUGAUGUCGCCAAUGUAAUA----------------------------------------------------------------------------------------------------   1   21
---------UUGAUGUCGCCAAUGUAA------------------------------------------------------------------------------------------------------   1   18
------------AUGUCGCCAAUGUAAUAA---------------------------------------------------------------------------------------------------   1   18
-------------UGUCGCCAAUGUAAUAACAGA-----------------------------------------------------------------------------------------------   4   21
--------------GUCGCCAAUGUAAUAACAGA-----------------------------------------------------------------------------------------------   1   20
---------------UCGCCAAUGUAAUAACAGAG----------------------------------------------------------------------------------------------   1   20
--------------------------------------------------------------------------------------CAAUAUACCUCUGUUAUUACAU---------------------   1   22
-----------------------------------------------------------------------------------------UAUACCUCUGUUAUUACAUUG-------------------   2   21
------------------------------------------------------------------------------------------AUACCUCUGUUAUUACAUUG-------------------   1   20
-----------------------------------------------------------------------------------------------UCUGUUAUUACAUUGGGGA---------------   1   19
-----------------------------------------------------------------------------------------------UCUGUUAUUACAUUGGGGAUAU------------   1   22
----------------------------------------------------------------------------------------------------UAUUACAUUGGGGAUAUCAACCGUC----   1   25
------------------------------------------------------------------------------------------------------UUACAUUGGGGAUAUCAACC-------   1   20
```

```
   U   C         G                   UA   A     AA                  C
AGG GAU GUuGAUGUC CCAAUGUAAUAAcAGAGGU  AUU UAUUA  ---------UAAUAUUU A
UCC CUG cAACUAUAG GGUUACAUUAuUGUCUCCA  UAA AUAAU           AUUAUAAA A
   C   C         G                   UA   C     CUUCAAUUACA         U
```

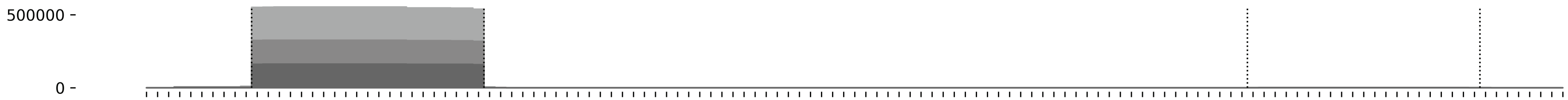


```
(((.(((.(((((((((.(((((((((((((((((((..(((.(((((..((((((((....))))))))...........))))).)))..))))))))))))))))))).))))))))).))).)))
AGGUGAUCGUUGAUGUCGCCAAUGUAAUAACAGAGGUUAAUUAUAUUAAAUAAUAUUUCAAUAAAUAUUAACAUUAACUUCUAAUACAAUAUACCUCUGUUAUUACAUUGGGGAUAUCAACCGUCCCCU
```

D. firmibasis dfi-mir-1196

```
(((((((((((((((((((((.((((((((((((((((((((((((...((((........)))).))))).....))))))))))))))))))).)))))))))))))))))))))   -75.37 kcal/mol
AAAAAAAACAuAGUGAAGAUGGGUAGAAGAuAAAAGCUGGUUUUGAAAAUUCAGAGAAUGGUGGGGUUAGAAAAUUACCAGCUUUUAUcUUCUACACAUCUUCACUAUgUUUUUUUU   count   length
Libraries combined          Precision: [ total = 76.8% | 5p-arm = 73.1% | 3p-arm = 79.1% ]
----------UAGUGAAGAUGGGUAGAAGAU--------------------------------------------------------------------------------------   18      21
----------------------------------------------------------------------------------------CUUCUACACAUCUUCACUAUG--------   34      21
--------------------------------------------------------------------------------------AUCUUCUACACAUCUUCACUA----------   2       21
-----------------------------------------------------------------------------------------UUCUACACAUCUUCACUAUGU-------   3       21
----------UAGUGAAGAUGGGUAGAAGAUA-------------------------------------------------------------------------------------   1       22
-----------------GAUGGGUAGAAGAUAAAAGCU-------------------------------------------------------------------------------   3       21
------------------AUGGGUAGAAGAUAAAAGCU-------------------------------------------------------------------------------   1       20
-----------AGUGAAGAUGGGUAGAAGAU--------------------------------------------------------------------------------------   1       20
----------------AGAUGGGUAGAAGAUAAAAGC--------------------------------------------------------------------------------   2       21
------------------------------------------------------------------------AAUUACCAGCUUUUAUCUUCUA-----------------------   1       22
-------------------------------------------------------------------------------------UAUCUUCUACACAUCUUCACU-----------   2       21
---------------------------------------------------------------------------------------UCUUCUACACAUCUUCACUAUG--------   1       22
Library = 1        Precision: [ total = 64.7% | 5p-arm = 75.0% | 3p-arm = 55.6% ]
----------UAGUGAAGAUGGGUAGAAGAU--------------------------------------------------------------------------------------   5       21
----------------------------------------------------------------------------------------CUUCUACACAUCUUCACUAUG--------   5       21
--------------------------------------------------------------------------------------AUCUUCUACACAUCUUCACUA----------   2       21
-----------------------------------------------------------------------------------------UUCUACACAUCUUCACUAUGU-------   2       21
----------UAGUGAAGAUGGGUAGAAGAUA-------------------------------------------------------------------------------------   1       22
-----------------GAUGGGUAGAAGAUAAAAGCU-------------------------------------------------------------------------------   1       21
------------------AUGGGUAGAAGAUAAAAGCU-------------------------------------------------------------------------------   1       20
Library = 2        Precision: [ total = 87.5% | 5p-arm = 71.4% | 3p-arm = 100.0% ]
----------------------------------------------------------------------------------------CUUCUACACAUCUUCACUAUG--------   9       21
----------UAGUGAAGAUGGGUAGAAGAU--------------------------------------------------------------------------------------   5       21
----------------AGAUGGGUAGAAGAUAAAAGC--------------------------------------------------------------------------------   1       21
-----------------GAUGGGUAGAAGAUAAAAGCU-------------------------------------------------------------------------------   1       21
Library = 3        Precision: [ total = 77.8% | 5p-arm = 72.7% | 3p-arm = 80.0% ]
----------------------------------------------------------------------------------------CUUCUACACAUCUUCACUAUG--------   20      21
----------UAGUGAAGAUGGGUAGAAGAU--------------------------------------------------------------------------------------   8       21
-------------------------------------------------------------------------------------UAUCUUCUACACAUCUUCACU-----------   2       21
-----------AGUGAAGAUGGGUAGAAGAU--------------------------------------------------------------------------------------   1       20
----------------AGAUGGGUAGAAGAUAAAAGC--------------------------------------------------------------------------------   1       21
-----------------GAUGGGUAGAAGAUAAAAGCU-------------------------------------------------------------------------------   1       21
------------------------------------------------------------------------AAUUACCAGCUUUUAUCUUCUA-----------------------   1       22
---------------------------------------------------------------------------------------UCUUCUACACAUCUUCACUAUG--------   1       22
-----------------------------------------------------------------------------------------UUCUACACAUCUUCACUAUGU-------   1       21
```

```
                     G                             AAA     GAG
AAAAAAAACAuAGUGAAGAUG GUAGAAGAuAAAAGCUGGU-----UUUGA   UUCA   A
UUUUUUUUgUAUCACUUCUAC CAUCUUcUAUUUUCGACCA     AGAUU --GGGU   A
                     A                   UUAAA     G      GGU
```

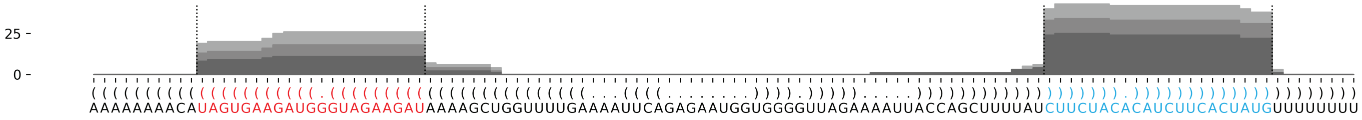

Supplement: gkae109_supplemental_files [file gkae109_supplemental_files.zip › Supplementary Data.pdf]
