# Supplementary material for: Evolution of microRNAs in Amoebozoa and implications for the origin of multicellularity
Source: Nucleic Acids Res. 2024 Feb 20;52(6):3121–36. doi: 10.1093/nar/gkae109 (PMC11014262; doi:10.1093/nar/gkae109)
Supplement: gkae109_supplemental_files [file gkae109_supplemental_files.zip › Supplementary figures.pdf]

Table of contents

Supplementary Fig. 1 2

Supplementary Fig. 2 4

Supplementary Fig. 3 6

Supplementary Fig. 4 7

Supplementary Fig. 5 8

Supplementary Fig. 6 9

Supplementary Fig. 7 11

Supplementary Fig. 8 12

Supplementary Fig. 9 13

Supplementary Fig. 10 14

Supplementary Fig. 11 15

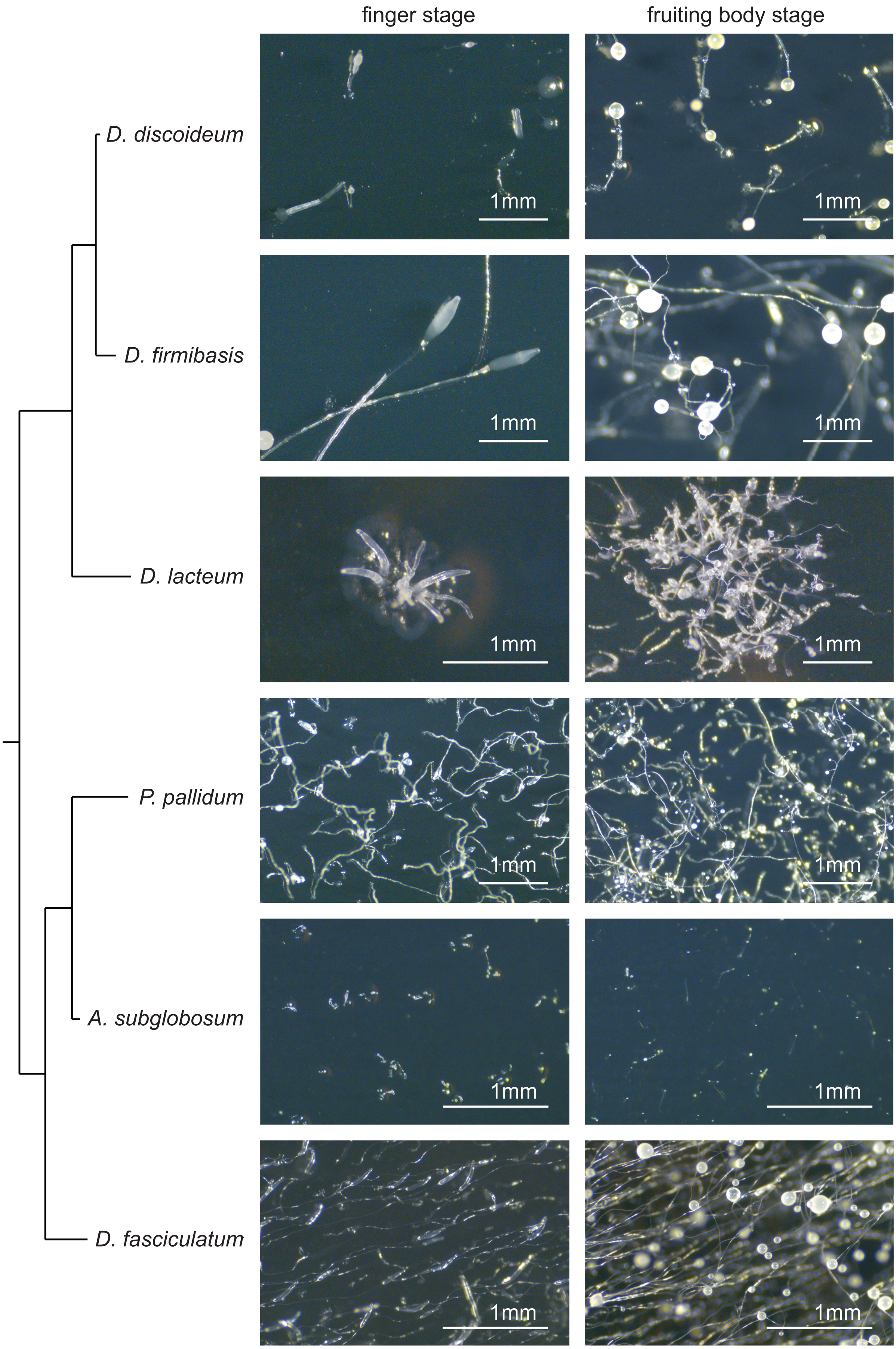
finger stage
fruiting body stage
D. discoideum
D. firmibasis
D. lacteum
P. pallidum
A. subglobosum
D. fasciculatum
1mm

Supplementary Fig. 1 | Phenotypes of studied Dictyostelia species at different stages of multicellular development. Small RNAs were sequenced from a mix of vegetative cells (not pictured) and during stages of multicellular development: the finger/slug stage (first column) and fruiting body stage (second column). Each of the social amoebae display distinct phenotypes at the different stages.

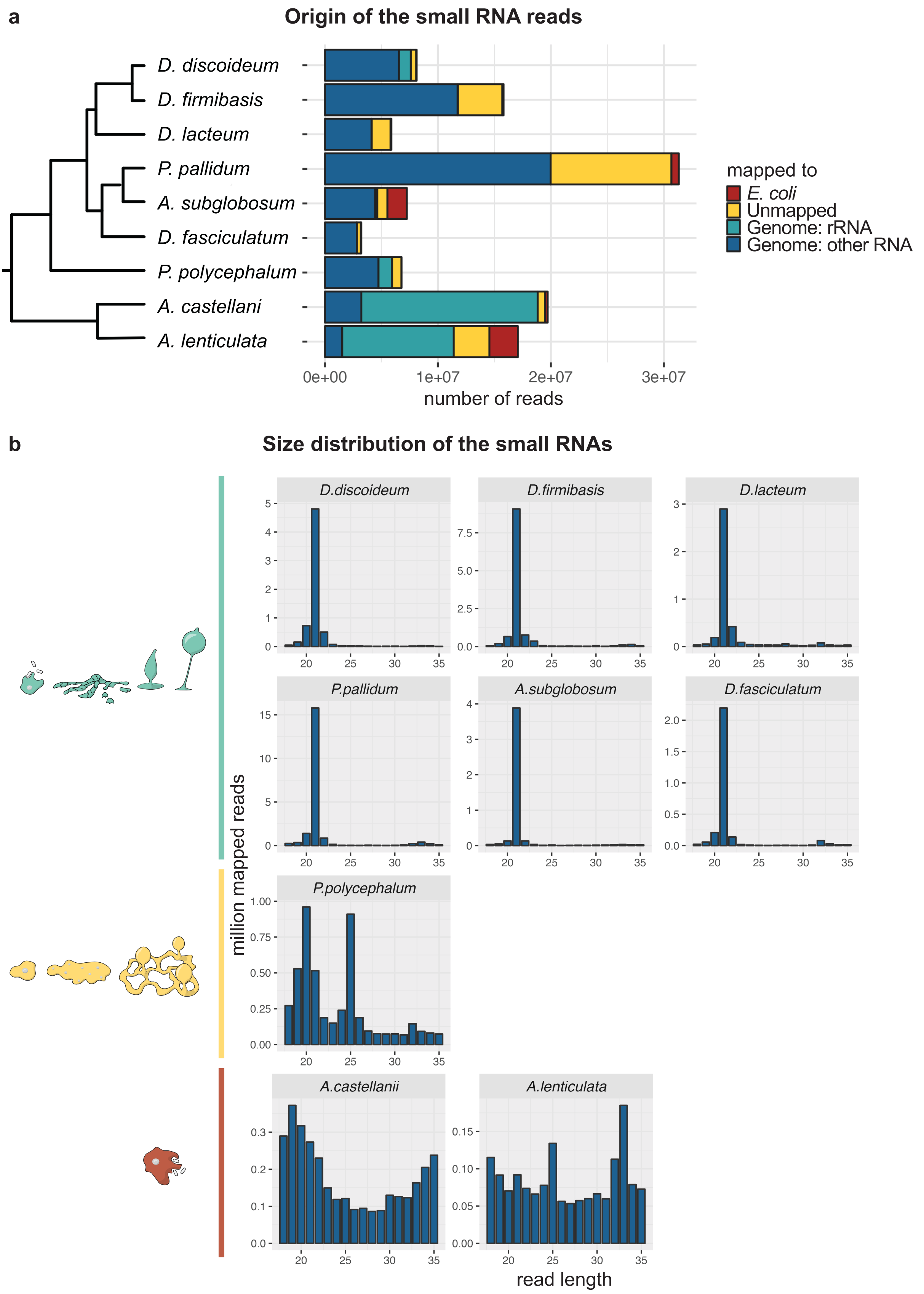

Supplementary Fig. 2 | Origin and length profile of isolated small RNAs. a Number of reads from small RNA sequencing mapping with maximum one mismatch to the food bacterium *E. coli* or the genomes of the indicated amoebae for which the reads were further subdivided into sequences mapping to the ribosomal RNA

(Genome_rRNA) or any other part of the genome (Genome_otherRNA). Reads that could not be mapped are marked as Unmapped. Number of reads are displayed in power of 10. **b** sRNA length profile of reads mapping to the genome, but not the ribosomal RNA (dark-blue in **a**). The characteristic phenotypes of the different amoebae included in the study are shown on the left, with Dictyostelia in green, Myxomycetes in yellow, and Discosea in red.

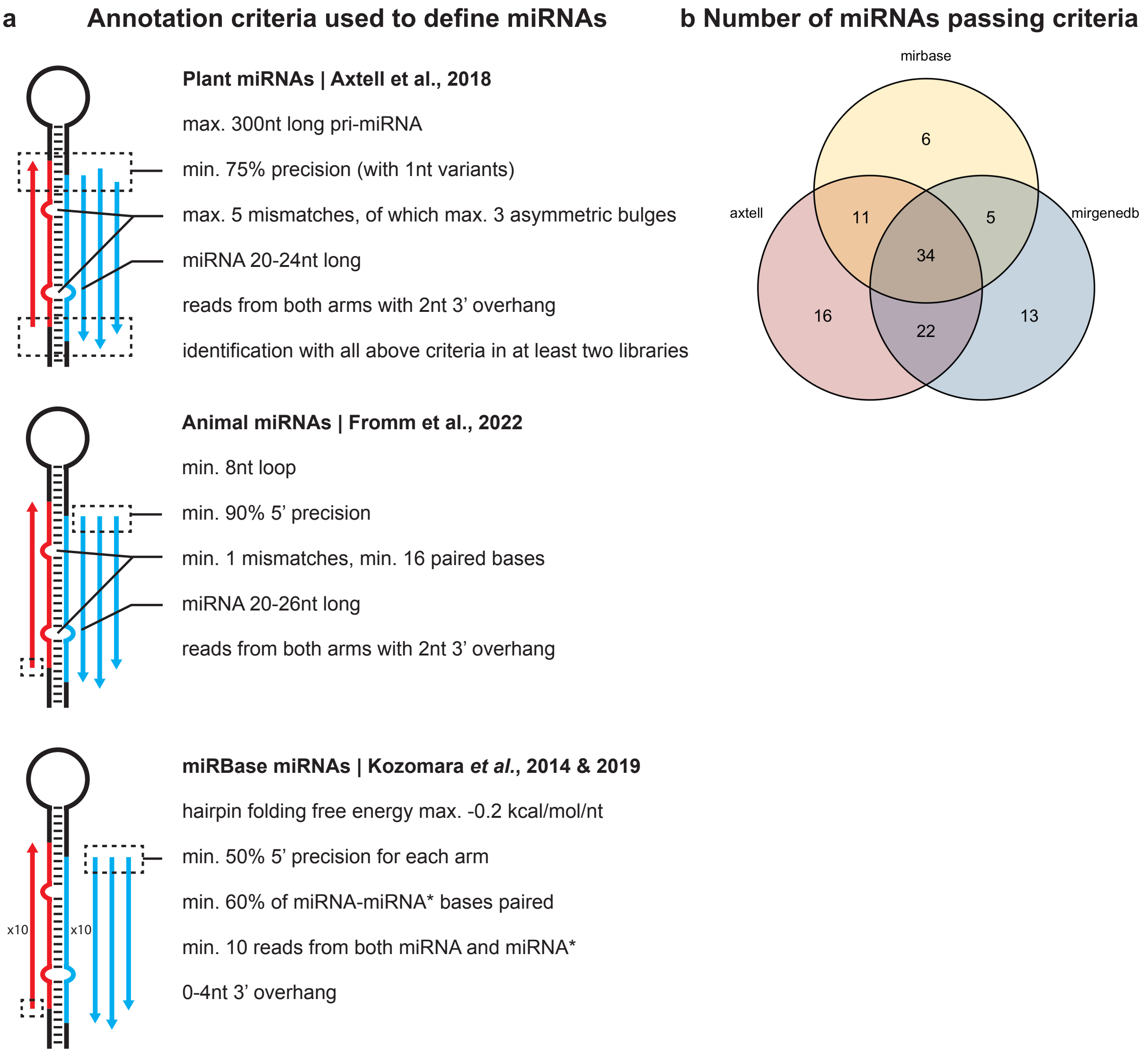

Supplementary Fig. 3 | Criteria used for miRNA annotation. **a** Graphical summary of the different sets of miRNA annotation criteria used in this study. ‘Precision’ is the percentage of miRNA-5p and miRNA-3p reads (e.g. ‘precise reads’) versus total reads mapped on the miRNA-hairpin, and is calculated differently for the different sets of criteria. For plant miRNAs (Axtell et al., 2018), all reads that map to the miRNA-5p or miRNA-3p with 1-nt variants for both the 5’ and 3’-ends are considered ‘precise’. For animal miRNAs (Fromm et al., 2022), only reads that map precisely to the 5’-end of the miRNA-5p or miRNA-3p are included. 3’-end variation is allowed. For miRBase miRNAs (Kozomara et al., 2014 & 2019), reads are also required to map precisely to the 5’-end of the miRNAs. Here however, calculations are done on the miRNA-5p-arm and miRNA-3p-arm separately, yielding two precision percentages per miRNA, both of which have to be larger than 50% **b** Venn diagram of number of miRNAs that passed all criteria for plant miRNAs (Axtell, red), animal miRNAs (mirgenedb, blue), or miRBase miRNAs (miRbase, yellow). Numbers based on data from *D. discoideum*, *D. firmibasis*, *D. lacteum*, *P. pallidum*, *A. subglobosum*, *P. polycephalum, A. castellanii*, and *A. lenticulata*.

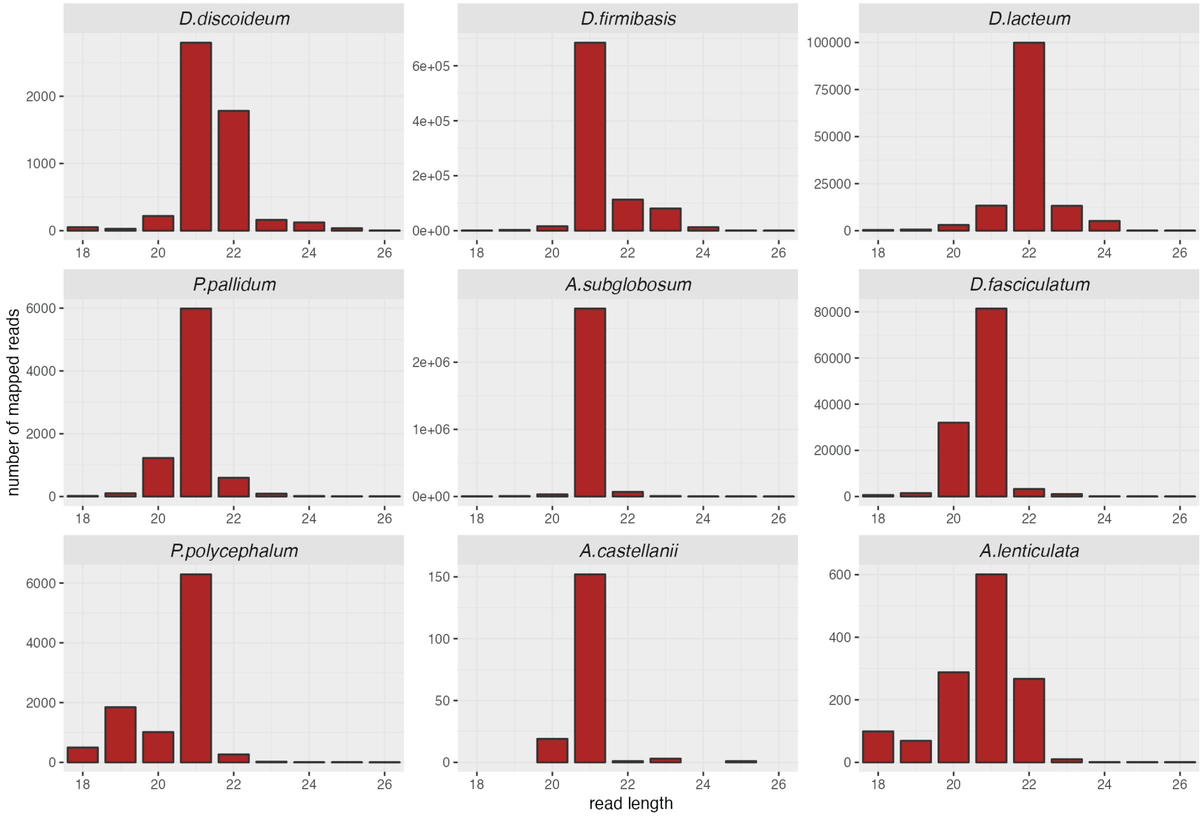

Supplementary Fig. 4 | Length distribution of sRNAs mapping to miRNA-loci. Histogram of read lengths mapping to loci of miRNA-hairpins. For *D. firmibasis*, mapping was done to the de-novo sequenced genome.

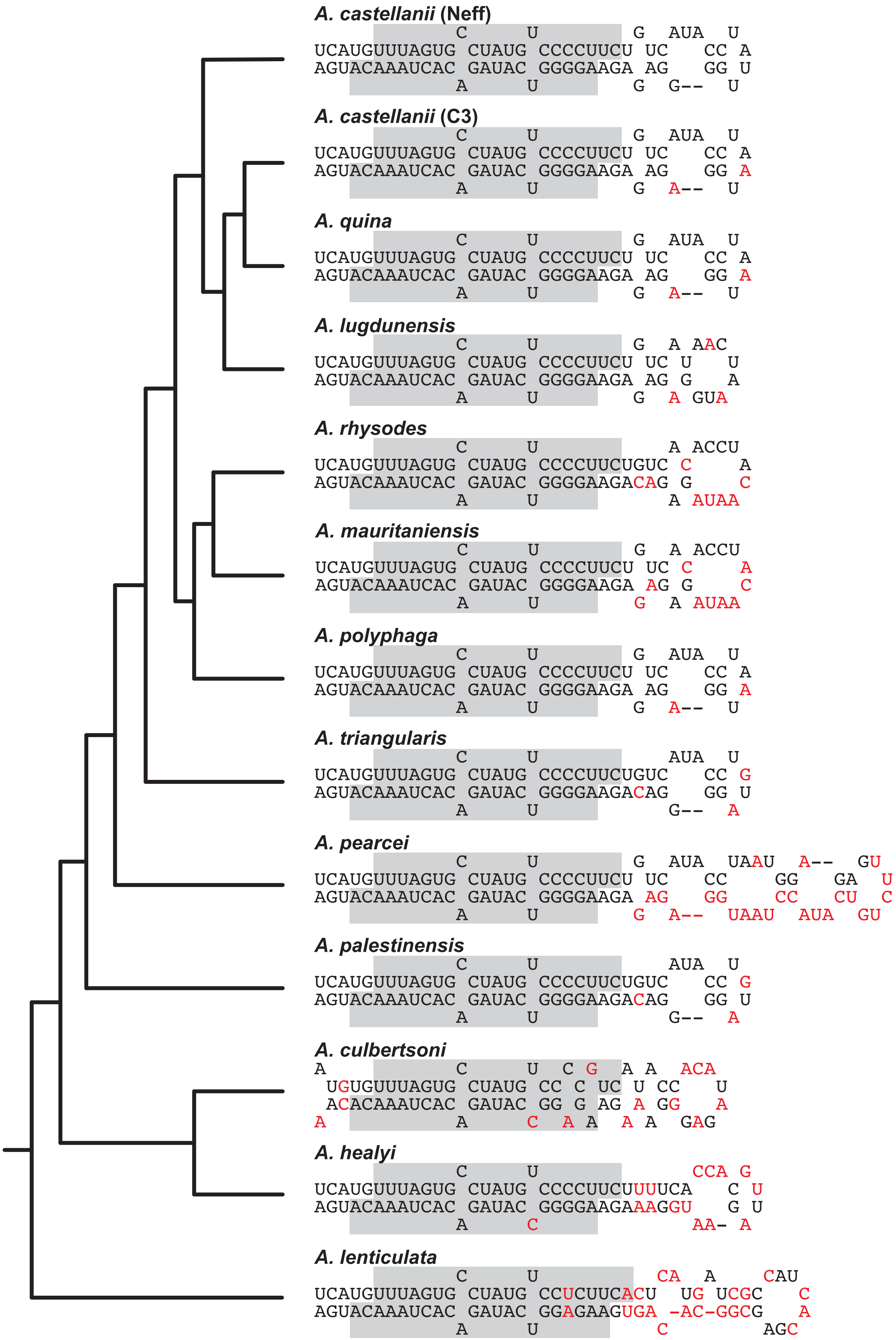

Supplementary Fig. 5 | Predicted secondary structure of mir-1228 in *Acanthamoeba* spp. Location of the predicted miRNA duplex indicated with a grey background. Nucleotides which differ relative to aca-mir-1228 (Neff; top) have been indicated red. Dendrogram showing phylogeny of the *Acanthamoebae* as in Fig. 2c.

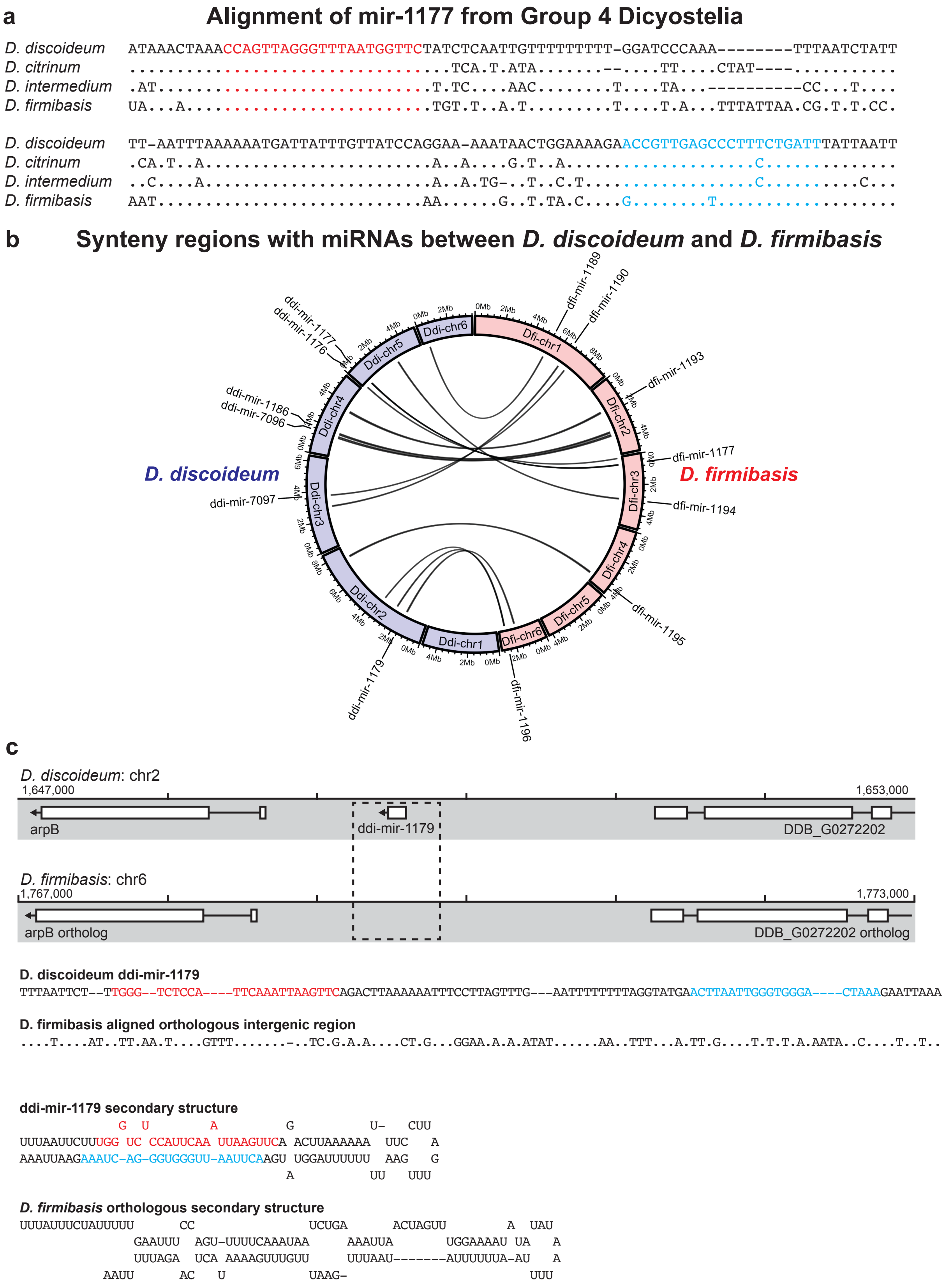

Supplementary Fig. 6 | miRNA conservation in Group 4 Dictyostelia and miRNA evolution between *D. discoideum* and *D. firmibasis*. **a** Alignment of the mir-1177 orthologs found in *D. discoideum*, *D. citrinum*, *D. intermedium*, and *D. firmibasis*. The DNA sequences from *D. citrinum* and *D. intermedium* were resolved by sequencing the genomic region in which the ortholog is located. The *D. firmibasis* ortholog was resolved by

whole genome sequencing. Positions of the mir-1177-5p and mir-1177-3p are indicated in red and cyan, respectively. **b** Circos plot of the *D. firmibasis* (pink) and *D. discoideum* (purple) main contigs, with links showing syntenic regions containing a miRNA in either *D. firmibasis* or *D. discoideum*. (Note that ddi-mir-1177 is the only miRNA identified to be conserved between the two dictyostelids). The name of the miRNAs present in the syntenic regions are shown on the outside of the plot. **c** Intergenic region containing ddi-mir-1179 in *D. discoideum*, and below the aligned syntenic region in *D. firmibasis*. The DNA sequence in the dashed box was further aligned and the hypothetical ncRNA folded in silico, which yielded the displayed hairpin-like structure. The positions of ddi-mir-1179-5p and ddi-mir-1179-3p are indicated in red and cyan, respectively, in both the DNA alignment and RNA secondary structure.

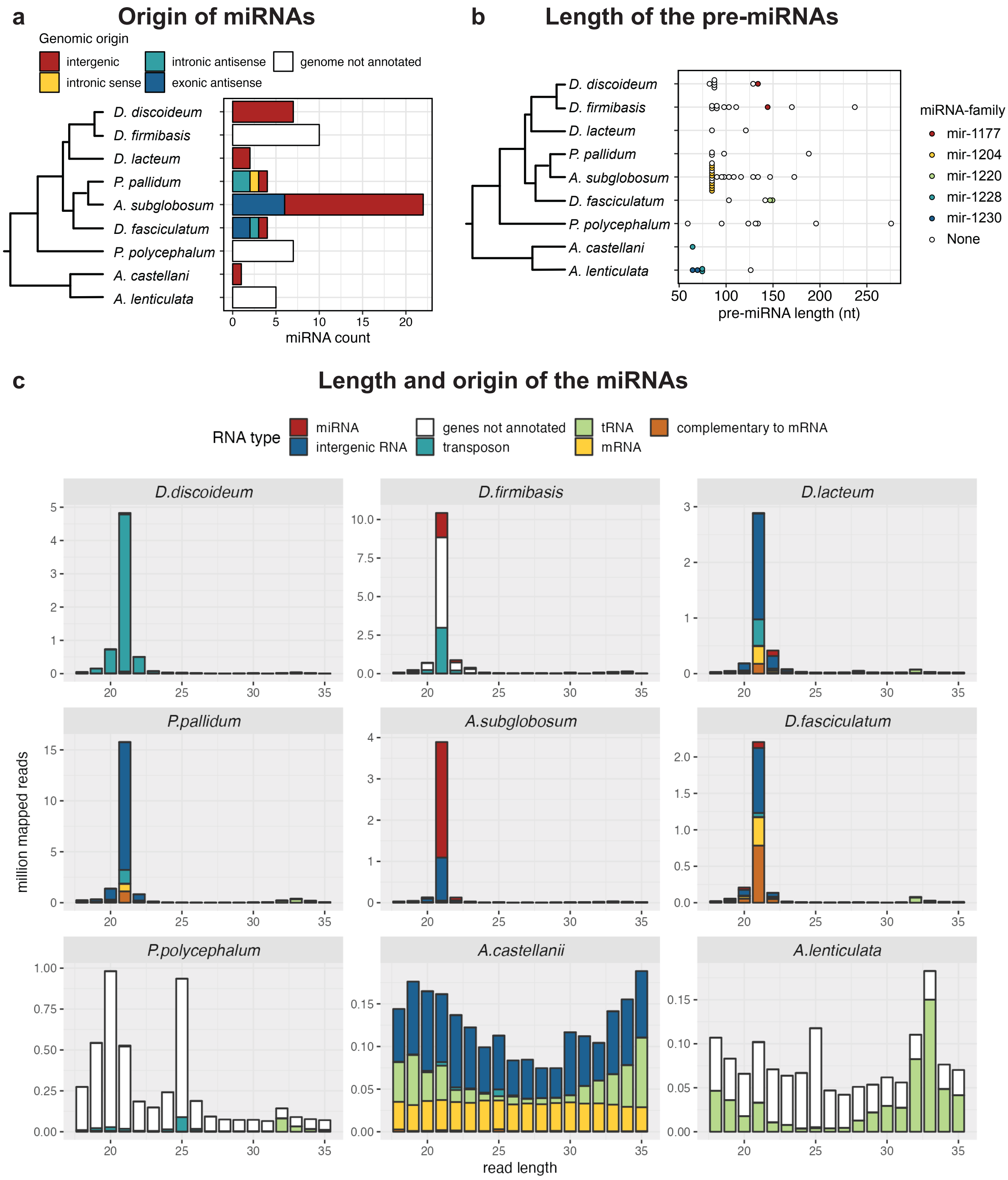

Supplementary Fig. 7 | Genomic origin and size of the miRNAs and sRNAs in Amoebozoa. **a** Barchart displaying the number of all discovered miRNAs in this study and their origin in the respective amoebae, with a simplified phylogenetic dendrogram. Genome annotations are not available for *D. firmibasis*, *P. polycephalum* and *A. lenticulata.* **b** miRNAs plotted based on their pre-miRNA length. miRNAs of the same family are plotted with a single color. **c** Length distribution of the small RNAs sequenced from each of the studied amoebae, as in Supplementary Fig. 2b, but with annotations, showing where the sRNAs map to the genome. Annotation of transposons is based on blast search with transposable elements available from Repbase. Annotations of tRNAs and other known non-coding RNAs was performed using Infernal with models based on the Rfam database. Number of sRNAs mapping to spliceosomal RNAs and other ncRNAs were omitted since they were very low, but they can be accessed through GitHub.

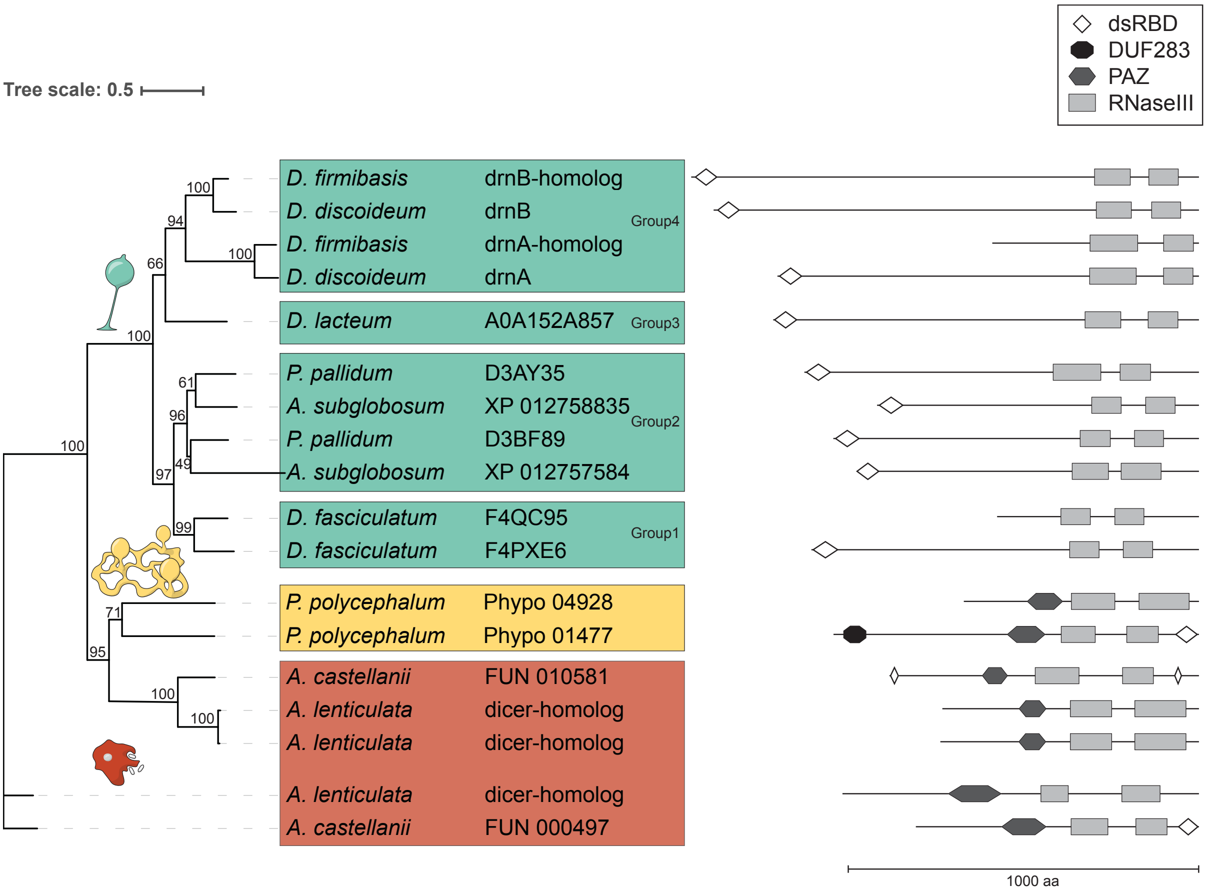

Supplementary Fig. 8 | Phylogeny of the Dicer proteins in Amoebozoa. Dicer homologs identified through search with OrthoFinder (Emms and Kelly, 2019) on the proteomes of all included species, except *D. firmibasis* and *A. lenticulata* for which no proteomes are available. Homologs for *D. firmibasis* and *A. lenticulata* were annotated by an additional BLAST search. Numbers after species names are the protein identifiers. For *D. discoideum*, *D. lacteum*, *P. pallidum*, *A. subglobosum* and *D. fasciculatum*, proteins can be accessed at UniProt. The domain structure of the homologs is shown (right). All harbor two Ribonuclease III domains (RNase III) and additionally some contain PAZ and/or double stranded RNA-binding domains (dsRBD). The amino acid sequences were aligned, trimmed and used to build a phylogenetic tree. The consensus tree is shown with bootstrap supports (%) on the branches. The scale shows evolutionary distance, defined as the number of nucleotide substitutions per site. Amoebae (and their Dicer homologs) belonging to Dictyostelia, Myxomycetes, and Discosea are marked with cyan, yellow, and orange back ground, respectively.

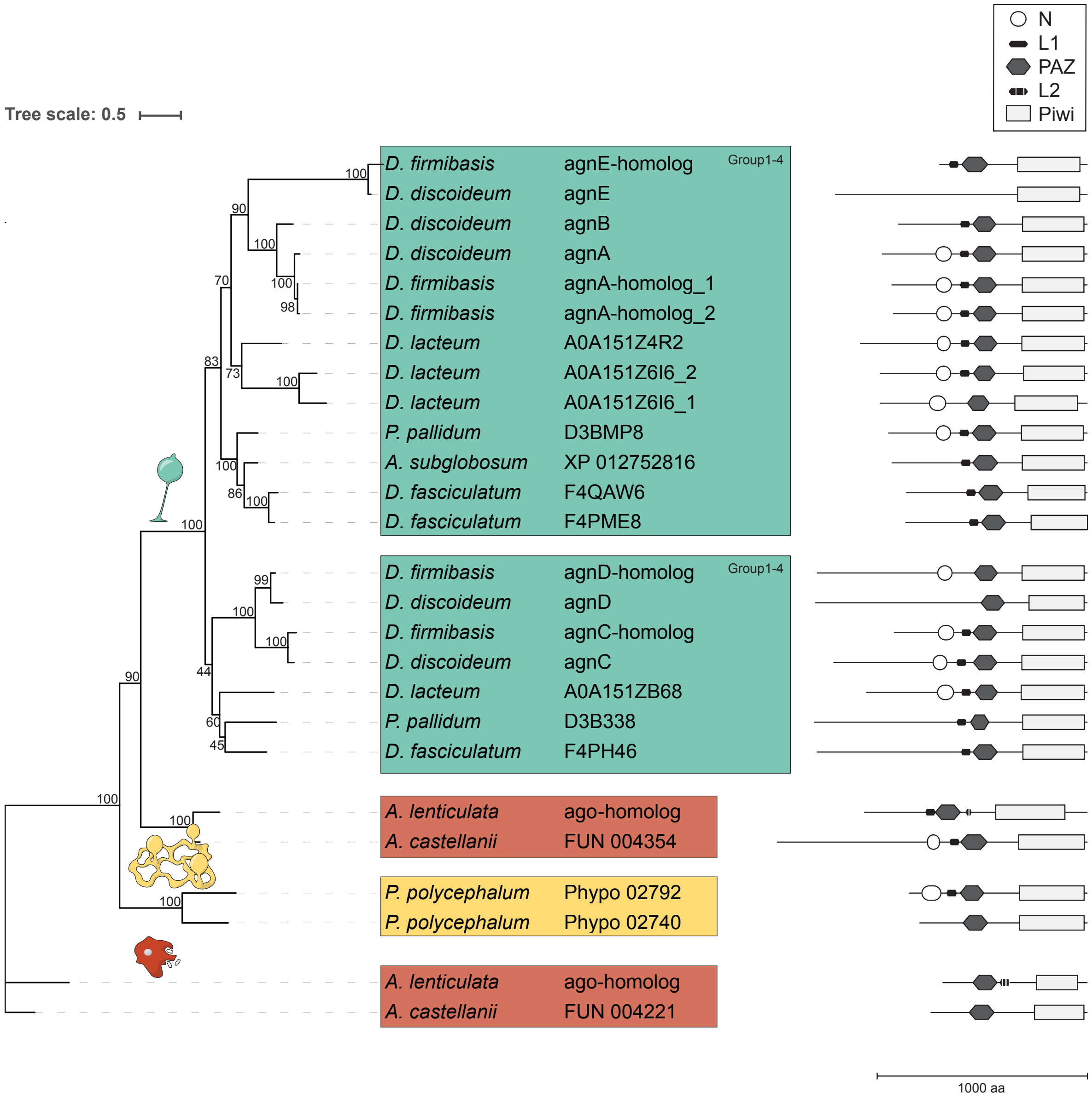

Supplementary Fig. 9 | Phylogeny of the Argonaute proteins in Amoebozoa. Argonaute homologs identified through search with OrthoFinder (Emms and Kelly, 2019) on the proteomes of all included species, except *D. firmibasis* and *A. lenticulata* for which no proteomes are available. Homologs for *D. firmibasis* and *A. lenticulata* were annotated by an additional BLAST search. Numbers after species names are the protein identifiers. For *D. discoideum*, *D. lacteum*, *P. pallidum*, *A. subglobosum* and *D. fasciculatum*, proteins can be accessed at UniProt. The domain structure of the homologs is shown (right). All identified Argonautes feature a single PAZ domain, followed by a single Piwi domain. Identified Argonaute N-terminal domains (N) as well as linker domains (L1, L2) are shown as well. The amino acid sequences of the identified Argonautes were aligned, trimmed and used to build a phylogenetic tree. The consensus tree is shown with bootstrap supports (%) on the branches. The scale shows evolutionary distance, defined as the number of nucleotide substitutions per site. Dictyostelia feature two main Argonaute orthologs which are present in all analyzed species, and which were probably both present in their last common ancestor. Amoebae (and their Argonaute homologs) belonging to Dictyostelia, Myxomycetes, and Discosea are marked with cyan, yellow, and orange back ground, respectively.

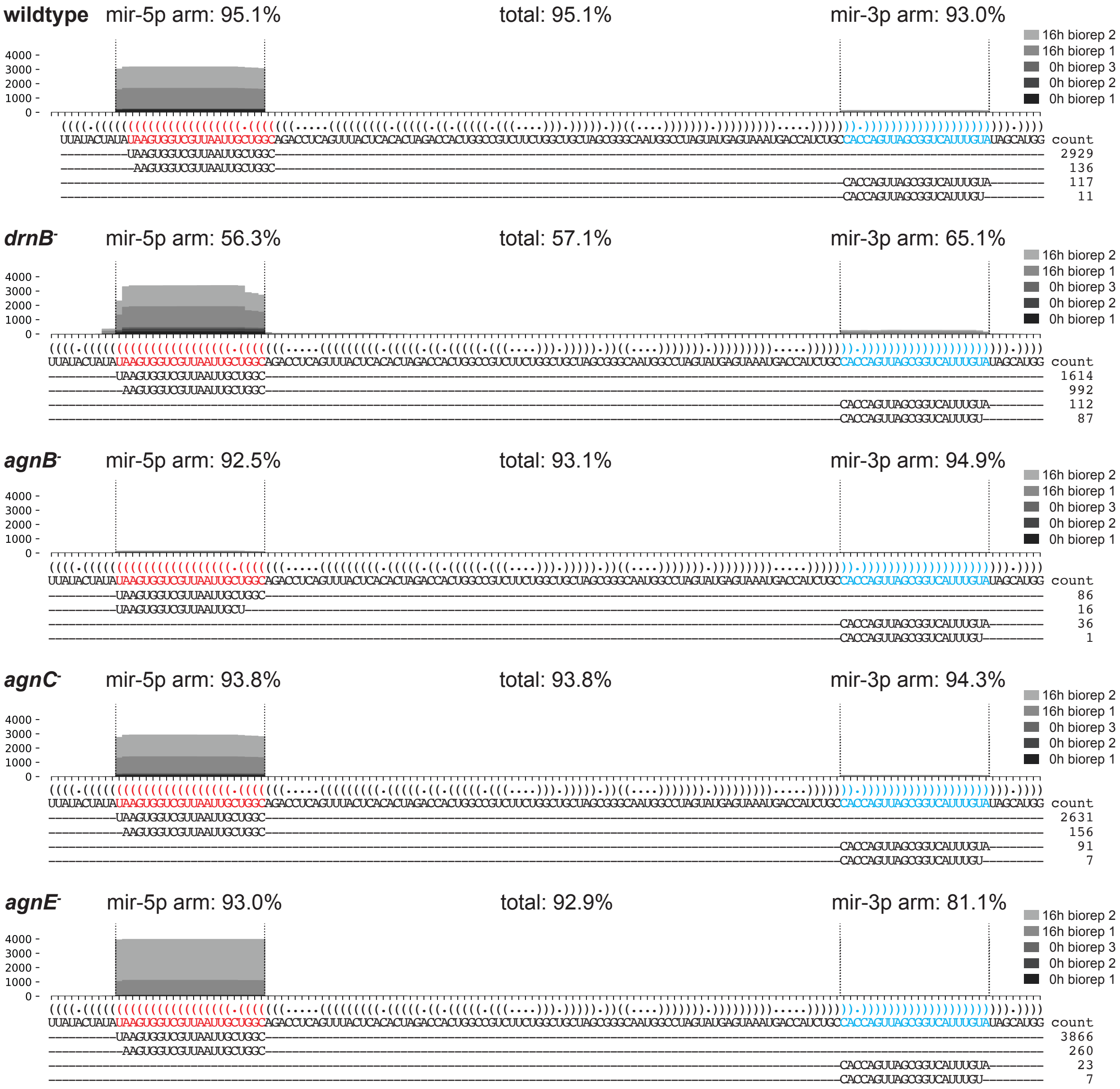

Supplementary Fig. 10 | ddi-mir-1183 abundance and processing in different RNAi machinery knockouts. Mapping of small RNA reads from indicated strains, five replicates each, to ddi-mir-1183 hairpin sequence. Histogram above the sequence illustrates the read distribution of all replicates, each with a distinct shade. Mapping precision for the mir-5p arm, mir-3p arm and total hairpin sequence above the sequence for each strain. The two most abundant reads mapping to each arm are shown below the hairpin sequence, with summed count from all replicates shown right.

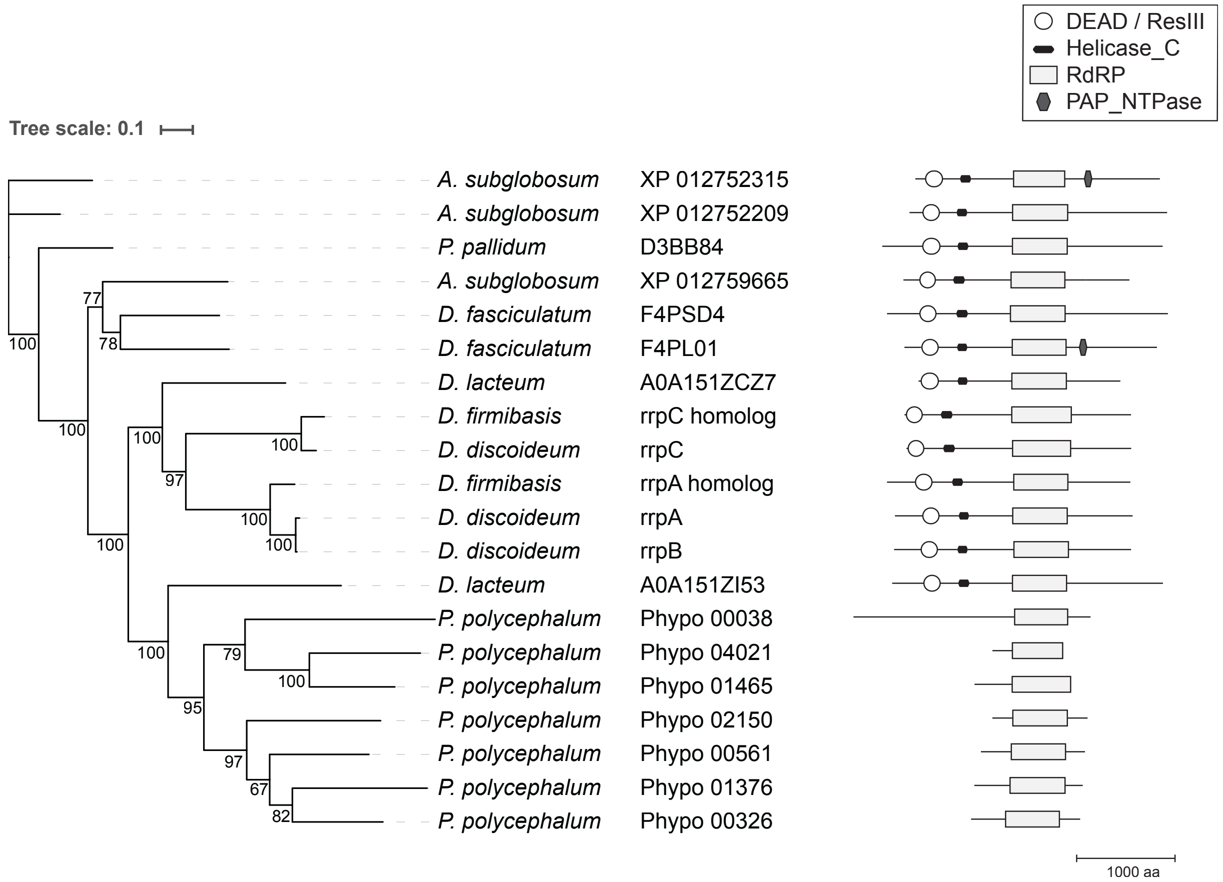

Supplementary Fig. 11 | Phylogeny of the RNA-dependent RNA polymerases in Amoebozoa. RNA-dependent RNA polymerase (RdRP) homologs identified through search with OrthoFinder (Emms and Kelly, 2019) on the proteomes of all included species, except *D. firmibasis* and *A. lenticulata* for which no proteomes are available. Homologs for *D. firmibasis* were annotated by an additional BLAST search. No homologs could be identified for *A. castelllanii* and *A. lenticulata*. Numbers after species names are the protein identifiers. For *D. discoideum*, *D. lacteum*, *P. pallidum*, *A. subglobosum* and *D. fasciculatum*, proteins can be accessed at UniProt. The domain structure of the homologs is shown (right). All identified RdRPs feature a RdRP domain. Identified ResIII/DEAD box helicase domains (ResIII/DEAD) and Helicase c-terminal domains (Helicase_C) are shown as well. Additionallly, two RdRPs contain a poly(A) polymerase nucleotidyltransferase domain (PAP_NTPase). The amino acid sequences of the identified RdRPs were aligned, trimmed and used to build a phylogenetic tree. The consensus tree is shown with bootstrap supports (%) on the branches. The scale shows evolutionary distance, defined as the number of nucleotide substitutions per site.
